# Chemical Evolution and Biological Evaluation of Natural Products for Efficient Therapy of Acute Lung Injury

**DOI:** 10.1002/advs.202305432

**Published:** 2023-12-21

**Authors:** Chengcheng Fan, Zeyi Zhang, Zhencheng Lai, Yanzi Yang, Jiaming Li, Lei Liu, Siyu Chen, Xueping Hu, Huajun Zhao, Sunliang Cui

**Affiliations:** ^1^ Institute of Drug Discovery and Design College of Pharmaceutical Sciences National Key Laboratory of Advanced Drug Delivery and Release Systems Zhejiang University 866 Yuhangtang Road Hangzhou 310058 China; ^2^ College of Pharmaceutical Sciences Zhejiang Chinese Medical University Hangzhou 311402 China; ^3^ Institute of Molecular Sciences and Engineering Institute of Frontier and Interdisciplinary Science Shandong University Qingdao 266237 China; ^4^ Jinhua Institute of Zhejiang University Jinhua Zhejiang 321299 China

**Keywords:** acute lung injury, anti‐inflammatory, heterocycle, multi‐component reaction, natural product

## Abstract

Acute lung injury (ALI) is one of the most common complications in COVID‐19 and also a syndrome of acute respiratory failure with high mortality rates, but lacks effective therapeutic drugs. Natural products provide inspiration and have proven to be the most valuable source for bioactive molecule discovery. In this study, the chemical evolution of the natural product Tanshinone IIA (Tan‐IIA) to achieve a piperidine‐fused scaffold through a synthetic route of pre‐activation, multi‐component reaction, and post‐modification is presented. Through biological evaluation, it is pinpointed that compound **8b** is a standout candidate with remarkable anti‐inflammation and anti‐oxidative stress properties, coupled with low toxicity. The mechanistic study unveils a multifaceted biological profile of **8b** and shows that **8b** is highly efficient in vivo for the treatment of ALI. Therefore, this work not only provides an effective strategy for the treatment of ALI, but also offers a distinctive natural product‐inspired drug discovery.

## Graphical abstract

Acute lung injury (ALI) is an acute respiratory failure with high mortality rates, but lacks effective therapy. Herein, a chemical evolution of natural products for efficient therapy of ALI is reported. A series of Tanshinone IIA‐inspired compounds are achieved through a concise synthesis. The biological evaluation identifies compound **8b** with a multiple biological profile of anti‐inflammation and anti‐oxidation as therapeutics.

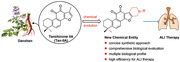

## 1 Introduction

Acute lung injury (ALI) and its more severe form, acute respiratory distress syndrome (ARDS), are complications of diverse conditions including systematic inflammation, direct injury, and infections in the lung.^[^ ^1^ ^]^ Recently, ALI/ARDS has been concerned with high incidence in different variants of severe acute respiratory syndrome coronavirus 2 (SARS‐Cov‐2)‐infected patients.^[^ ^2^ ^]^ ALI carries a devastatingly high mortality rate, and unfortunately, no effective and specialized therapeutic drug could markedly ameliorate ALI, thus making it an urgent concern in contemporary medicine. Oxidative stress is a well‐studied mechanism involved in ALI occurrence leading to inflammatory storms.^[^ ^3^ ^]^ Therefore, targeting the pivotal pathways of oxidative stress and cytokines secretion would be a promising strategy for the development of ALI therapeutic medicine.^[^ ^4^ ^]^

**Figure 1:**
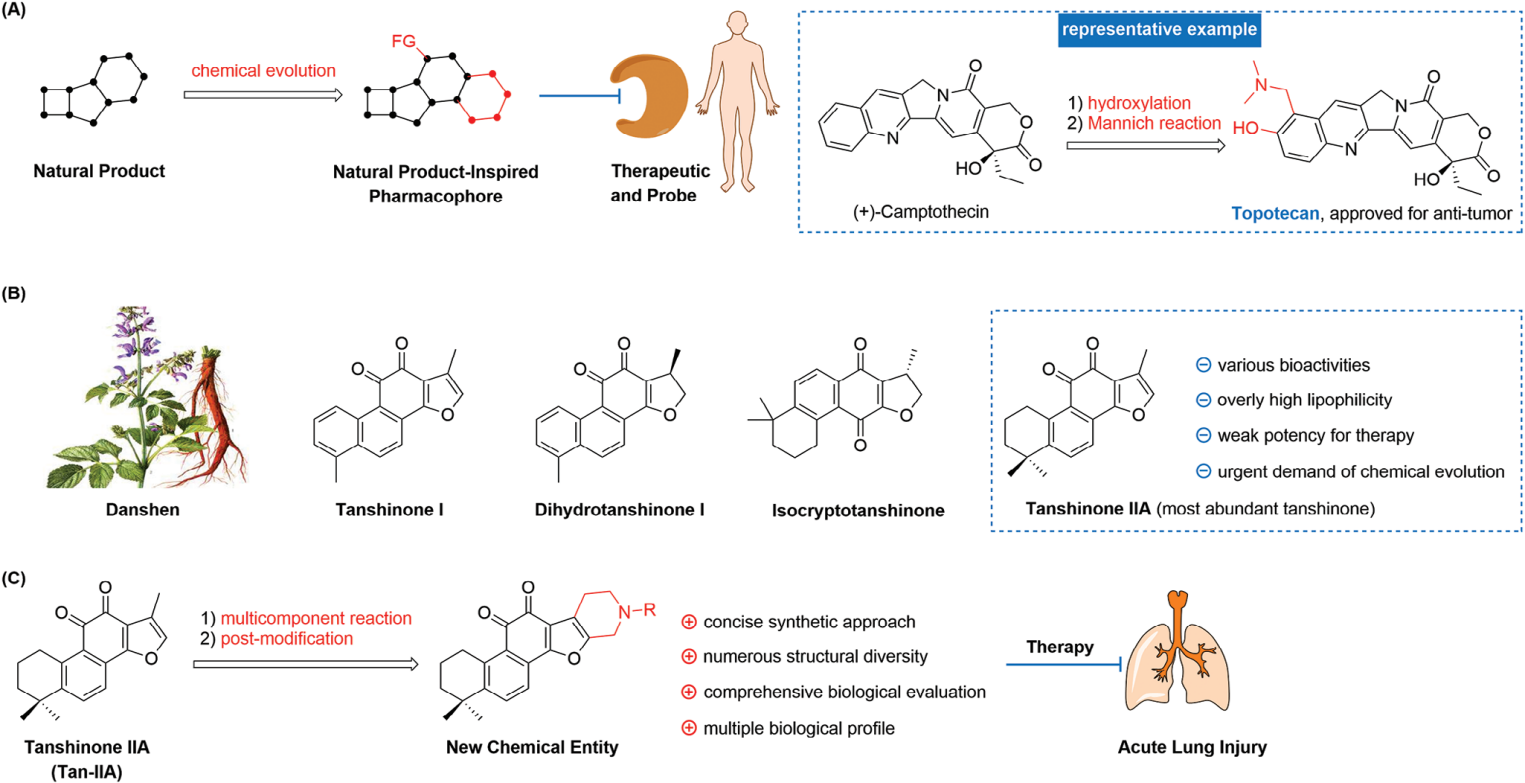
Natural product‐inspired bioactive molecule discovery. A) Representative chemical evolution strategy of natural products. B) Representative Tanshinones. C) This work: chemical evolution and biological evaluation of Tanshinone IIA for efficient treatment of ALI.

Small molecules are powerful tools for the dissections of complex biological processes due to their capacity to acutely modulate the biological targets and therefore become the dominant chemical entities to treat disease.^[^ ^5^ ^]^ In particular, natural products are the result of nature's evolutional exploration of biologically relevant chemical space and serve as an invaluable source of bioactive small molecules for chemical biology and therapeutic development.^[^ ^6^ ^]^ Pioneering strategy for the chemical evolution of natural products may enable the discovery of more potent biologically relevant small molecules (**Figure** **1**A).^[^ ^7^ ^]^ Tanshinone IIA (Tan‐IIA), the most abundant component in *Salviae miltiorrhiza* (Figure 1B), possesses a wide spectrum of bioactivities,^[^ ^8^ ^]^ including anti‐inflammation, anti‐atherosclerosis, cardio‐protection, neuro‐protection, and anti‐tumor properties.^[^ ^9^ ^]^ However, the overly high lipophilicity, poor water solubility, and weak potency of Tan‐IIA hamper it from being developed as a therapeutic probe. Therefore, it is attractive and important to implement a chemical evolution of Tan‐IIA for the discovery of more potent bioactive small molecules.

As part of our research on discovering bioactive molecules and innovative synthetic methods,^[^ ^10^ ^]^ herein, we present a remarkable chemical evolution of the natural product Tan‐IIA, aimed at gaining efficient anti‐inflammation and anti‐oxidative stress therapeutic candidate of ALI treatment (Figure 1C). In this protocol, we have developed a concise synthetic route encompassing pre‐activation, multi‐component reaction, and post‐modification of Tan‐IIA. The in vitro biological evaluation showed that these new structure chemical entities possess commendable biological activities, accompanied by low toxicity. Using RNA‐sequencing analysis, label‐free proteomic analysis, and experimental validation, we demonstrate that the lead compound **8b** exhibits a multifaceted biological profile that is highly relevant to the treatment of ALI.

## 2 Results

### 2.1 Chemical Evolution of Tanshinone IIA for New Structure Chemical Entities

**Figure 2:**
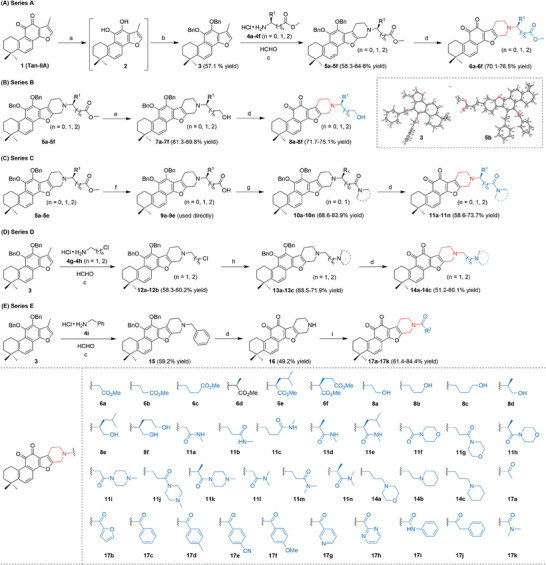
Synthetic routes: A) Pd/C, H_2_, DMF; B) Cs_2_CO_3_, DMF, BnCl, 50 °C; C) **4**, HCHO, AcOH, 90 °C; D) Pd/C, H_2_, THF, then open under air; E) LiAlH_4_, THF; F) LiOH, THF/MeOH; G) amino hydrochloride, HATU, DIPEA, DCM; H) amine, CH_3_CN; I) carboxylic acid, HATU, DIPEA, DCM.

Tan‐IIA (**1**) was preactivated via hydrogenation to give intermediate **2**, and **2** was directly benzylated upon treatment with Cs_2_CO_3_ and BnCl leading to **3** whose structure is confirmed by X‐ray analysis.^[^ ^11^ ^]^ **3** was subjected to the multi‐component reaction (MCR) with various amino acid derivatives and formaldehyde to achieve the piperidine‐fused scaffold, and there were five series compounds prepared based on this scaffold. For series A, a variety of α‐, β‐, γ‐amino acids derivatives were subjected to this MCR process to deliver intermediates **5a**–**5f**, including glycine, 3‐aminopropanoic acid, 4‐aminobutanoic acid, alanine, glutamic acid, phenylalanine, tyrosine, leucine, isoleucine and valine. The structure of compound **5b** was confirmed by X‐ray analysis.^[^ ^12^ ^]^ The following de‐benzylation and oxidation upon air exposure of **5** would recover the quinone moiety to deliver new structure compounds **6a**–**6f**. For series B, compound **5** was reduced by LiAlH_4_ to deliver alcoholic compounds **7**, which were de‐benzylated and oxidized under air to deliver compounds **8a**–**8f**. For series C, compound **5** was hydrolyzed to acid **9** upon treatment with LiOH, and the following ligation of **9** with amines, de‐benzylation, and oxidation upon air exposure would give compounds **11a**–**11n**. For series D, **3** was subjected to the MCR with amino hydrochloride **4g**‐**4 h** and formaldehyde to yield intermediates **12a**‐**12b**, which were further modified by functionalized amines to deliver **13a**–**13c**; and the similar de‐benzylation would afford products **14a**–**14c**. For series E, **3** was subjected to the MCR with benzylamine hydrochloride **4i** and formaldehyde to yield intermediate **15**, which was de‐benzylated to furnish intermediate **16**, and the sequential ligation with various carboxylic acids would provide products **17a**–**17k**. Upon this chemical evolution, the overly high lipophobic Tan‐IIA was transformed into new structural heterocyclic chemical entities, and there was a total of 40 compounds synthesized (**Figure** **2**).

### 2.2 In Vitro Anti‐Inflammatory Evaluation

**Figure 3:**
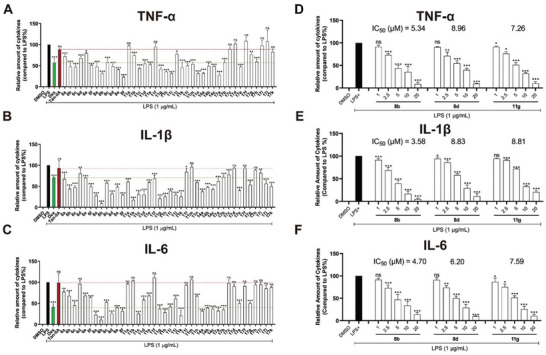
Anti‐inflammatory evaluation of the new structure chemical entities. A–C) The inhibitory effect of compounds on the release of TNF‐α, IL‐6, IL‐1β at a concentration of 10 µm. D–F) The IC_50_ value of the lead compounds inhibits the secretion of TNF‐α, IL‐1β, and IL‐6. Data are normalized to respective controls. All data were presented as means ± SD (*n* = 3; * *p* < 0.05, ** *p* < 0.01, *** *p* < 0.001, and ns: no significant vs LPS).

Blocking the excessive production of cytokines represents a promising strategy to prevent and treat ALI. These new structure compounds were first subjected to anti‐inflammatory evaluation. In the context of the inflammatory response, the release of pro‐inflammatory cytokines would trigger inflammatory progress and the main cytokines include tumor necrosis factor‐α (TNF‐α), interleukin‐6 (IL‐6), and interleukin‐1β (IL‐1β).^[^ ^13^ ^]^ We selected activated mice peritoneal macrophages (PMs) for in vitro evaluation, utilizing enzyme‐linked immunosorbent assay (ELISA) (**Figure** **3**A). The PMs were initially pre‐treated with compounds at a concentration of 10 µm for 1 h and subsequently stimulated with 1 µg mL^−1^ LPS for an additional 24 h, wherein Dexamethasone (Dex) and Tan‐IIA were used as positive controls. As shown in Figure 3, Tan‐IIA barely showed cytokines release inhibition activities at 10 µm, while many of the new structure compounds exhibited robust inhibition effects for the secretion of TNF‐α, IL‐1β, and IL‐6. In particular, several hit compounds were selected to characterize the IC_50_ values for the secretion suppression of these three cytokines. For instance, **8b**, **8d,** and **11** **g** were characterized with IC_50_ values of 5.34, 8.96, and 7.26 µm for TNF‐α suppression; IC_50_ values of 3.58, 8.83, and 8.81 µm for IL‐1β suppression; and IC_50_ values of 4.70, 6.20, and 7.59 µm for IL‐6 suppression. Besides, cytotoxicity should be considered in the process of natural product evolution and drug discovery. Thus, these effective compounds were subjected to the cytotoxicity assay on PMs at a concentration of 10 µm, and most of them showed low toxicity (see Figure S3, Supporting Information). In the case of compound **8b**, no obvious cytotoxicity was observed even at a concentration of 50 µm. Taking together, the in vitro anti‐inflammation evaluation demonstrated the success of the chemical evolution of Tan‐IIA, and **8b** was chosen as the lead compound for further evaluation.

**Figure 4:**
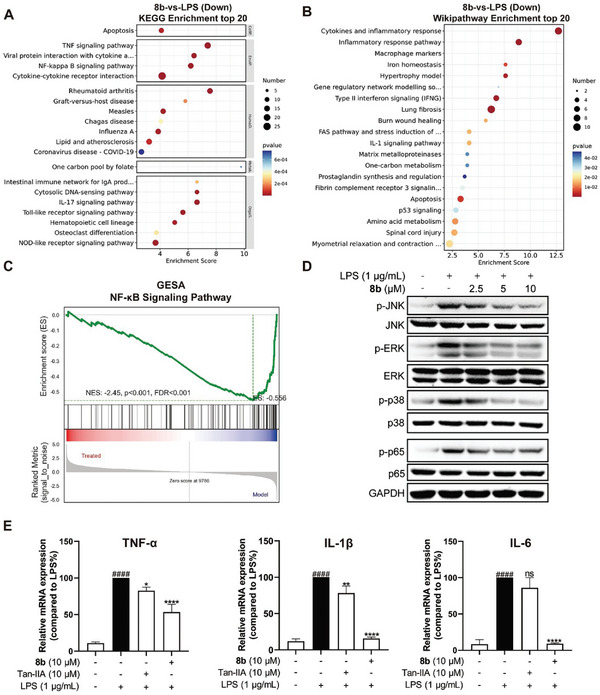
Effect of **8b** on LPS (1 µg mL^−1^)‐induced gene expression. A) KEGG enrichment analysis of the down‐regulated genes upon **8b** (10 µm) treatment for 24 h identified by RNA‐seq in RAW264.7 macrophages. B) Wikipathway enrichment analysis of the down‐regulated genes. C) Gene Set Enrichment Analysis (GSEA) of NF‐κB pathway. D) **8b** inhibited the phosphorylation of MAPKs and NF‐κB in a dose‐dependent manner. E) Effects of **8b** on the LPS‐induced mRNA expression of inflammatory genes. Data are normalized to respective LPS groups. All data were presented as mean ± SD (*n* = 3; * vs LPS, ^#^ vs Con, * *p* < 0.05; ** *p* < 0.01; ^****^ *p* < 0.0001; ^####^ *p* < 0.0001; ns, no significant vs LPS).

To identify the molecular pathways involved in the anti‐inflammation effect of **8b**, we conducted an RNA sequencing (RNA‐seq) analysis to explore the obviously changed genes by treatment of **8b** on RAW264.7 macrophages. In total, there were 795 differentially expressed genes (DEGs), including 404 up‐regulated DEGs and 391 down‐regulated DEGs when comparing the LPS + **8b** group and LPS‐only group (Figure S4, Supporting Information). In particular, the KEGG pathway enrichment analysis revealed that the gene expressions with significant differences were mainly enriched in the signaling pathways including TNF, NF‐κB (Nuclear factor‐κB), Cytokine‐cytokine receptor interaction, IL‐17, Toll‐like receptor, Nod‐like receptor, and other signaling pathways, these pathways are all highly relevant to inflammation progression (**Figure** **4**A). We also conducted a Wikipathway enrichment analysis and the result revealed that the different expressed genes were mainly enriched in the Cytokines and inflammatory response, Inflammatory response pathway (Figure 4B). Notably, treatment with **8b** in LPS‐stimulated macrophages could down‐regulate lung fibrosis‐related genes, which is mainly caused by pneumonia and damage to lung necrosis. The gene set enrichment analysis (GSEA) demonstrated **8b** down‐regulated NF‐κB signaling pathway in LPS‐stimulated macrophages (Figure 4C).^[^ ^14^ ^]^ NF‐κB is a crucial pro‐inflammatory signaling pathway and usually serves as a therapeutic target in the treatment of inflammatory and auto‐immune diseases. NF‐κB can be activated by LPS to act as a transcription factor and induce the expression of different cytokines, including TNF‐α, IL‐1β, and IL‐6. We also used Western blotting and reversed transcription‐polymerase chain reaction (RT‐PCR) analysis to validate the results of RNA‐seq. **8b** treatment could significantly alleviate the phosphorylation of MAPKs (including the JNK, ERK, and p38 kinases) and NF‐κB in RAW264.7 macrophages in a dose‐dependent manner (Figure 4D), and the result of RT‐PCR revealed that **8b** could potently decrease the LPS up‐regulated mRNA levels of these cytokines at a concentration of 10 µm (Figure 4E). All these results indicated that suppressing the NF‐κB signaling pathway is the basis for **8b** to alleviate LPS‐induced inflammation in macrophages.

### 2.3 Anti‐Oxidative Stress Effect of 8b in Macrophages

**Figure 5:**
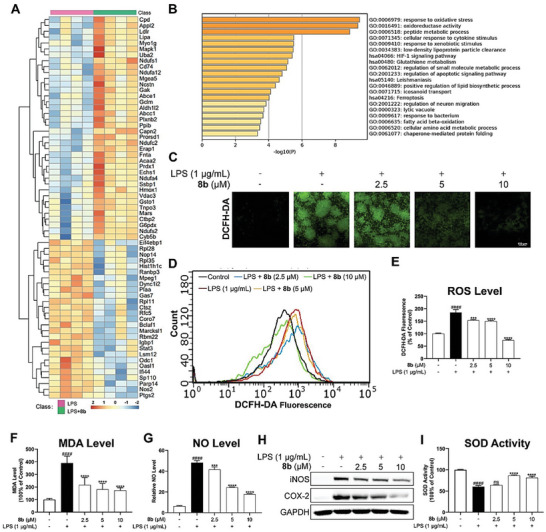
Effect of **8b** on LPS‐induced oxidative stress. A) Heatmap of regulated proteins upon **8b** (10 µm) treatment for 24 h identified by label‐free proteomics analysis in RAW264.7 cells. B) Bioinformatic analysis of regulated proteins. C) Intracellular ROS level was observed by fluorescence microscope. D,E) The fluorescence intensity of DCFH‐DA in each group was measured by flow cytometry. F) The levels of MDA in cells were determined. G) The levels of NO in cells were determined. H) The protein levels of iNOS and COX‐2 were determined by Western Blotting analysis. I) Intracellular SOD activity was determined. Data for E), F), and I) are normalized to respective controls. All data were presented as mean ± SD (*n* = 3; * vs LPS, ^#^ vs Con, * *p* < 0.05; ** *p* < 0.01; ^****^ *p* < 0.0001; ^####^ *p* < 0.0001; ns, no significant vs LPS).

Inflammatory reactions are usually driven by oxidative stress and in ALI occurrence and progress, inflammatory cells induce the excessive production of reactive oxygen species (ROS), establishing a vicious cycle that exacerbates ALI development.^[^ ^15^ ^]^ Therefore, blocking the key pathway of oxidative stress and associated inflammation presents a capable strategy for treating ALI. Having shown that **8b** could effectively reduce pro‐inflammatory cytokines secretion in LPS‐stimulated macrophages, we hypothesized that **8b** might alleviate oxidative stress. At this stage, a label‐free proteomic analysis was conducted. The Heatmap showed that there were 38 proteins up‐regulated and 27 proteins down‐regulated in the **8b** + LPS group, compared with the LPS‐only group (**Figure** **5**A). The bioinformatics analysis indicated that **8b** treatment could potently activate cell responses to oxidative stress (Figure 5B). Oxidative stress occurs when ROS production surpasses its clearance. We stained RAW264.7 cells with a DCFH‐DA probe to measure the ROS level, LPS treatment induced an increase in green fluorescence, indicating the accumulation of ROS in cells, while **8b** pre‐treatment for 1 h significantly reduced the production of ROS (Figure 5C–E). Additionally, levels of malondialdehyde (MDA), NO, and proteins iNOS, and COX‐2 were elevated in LPS‐treated cells and **8b** pre‐treatment could reduce these levels. (Figure 5F–H). Finally, we found that the LPS‐inhibited activity of Superoxide Dismutase (SOD) could be recovered by **8b** (Figure 5I). Combining proteomic analysis and experimental verification, we demonstrated that **8b** effectively ameliorated oxidative stress in LPS‐stimulated macrophages.

### 2.4 8b Plays an Anti‐Oxidant Role by Activating Nrf2

**Figure 6:**
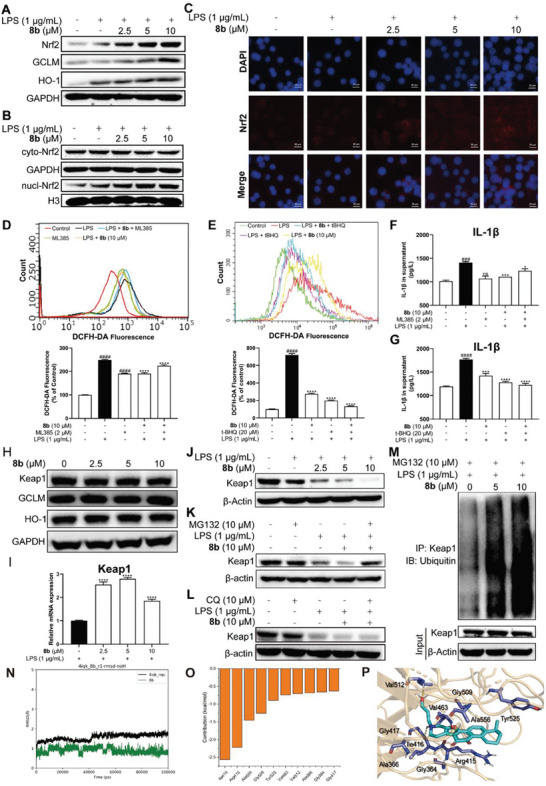
**8b** activates Nrf2 by inducing degradation of Keap1. A) The protein levels of Nrf2, GCLM, and HO‐1 in cells were determined by Western Blotting analysis. B) The protein level of Nrf2 in the nucleus and cytoplasm. C) Macrophages were subjected to immunofluorescence staining of Nrf2. Scale bar, 20 µm. D) The fluorescence intensity of DCFH‐DA in **8b** and ML385 co‐treated cells was measured by flow cytometry. E) The fluorescence intensity of DCFH‐DA in **8b** and t‐BHQ co‐treated cells was measured by flow cytometry. F‐G) IL‐1β level in the supernatant of each group was determined using an ELISA kit. H) **8b** itself did not change the protein levels of Keap1 and the downstream antioxidative proteins without LPS stimulation. I) mRNA level of Keap1 was detected by RT‐PCR analysis. J) **8b** decreased protein level of Keap1 in cells. K,L) The protein level of Keap1 was detected in cells after co‐treatment with **8b** and MG132 (K) or CQ (L). M) The protein levels of Ubiquitin and Keap1 in cells were determined after co‐treatment with **8b** and MG132. N) The timing evolution of the RMSDs of the heavy atoms of **8b** and Keap1. O) The 10‐top ranked residues in Keap1 are responsible for the binding of **8b** predicted by MM/GBSA. P) The structural analysis of the 10 top‐ranked residues to the binding of **8b**. Data for (D) and (E) are normalized to respective controls. All data were presented as means ± SD (*n* = 3; * vs LPS, ^#^ vs Con, * *p* < 0.05; *** *p* < 0.001; ^****^ *p* < 0.0001; ^####^ *p* < 0.0001; ns, no significant vs LPS).

According to the results of proteomics, **8b** could mainly regulate the expression of proteins that respond to oxidative stress. Thus, we speculated that the anti‐oxidative stress effect of **8b** should be related to the nuclear factor‐erythroid 2‐related factor 2 (Nrf2). The Nrf2 signaling pathway is one of the most critical endogenous antioxidant defense systems that is implicated in the pathogenesis of oxidative stress‐related diseases. At homeostasis, Nrf2 is maintained in an inactive state in the cytosol by associated with its endogenous regulator Kelch‐like epichlorohydrin‐related protein (Keap1), which directs Nrf2 for proteasomal degradation. Upon oxidative stress conditions, Keap1 is inactivated, allowing Nrf2 to initiate Nrf2‐responsive and antioxidant response element (ARE)‐dependent genes.^[^ ^16^ ^]^ In order to investigate the effect of **8b** on Nrf2, the Western blotting analysis was conducted and revealed that **8b** could up‐regulate the protein level of Nrf2 and important anti‐oxidant downstream members, such as GCLM and HO‐1 (**Figure** **6**A). Moreover, **8b** promoted nuclear translocation of Nrf2 was also detected (Figure 6B,C). To further verify the **8b** activated cell antioxidative system through affecting Nrf2, the Nrf2 inhibitor ML385 and activator t‐BHQ were used to co‐treat with **8b** in RAW264.7 cells, and the production of ROS was detected. **8b** decreased ROS production in LPS‐stimulated cells, and this effect could be reversed by ML385 and enhanced by t‐BHQ (Figure 6D,E). Meanwhile, the inhibition effect of **8b** for LPS‐induced IL‐1β secretion in cells was also reversed by ML385, but promoted by t‐BHQ. (Figure 6F,G). These findings suggested that the inhibitory effects of **8b** on the release of pro‐inflammatory cytokines and anti‐oxidative stress activity are derived from the activation of Nrf2.

To explore whether **8b** activates Nrf2 from its effect on Keap1, we first detected the protein and mRNA levels of Keap1. According to the results, **8b** decreased the protein level of Keap1 without inhibiting its mRNA level (Figure 6I,J), indicating there exists a post‐translation modification mechanism. To explore the pathway of **8b**‐induced Keap1 degradation, we applied MG132 (a proteasome inhibitor) and CQ (a lysosomal inhibitor) to co‐treat Raw264.7 cells with **8b**, and then collected cells for Western blotting analysis. The results showed that MG132 could effectively rescue the degradation of Keap1 induced by **8b**, while CQ could not, suggesting that the **8b**‐induced Keap1 degradation was achieved through a ubiquitination pathway rather than an autophagy‐lysosome pathway (Figure 6K–M). Notably, **8b** itself did not alter the protein level of Keap1 or downstream antioxidative proteins of Nrf2 without LPS stimulation (Figure 6H), indicating that **8b** only plays an antioxidative role when macrophages were stimulated by pathogen‐associated molecular patterns.

To gain insights into the interaction between Keap1 (PDB ID: 4IQK) and **8b**, molecular docking, and molecular dynamics (MD) simulations were performed. As shown in Figure 6N, the binding mode of compound **8b** was quite stable during the 100 ns MD simulation, with the root‐mean‐square deviations (RMSDs) fluctuations within 1 Å. The 10 top‐ranked residues for the binding of **8b** to the Keap1 predicted by the per‐residue decomposition of the Molecular Mechanics/Generalized Born Surface Area (MM/GBSA) approach were Ile416, Arg415, Ala556, Gly509, Tyr525, Val463, Val512, Ala366, Gly364, and Gly417. The structural analysis indicates that the side chain of *N*‐piperidine can be inserted into the deeper pockets of the protein, and forms hydrogen bond interactions with Ile416 and Val 512. The scaffold framework of compound **8b** forms hydrophobic interactions with Arg415, Ala556, Gly509, Tyr525, Val463, and Gly364. The structural analysis suggested the possibility that **8b** promoted Keap1 degradation through direct binding to its kelch domain,^[^ ^17^ ^]^ thereby preventing Keap1/Nrf2 protein‐protein interaction and protecting Nrf2 induction from the effect of protein ubiquitination. Taken together, **8b** could effectively alleviate oxidative stress by promoting the activities of Nrf2 and downstream protein.

### 2.5 In Vivo Evaluation for ALI Therapy

**Figure 7:**
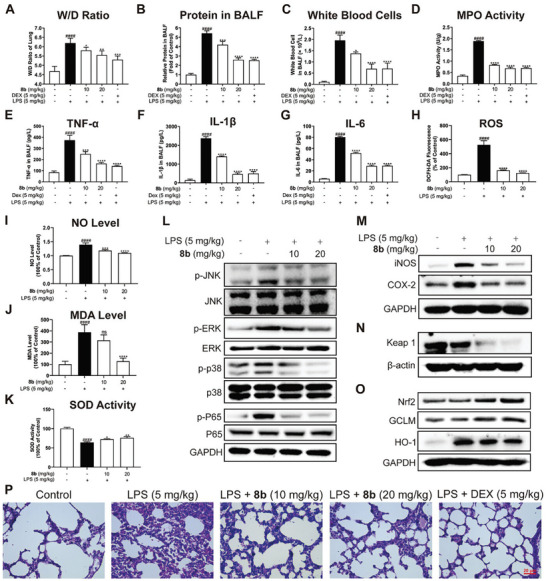
In vivo evaluation of compound **8b** for ALI treatment. A) Wet/dry ratio. B) Total protein concentration in BALF. C) Number of white blood cells in BALF. D) MPO activity in lung tissues. E) The amount of TNF‐α in BALF. F) The amount of IL‐1β in BALF. G) The amount of IL‐6 in BALF. H) ROS level in BALF. I) NO level in BALF. J) MDA level in lung tissues. K) SOD activity in lung tissues. L) **8b** inhibited MAPK and NF‐κB phosphorylation in lung tissues. M) The protein levels of iNOS and COX2 were determined by Western blotting analysis. N) The protein level of Keap1 in lung tissues. O) The protein levels of Nrf2 and HO‐1, GCLM were determined by Western blotting analysis. P) Representative images of lung H&E staining of Control, LPS, and **8b** treatment groups. Data for (H–J) are normalized to respective controls. All data were presented as means ± SD (*n* = 6; * vs LPS, ^#^ vs Con, * *p* < 0.05; ** *p* < 0.01; *** *p* < 0.001; ^****^ *p* < 0.0001; ^####^ *p* < 0.0001; ns, no significant vs LPS).

Since compound **8b** showed a multiple biological profile of anti‐oxidation and anti‐inflammation, which are highly relevant to ALI, **8b** was then subjected to the in vivo therapeutic evaluation in the ALI model.^[^ ^18^ ^]^ First, the in vivo pharmacokinetic (PK) profile was conducted for **8b**, and the parameters are listed in Table S2 (Supporting Information). The data showed that **8b** possessed an acceptable intraperitoneal injections (IP) bioavailability. Besides, **8b** was administrated with IP at the dosage of 10 mg kg^−1^, and its distribution in lung tissue was also detected. The results showed that the lung drug concentration is 9160 ng g^−1^ at 0.25 h, indicating that lung accumulation of **8b** is suitable for treating ALI. In the LPS‐induced ALI model, there would be inflammation disease accompanied by lung edema, inflammatory cell infiltration, pulmonary congestion, distinctive alveolar wall thickening, and alveolar structure destruction.^[^ ^19^ ^]^ The lung wet/dry ratio (W/D) and concentration of total proteins were dramatically increased in mice bronchial alveolar lavage fluid (BALF) to indicate the increased pulmonary capillary permeability in the LPS group. In contrast, treatment with **8b** via intraperitoneal injection at a dose of either 10 or 20 mg kg^−1^ could remarkably decrease W/D and protein content (**Figure** **7**A,B). Additionally, treatment with **8b** also significantly reduced the number of white blood cells in BALF (Figure 7C). Besides, myeloperoxidase (MPO) is an indicator of neutrophil aggregation and a pre‐treatment of **8b** significantly inhibited the increase of MPO activity induced by LPS (Figure 7D).

Consistent with this phenotype result, the levels of inflammatory cytokines like TNF‐α, IL‐1β, and IL‐6 and the corresponding mRNA levels in BALF were significantly decreased by **8b** pre‐treatment (Figure 7E–G; Figure S5, Supporting Information). Furthermore, **8b** was found to effectively inhibit the phosphorylation of MAPKs (JNK, ERK, and p38 kinases) and NF‐κB in the lung tissue of mice (Figure 7L). Meanwhile, the NO level in BALF, and the ROS, and MDA levels in lung tissues were up‐regulated by LPS stimulation and reversed by treatment of **8b** (Figure 7H–J). SOD activity was also rescued by **8b** (Figure 7K). The Western blotting analysis showed that **8b** pre‐treatment could downregulate the expression of iNOS and COX‐2, and also promote the Keap1 degradation to activate the expression of Nrf2, GCLM, and HO‐1 (Figure 7M–O).

In addition, the morphological changes of lung tissues were observed by light microscope, wherein the LPS injected mice exhibited similar pathological features to ALI with thickened alveolar and infiltration of inflammatory cells. In contrast, when mice were treated with **8b**, the alveolar structures were relatively intact, and the inflammatory cell infiltrations were obviously reduced along with mild alveolar thickening (Figure 7P). Notably, after treatment on ICR rat with a single intraperitoneal injection of **8b** at 200 mg kg^−1^, there was no obvious toxic reaction in 7 days and the H&E staining results suggested that **8b** did not cause significant tissue damage and inflammatory response (Figure S6, Supporting Information). Collectively, these findings indicated that **8b** could be effective in the ALI treatment in vivo.

## 3 Discussion

ALI is a complex pulmonary destructive disease with limited therapeutic approaches. Despite numerous clinical studies, ALI remains a severe disorder with high mortality and morbidity. Natural products have always inspired drug discovery, and many reports have highlighted the pivotal role of natural compounds as therapeutic agents. Particularly, Tan‐IIA possesses a variety of biological activities including anti‐inflammatory effects but suffers from poor drug‐likeness and weak potency. Consequently, the chemical evolution of Tan‐IIA toward lead compound discovery is important and attractive. For example, Zhang and Liang et al. reported a scaffold hybrid of Tan‐IIA with Salviadione as a protective anti‐inflammatory agent for ALI with improved physicochemical properties and pharmacokinetics.^[^ ^14^ ^]^ Meanwhile, the Mannich alkylamination at the C‐15 position of Tan‐IIA can introduce amino and amide functionality to improve drug‐likeness and potency. Diverging from the prior modification of Tan‐IIA, we used our seminal developed double Mannich alkylamination reaction to construct Tan‐IIA‐piperidine scaffold hybrid compounds. In this process, a variety of functionalities and chiral fragments could be directly incorporated into the products, while the vital pharmacophore of *ortho*‐quinone was retained. Upon this new evolution strategy, the overly high lipophilic Tan‐IIA could be easily transformed into *N*‐heterocyclic derivatives. Thus, these natural product‐inspired new structure chemical entities were easily achieved to provide ample opportunity for biological exploration.

**Figure 8:**
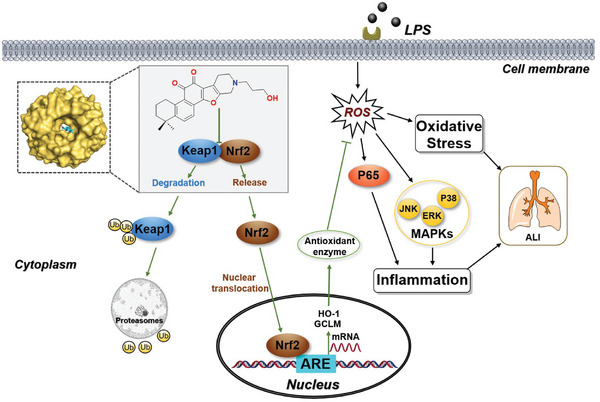
8b regulates Keap1/Nrf2 signaling pathway and alleviates oxidative stress and inflammation for ALI therapy.

Studies have suggested that when ALI is triggered by pathogen‐associated molecular patterns (PAMPs), such as LPS, excessive ROS will accumulate in macrophages. Afterward, two mutually reinforcing pathological events unfold, oxidative stress amplifies pro‐inflammatory gene expression, while inflammatory cytokines induce the production of ROS. The synergistic effect between oxidative stress and inflammatory response is a well‐accepted mechanism involved in ALI occurrence and progress. After obtaining the new structural compounds, we evaluated their bioactivity against ALI. Firstly, these compounds were subjected to anti‐inflammatory evaluation. Phenotypic screening of these compounds revealed that compound **8b** could significantly suppress the secretion of the main cytokines including TNF‐α, IL‐1β, and IL‐6, with IC_50_ values of 5.34, 3.58, and 4.70 µm, respectively. Besides, **8b** did not find any significant cytotoxicity even at 50 µm against mice peritoneal macrophages (PMs). These results inspired us to continue further research. In order to identify the molecular pathways involved in the anti‐inflammation of **8b**, an RNA‐sequencing analysis was performed on macrophage cells, and the results showed that **8b** down‐regulated expression genes with significant differences were mainly enriched in the inflammation progression‐related signaling pathways. Consistent with the results of phenotypic screening and RNA‐seq, the mRNA levels of TNF‐α, IL‐1β, and IL‐6 were down‐regulated in LPS‐stimulated cells upon **8b** treatment. Western blotting analysis further confirmed that the level of phosphorylated MAPKs (including the JNK, ERK, and P38 kinases) and NF‐κB were reduced by **8b** treatment. Therefore, the anti‐inflammation evaluation demonstrated that the success of the chemical evolution of Tan‐IIA and **8b** was chosen for further evaluation. Secondly, a label‐free proteomic analysis was conducted and bioinformatic analysis indicated that **8b** treatment activated cell response to oxidative stress. The elevated levels of ROS, NO, and MDA in LPS‐stimulated macrophages were found significantly decreased by pre‐treatment of **8b** and the LPS‐inhibited activity of Superoxide Dismutase (SOD) could be recovered. Therefore, combining proteomic analysis and experimental verification, we demonstrated that **8b** could effectively alleviate the oxidative stress in LPS‐stimulated macrophages. This biological aspect of **8b** is derived from the activation of Nrf2. The Western blotting analysis disclosed that **8b** promoted the nuclear translocation of Nrf2 and activated downstream anti‐oxidant members, GCLM and HO‐1. Since the degradation of Keap1 is the base of Nrf2 release and nuclear translocation, we further explored the effect of **8b** on Keap1. The results showed that **8b** could not inhibit the mRNA level of Keap1 but induce Keap1 degradation via a ubiquitination pathway. On the other hand, it is well known that MAPK represents one of the most redox‐sensitive signaling pathways. Increased ROS can sustainably activate the MAPK pathway by activating MAPK kinase and inhibiting MAPK phosphatase. Over‐phosphorylated MAPK can amplify NF‐κB release, transfer NF‐κB into the nucleus, and further activate the inflammatory response. Meanwhile, ROS can activate NF‐κB directly by replacing IκBα phosphorylation, which leads to degradation of IκBα and enhance NF‐κB DNA binding. Among the processes regulating oxidative stress, inhibiting the function of Keap1 to reduce the degradation of Nrf2 is one of the most important endogenous antioxidative regulatory mechanisms. Hence, the Keap1/Nrf2 signaling pathway has always been an important drug target for the treatment of oxidative stress‐related diseases. Based on the series of examination results, a scheme for a comprehensive summary of the mode of action of **8b** in this study is given in **Figure** **8**. We speculate that **8b** exerts antioxidative effects by activating Nrf2 and experiments confirmed that **8b** could induce the degradation of Keap1, activate Nrf2, clear ROS, and consequently lead to the inhibition of MAPK and NF‐κB signaling pathways. According to molecular docking studies, **8b** could fit well into the pockets of the Keap1 protein by generating hydrogen‐bond and hydrophobic interactions.

At last, the in vivo evaluation in the LPS‐induced ALI model showed that **8b** could effectively decrease the levels of inflammatory cytokines, W/D, and protein content in the lung tissues, reduce the number of white blood cells in BALF, and inhibit the increase of MPO activity. Meanwhile, the levels of phosphorylated MAPKs (JNK, ERK1/2, and p38) and NF‐κB were decreased in the lung tissue, while the degradation of Keap1 was promoted, leading to activation of Nrf2, GCLM, and HO‐1.

In summary, we have established a chemical evolution and biological evaluation of the natural product Tan‐IIA for efficient therapy of ALI. This work not only provides an effective strategy for the treatment of ALI, but also offers a distinctive natural product‐inspired drug discovery.

## 4 Experimental Section

### Reagents and Instruments

All chemical reagents and solvents employed, unless otherwise noted, were purchased commercially and were used as provided without further purification. All anhydrous reactions were performed under an argon atmosphere using dry solvents. Flash column chromatography was carried out over silica gel (200–300 mesh). ^1^H NMR and ^13^C NMR spectra were recorded on a Bruker AV‐600 spectrometer, Bruker AV‐500 spectrometer, or a WNMR‐I‐400 spectrometer at room temperature. CDCl_3_, CD_3_OD, or DMSO‐*d*_6_ were used as a solvent, chemical shifts were referenced relative to the residual solvent. All NMR spectra were analyzed using MestReNova 10.0 program software. Multiplicity patterns were designated as follows: bs, broad singlet; s, singlet; d, doublet; t, triplet; q, quartet; m, multiplet. Coupling constants (*J*) were reported in Hertz (Hz). HRMS were performed on Agilent Technologies 6546‐LC/Q‐TOF LC/MS apparatus (ESI‐TOF). Melting points were measured with an X‐4 micro melting point apparatus. The analytical data for the final compounds are provided in Supporting Information. All tested compounds were determined to be >95% purity by HPLC. Solvent: methanol; flow rate = 1.0 mL min^−1^; wavelength, 254 nm; SHIMADZU Shim‐pack GIST, Material: 5 µm C18, Dimensions: 4.6 × 150 mm, P/N: 227‐30017‐07.

LPS, MTT, and DMSO were purchased from Sigma (MO, USA). Antibodies against Keap1 (#8047S), HO‐1 (#43966S), COX‐2 (#12282S), iNOS (#13120S), p‐p65 (#3033), p65 (#8242), p‐p38 (#4511S), p38 (#8690S), p‐ERK (#9101), ERK (#9102), p‐JNK (#4668), JNK (#9258), Ubiquitin (#3936) and GAPDH (#2118) were purchased from Cell Signaling Technology (Massachusetts, USA). Antibody against Nrf2 (abs130481) was purchased from Absin Bioscience Inc (Shang, China), antibody against GCLM (ab126704) was purchased from Abcam (England), antibody against Histone H3 (#06–599) was purchased from Merck Millipore (Merck, Germany), and antibody against β‐actin (#380 624) was purchased from Chengdu Zen Biotechnology (Chengdu, China).

### Hydrogenation and Benzylation of Tan‐IIA for the Synthesis of **3**

A two‐necked flask equipped with a magnetic stirrer bar was charged with Tanshinone IIA (2.94 g, 0.01 mol, 1 equiv.) and 10% Pd/C (0.03 g, 5% palladium on activated carbon, wetted with ≈55% water), purged with hydrogen several times, and anhydrous *N*, *N*‐dimethylformamide (DMF, 15 mL) was added. The resulting reaction mixture was stirred at room temperature until the red color disappeared. Afterward, the mixture was transferred by a syringe into another two‐necked flask charged with Cs_2_CO_3_ (13.04 g, 0.04 mol, 4 equiv.) under an argon atmosphere (take note: avoiding contact with air during transfer operations), then benzyl chloride (5.06 g, 0.04 mol, 4 equiv.) was added in one portion. The reaction solution was heated at 50 °C for 5 h. Afterward, the reaction mixture was filtered through diatomite and washed with EtOAc (100 mL). The filtrate was washed with brine to remove DMF and dried over anhydrous Na_2_SO_4_. Evaporation of the solvents afforded the crude product, which was further purified by silica gel column chromatography, eluting with Petroleum Ether/EtOAc to give compound **3**.

### Procedure of the Multicomponent Reaction for the Synthesis of **5**

A sealed tube with a magnetic stirrer bar was charged with compound **3** (0.48 g, 1 mmol), paraformaldehyde (0.24 g, 8 mmol, 8 equiv), amino acid ester hydrochloride **4** (4 mmol, 4 equiv), and anhydrous AcOH (15 mL) was added as a solvent. The reaction mixture was stirred at 90 °C and monitored by TLC. After completion, the reaction was quenched by saturated aqueous NaHCO_3_. The aqueous layer was extracted with EtOAc (50 mL × 3), and the combined organic layer was washed with brine, dried over anhydrous Na_2_SO_4_, and concentrated in vacuo. The residue was purified by silica gel column chromatography to give the intermediate **5**.

### Procedure of the De‐Benzylation to Recover Quinone Moiety for the Synthesis of **6**

A two‐necked flask equipped with a magnetic stirrer bar was charged with **5** (1 mmol) and 10% Pd/C (5% palladium on activated carbon, wetted with ca. 55% water), anhydrous THF (15 mL) was added, and purged with hydrogen several times. The resulting mixture was stirred under a hydrogen atmosphere overnight. The debenzylation and following oxidation upon air exposure of **5** would recover the quinone moiety to deliver compound **6**.

### Cell Culture

Mice primary peritoneal macrophages were collected from mice, which were intraperitoneally injected with 1 mL 3% fluid thioglycollate medium for 5 days and cultured in RPMI 1640 medium (Gibco, USA) supplemented with 10% fetal bovine serum (Gibco, USA) and 1% penicillin under an atmosphere of 5% CO_2_ at 37 °C. Murine macrophage Raw264.7 cells line was purchased from the Shanghai Institutes for Biological Sciences (Chinese Academy of Science, Shanghai) and cultured in DMEM‐High glucose growth media (Gibco, USA) with 10% fetal bovine serum (Gibco, USA), 1% penicillin and 100 µg mL^−1^ streptomycin under an atmosphere of 5% CO_2_ at 37 °C.

### MTT Assay

Cells seeded in a 96‐well plate (8 × 10^3^ cells per well) were exposed to various concentrations of compounds for 48 h. Next, 20 µL MTT (0.5%) was added per well. After incubating at 37 °C for 4 h. Then 100 µL of triplex 10% SDS‐0.1% HCl‐PBS solutions was added to dissolve the formazan deposited on the bottom of the plates, and the plates were further incubated at 37 °C overnight. The absorbance at 570 nm was measured with the reference wavelength at 650 nm using a microplate reader (Bio‐Tek, CA, USA).

### Elisa Analysis

Supernatants from peritoneal macrophage culture assayed for IL‐6, IL‐1β, and TNF‐α (BOSTER, Wuhan, China) according to the manufacturer's instructions. In the screening assay, peritoneal macrophages were seeded in a 96‐well plate at 2 × 10^4^ per well. The PMs were pre‐treated with compounds at a concentration of 10 µm for 1 h and then stimulated with LPS for another 24 h. Cell supernatants were analyzed by the ELISA assay. For in vivo analysis, after appropriate treatment, BALF of mice and cells supernatants were collected for ELISA analysis. The absorbance of each sample was measured by a microplate reader (BioTek, CA, USA) at 450 nm.

### RT‐PCR Analysis

Raw264.7 cells were seeded in a 24‐well plate (8 × 10^5^ cells per well) for 24 h. The cells were pre‐treated with different concentrations of **8b** for 1 h and then stimulated with LPS (1 µg mL^−1^) for 24 h. Total RNA was extracted using Trizol agent (Invitrogen, Carlsbad, CA). Reverse transcription was carried out according to PrimeScript 1st Strand cDNA Synthesis Kit (Takara, Japan) instructions. After cDNA was mixed with SYBR Green (Bio‐Rad, Berkeley, CA), quantitative PCR reactions were performed on the CFX96 Touch Real‐Time PCR Detection System (Bio‐Rad). Triplicate samples per condition were analyzed. Then data were analyzed by the 2DDCt method and compared with GAPDH for normalization of the samples. The primers used were as follows (**Table** **1**).

**Table 1:** Sequences of the primers used for RT‐PCR.

Gene	Forward	Reverse
Keap1	CGGGGACGCAGTGATGTATG	TGTGTAGCTGAAGGTTCGGTTA
TNF‐α	AGTCCGGGCAGGTCTACTTT	TTGGACCCTGAGCCATAATC
IL‐6	GAGCCCACCAAGAACGATAG	TTTCCACGATTTCCCAGAGA
IL‐1β	TGAGCACCTTCTTTTCCTTCATC	TGTCTAATGGGAACGTCACACAC
GAPDH	CACTCACGGCAAATTCAACGGCAC	GACTCCACGACATACTCAGCAC

### Detection of Nitric Oxide

After being seeded in 24‐well plates, Raw264.7 cells were pre‐treated with **8b** (2.5, 5, or 10 µm) for 1 h. Then, cells were exposed to 1 µg mL^−1^ LPS for 24 h. The supernatant was collected, and nitric oxide levels were measured by using the NO assay kit (S0021, Beyotime).

### Detection of ROS

Intracellular ROS levels in Raw264.7 cells were tested using a DCFH‐DA fluorescent probe. Cells were seeded into a 12‐well plate (4 × 10^5^ cells per well) and were pre‐treated with **8b** (2.5, 5, or 10 µm) for 1 h. Then, cells were exposed to 1 µg mL^−1^ LPS for 24 h. Afterward, the treated cells were collected, washed with PBS three times, and then stained with 500 µL PBS containing 50 µm DCFH‐DA for 20 min at 37 °C and then rinsed with serum‐free medium three times to remove excess DCFH‐DA and resuspended in PBS. Then, ROS in treated cells were analyzed using a flow cytometer at the excitation and emission wavelengths of 485 and 535 nm.

### Immunofluorescence

Raw264.7 cells were treated as mentioned above. Then, treated cells were fixed with 4% paraformaldehyde for 15 min. Next, cells were saturated with 0.5% Triton X‐100 for 10 min. Afterward, cells were blocked with 1% BSA for 60 min. The primary antibody against Nrf2 and secondary antibody were used for incubation in turn. After being washed in PBS, the slides were subjected to DAPI staining. Fluorescence detection was performed to observe the fluorescence of Nrf2.

### Western Blotting Analysis

Raw264.7 cells were seeded in a 6‐well plate in 1 × 10^6^ per well and pre‐treated with **8b** for 1 h, then exposed to 1 µg mL^−1^ LPS for 24 h. The cell supernatants were collected and centrifuged for 20 min, the liquid on the upper layer was collected, and cells were washed by PBS 3 times and lysed with lysis buffer for 30 min. The samples were separated on 10% SDS‐PAGE by electrophoresis and then transferred to PVDF membranes. The PVDF membranes were blocked with 5% BSA IN TNST for 1 h and incubated with the primary antibodies overnight at 4 °C. On day 2, the membranes were incubated with secondary antibodies for 1 h at room temperature. Finally, the signal was detected by the ChemiDocTM Touch imaging system (Bio‐Rad, Hercules, CA, USA). The antibodies against Keap1, HO‐1, COX‐2, iNOS, p‐p65, p65, p‐p38, p‐ERK, ERK, p‐JNK, JNK, Nrf2, p38, β‐actin, H3, GAPDH, and Ubiquitin were employed in this assay.

### Nucleocytoplasmic Separation

Stimulated with LPS or not for another 24 h. To separate the cytoplasmic and nuclear proteins, cell pellets were processed using the nuclear and cytoplasmic extraction kit (Beyotime, China) according to the manufacturer's instructions.

### Animal Treatment

C57BL/6 mice (aged 6–8 weeks, male) were purchased from Shanghai Family Planning Research Institute. Experiments were preapproved by the Institutional Animal Care and Use Committee of Zhejiang Chinese Medical University. Thirty C57BL/6 mice were randomly divided into five groups (*n* = 6 per group), including the control group, LPS‐treatment group, LPS + **8b** (10 mg kg^−1^) group, LPS + **8b** (20 mg kg^−1^) group, and LPS + Dex (5 mg kg^−1^) group. Dexamethasone (Dex) was used as a positive control. Specifically, mice in the control group and the LPS group were intraperitoneally injected with saline water, while mice in the administration groups were injected with **8b** or Dex once a day for 3 days. On the 3rd day, **8b** (10 or 20 mg kg^−1^) and Dex (5 mg kg^−1^) were intraperitoneally injected 1 h before LPS treatment. The mice were anesthetized by pentobarbital sodium, and a 3–5 mm longitudinal incision was made in the neck to expose the trachea. A scalp needle was inserted into the trachea, and the LPS‐containing air was quickly pushed in. The skin was sutured with a needle and thread. After the treatment of LPS for 24 h, mice were euthanized. Bronchoalveolar lavage (BALF) and lung tissues were collected for subsequent staining and analysis.

### Pharmacokinetic Analysis in SD Rats

Three male SD rats were in a group for PO (10 mg kg^−1^), IP (10 mg kg^−1^), and IV (5 mg kg^−1^) administration. The time points for blood sample collection were 0.08, 0.17, 0.33, 0.67, 1, 2, 4, 6, 9, and 24 h after administration. The plasma samples were extracted with acetonitrile and analyzed by high‐pressure liquid chromatography/tandem mass spectrometry (LC/MS/MS) with an Agilent Eclipse XDB‐C18 (2.1 mm × 100 mm, 3.5 µm) with an isocratic mobile phase of acetonitrile/water (10:90, v/v) containing 0.1% formic acid at 0.3 mL min^−1^ flow rate. Compound detection was performed with a mass spectrometer in multiple reaction monitoring (MRM) positive ionization mode. The PK parameters were calculated with DAS 3.0.

### Lung Distribution Analysis

Compound **8b** dissolved in distilled water and was intraperitoneally administrated to male SD rats (10 mg kg^−1^, *n* = 3). Lung tissue was collected at 0.25, 2, and 12 h after administration. Three times the weight of the volume of PBS (1×) were added and then homogenized. The homogenates were precipitated by five times of acetonitrile with internal standard. They were centrifuged for 5 min, and then 20 µL of the supernatant was mixed with 20 µL of water for analysis. Samples were analyzed by high‐pressure liquid chromatography/tandem mass spectrometry (LC/MS/MS) with an Agilent Eclipse XDB‐C18 (2.1 mm × 100 mm, 3.5 µm) with an isocratic mobiharmale phase of acetonitrile/water (10:90, v/v) containing 0.1% formic acid at 0.3 mL min^−1^ flow rate. Compound detection was performed with a mass spectrometer in multiple reaction monitoring (MRM) positive ionization mode.

### Bronchoalveolar Lavage Fluid (BALF) Collection

The thoracic cavity and neck were opened and the trachea was exposed. The lungs were lavaged three times with 1.0 mL of PBS. Then BALF was centrifuged for 10 min at 300 × g. Cell pellets and supernatant of BALF were collected separately. The cell pellet was resuspended in PBS, and then, a hemocytometer was used to count total white blood cells. The BCA Protein Assay kit (Beyotime, China) was used to detect the concentration of total protein. The nitric oxide in BALF was detected by using a NO Assay Kit (S0021, Beyotime, China).

### Lung Wet/Dry (W/D) Weight Measurement

The W/D ratio was used to assess the severity of pulmonary edema. After the mice were sacrificed, the lung tissues were removed. The lung tissues were weighed immediately and recorded as wet weight (W). Next, the wet lung tissues were placed in an oven at 70 °C for 48 h. And the lung tissues were weighed again to obtain the dry weight (D). Then the W/D ratio was calculated.

### H&E Staining

Fixing the lung tissues in 10% neutral buffered formalin for 24 h. After being dehydrated and embedded in paraffin, the lung tissues were sectioned at 3 µm thickness on a rotary microtome, and then, stained with hematoxylin and eosin (H&E) staining kit (Beyotime, China).

### Measurement of MPO, MDA, and SOD Levels

In order to analyze the MPO, MDA, and SOD content, the lung tissues were homogenized and dissolved in an extraction buffer. According to the manufacturer's instructions, MPO, MDA, and SOD content were assessed using assay kits. MPO and MDA content were used to evaluate the accumulation of neutrophils and the level of lipid peroxidation in the lung tissues. SOD content was used to evaluate the antioxidative enzyme activities in the lung tissues.

### Molecular Docking

The conformation of Keap1 (4IQK) was selected for molecular docking. Keap1 complex was prepared using the Protein Preparation Wizared Din Schrödinger 2018. The grid generation was based on the original ligand. **8b** was prepared using LigPrep and docked into the prepared structure by using the *Glide* module. The binding mode was analyzed with PyMOL. The **8b**‐Keap1 conformation with the highest docking score was submitted for 100 ns MD simulations.

### MM/GBSA Free Energy Decomposition

The interactions between each residue in Keap1 and **8b** were analyzed using the previously reported procedures. Especially, the last 50 ns MD simulation trajectory with 100 snapshots was submitted to the Molecular Mechanics/Generalized Born Surface Area (MM/GBSA) binding free energy calculation.^[^ ^20^ ^]^

### MD Simulations

The structure of the Keap1 bond with **8b** predicted by molecular docking was used as the initial conformations for the MD simulations. The MD simulations were performed with Amber18. Briefly, the AM1‐BCC atomic partial charges for the ligand were assigned with an antechamber. The FF14SB and GAFF2 force fields were solvated in a TIP3P water cubic box (10 Å), and Na+ ions were added to neutralize the net charge of the system. Four‐step energy minimizations were used to remove unfavorable contacts for the prepared systems. The systems were heated to 300 K over a period of 30 ps, and followed by 110 ps of equilibration in the NPT ensemble (*T* = 300 K and P = 1 bar). Finally, the systems were submitted to 100 ns MD simulations in the NPT ensemble (*T* = 300 K and P = 1 bar) with the PMEMD program. The snapshots were saved at 10 ps intervals. The RMSD value of the heavy atoms was determined using the cpptraj module included in AmberTools18.

### Statistical Analysis

All data represented at least three independent experiments. All experimental data were expressed as mean ± standard deviation (SD). Unpaired two‐tailed Student *t*‐test or one‐way analysis of variance (ANOVA) followed by Bonferroni's multiple comparisons test was employed to analyze the differences between sets of data. *p*‐Value < 0.05 was considered significant. (*p < 0.05, **p < 0.01, ***p < 0.001, ^****^ *p* < 0.0001, ns: no significant). Some results were normalized to the control to avoid sources of variation. Statistical analyses were performed using GraphPad Pro Prism 8.0 (GraphPad, San Diegao, CA).

## Conflict of Interest

The authors declare no conflict of interest.

## Author Contributions

C.F. and Z.Z. contributed equally to this work. S.C. conceived and directed the project. Experiments and data analysis were conducted by C.F., Z.Z., Z.L., Y.Y, J.L., L.L., S.C, and X.H., and the mechanism was proposed by S.C., H.Z., C.F., and H.Z., and S.C. wrote the manuscript.

## Supporting information

Supporting Information

Supporting Information

## Data Availability

The data that support the findings of this study are available in the supplementary material of this article.
