# Supplementary material for: Chemical Evolution and Biological Evaluation of Natural Products for Efficient Therapy of Acute Lung Injury
Source: Adv Sci (Weinh). 2023 Dec 21;11(7):2305432. doi: 10.1002/advs.202305432 (PMC10870070; doi:10.1002/advs.202305432)
Supplement: Supplementary file 1 — Supporting Information [file ADVS-11-2305432-s001.pdf]

ADVANCED SCIENCE
Open Access

Supporting Information

for *Adv. Sci.*, DOI 10.1002/advs.202305432

Chemical Evolution and Biological Evaluation of Natural Products for Efficient Therapy of Acute Lung Injury

Chengcheng Fan, Zeyi Zhang, Zhencheng Lai, Yanzi Yang, Jiaming Li, Lei Liu, Siyu Chen, Xueping Hu, Huajun Zhao and Sunliang Cui**

Supporting Information

Chemical Evolution and Biological Evaluation of Natural Product for Efficient Therapy of Acute Lung Injury

Chengcheng Fan, Zeyi Zhang, Zhencheng Lai, Yanzi Yang, Jiaming Li, Lei Liu, Siyu Chen, Xueping Hu, Huajun Zhao,* and Sunliang Cui*

1. General Information

All chemical reagents and solvents employed, unless otherwise noted, were purchased commercially and were used as provided without further purification. All anhydrous reactions were performed under an argon atmosphere using dry solvents. Flash column chromatography was carried out on over silica gel (200–300 mesh). ^{1}H NMR and ^{13}C NMR spectra were recorded on a Bruker AV-500 spectrometer or a WNMR-I-400 spectrometer at room temperature. $CDCl_3$, CD_3OD or DMSO-d_6 was used as solvent, chemical shifts were referenced relative to residual solvent. All NMR spectra were analyzed using MestReNova 10.0 program software. Multiplicity patterns are designated as follows: bs, broad singlet; s, singlet; d, doublet; t, triplet; q, quartet; m, multiplet. Coupling constants (*J*) are reported in Hertz (Hz). HRMS were performed on Agilent Technologies 6546-LC/Q-TOF LC/MS apparatus (ESI-TOF). Melting points were measured with X-4 micro melting point apparatus. The analytical data for the final compounds are provided in the Supporting Information. All tested compounds were determined to be >95% purity by HPLC. Solvent: methanol; flow rate = 1.0 mL/min; wavelength, 254 nm; SHIMADZU Shim-pack GIST, Material: 5 μM C18, Dimensions: 4.6 × 150 mm, P/N: 227-30017-07.

LPS, MTT and DMSO were purchased from Sigma (MO, USA). Antibodies against Keap1 (#8047S), HO-1 (#43966S), COX-2 (#12282S), iNOS (#13120S), p-p65 (#3033), p65 (#8242), p-p38 (#4511S), p38 (#8690S), p-ERK (#9101), ERK (#9102), p-JNK (#4668), JNK (#9258), Ubiquitin (#3936) and GAPDH (#2118) were purchased from Cell Signaling Technology (Massachusetts, USA). Antibody against Nrf2 (abs130481) was purchased from Absin Bioscience Inc (Shang, China), antibody against GCLM (ab126704) was purchased from Abcam (England), antibody against Histone H3 (#06–599) was purchased from Merck Millipore (Merck, Germany), and antibody against β-actin (#380624) was purchased from Chengdu Zen Biotechnology (Chengdu, China).

2. Chemical Synthesis

Procedure for the synthesis of 3

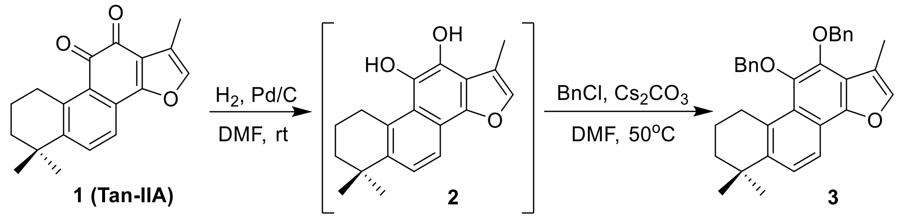

A two-necked flask equipped with a magnetic stirrer bar was charged with Tanshinone IIA (2.94 g, 0.01 mol, 1 equiv.) and 10% Pd/C (0.3g, 5% Palladium on activated carbon, wetted with ca. 55% water), purged with hydrogen several times, and anhydrous *N*, *N*-dimethylformamide (DMF, 15 mL) was added. The resulting reaction mixture was stirred at room temperature until the red color disappeared. Afterward, the mixture was transferred by a syringe into another two-necked flask charged with Cs_2CO_3 (13.04 g, 0.04 mol, 4 equiv.) under an argon atmosphere (take note: avoiding contact with air during transfer operations), then, benzyl chloride (5.06 g, 0.04 mol, 4 equiv.) was added in one portion. The reaction solution was heated at 50 °C for 5 h. Afterwards, the reaction mixture was filtered through diatomite and washed with EtOAc (100 mL). The filtrate was washed with brine to remove DMF and dried over anhydrous Na_2SO_4. Evaporation of the solvents afforded the crude product, which was further purified by silica gel column chromatography, eluting with Petroleum Ether/EtOAc to give compound **3**.

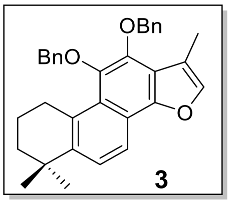

10,11-bis(benzyloxy)-1,6,6-trimethyl-6,7,8,9-tetrahydrophenanthro[1,2-*b*]furan

White solid (2.71 g, 57% yield). Rf = 0.1 (Petroleum ether / EtOAc = 100:1) ; m.p. 87-90 °C.

^{1}H NMR (400 MHz, $CDCl_3$) δ 8.11 (d, *J* = 8.8 Hz, 1H), 7.57 – 7.52 (m, 3H), 7.45 – 7.36 (m, 9H), 5.30 (s, 2H), 5.10 (s, 2H), 3.59 (t, *J* = 6.4 Hz, 2H), 2.34 (s, 3H), 1.85 (m, 2H), 1.77 (m, 2H), 1.43 (s, 6H).

^{13}C NMR (101 MHz, $CDCl_3$) δ 149.16, 145.39, 144.64, 143.28, 140.85, 137.89, 137.54, 132.72, 128.50 (×2), 128.43 (×4), 128.01, 127.88, 127.69(×2), 126.30, 125.31, 119.02, 118.34, 118.03, 116.36, 76.36, 76.28, 38.91, 35.03, 32.27 (×2), 30.87, 20.36, 9.73.

General procedure for the synthesis of 5

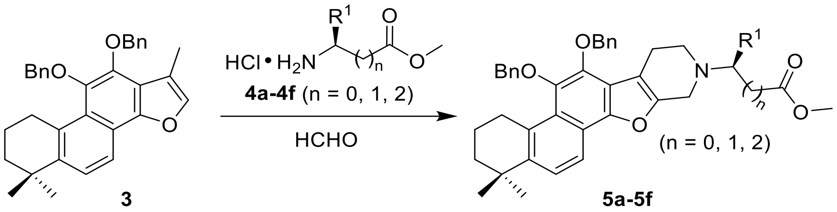

A sealed tube with a magnetic stirrer bar was charged with compound **3** (0.48 g, 1 mmol), paraformaldehyde (0.24 g, 8 mmol, 8 equiv), amino acid ester hydrochloride **4** (4 mmol, 4 equiv), and anhydrous AcOH 15 mL was added as solvent. The reaction mixture was stirred at 90 ^{o}C and monitored by TLC. After completion, the reaction was quenched by saturated aqueous $NaHCO_3$. The aqueous layer was extracted with EtOAc (50 mL × 3), and the combined organic layer was washed with brine, dried over anhydrous Na_2SO_4, concentrated in vacuo. The residue was purified by silica gel column chromatography to give the intermediates **5**.

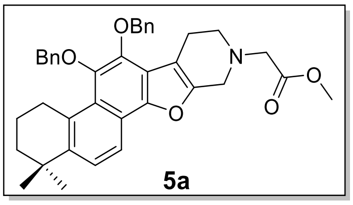

Methyl 2-(7,8-bis(benzyloxy)-3,3-dimethyl-3,5,6,9,10,12-hexahydrophenanthro[2',1':4,5]furo[2,3-*c*] pyridin-11(4*H*)-yl)acetate

White solid (0.36 g, 61.2% yield). Rf = 0.25 (Petroleum ether / EtOAc = 4:1); m.p. 123-127 ^{o}C.

^{1}H NMR (400 MHz, $CDCl_3$) δ 8.06 (d, *J* = 8.8 Hz, 1H), 7.53 (m, 3H), 7.39 (m, 8H), 5.25 (s, 2H), 5.10 (s, 2H), 3.99 (bs, 2H), 3.82 (bs, 3H), 3.55 (bs, 4H), 3.00 (t, *J* = 5.8 Hz, 2H), 2.90 (bs, 2H), 1.84 (m, 2H), 1.76 (s, 2H), 1.42 (s, 6H).

^{13}C NMR (101 MHz, $CDCl_3$) δ 170.97, 149.62, 148.01, 144.79, 144.62, 142.91, 137.90, 137.59, 132.77, 128.46 (×5), 128.03, 127.89 (×2), 127.69, 125.82, 125.29, 118.64, 118.27, 117.78, 111.35, 76.41, 76.33, 57.99, 51.90, 50.35, 50.05, 38.92, 35.00, 32.24 (×2), 30.85, 22.08, 20.35.

General procedure for the synthesis of 6a–6f

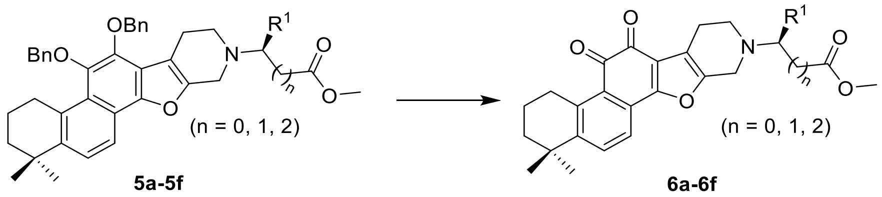

A two-necked flask equipped with a magnetic stirrer bar was charged with **5** (1 mmol) and 10% Pd/C (5% Palladium on activated carbon, wetted with ca. 55% water), anhydrous THF 15 mL was added, and purged with hydrogen several times. The resulting mixture was stirred under

a hydrogen atmosphere overnight. The debenzylation and following oxidation upon air exposure of **5** would recover the quinone moiety to deliver hybrid **6**.

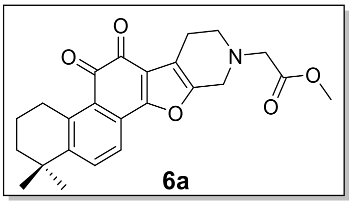

Methyl 2-(3,3-dimethyl-7,8-dioxo-3,5,6,7,8,9,10,12-octahydrophenanthro[2',1':4,5] furo[2,3-*c*]pyridin-11(4*H*)-yl)acetate

Red solid (0.29 g, 70.1 % yield). Rf = 0.3 (DCM / MeOH = 50:1); m.p. 121-125 °C.

^{1}H NMR (500 MHz, CDCl$_3$) δ 7.61 (d, *J* = 8.1 Hz, 1H), 7.50 (d, *J* = 8.2 Hz, 1H), 3.82 (t, *J* = 2.0 Hz, 2H), 3.77 (s, 3H), 3.51 (s, 2H), 3.18 (t, *J* = 6.4 Hz, 2H), 2.94 (t, *J* = 5.6 Hz, 2H), 2.84 (dq, *J* = 5.9, 3.7, 2.8 Hz, 2H), 1.82 – 1.76 (m, 2H), 1.68 – 1.63 (m, 2H), 1.31 (s, 6H).

^{13}C NMR (126 MHz, CDCl$_3$) δ 183.49, 175.27, 170.69, 160.72, 150.65, 149.97, 144.67, 133.46, 127.55, 126.24, 120.03, 119.40, 115.66, 57.73, 51.89, 49.70, 49.05, 37.84, 34.65, 31.84(×2), 29.95, 21.41, 19.12.

HRMS (ESI) m/z calcd for $C_{24}H_{26}NO_5$ $(M + H)^+$: 408.1811; Found: 408.1807.

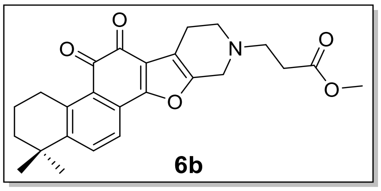

Methyl 3-(3,3-dimethyl-7,8-dioxo-3,5,6,7,8,9,10,12-octahydrophenanthro[2',1':4,5] furo[2,3-*c*]pyridin-11 (4*H*)-yl)propanoate

Red solid (0.30 g, 71.3 % yield). Rf = 0.3 (DCM / MeOH = 50:1); m.p. 123-126 °C.

^{1}H NMR (500 MHz, CDCl$_3$) δ 7.61 (d, *J* = 8.1 Hz, 1H), 7.50 (d, *J* = 8.1 Hz, 1H), 3.71 (s, 3H), 3.64 (s, 2H), 3.17 (t, *J* = 6.4 Hz, 2H), 2.95 (t, *J* = 7.2 Hz, 2H), 2.82 – 2.78 (m, 4H), 2.61 (t, *J* = 7.2 Hz, 2H), 1.82 – 1.77 (m, 2H), 1.67 – 1.64 (m, 2H), 1.31 (s, 6H).

^{13}C NMR (126 MHz, CDCl$_3$) δ 183.52, 175.27, 172.61, 160.68, 150.96, 149.92, 144.65, 133.46, 127.59, 126.22, 120.01, 119.42, 115.87, 52.39, 51.81, 49.80, 49.53, 37.85, 34.65, 32.66, 31.84(×2), 29.94, 21.42, 19.12.

HRMS (ESI) m/z calcd for $C_{25}H_{28}NO_5$ $(M + H)^+$: 422.1967; Found: 422.1969.

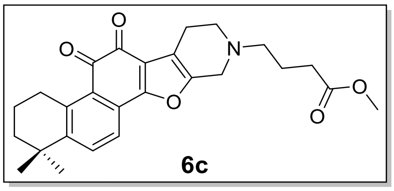

6c

Methyl 4-(3,3-dimethyl-7,8-dioxo-3,5,6,7,8,9,10,12-octahydrophenanthro[2',1':4,5] furo[2,3-*c*]pyridin-11 (4*H*)-yl)butanoate

Red solid (0.31 g, 70.3 % yield). Rf = 0.25 (DCM / MeOH = 50:1); m.p. 123-127 ^{o}C.

^{1}H NMR (500 MHz, CDCl$_3$) δ 7.60 (d, *J* = 8.1 Hz, 1H), 7.49 (d, *J* = 8.1 Hz, 1H), 3.66 (s, 3H), 3.59 (t, *J* = 1.9 Hz, 2H), 3.17 (t, *J* = 6.4 Hz, 2H), 2.78 (m, 4H), 2.63 (t, *J* = 7.2 Hz, 2H), 2.41 (t, *J* = 7.3 Hz, 2H), 1.91 (p, *J* = 7.2 Hz, 2H), 1.82 – 1.77 (m, 2H), 1.67 – 1.64 (m, 2H), 1.31 (s, 6H).

^{13}C NMR (126 MHz, CDCl$_3$) δ 183.55, 175.28, 173.92, 160.59, 151.43, 149.84, 144.60, 133.44, 127.65, 126.20, 119.99, 119.46, 115.91, 56.51, 51.59, 50.06, 49.61, 37.85, 34.64, 31.84 (×2), 31.76, 29.93, 22.61, 21.56, 19.13.

HRMS (ESI) m/z calcd for $C_{26}H_{30}NO_5$ $(M + H)^+$: 436.2124; Found: 436.2123.

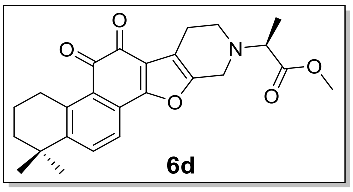

6d

Methyl (*S*)-2-(3,3-dimethyl-7,8-dioxo-3,5,6,7,8,9,10,12-octahydrophenanthro[2',1':4,5] furo[2,3-*c*] pyridin-11(4*H*)-yl)propanoate

Red solid (0.32 g, 76.5 % yield). Rf = 0.25 (DCM / MeOH = 100:1); m.p. 121-125 ^{o}C.

^{1}H NMR (500 MHz, CDCl$_3$) δ 7.60 (d, *J* = 8.2 Hz, 1H), 7.49 (d, *J* = 8.1 Hz, 1H), 3.84 (t, *J* = 2.0 Hz, 2H), 3.75 (s, 3H), 3.61 (q, *J* = 7.1 Hz, 1H), 3.17 (t, *J* = 6.4 Hz, 2H), 2.99 – 2.94 (m, 1H), 2.87 – 2.82 (m, 1H),2.81 – 2.77 (m, 2H), 1.82 – 1.77 (m, 2H), 1.67 – 1.64 (m, 2H),1.-43 (d, *J* = 7.1 Hz, 3H), 1.31 (s, 6H).

^{13}C NMR (126 MHz, CDCl$_3$) δ 184.06, 175.25, 173.28, 160.64, 151.32, 149.88, 144.63, 133.-46,127.61, 126.22, 119.98, 119.48, 115.88, 61.66, 51.64, 46.92, 45.-78, 37.85, 34.64, 31.84(×2), 29.94, 22.19, 19.12, 15.21.

HRMS (ESI) m/z calcd. for $C_{25}H_{28}NO_5$ $(M + H)^+$: 422.1967; Found: 422.1985.

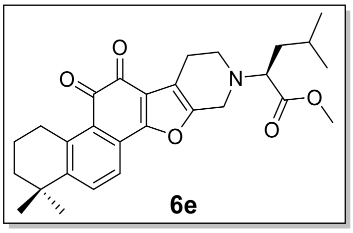

6e

Methyl (*S*)-2-(3,3-dimethyl-7,8-dioxo-3,5,6,7,8,9,10,12-octahydrophenanthro[2',1':4,5] furo[2,3-*c*] pyridin-11(4*H*)-yl)-4-methylpentanoate

Red solid (0.33 g, 70.9 % yield). Rf = 0.2 (DCM / MeOH = 100:1); m.p. 119-124 °C.

^{1}H NMR (500 MHz, $CDCl_3$) δ 7.60 (d, *J* = 8.1 Hz, 1H), 7.49 (d, *J* = 8.1 Hz, 1H), 3.83 (t, *J* = 2.1 Hz, 2H), 3.72 (s, 3H), 3.53 (t, *J* = 7.5 Hz, 1H), 3.17 (t, *J* = 6.4 Hz, 2H), 3.01 – 2.92 (m, 1H), 2.86 – 2.69 (m, 3H),1.84 – 1.75 (m, 2H), 1.71 – 1.63 (m, 5H), 1.31 (s, 6H), 0.94 (dd, *J* = 15.5, 6.5 Hz, 6H).

^{13}C NMR (126 MHz, $CDCl_3$) δ 183.61, 175.32, 172.97, 160.57, 151.63, 149.84, 144.62, 133.46, 127.69, 126.22,119.97, 119.55, 115.90, 64.75, 51.28, 46.88, 45.80, 38.45, 37.86, 34.64, 31.84 (×2), 29.93, 24.92, 22.69, 22.36, 19.13.

HRMS (ESI) m/z calcd. for $C_{28}H_{34}NO_5$ $(M + H)^+$: 464.2437; Found: 464.2435.

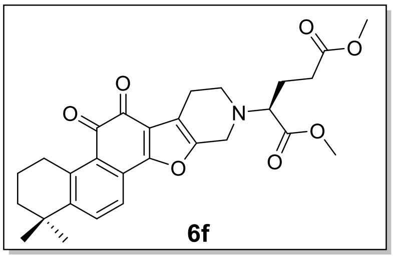

6f

Dimethyl (*S*)-2-(3,3-dimethyl-7,8-dioxo-3,5,6,7,8,9,10,12-octahydrophenanthro[2',1':4,5] furo[2,3-*c*] pyridin-11(4*H*)-yl)pentanedioate

Red solid (0.37 g, 74.9 % yield). Rf = 0.2 (DCM / MeOH = 30:1); m.p. 119-125 °C.

^{1}H NMR (500 MHz, $CDCl_3$) δ 7.61 (d, *J* = 8.1 Hz, 1H), 7.49 (d, *J* = 8.1 Hz, 1H), 3.90 – 3.72 (m, 2H), 3.74 (s, 3H), 3.63 (s, 3H), 3.51 (dd, *J* = 8.6,6.8 Hz, 1H), 3.17 (t, *J* = 6.5 Hz, 2H), 3.03 – 2.97 (m, 1H), 2.80 – 2.72 (m, 3H), 2.45 (td, *J* = 7.2, 2.9 Hz, 2H), 2.16 – 2.09 (m, 2H), 1.82 – 1.76 (m, 2H), 1.67 – 1.62 (m, 2H), 1.31 (s, 6H).

^{13}C NMR (126 MHz, $CDCl_3$) δ 183.54, 175.28, 173.47, 172.04, 160.59, 151.40, 149.87, 144.63, 133.48, 127.61, 126.19, 119.99, 119.47, 115.83, 76.78, 65.64, 51.65, 51.48, 46.89, 45.80, 37.84, 34.65, 31.84 (×2), 30.59, 29.94, 24.30, 22.27, 19.12.

HRMS (ESI) m/z calcd. for $C_{28}H_{32}NO_7$ $(M + H)^+$: 494.2179; Found: 494.2180.

General procedure for the synthesis of 7a–7f

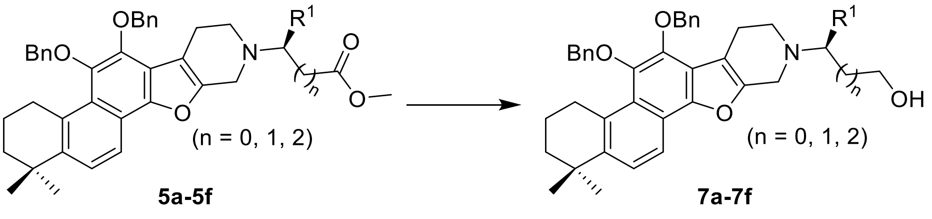

A two-necked flask equipped with a magnetic stirrer bar was charged with compound **5** (1 mmol) and anhydrous THF was added as the solvent. This reaction mixture was cooled to 0 °C,

and $LiAlH_4$ (10 mmol) was added portions, then the reaction mixture kept at 0 °C for 1 h under an argon atmosphere. Afterwards, 50 mL EtOAc was added to quench the reaction and the resulting mixture was diluted with 1M NaOH aqueous solution, extracted with EtOAc three times. The combined organic layers were dried with anhydrous Na_2SO_4. filtered and concentrated under vacuum to obtain the crude product, which was further purified by silica gel column chromatography using DCM/MeOH as the eluent to give compounds **7**.

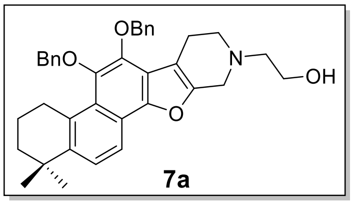

7a

2-(7,8-bis(benzyloxy)-3,3-dimethyl-3,5,6,9,10,12-hexahydrophenanthro [2',1':4,5]furo[2,3-*c*] pyridin-11(4*H*)-yl)ethan-1-ol

White solid (0.5 g, 89.6 % yield). Rf = 0.3 (DCM / MeOH = 50:1); m.p. 115-118 °C.

^{1}H NMR (500 MHz, $CDCl_3$) δ 8.01 (d, *J* = 8.7 Hz, 1H), 7.53 – 7.45 (m, 3H), 7.41 – 7.28 (m, 8H), 5.22 (s, 2H), 5.05 (s, 2H), 3.81 (bs, 2H), 3.73 (t, *J* = 5.4 Hz, 2H), 3.53 (t, *J* = 6.3 Hz, 2H), 2.87 (t, *J* = 5.6 Hz, 2H), 2.80 (m, 4H), 1.79 (m, 2H), 1.76 – 1.67 (m, 2H), 1.37 (s, 6H).

^{13}C NMR (126 MHz, $CDCl_3$) δ 149.95, 147.93, 144.78, 144.54, 142.88, 137.83, 137.55, 132.73, 128.41 (×4), 128.00 (×2), 127.87 (×2), 127.68 (×2), 125.78, 125.30, 118.60, 118.24, 117.71,111.60, 76.35, 76.30, 58.40, 58.23, 50.35, 50.25, 38.85, 34.97, 32.20 (×2), 30.81, 22.14, 20.30.

General procedure for the synthesis of 8a–8f

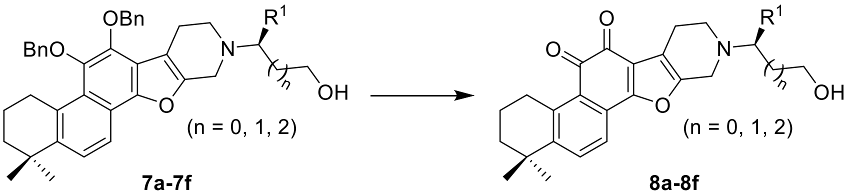

The synthesis method for **8a** – **8f** was same to preparation of compounds **6a** – **6f**. Generally, a two-necked flask equipped with a magnetic stirrer bar was charged with **7** (1 mmol) and Pd/C (10% of the weight of **7**), purged with hydrogen several times, and anhydrous THF 15 mL was added. The resulting mixture was stirred under a hydrogen atmosphere overnight and then, oxidized upon exposure to air to afford compounds **8a-8f**.

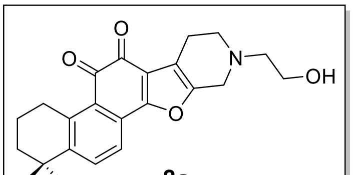

8a

11-(2-hydroxyethyl)-3,3-dimethyl-3,4,5,6,9,10,11,12-octahydrophenanthro [2',1':4,5]furo[2,3-*c*] pyridine-7,8-dione

Red solid (0.27 g, 72.3 % yield). Rf = 0.2 (DCM / MeOH = 25:1); m.p. 116-120 °C.

^{1}H NMR (500 MHz, CDCl$_3$) δ 7.59 (d, *J* = 8.1 Hz, 1H), 7.46 (d, *J* = 8.2 Hz, 1H), 3.73 (t, *J* = 5.3 Hz, 2H), 3.68 (s, 2H), 3.17 (t, *J* = 6.4 Hz, 2H), 2.86 (t, *J* = 5.1 Hz, 2H), 2.80 (m, 4H), 1.84 – 1.75 (m, 2H), 1.69 – 1.63 (m, 2H), 1.31 (s, 6H).

^{13}C NMR (126 MHz, CDCl$_3$) δ 183.41, 175.19, 160.65, 1-50.99, 149.98, 144.67, 133.47, 127.53, 126.19, 119.98, 119.39, 115.96, 58.48, 58.26, 49.83, 49.49, 37.84, 34.66, 31.85 (×2), 29.95, 2- 1.39, 19.12.

HRMS (ESI) m/z calcd. for $C_{23}H_{26}NO_4$ $(M + H)^+$: 380.1862; Found: 380.1851.

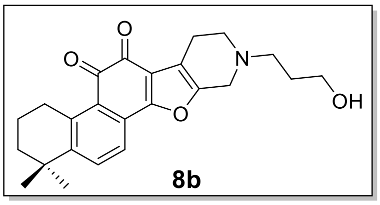

11-(3-hydroxypropyl)-3,3-dimethyl-3,4,5,6,9,10,11,12-octahydrophenanthro [2',1':4,5]furo[2,3-*c*] pyridine-7,8-dione

Red solid (0.30 g, 75.1 % yield). Rf = 0.2 (DCM / MeOH = 25:1); m.p. 118-123 °C.

^{1}H NMR (500 MHz, CDCl$_3$) δ 7.61 (d, *J* = 8.1 Hz, 1H), 7.49 (d, *J* = 8.1 Hz, 1H), 3.86 – 3.83 (t, *J* = 5.5 Hz 2H), 3.69 (t, *J* = 1.9 Hz, 2H), 3.17 (t, *J* = 6.4 Hz, 2H), 2.88 – 2.84 (m, 4H), 2.83 – 2.80 (m, 2H), 1.87 – 1.75 (m, 4H), 1.68 – 1.63 (m, 2H), 1.31 (s, 6H).

^{13}C NMR (126 MHz, CDCl$_3$) δ 183.42, 175.22, 160.75, 150.62, 149.99, 144.68, 133.48, 127.52, 126.22, 120.00, 119.34, 115.91, 64.15, 57.42, 50.20, 49.88, 37.84, 34.66, 31.84 (×2), 29.94, 28.02, 21.30, 19.12.

HRMS (ESI) m/z calcd. for $C_{24}H_{28}NO_4$ $(M + H)^+$: 394.2018; Found: 394.2030.

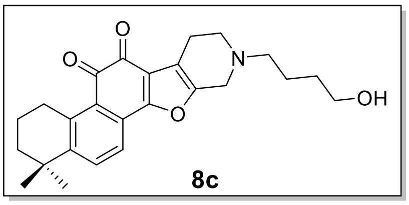

11-(4-hydroxybutyl)-3,3-dimethyl-3,4,5,6,9,10,11,12-octahydrophenanthro [2',1':4,5]furo[2,3-*c*] pyridine-7,8-dione

Red solid (0.30 g, 74.7 % yield). Rf = 0.2 (DCM / MeOH = 25:1); m.p. 120-124 °C.

^{1}H NMR (500 MHz, CDCl$_3$) δ 7.60 (d, *J* = 8.1 Hz, 1H), 7.48 (d, *J* = 8.1 Hz, 1H), 3.66 (s, 2H), 3.62 (t, *J* = 5.1 Hz, 2H), 3.17 (t, *J* = 6.4 Hz, 2H), 2.84 (s, 4H), 2.66 (t, *J* = 5.7 Hz, 2H), 1.78 – 1.82 (m, 2H), 1.78 – 1.71 (m, 4H), 1.67 – 1.64 (m, 2H), 1.31 (s, 6H).

^{13}C NMR (126 MHz, CDCl$_3$) δ 183.41, 175.18, 160.78, 150.45, 149.98, 144.68, 133.46, 127.53, 126.22, 119.99, 119.38, 115.96, 77.28, 62.74, 57.34, 49.84, 49.57, 37.84, 34.66, 32.05, 31.84(×2), 29.94, 25.48, 21.06, 19.12. **HRMS (ESI)** m/z calcd. for $C_{25}H_{30}NO_4$ (M + H)$^+$: 408.2175; Found: 408.2172.

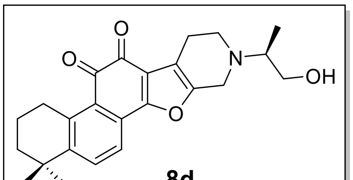

8d

(*S*)-11-(1-hydroxypropan-2-yl)-3,3-dimethyl-3,4,5,6,9,10,11,12-octahydrophenanthro [2',1':4,5]furo [2,3-*c*]pyridine-7,8-dione

Red solid (0.29 g, 72.6 % yield). Rf = 0.3 (DCM / MeOH = 30:1); m.p. 117-121 °C.

^{1}H NMR (500 MHz, CDCl$_3$) δ 7.60 (dd, *J* = 8.1, 1.3 Hz, 1H), 7.52 – 7.44 (m, 1H), 3.81 – 3.58 (m, 2H), 3.53 – 3.45 (m, 2H), 3.18 (t, *J* = 6.4 Hz, 2H), 3.10 – 3.05 (m, 1H), 2.95 (m, 1H), 2.86 – 2.72 (m, 2H), 2.66 (m, 1H), 1.78 (s, 2H), 1.69 – 1.63 (m, 2H), 1.31 (s, 6H), 1.04 (d, J = 6.7 Hz, 3H).

^{13}C NMR (126 MHz, CDCl$_3$) δ 183.45, 175.24, 160.64, 151.46, 149.93, 144.67, 133.47, 127.57, 126.20, 119.95, 119.45, 116.13, 62.78, 60.03, 45.11, 44.95, 37.84, 34.66, 31.85 (×2), 29.95, 22.30, 19.12, 9.85.

HRMS (ESI) m/z calcd. for $C_{24}H_{28}NO_4$ (M + H)$^+$: 394.2018; Found: 394.2031.

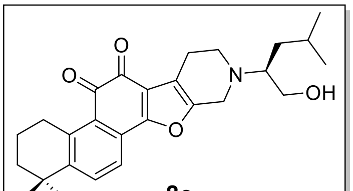

8e

(*S*)-11-(1-hydroxy-4-methylpentan-2-yl)-3,3-dimethyl-3,4,5,6,9,10,11,12-octahydro phenanthro[2',1':4,5]furo[2,3-*c*]pyridine-7,8-dione

Red solid (0.31 g, 72.3 % yield). Rf = 0.4 (DCM / MeOH = 30:1); m.p. 121-125 °C.

^{1}H NMR (500 MHz, CDCl$_3$) δ 7.60 (d, *J* = 8.2 Hz, 1H), 7.45 (d, *J* = 8.1 Hz, 1H), 3.85 – 3.61 (m, 2H), 3.59 (dd, *J* = 10.8, 4.9 Hz, 1H), 3.42 (t, *J* = 10.6 Hz, 1H), 3.17 (t, *J* = 6.4 Hz, 2H), 3.03 – 2.92 (m, 2H), 2.83 – 2.69 (m, 2H), 2.69 – 2.64 (m, 1H), 1.84 – 1.75 (m, 2H), 1.69 – 1.64 (m, 2H), 1.67 – 1.54 (m, 1H), 1.44 (m, 1H), 1.31 (s, 6H), 1.17 (m, 1H), 0.95 (t, J = 6.8 Hz, 6H).

^{13}C NMR (126 MHz, CDCl$_3$) δ 183.44, 175.21, 160.59, 151.50, 149.92, 144.66, 133.47, 127.58, 126.19, 119.92, 119.48, 117.33, 62.90, 61.10, 45.26, 45.13, 37.84, 34.71, 34.66, 31.85 (×2), 29.94, 25.50, 23.64, 22.51, 22.19, 19.13.

HRMS (ESI) m/z calcd. for $C_{27}H_{34}NO_4$ (M + H)$^+$:436.2488; Found: 436.2485.

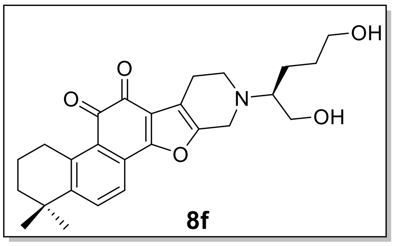

(*S*)-11-(1,5-dihydroxypentan-2-yl)-3,3-dimethyl-3,4,5,6,9,10,11,12-octahydrophenanthro [2',1':4,5]furo[2,3-*c*]pyridine-7,8-dione

Red solid (0.31 g, 71.7 % yield). Rf = 0.2 (DCM / MeOH = 20:1); m.p. 116-120 °C.

^{1}H NMR (500 MHz, CDCl$_3$) δ 7.59 (d, *J* = 8.1 Hz, 1H), 7.45 (d, *J* = 8.2 Hz, 1H), 3.86 – 3.72 (m, 2H), 3.71 – 3.65 (m, 3H), 3.55 (dd, *J* = 11.0, 9.3 Hz, 1H), 3.17 (t, *J* = 6.4 Hz, 2H), 3.02 – 2.87 (m, 2H), 2.83 – 2.73 (m, 3H), 1.83 – 1.77 (m, 2H), 1.75 – 1.69 (m,1H), 1.68 – 1.64 (m, 3H), 1.63 – 1.56 (m, 1H), 1.49 – 1.41 (m, 1H), 1.31 (s, 6H). **^{13}C NMR (126 MHz, CDCl$_3$)** δ 183.37, 175.16, 160.66, 151.26, 149.95, 144.68, 133.48, 127.52, 126.15, 119.94, 119.43, 116.10, 65.08, 62.72, 61.14, 45.52, 45.47, 37.83, 34.66, 31.85 (×2), 30.40, 29.96, 23.25, 22.38, 19.12. **HRMS (ESI)** m/z calcd. for $C_{26}H_{32}NO_5$ $(M + H)^+$: 438.2280; Found: 438.2288.

General procedure for the synthesis of 10a–10n

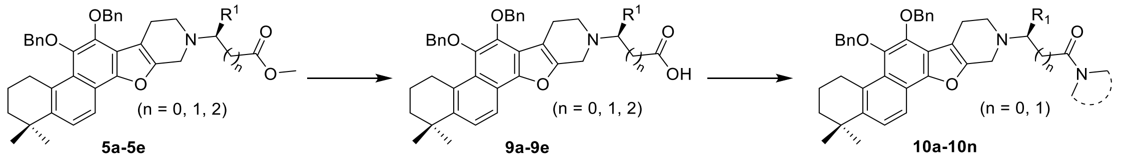

To a solution of compound **5** (1 mmol) in 20 mL THF and distilled water [V(THF)/V(H_2O) = 1:1] was added LiOH (0.24g, 10 mmol), and this reaction mixture was heated and stirred at room temperature for 12 h. Afterward, 1M HCl aqueous was added slowly to adjust pH until pH = 7. Then, the mixture was diluted with water and extracted with EtOAc three times. The combined organic layer was washed with brine and dried over anhydrous Na_2SO_4. Evaporation of the solvent to afford corresponding acid **9**, which was used in the next step directly without any purification. Compound **9** (1 mmol) was dissolved in 20 mL DCM, and mixed with methylamine hydrochloride (0.14 g, 2 mmol), HATU (0.57 g, 1.5 mmol) and DIPEA (0.52 g, 4 mmol). The mixture was stirred at room temperature overnight under an argon atmosphere. After reaction completed, the mixture was diluted with saturated NH_4Cl aqueous and extracted with EtOAc three times. The combined organic layer was washed with brine and dried over anhydrous Na_2SO_4. After filtration and evaporation, the crude product was purified through column chromatography over silica gel to obtain compound **10**.

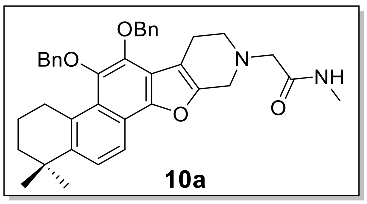

10a

White solid (0.41 g, 70.1 % yield of two steps). Rf = 0.4 (DCM / MeOH = 25:1); m.p. 125-129 °C.

^{1}H NMR (500 MHz, CDCl$_3$) δ 8.01 (d, *J* = 8.7 Hz, 1H), 7.52 – 7.47 (m, 3H), 7.38 – 7.32 (m, 8H), 7.23 (s, 1H), 5.23 (s, 2H), 5.05 (s, 2H), 3.82 (s, 2H), 3.53 (t, *J* = 6.3 Hz, 2H), 3.27 (s, 2H), 2.88 – 2.84 (m, 5H), 2.79 (bs, 2H), 1.80 (m, 2H), 1.70 (m, 2H), 1.38 (s, 6H).

^{13}C NMR (126 MHz, CDCl$_3$) δ 170.77, 149.42, 147.97, 144.85, 144.46, 143.04, 137.75, 137.54, 132.77, 128.42 (×4), 128.38 (×2), 128.02, 127.88 (×2), 127.72, 125.91, 125.39, 118.44, 118.22, 117.70, 111.40, 76.34, 76.32, 60.57, 51.16, 50.87, 38.84, 34.98, 32.20 (×2), 30.81, 25.84, 22.30, 20.29.

General procedure for the synthesis of 11a–11n

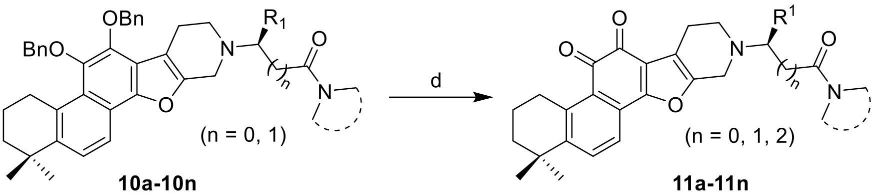

According to the procedure for preparing compounds **6**, compounds **11a** – **11n** was synthesized from **10a** – **10n** through *de*-benzylation and oxidation reactions.

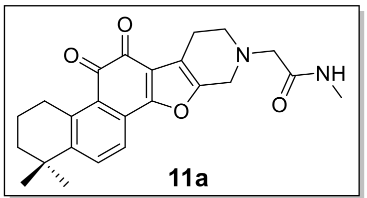

11a

2-(3,3-dimethyl-7,8-dioxo-3,5,6,7,8,9,10,12-octahydrophenanthro[2',1':4,5]furo[2,3-*c*]pyridin-11(4*H*)-yl)-*N*-methylacetamide

Red solid (69.8 % yield). Rf = 0.3 (DCM / MeOH = 50:1). m.p. 128-132 °C.

^{1}H NMR (500 MHz, CDCl$_3$) δ 7.56 (d, J = 8.1 Hz, 1H), 7.36 (d, J = 8.1 Hz, 1H), 3.65 (t, J = 1.9 Hz, 2H), 3.27 (s, 2H), 3.17 (t, J = 6.4 Hz, 2H), 2.90 (d, J = 5.0 Hz, 3H), 2.85 (m, 2H), 2.80 (td, J = 5.0, 2.4 Hz, 2H), 1.84 – 1.78 (m, 2H), 1.68 – 1.65 (m, 2H), 1.32 (s, 6H).

^{13}C NMR (126 MHz, CDCl$_3$) δ 183.11, 174.94, 170.38, 160.65, 150.47, 150.09, 144.71, 133.47, 127.30, 126.08, 119.91, 119.27, 115.76, 60.54, 50.61, 50.01, 37.81, 34.68, 31.86(×2), 29.99, 25.88, 21.66, 19.12.

HRMS (ESI) m/z calcd. for $C_{24}H_{27}N_2O_4$ $(M + H)^+$: 407.1971; Found: 407.1963.

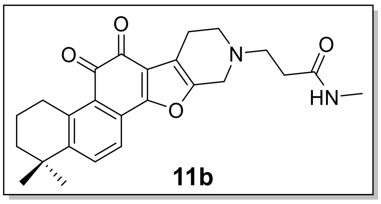

3-(3,3-dimethyl-7,8-dioxo-3,5,6,7,8,9,10,12-octahydrophenanthro[2',1':4,5]furo[2,3-*c*]pyridin-11(4*H*)-yl)-*N*-methylpropanamide

Red solid (70.1 % yield). Rf = 0.3 (DCM / MeOH = 50:1); m.p. 131-135 °C.

^{1}H NMR (500 MHz, CDCl$_3$) δ 7.60 (d, *J* = 8.2 Hz, 1H), 7.47 (d, *J* = 8.1 Hz, 1H), 3.66 (d, *J* = 1.9 Hz, 2H), 3.17 (t, *J* = 6.4 Hz, 2H), 2.90 (t, *J* = 6.3 Hz, 2H), 2.80 – 2.85 (m, 4H), 2.79 (d, *J* = 4.8 Hz, 3H), 2.49 (t, *J* = 6.2 Hz, 2H), 1.83 – 1.77 (m, 2H), 1.69 – 1.64 (m, 2H), 1.32 (s, 6H).

^{13}C NMR (126 MHz, CDCl$_3$) δ 183.29, 175.17, 172.51, 160.79, 150.57, 150.10, 144.72, 133.49, 127.43, 126.18, 119.99, 119.33, 115.89, 53.03, 49.64, 49.32, 37.81, 34.67, 33.05, 31.85 (×2), 29.96, 26.06, 21.42, 19.11.

HRMS (ESI) m/z calcd. for $C_{25}H_{29}N_2O_4$ $(M + H)^+$:421.2127; Found: 421.2141.

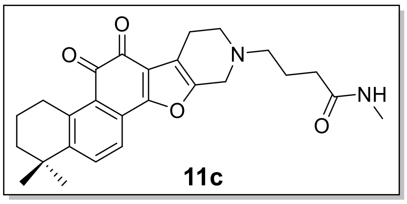

4-(3,3-dimethyl-7,8-dioxo-3,5,6,7,8,9,10,12-octahydrophenanthro[2',1':4,5]furo[2,3-*c*]pyridin-11(4*H*)-yl)-*N*-methylbutanamide

Red solid (70.9 % yield). Rf = 0.25 (DCM / MeOH = 50:1); m.p. 134-139 °C.

^{1}H NMR (500 MHz, CDCl$_3$) δ 7.59 (d, *J* = 8.2 Hz, 1H), 7.45 (d, *J* = 8.1 Hz, 1H), 3.61 (s, 2H), 3.16 (t, *J* = 6.4 Hz, 2H), 2.80 (7H), 2.67 (t, *J* = 6.8 Hz, 2H), 2.32 (t, *J* = 7.2 Hz, 2H), 1.93 (p, *J* = 6.9 Hz, 2H), 1.83 – 1.76 (m, 2H), 1.69 – 1.62 (m, 2H), 1.31 (s, 6H).

^{13}C NMR (126 MHz, CDCl$_3$) δ 183.42, 175.20, 173.44, 160.73, 151.07, 150.00, 144.68, 133.51, 127.53, 126.16, 120.02, 119.37, 115.87, 56.57, 50.08, 49.52, 37.84, 34.66, 34.14, 31.84 (×2), 29.95, 26.31, 22.87, 21.50, 19.12. **HRMS (ESI)** m/z calcd. for $C_{26}H_{31}N_2O_4$ $(M + H)^+$: 435.2284; Found: 435.2280

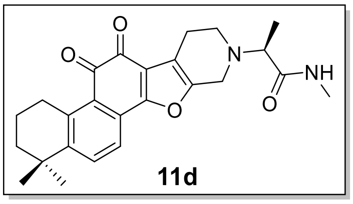

(*S*)-2-(3,3-dimethyl-7,8-dioxo-3,5,6,7,8,9,10,12-octahydrophenanthro[2',1':4,5]furo[2,3-*c*]pyridin-11(4*H*)-yl)-*N*-methylpropanamide

Red solid (71.4 % yield). Rf = 0.4 (DCM / MeOH = 50:1); m.p. 127-131 °C.

^{1}H NMR (500 MHz, $CDCl_3$) δ 7.57 (d, *J* = 8.1 Hz, 1H), 7.39 (d, *J* = 8.1 Hz, 1H), 3.72 – 3.60 (dd, 2H), 3.35 (q, *J* = 7.0 Hz, 1H), 3.17 (t, *J* = 6.4 Hz, 2H), 2.87 (d, *J* = 5.0 Hz, 3H), 2.82 – 2.72 (m, 4H), 1.84 – 1.77 (m, 2H), 1.68 – 1.65 (m, 2H), 1.37 (d, *J* = 7.0 Hz, 3H), 1.32 (d, *J* = 3.2 Hz, 6H).

^{13}C NMR (126 MHz, $CDCl_3$) δ 183.20, 175.02, 173.86, 160.63, 151.11, 150.05, 144.71, 133.49, 127.40, 126.11, 119.93, 119.35, 115.92, 63.46, 47.41, 46.54, 37.82, 34.68, 31.86 (×2), 29.99, 26.05, 22.10, 19.12, 12.17.

HRMS (ESI) m/z calcd. for $C_{25}H_{29}N_2O_4$ $(M + H)^+$: 421.2127; Found: 421.2133.

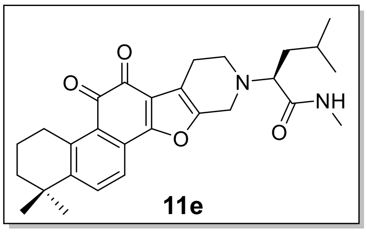

(*S*)-2-(3,3-dimethyl-7,8-dioxo-3,5,6,7,8,9,10,12-octahydrophenanthro[2',1':4,5]furo[2,3-*c*]pyridin-11(4*H*)-yl)-*N*,4-dimethylpentanamide

Red solid (72.5 % yield). Rf = 0.4 (DCM / MeOH = 50:1); m.p. 124-128 °C.

^{1}H NMR (500 MHz, $CDCl_3$) δ 7.56 (d, *J* = 8.2 Hz, 1H), 7.35 (d, *J* = 8.1 Hz, 1H), 7.06 (q, *J* = 5.0 Hz, 1H), 3.78 – 3.61 (m, 2H), 3.30 – 3.27 (m, 1H), 3.16 (t, *J* = 6.4 Hz, 2H), 2.86 (d, *J* = 4.9 Hz, 3H), 2.83 (t, *J* = 5.7 Hz, 2H), 2.79 – 2.74 (m, 2H), 1.84 – 1.76 (m, 4H), 1.69 – 1.63 (m, 2H), 1.51 (m, 1H), 1.32 (d, *J* = 4.4 Hz, 6H), 0.98 (dd, *J* = 9.6, 6.4 Hz, 6H).

^{13}C NMR (126 MHz, $CDCl_3$) δ 183.17, 174.95, 173.99, 160.51, 151.33, 149.96, 144.65, 133.46, 127.43, 126.06, 119.86, 119.41, 115.92, 66.09, 47.50, 46.28, 37.83, 36.81, 34.66, 31.86 (×2), 29.46, 26.02, 25.94, 23.25, 22.30, 22.23, 19.12.

HRMS (ESI) m/z calcd. for $C_{28}H_{35}N_2O_4$ $(M + H)^+$: 463.2597; Found: 463.2603.

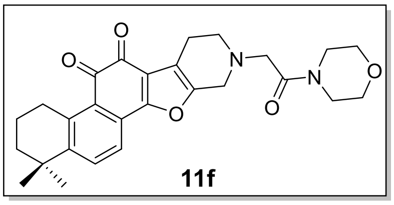

3,3-dimethyl-11-(2-morpholino-2-oxoethyl)-3,4,5,6,9,10,11,12-octahydrophenanthro [2',1':4,5]furo[2,3-*c*]pyridine-7,8-dione

Red solid (73.7 % yield). Rf = 0.3 (DCM / MeOH = 25:1); m.p. 141-145 °C.

^{1}H NMR (500 MHz, $CDCl_3$) δ 7.60 (d, *J* = 8.1 Hz, 1H), 7.47 (d, *J* = 8.1 Hz, 1H), 3.73 (t, *J* = 1.9 Hz, 2H), 3.71 – 3.60 (m, 8H), 3.46 (s, 2H), 3.17 (t, *J* = 6.4 Hz, 2H), 2.88 (t, *J* = 5.6 Hz, 2H), 2.81 (m, 2H), 1.84 – 1.75 (m, 2H), 1.69 – 1.63 (m, 2H), 1.31 (s, 6H).

^{13}C NMR (126 MHz, $CDCl_3$) δ 183.43, 175.26, 167.88, 160.70, 150.70, 150.04, 144.67, 133.47, 127.52, 126.20, 120.02, 119.39, 115.65, 66.97, 66.92, 59.47, 49.90, 49.32, 46.11, 42.58, 37.84, 34.66, 31.84 (×2), 29.94, 21.39, 19.12.

HRMS (ESI) m/z calcd. for $C_{27}H_{31}N_2O_5$ $(M + H)^+$: 463.2233; Found: 463.2239.

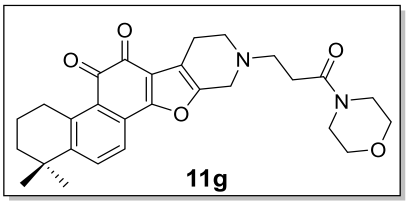

3,3-dimethyl-11-(3-morpholino-3-oxopropyl)-3,4,5,6,9,10,11,12-octahydrophenanthro [2',1':4,5] furo[2,3-*c*]pyridine-7,8-dione

Red solid (74.5 % yield). Rf = 0.2 (DCM / MeOH = 25:1); m.p. 144-149 °C.

^{1}H NMR (400 MHz, $CDCl_3$) δ 7.64 (d, *J* = 8.2 Hz, 1H), 7.52 (d, *J* = 8.0 Hz, 1H), 3.70 (m, 8H), 3.57 – 3.50 (m, 2H), 3.20 (t, *J* = 6.4 Hz, 2H), 3.01 (t, *J* = 7.5 Hz, 2H), 2.83 (s, 4H), 2.65 (t, *J* = 7.4 Hz, 2H), 1.82 (m, 2H), 1.72 – 1.65 (m, 2H), 1.34 (s, 6H).

^{13}C NMR (101 MHz, $CDCl_3$) δ 183.55, 175.32, 170.04, 160.73, 151.17, 150.00, 144.69, 133.50, 127.63, 126.28, 120.04, 119.46, 115.88, 66.93, 66.66, 52.92, 50.13, 50.04, 46.03, 42.03, 37.89, 34.69, 31.88 (×2), 31.52, 29.97, 21.62, 19.16.

HRMS (ESI) m/z calcd. for $C_{28}H_{33}N_2O_5$ $(M + H)^+$: 477.2389; Found: 477.2395.

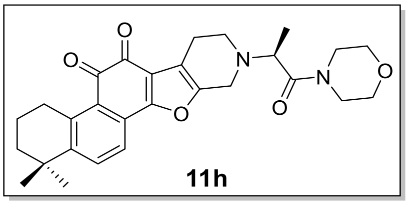

(*S*)-3,3-dimethyl-11-(1-morpholino-1-oxopropan-2-yl)-3,4,5,6,9,10,11,12-octahydro phenanthro[2',1':4,5]furo[2,3-*c*]pyridine-7,8-dione

Red solid (73.7 % yield). Rf = 0.3 (DCM / MeOH = 25:1); m.p. 134-139 °C.

^{1}H NMR (400 MHz, $CDCl_3$) δ 7.63 (d, *J* = 8.2 Hz, 1H), 7.51 (d, *J* = 8.2 Hz, 1H), 3.87 – 3.49 (m, 11H), 3.20 (t, *J* = 6.4 Hz, 2H), 2.87 (m, 2H), 2.79 (m, 2H), 1.88 – 1.78 (m, 2H), 1.69 (m, 2H), 1.35 (m, 9H).

^{13}C NMR (101 MHz, $CDCl_3$) δ 183.52, 175.75, 170.54, 160.71, 151.40, 150.01, 145.44, 133.49, 127.61, 126.26, 120.03, 119.49, 115.78, 67.12, 59.78, 46.60, 46.37, 45.13, 42.52, 37.88, 34.69, 31.87 (×2), 29.95, 22.35, 19.16, 10.49.

HRMS (ESI) m/z calcd. for $C_{28}H_{33}N_2O_5$ $(M + H)^+$: 477.2389; Found: 477.2395.

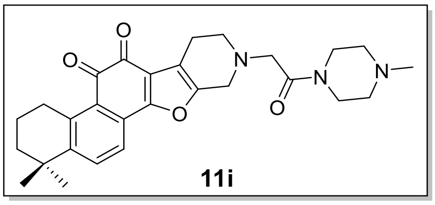

3,3-dimethyl-11-(2-(4-methylpiperazin-1-yl)-2-oxoethyl)-3,4,5,6,9,10,11,12-octahydro phenanthro[2',1':4,5]furo[2,3-*c*]pyridine-7,8-dione

Red solid (70.2 % yield). Rf = 0.2 (DCM / MeOH = 20:1); m.p. 143-146 ^{o}C.

^{1}H NMR (500 MHz, CDCl$_3$) δ 7.61 (d, *J* = 8.2 Hz, 1H), 7.49 (d, *J* = 8.2 Hz, 1H), 3.73 (m, 2H), 3.66 (t, *J* = 5.2 Hz, 2H), 3.61 (t, *J* = 5.0 Hz, 2H), 3.46 (s, 2H), 3.17 (t, *J* = 6.4 Hz, 2H), 2.87 (d, *J* = 10.4 Hz, 2H), 2.84 – 2.79 (m, 2H), 2.40 (t, *J* = 5.1 Hz, 4H), 2.30 (s, 3H), 1.84 – 1.75 (m, 2H), 1.68 – 1.63 (m, 2H), 1.31 (s, 6H).

^{13}C NMR (126 MHz, CDCl$_3$) δ 183.51, 175.33, 167.65, 160.71, 150.85, 149.97, 144.67, 133.50, 127.57, 126.20, 120.05, 119.42, 115.66, 59.58, 55.34, 54.82, 49.87, 49.36, 46.07, 45.45, 41.77, 37.84, 34.66, 31.85 (×2), 29.95, 21.46, 19.12.

HRMS (ESI) m/z calcd. for $C_{28}H_{34}N_3O_4$ (M + H)$^{+}$: 476.2549; Found: 476.2541.

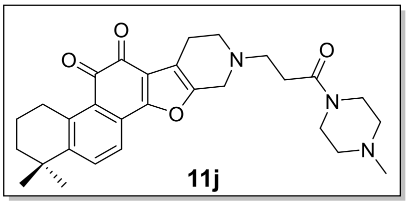

3,3-dimethyl-11-(3-(4-methylpiperazin-1-yl)-3-oxopropyl)-3,4,5,6,9,10,11,12-octahydro phenanthro[2',1':4,5]furo[2,3-*c*]pyridine-7,8-dione

Red solid (69.3 % yield). Rf = 0.2 (DCM / MeOH = 10:1); m.p. 145-150 ^{o}C.

^{1}H NMR (400 MHz, CDCl$_3$) δ 7.63 (d, *J* = 8.2 Hz, 1H), 7.51 (d, *J* = 8.2 Hz, 1H), 3.69 (bs, 4H), 3.60 – 3.53 (m, 2H), 3.20 (t, *J* = 6.4 Hz, 2H), 3.01 (t, *J* = 7.5 Hz, 2H), 2.84 (s, 4H), 2.66 (t, *J* = 7.4 Hz, 2H), 2.46 (m, 4H), 2.36 (s, 3H), 1.87 – 1.77 (m, 2H), 1.74 – 1.63 (m, 2H), 1.34 (s, 6H).

^{13}C NMR (101 MHz, CDCl$_3$) δ 183.54, 175.79, 169.78, 160.32, 151.14, 150.00, 144.70, 133.52, 128.18, 126.26, 120.06, 119.45, 115.87, 55.10, 54.68, 53.01, 50.13, 49.99, 45.97, 45.39, 41.48, 37.89, 34.69, 31.88 (×2), 31.60, 29.98, 21.60, 19.16.

HRMS (ESI) m/z calcd. for $C_{29}H_{36}N_3O_4$ (M + H)$^{+}$: 490.2706; Found: 490.2714.

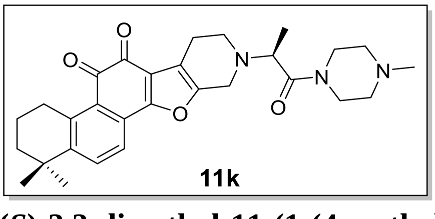

(*S*)-3,3-dimethyl-11-(1-(4-methylpiperazin-1-yl)-1-oxopropan-2-yl)-3,4,5,6,9,10,11,12-octahydrophenanthro[2',1':4,5]furo[2,3-*c*]pyridine-7,8-dione

Red solid (66.4 % yield). Rf = 0.3 (DCM / MeOH = 10:1); m.p. 138-145 °C.

^{1}H NMR (400 MHz, CDCl$_3$) δ 7.64 (d, *J* = 8.2 Hz, 1H), 7.51 (d, *J* = 8.2 Hz, 1H), 3.94 – 3.50 (m, 7H), 3.20 (t, *J* = 6.5 Hz, 2H), 2.85 (bs, 2H), 2.78 (s, 1H), 2.57 (bs, 2H), 2.41 - 2.35 (5H), 1.83 (m, 2H), 1.72 – 1.65 (m, 2H), 1.35 (bs, 9H).

^{13}C NMR (101 MHz, CDCl$_3$) δ 183.57, 174.59, 170.48, 160.74, 151.98, 150.00, 144.71, 133.54, 127.65, 126.21, 119.50, 115.79, 59.82, 55.43, 54.96, 46.58, 45.94, 45.42, 45.23, 41.82, 37.88, 34.69, 31.87 (×2), 29.97, 22.33, 19.15, 10.83.

HRMS (ESI) m/z calcd. for $C_{29}H_{36}N_3O_4$ $(M + H)^+$: 490.2706; Found: 490.2713.

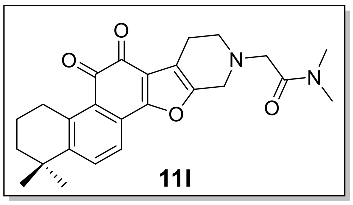

11l

2-(3,3-dimethyl-7,8-dioxo-3,5,6,7,8,9,10,12-octahydrophenanthro[2',1':4,5]furo[2,3-*c*]pyridin-11 (4H)-yl)-*N*,*N*-dimethylacetamide

Red solid (72.9 % yield). Rf = 0.2 (DCM / MeOH = 30:1); m.p. 136-140 °C.

^{1}H NMR (500 MHz, DMSO-*d6*) δ 7.79 (d, *J* = 8.2 Hz, 1H), 7.46 (d, *J* = 8.2 Hz, 1H), 4.54 (s, 2H), 4.44 (s, 2H), 3.55 (s, 2H), 3.06 (t, *J* = 6.3 Hz, 2H), 3.00 (t, *J* = 6.2 Hz, 2H), 2.95 (s, 6H), 1.78 – 1.70 (m, 2H), 1.66 – 1.60 (m, 2H), 1.30 (s, 6H).

^{13}C NMR (126 MHz, DMSO-*d6*) δ 181.90, 174.54, 164.75, 160.61, 150.31, 144.81, 143.70, 133.93, 126.78, 126.47, 120.30, 118.81, 114.95, 55.02, 50.71, 48.84, 37.79, 36.21, 35.52, 34.87, 31.94 (×2), 30.05, 19.16, 18.26.

HRMS (ESI) m/z calcd. for $C_{25}H_{29}N_2O_4$ $(M + H)^+$: 421.2127; Found: 421.2134.

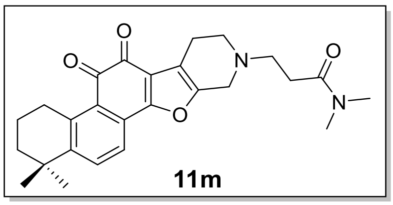

11m

3-(3,3-dimethyl-7,8-dioxo-3,5,6,7,8,9,10,12-octahydrophenanthro[2',1':4,5]furo[2,3-*c*]pyridin-11(4*H*)-yl)-*N*,*N*-dimethylpropanamide

Red solid (64.9 % yield). Rf = 0.2 (DCM / MeOH = 30:1); m.p. 133-147 °C.

^{1}H NMR (400 MHz, CDCl$_3$) δ 7.63 (d, *J* = 8.3 Hz, 1H), 7.52 (d, *J* = 8.2 Hz, 1H), 3.69 (s, 1H), 3.20 (d, *J* = 12.7 Hz, 2H), 3.08 (s, 3H), 3.01 (m, 5H), 2.84 (bs, 4H), 2.65 (t, *J* = 7.5 Hz, 2H), 1.88 – 1.77 (m, 2H), 1.72 – 1.64 (m, 2H), 1.34 (s, 6H).

^{13}C NMR (101 MHz, CDCl$_3$) δ 183.58, 175.32, 170.84, 160.71, 151.25, 149.95, 144.67, 133.49, 128.28, 126.28, 120.05, 119.05, 115.90, 53.01, 50.14, 50.01, 37.90, 37.29, 35.48, 34.68, 31.88 (×2), 31.74, 29.96, 21.63, 19.16.

HRMS (ESI) m/z calcd. for $C_{26}H_{31}N_2O_4$ $(M + H)^+$: 435.2284; Found: 435.2294.

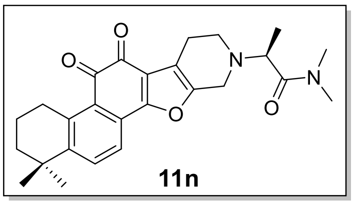

(*S*)-2-(3,3-dimethyl-7,8-dioxo-3,5,6,7,8,9,10,12-octahydrophenanthro[2',1':4,5]furo[2,3-*c*]pyridin-11(4*H*)-yl)-*N*,*N*-dimethylpropanamide

Red solid (58.6 % yield). Rf = 0.3 (DCM / MeOH = 50:1); m.p. 128-143 °C.

^{1}H NMR (500 MHz, CDCl$_3$) δ 7.56 (bs, 1H), 7.37 (bs, 1H), 3.84 (q, J = 6.9 Hz, 1H), 3.68 (bs, 2H), 3.16 – 3.10 (m, 5H), 3.01 – 2.94 (m, 4H), 2.76 (m, 3H), 1.78 m, 2H), 1.64 (m, 2H), 1.35 – 1.29 (m, 9H).

^{13}C NMR (126 MHz, CDCl$_3$) δ 151.65, 149.94, 144.81, 133.54, 119.44, 115.77, 59.33, 45.66, 37.82, 37.27, 36.00, 34.63, 31.86 (×2), 31.75, 29.93, 22.14, 19.09, 11.81.

HRMS (ESI) m/z calcd. for $C_{26}H_{31}N_2O_4$ $(M + H)^+$: 435.2284; Found: 435.2293.

General procedure for the synthesis of 12a–12b

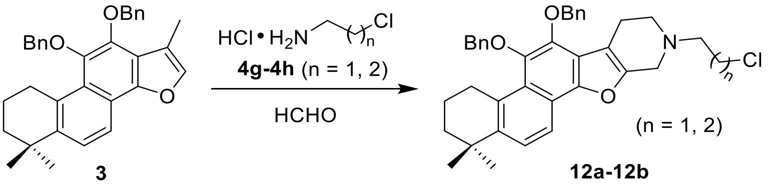

A sealed tube with a magnetic stirrer bar was charged with compound **3** (1 mmol), paraformaldehyde (8 mmol), and chloroalkylamine hydrochloride (4 mmol), then, anhydrous AcOH was added as solvent. The mixture was stirred at 90 °C for 4 h. After completion, the reaction was quenched by saturated aqueous $NaHCO_3$. The aqueous layer was extracted with EtOAc for three times, and the combined organic layer was washed with brine, dried over anhydrous Na_2SO_4, and concentrated in vacuo. The crude product was purified with silica gel column chromatography to give intermediates **12**.

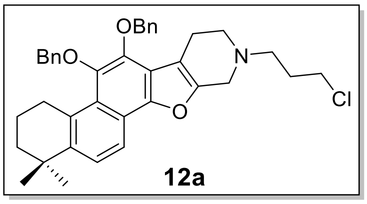

7,8-bis(benzyloxy)-11-(3-chloropropyl)-3,3-dimethyl-3,4,5,6,9,10,11,12-octahydro phenanthro[2',1':4,5]furo[2,3-*c*]pyridine

White solid (0.35 g, 58.3 % yield). Rf = 0.3 (Petroleum ether / EtOAc = 10:1); m.p. 111-114 °C.

^{1}H NMR (400 MHz, CDCl$_3$) δ 8.06 (d, *J* = 8.8 Hz, 1H), 7.53 (m, 3H), 7.44 – 7.33 (m, 8H), 5.25 (s, 2H), 5.09 (s, 2H), 3.80 (s, 2H), 3.71 (t, *J* = 6.5 Hz, 2H), 3.57 (t, *J* = 6.2 Hz, 2H), 2.85 (s, 4H), 2.81 (t, *J* = 7.0 Hz, 2H), 2.11 (p, *J* = 6.7 Hz, 2H), 1.82 (m, 2H), 1.79 – 1.71 (m, 2H), 1.42 (s, 6H).

^{13}C NMR (101 MHz, CDCl$_3$) δ 150.24, 148.04, 144.78, 144.61, 143.74, 142.85, 137.90, 137.63, 132.75, 128.42 (×5), 128.00, 127.90 (×2), 127.68, 125.77, 125.28, 118.69, 118.29, 117.75, 111.59, 76.36, 54.47, 50.76, 50.66, 43.11, 38.92, 35.00, 32.24 (×2), 30.84, 30.52, 22.33, 20.35.

General procedure for the synthesis of 13a–13c

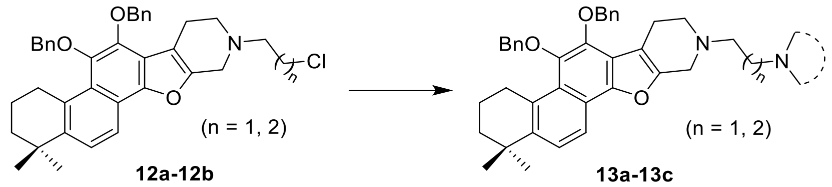

A solution of **12** (1 mmol), K_2CO_3 (3 mmol) and the corresponding amine (3 mmol) in DMF was stirred at room temperature until the complete consumption of **12**. The reaction solution was the diluted with brine and extracted with DCM for three times. The combined organic layer was washed with brine and dried over anhydrous Na_2SO_4, and concentrated in vacuo. The crude product was purified with silica gel chromatography using DCM/MeOH system as eluent to afford intermediates **13**.

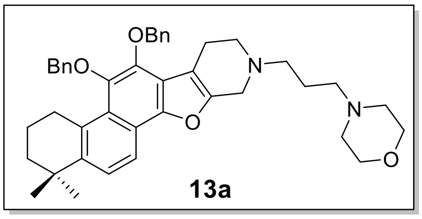

7,8-bis(benzyloxy)-3,3-dimethyl-11-(3-morpholinopropyl)-3,4,5,6,9,10,11,12-octahydro phenanthro[2',1':4,5]furo[2,3-*c*]pyridine

White solid (0.46 g, 71.9 % yield). Rf = 0.2 (DCM/MeOH = 25 :1); m.p. 123-127 ^{o}C.

^{1}H NMR (400 MHz, CDCl$_3$) δ 8.06 (s, 1H), 7.55 – 7.50 (m, 3H), 7.45 – 7.32 (m, 8H), 5.24 (s, 2H), 5.09 (s, 2H), 3.79 (bs, 6H), 3.56 (bs, 2H), 2.86 (bs, 4H), 2.71 (bs, 2H), 2.54 (bs, 6H), 2.00 – 1.64 (m, 6H), 1.41 (s, 6H).

^{13}C NMR (101 MHz, CDCl$_3$) δ 149.82, 148.12, 142.85, 137.89, 137.63, 132.44, 128.41 (×5), 127.98, 127.88 (×2), 127.67, 125.53, 125.27, 118.22, 117.74, 111.67, 76.36, 76.33, 66.90, 56.98, 55.56, 53.76 (×2), 50.71, 50.65, 50.60, 38.91, 34.99, 32.22 (×2), 30.83, 24.48, 22.25, 20.33.

General procedure for the synthesis of 14a–14c

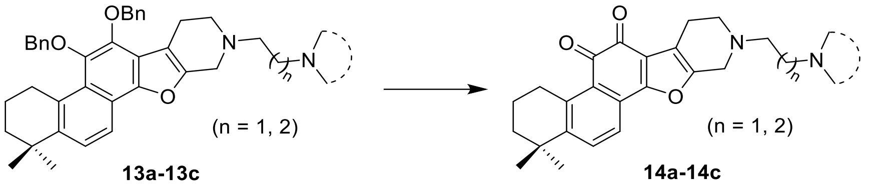

According to the procedure for preparing compounds **6**, compounds **14a**–**14c** was synthesized from **13a** – **13c** through *de*-benzylation and oxidation reactions.

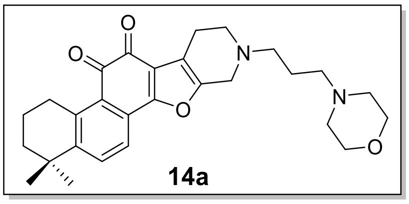

3,3-dimethyl-11-(3-morpholinopropyl)-3,4,5,6,9,10,11,12-octahydrophenanthro [2',1':4,5]furo[2,3-*c*] pyridine-7,8-dione

Red solid (60.1 % yield). Rf = 0.2 (DCM / MeOH = 20:1); m.p. 139-144 °C.

^{1}H NMR (500 MHz, $CDCl_3$) δ 7.61 (d, *J* = 8.2 Hz, 1H), 7.50 (d, *J* = 8.1 Hz, 1H), 3.73 (t, *J* = 4.7 Hz, 4H), 3.60 (t, *J* = 1.8 Hz, 2H), 3.17 (t, *J* = 6.4 Hz, 2H), 2.81 – 2.75 (m, 4H), 2.64 (t, *J* = 7.4 Hz, 2H), 2.47 (s, 4H), 2.47 – 2.40 (m, 2H), 1.83 – 1.76 (m, 4H), 1.68 – 1.63 (m, 2H), 1.31 (s, 6H).

^{13}C NMR (126 MHz, $CDCl_3$) δ 183.55, 175.30, 160.65, 151.35, 149.90, 144.64, 133.46, 127.63, 126.21, 119.99, 119.46, 115.92, 77.28, 66.92 (×2), 56.86, 55.45, 53.76 (×2), 50.12, 49.79, 37.84, 34.65, 31.84(×2), 29.94, 24.51, 21.55, 19.12.

HRMS (ESI) m/z calcd. for $C_{28}H_{35}N_2O_4$ $(M + H)^+$: 463.2597; Found: 463.2591.

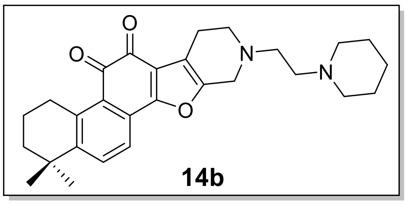

3,3-dimethyl-11-(2-(piperidin-1-yl)ethyl)-3,4,5,6,9,10,11,12-octahydrophenanthro [2',1':4,5]furo [2,3-*c*]pyridine-7,8-dione

Red solid (54.3 % yield). Rf = 0.2 (DCM / MeOH = 10:1); m.p. 141-145 °C.

^{1}H NMR (400 MHz, $CDCl_3$) δ 7.62 (s, 1H), 7.52 (d, *J* = 8.2 Hz, 2H), 3.70 (bs, 2H), 3.20 (t, *J* = 6.4 Hz, 2H), 2.82 (m, 6H), 2.63 (t, *J* = 7.1 Hz, 2H), 2.53 (bs, 4H), 2.13 – 2.07 (m, 2H), 1.82 (m, 2H), 1.67 (m, 4H), 1.49 (m,2H), 1.34 (s, 6H).

^{13}C NMR (101 MHz, $CDCl_3$) δ 183.60, 175.33, 160.68, 151.36, 149.93, 144.67, 133.50, 127.68, 126.25, 120.05, 119.50, 115.88, 57.09, 55.12 (×2), 54.51, 50.44, 50.08, 37.90, 34.68, 31.88 (×2), 29.97, 25.75 (×2), 24.20, 21.50, 19.17.

HRMS (ESI) m/z calcd. for $C_{28}H_{35}N_2O_3$ $(M + H)^+$: 447.2648; Found: 447.2659.

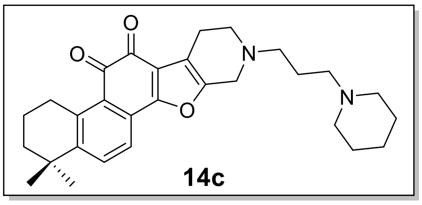

3,3-dimethyl-11-(3-(piperidin-1-yl)propyl)-3,4,5,6,9,10,11,12-octahydrophenanthro [2',1':4,5]furo [2,3-*c*]pyridine-7,8-dione

Red solid (51.2 % yield). Rf = 0.2 (DCM / MeOH = 10:1); m.p. 145-150 °C.

^{1}H NMR (400 MHz, CDCl$_3$) δ 7.64 (d, *J* = 8.2 Hz, 1H), 7.53 (d, *J* = 8.3 Hz, 1H), 3.67 (bs, 2H), 3.24 – 3.07 (m, 6H), 2.80 (s, 4H), 2.75 (t, *J* = 6.3 Hz, 2H), 2.24 (m, 2H), 2.07 (m, 6H), 1.86 – 1.79 (m, 2H), 1.68 (bs, 2H), 1.43 (t, *J* = 7.4 Hz, 2H), 1.34 (s, 6H).

^{13}C NMR (101 MHz, CDCl$_3$) δ 183.51, 175.27, 160.83, 151.04, 150.04, 144.65, 133.59, 127.56, 126.16, 120.19, 118.71, 115.77, 55.79, 54.08, 53.59 (×2), 50.22, 49.41, 37.87, 34.69, 31.86 (×2), 29.98, 22.70 (×2), 22.19, 21.59, 21.50, 19.15.

HRMS (ESI) m/z calcd. for $C_{29}H_{37}N_2O_3$ $(M + H)^+$: 461.2804; Found: 461.2810.

Procedure for the synthesis of 15

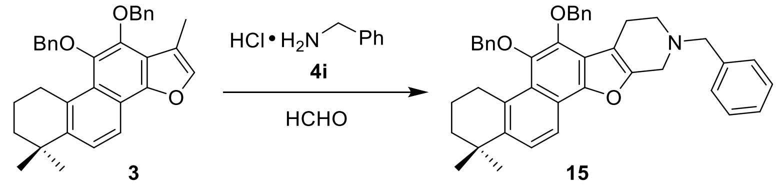

A sealed tube with a magnetic stirrer bar was charged with compound **3** (0.48 g, 1 mmol), paraformaldehyde (0.24 g, 8 mmol, 8 equiv), Benzylamine hydrochloride **4i** (4 mmol, 4 equiv), and anhydrous AcOH 15 mL was added as solvent. The reaction mixture was stirred at 90 °C and monitored by TLC. After completion, the reaction was quenched by saturated aqueous $NaHCO_3$. The aqueous layer was extracted with EtOAc (50 mL × 3), and the combined organic layer was washed with brine, dried over anhydrous Na_2SO_4, concentrated in vacuo. The residue was purified by silica gel column chromatography to give the intermediates **15**.

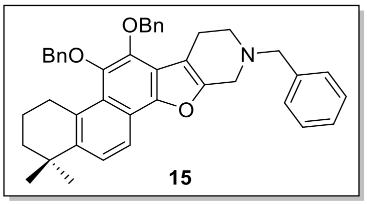

11-benzyl-7,8-bis(benzyloxy)-3,3-dimethyl-3,4,5,6,9,10,11,12-octahydrophenanthro [2',1':4,5]furo [2,3-*c*] pyridine

White solid (59.2 % yield). Rf = 0.2 (Petroleum Ether / EtOAc = 10:1); m.p. 132-135 °C.

^{1}H NMR (400 MHz, CDCl$_3$) δ 8.03 (d, *J* = 8.8 Hz, 1H), 7.56 – 7.49 (m, 3H), 7.48 – 7.31 (m, 13H), 5.25 (s, 2H), 5.09 (s, 2H), 3.82 (s, 2H), 3.78 (s, 2H), 3.57 (t, *J* = 6.2 Hz, 2H), 2.87 (s, 4H), 1.83 (m, 2H), 1.74 (m, 2H), 1.42 (s, 6H).

^{13}C NMR (101 MHz, CDCl$_3$) δ 150.52, 148.02, 144.77, 144.63, 142.78, 138.18, 137.92, 137.63, 132.72, 129.24 (×2), 128.47 (×2), 128.42 (×5), 127.98, 127.90 (×2), 127.67, 127.38, 125.71, 125.25, 118.40, 118.30, 117.75, 111.53, 76.34, 76.30, 62.08, 50.55, 50.40, 38.93, 34.99, 32.24 (×2), 30.84, 22.39, 20.35.

Procedure for the synthesis of 16

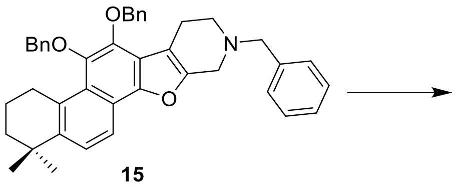

A two-necked flask equipped with a magnetic stirrer bar was charged with **15** (0.5 mmol) and 10% Pd/C (5% Palladium on activated carbon, wetted with ca.55% water), anhydrous THF 5 mL and MeOH 5 mL was added, and purged with hydrogen several times. The resulting mixture was stirred under a hydrogen atmosphere overnight. The debenzylation and following oxidation upon air exposure of **15** to deliver **16**.

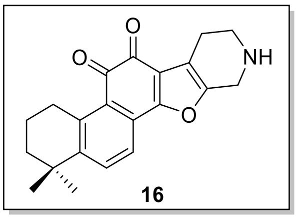

3,3-dimethyl-3,4,5,6,9,10,11,12-octahydrophenanthro[2',1':4,5]furo[2,3-*c*]pyridine-7,8-dione

Red solid (49.2 % yield). Rf = 0.2 (DCM / MeOH = 20:1); m.p. 124-128 °C.

^{1}H NMR (400 MHz, DMSO-*d6*) δ 7.77 (m, 1H), 7.52 (m, 1H), 3.80 (s, 2H), 3.07 (t, *J* = 6.7 Hz, 2H), 2.89 (t, *J* = 5.6 Hz, 2H), 2.57 (t, *J* = 6.2 Hz, 2H), 1.79 – 1.67 (m, 2H), 1.62 (m, 2H), 1.30 (s, 6H).

^{13}C NMR (101 MHz, DMSO-*d6*) δ 153.50, 149.45, 143.46, 133.93, 127.30, 120.15, 119.52, 116.21, 42.56, 42.41, 37.92, 34.80, 31.98 (×2), 30.03, 23.40, 19.24.

General procedure for the synthesis of 17a–17k

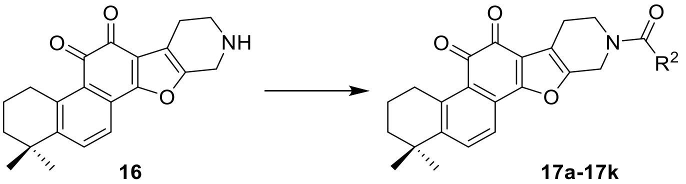

A two-necked flask equipped with a magnetic stirrer bar was charged with **16** (0.5 mmol), corresponding acid (0.75 mmol), HATU (1 mmol), the anhydrous DCM 15 mL and DIPEA (1.5 mol) was added. The reaction mixture was stirred and kept at room temperature overnight. Upon completion, brine was added to the reaction solution, and the aqueous layer was extracted with DCM for three times. The combined organic layer was washed with brine, dried over anhydrous Na_2SO_4 and concentrated in vacuo. The crude product was further purified by silica column chromatography using DCM/MeOH as eluent to afford the targets compounds **17a**–**17k**.

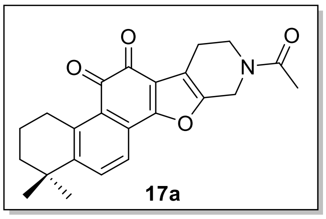

11-acetyl-3,3-dimethyl-3,4,5,6,9,10,11,12-octahydrophenanthro[2',1':4,5]furo[2,3-*c*]pyridine-7,8-dione

Red solid (81.1 % yield). Rf = 0.3 (DCM / MeOH = 100:1); m.p. 146-150 °C.

^{1}H NMR (400 MHz, $CDCl_3$) δ 7.64 (d, *J* = 8.2 Hz, 1H), 7.52 (d, *J* = 8.1 Hz, 1H), 4.74 (s, 2H), 3.73 (t, *J* = 5.6 Hz, 2H), 3.19 (t, *J* = 6.3 Hz, 2H), 2.89 (t, *J* = 5.6 Hz, 3H), 2.24 (s, 3H), 1.86 – 1.79 (m, 2H), 1.70 – 1.67 (m, 2H), 1.34 (s, 6H).

^{13}C NMR (101 MHz, $CDCl_3$) δ 183.10, 175.10, 169.94, 161.11, 150.39, 149.80, 144.86, 133.63, 127.21, 126.19, 120.20, 119.10, 115.73, 43.85, 40.00, 37.83, 34.74, 31.87 (×2), 30.01, 22.28, 21.59, 19.13.

HRMS (ESI) m/z calcd. for $C_{23}H_{24}NO_4$ $(M + H)^+$: 378.1705; Found: 378.1720.

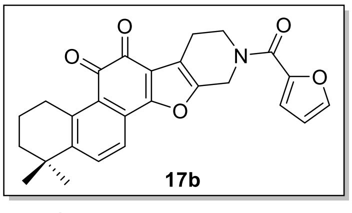

11-(furan-2-carbonyl)-3,3-dimethyl-3,4,5,6,9,10,11,12-octahydrophenanthro [2',1':4,5]furo[2,3-*c*] pyridine-7,8-dione

Red solid (83.3 % yield). Rf = 0.3 (DCM / MeOH = 100:1); m.p. 151-154 °C.

^{1}H NMR (400 MHz, $CDCl_3$) δ 7.67 – 7.64 (m, 3H), 7.51 (d, J = 8.1 Hz, 1H), 7.19 – 7.16 (m, 1H), 4.40 – 4.39 (s, 2H), 3.50 (t, J = 5.7 Hz, 2H), 3.18 (t, J = 6.4 Hz, 2H), 2.90 (td, J = 5.6, 2.8 Hz, 2H), 1.86 – 1.78 (m, 2H), 1.70 – 1.64 (m, 2H), 1.34 (s, 6H).

^{13}C NMR (101 MHz, CDCl$_3$) δ 182.95, 174.96, 161.26, 150.59, 147.69, 144.95, 137.01, 133.62, 132.73, 132.60, 127.82, 127.01, 126.27, 120.16, 118.91, 116.00, 43.63, 43.39, 37.80, 34.75, 31.87 (×2), 30.00, 21.47, 19.11.

HRMS (ESI) m/z calcd. for $C_{26}H_{24}NO_5$ $(M + H)^+$: 430.1654; Found: 430.1661.

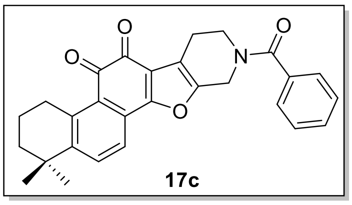

11-benzoyl-3,3-dimethyl-3,4,5,6,9,10,11,12-octahydrophenanthro[2',1':4,5]furo[2,3-*c*]pyridine-7,8-dione

Red solid (83.9 % yield). Rf = 0.3 (DCM / MeOH = 100:1); m.p. 155-160 ºC.

^{1}H NMR (400 MHz, DMSO-*d6*) δ 7.75 (s, 1H), 7.52 (s, 6H), 4.74 – 4.56 (2H), 3.93, 3.58 (2H), 3.04 (bs, 2H), 2.72 (bs, 2H), 1.77 – 1.70 (m, 2H), 1.63 (bs, 2H), 1.29 (s, 6H).

^{13}C NMR (101 MHz, DMSO-*d6*) δ 171.45, 149.86, 143.57, 133.90, 129.13, 126.92, 118.82, 37.87, 35.44, 32.54, 30.04(×2), 20.44.

HRMS (ESI) m/z calcd. for $C_{28}H_{26}NO_4$ $(M + H)^+$: 440.1862; Found: 440.1858.

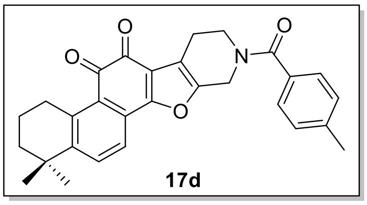

3,3-dimethyl-11-(4-methylbenzoyl)-3,4,5,6,9,10,11,12-octahydrophenanthro [2',1':4,5]furo[2,3-*c*]pyridine-7,8-dione

Red solid (84.4 % yield). Rf = 0.3 (DCM / MeOH = 100:1); m.p. 157-162 ºC.

^{1}H NMR (400 MHz, CDCl3) δ 7.62 (d, *J* = 8.5 Hz, 1H), 7.52 (bs, 1H), 7.37 (d, *J* = 8.2 Hz, 2H), 7.26 (d, *J* = 8.2 Hz, 2H), 4.81 (s, 2H), 3.68 (s, 2H), 3.18 (t, *J* = 6.4 Hz, 2H), 2.86 (s, 2H), 2.41 (s, 3H), 1.80 (m, 2H), 1.71 – 1.60 (m, 2H), 1.32 (s, 6H).

^{13}C NMR (101 MHz, CDCl3) δ 183.18, 175.16, 171.62, 161.19, 150.43, 149.50, 144.90, 140.61, 133.62, 132.50, 129.35(×2), 127.26, 127.12, 126.28, 120.19, 119.24, 45.17, 40.92, 37.85, 34.75, 31.88 (×2), 30.01, 22.48, 21.49, 19.14.

HRMS (ESI) m/z calcd. for $C_{29}H_{28}NO_4$ $(M + H)^+$: 454.2018; Found: 454.2028.

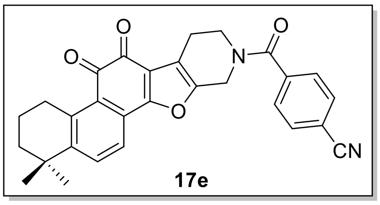

4-(3,3-dimethyl-7,8-dioxo-3,4,5,6,7,8,9,10,11,12-decahydrophenanthro[2',1':4,5]furo[2,3-*c*] pyridine-11-carbonyl)benzonitrile

Red solid (84.1 % yield). Rf = 0.3 (DCM / MeOH = 100:1); m.p. 154-158 ºC.

^{1}H NMR (500 MHz, $CDCl_3$) δ 7.78 (d, *J* = 8.2 Hz, 2H), 7.64 (d, *J* = 7.8 Hz, 1H), 7.59 (d, *J* = 8.1 Hz, 2H), 7.52 (d, *J* = 9.2 Hz, 1H), 4.87 – 4.50 (2H), 4.04 - 3.61 (2H), 3.18 (t, *J* = 6.4 Hz, 2H), 2.95 - 2.84 (2H), 1.85 – 1.77 (m, 2H), 1.67 (m, *J* = 5.5 Hz, 2H), 1.33 (s, 6H).

^{13}C NMR (126 MHz, $CDCl_3$) δ 182.89, 174.99, 169.55, 168.00, 161.24, 150.64, 144.99, 139.68, 133.63, 132.67, 127.63, 127.00, 126.23, 120.20, 119.25, 117.93, 115.80, 114.16, 45.04, 40.75, 37.77, 34.74, 31.84 (×2), 29.98, 22.35, 19.07.

HRMS (ESI) m/z calcd. for $C_{29}H_{25}N_2O_4$ $(M + H)^+$: 465.1814; Found: 465.1811.

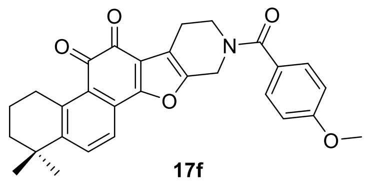

17f

11-(4-methoxybenzoyl)-3,3-dimethyl-3,4,5,6,9,10,11,12-octahydrophenanthro [2',1':4,5]furo[2,3-*c*] pyridine-7,8-dione

Red solid (83.8 % yield). Rf = 0.3 (DCM / MeOH = 100:1); m.p. 157-161 ºC.

^{1}H NMR (500 MHz, $CDCl_3$) δ 7.62 (d, *J* = 8.1 Hz, 1H), 7.82 – 7.42 (3H), 6.96 (d, J = 8.5 Hz, 2H), 4.77 (s, 2H), 3.86 (s, 3H), 3.75 (s, 2H), 3.17 (t, *J* = 6.5 Hz, 2H), 2.88 (s, 2H), 1.80 (m, *J* = 6.3 Hz,2H), 1.69 – 1.63 (m, 2H), 1.32 (s, 6H).

^{13}C NMR (126 MHz, $CDCl_3$) δ 183.08, 175.07, 171.43, 161.27, 161.13, 150.39, 149.55, 144.86, 133.58, 129.86, 129.12, 127.43, 127.20, 126.22, 120.12, 119.21, 113.96, 52.17, 36.74, 34.51, 31.84 (×2), 29.97, 21.60, 20.55.

HRMS (ESI) m/z calcd. for $C_{29}H_{28}NO_5$ $(M + H)^+$: 470.1967; Found: 470.1972.

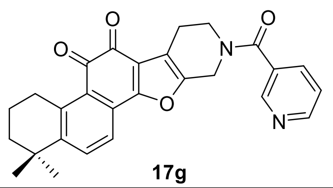

17g

3,3-dimethyl-11-nicotinoyl-3,4,5,6,9,10,11,12-octahydrophenanthro[2',1':4,5]furo[2,3-*c*]pyridine-7,8-dione

Red solid (81.7 % yield). Rf = 0.2 (DCM / MeOH = 100:1); m.p. 151-155 ºC.

^{1}H NMR (500 MHz, $CDCl_3$) δ 8.78 – 8.71 (m, 2H), 7.83 (d, *J* = 6.7 Hz, 1H), 7.68 – 7.36 (m, 3H), 4.87 – 4.60 (2H), 4.09 - 3.68 (2H), 3.18 (t, *J* = 6.4 Hz, 2H), 2.95 (2H), 1.84 – 1.78 (m, 2H), 1.70 – 1.64 (m, 2H), 1.32 (s, 6H).

^{13}C NMR (126 MHz, CDCl$_3$) δ 182.94, 174.99, 168.46, 161.30, 151.36, 150.57, 148.90, 147.86, 144.95, 134.87, 133.61, 131.32, 127.05, 126.24, 123.64, 120.19, 119.08, 115.31, 45.44, 40.85, 37.40, 34.73, 31.84 (×2), 29.98, 22.51, 19.08.

HRMS (ESI) m/z calcd. for $C_{27}H_{25}N_2O_4$ (M + H)$^+$: 441.1814; Found: 441.1823.

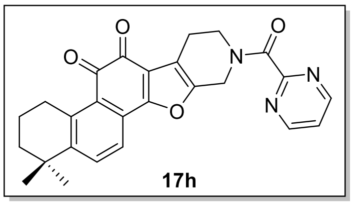

17h

3,3-dimethyl-11-(pyrimidine-2-carbonyl)-3,4,5,6,9,10,11,12-octahydrophenanthro [2',1':4,5]furo [2,3-*c*]pyridine-7,8-dione

Red solid (79.6 % yield). Rf = 0.3 (DCM / MeOH = 50:1); m.p. 146-151 ^{o}C.

^{1}H NMR (500 MHz, CDCl$_3$) δ 8.88 (m, 2H), 7.66 – 7.57 (2H), 7.56, 7.53, 7.41, 7.39 (2H), 7.43 (t, *J* = 5.0 Hz, 1H), 4.93, 4.57 (2H), 4.10 – 4.09, 3.61 – 3.59 (2H), 3.19 (m, 2H), 2.97, 2.91 (2H), 1.81 (m, 2H), 1.66 (m, 2H), 1.31, 1.30 (s, 6H).

^{13}C NMR (126 MHz, CDCl$_3$) δ 183.08, 175.06, 166.12, 161.79, 161.26, 157.57 (×2), 150.45, 148.67, 144.87, 133.64, 127.17, 126.22, 121.60, 120.24, 119.13, 116.19, 44.49, 40.39, 37.81, 34.72, 31.82 (×2), 29.98, 22.16, 19.10.

HRMS (ESI) m/z calcd. for $C_{27}H_{25}N_2O_4$ (M + H)$^+$: 442.1767; Found: 442.1777.

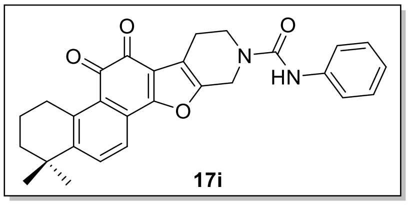

17i

3,3-dimethyl-7,8-dioxo-*N*-phenyl-3,5,6,7,8,9,10,12-octahydrophenanthro [2',1':4,5]furo[2,3-*c*] pyridine-11(4*H*)-carboxamide

Red solid (61.4 % yield). Rf = 0.2 (DCM / MeOH = 50:1); m.p. 141-145 ^{o}C.

^{1}H NMR (500 MHz, CDCl$_3$) δ 7.61 (d, *J* = 8.2 Hz, 1H), 7.48 (d, *J* = 7.7 Hz, 1H), 7.38 (d, *J* = 7.3 Hz, 2H), 7.30 (t, *J* = 7.8 Hz, 2H), 7.06 (t, *J* = 7.3 Hz, 1H), 6.73 (bs, 1H), 4.66 (s, 2H), 3.74 (t, *J* = 5.6 Hz, 2H), 3.16 (t, *J* = 6.4 Hz, 2H), 2.89 (t, *J* = 6.2 Hz, 2H), 1.84 – 1.75 (m, 2H), 1.69 – 1.63 (m, 2H), 1.32 (s, 6H).

^{13}C NMR (126 MHz, CDCl$_3$) δ 183.13, 175.11, 161.10, 155.27, 150.40, 149.72, 145.65, 138.69, 133.60, 128.97, 127.22, 126.19, 123.55, 120.37, 120.14, 119.18, 116.39, 42.50, 42.00, 37.80, 34.71, 31.84 (×2), 29.98, 21.89, 19.09.

HRMS (ESI) m/z calcd. for $C_{28}H_{27}N_2O_4$ (M + H)$^+$: 455.1971; Found: 455.1977.

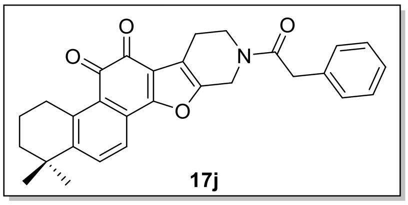

3,3-dimethyl-11-(2-phenylacetyl)-3,4,5,6,9,10,11,12-octahydrophenanthro [2',1':4,5]furo[2,3-*c*]pyridine-7,8-dione

Red solid (82.9 % yield). Rf = 0.3 (DCM / MeOH = 100:1); m.p. 155-160 ºC.

^{1}H NMR (500 MHz, CDCl$_3$) δ 7.59 (s, 1H), 7.47 (d, *J* = 8.1 Hz, 1H), 7.33 - 7.27 (5H), 4.74, 4.52 (s, 2H), 3.89, 3.70 (t, *J* = 5.8 Hz, 2H), 3.83 (s, 2H), 3.16 (t, *J* = 6.4 Hz, 2H), 2.81, 2.58 (s, 2H), 1.85 – 1.76 (m, 2H), 1.70 – 1.62 (m, 2H), 1.31 (s, 6H).

^{13}C NMR (126 MHz, CDCl$_3$) δ 183.04, 175.00, 170.51, 161.02, 150.36, 149.65, 144.77, 134.54, 133.53, 128.92 (×2), 128.57 (×2), 127.18, 127.02, 126.19, 120.12, 119.07, 115.73, 43.59, 41.36, 40.27, 37.83, 34.69, 31.82 (×2), 29.91, 22.07, 19.09.

HRMS (ESI) m/z calcd. for $C_{29}H_{28}NO_4$ $(M + H)^+$: 454.2018; Found: 454.2029.

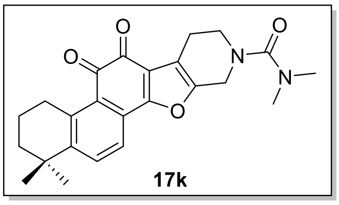

***N*,*N*,3,3-tetramethyl-7,8-dioxo-3,5,6,7,8,9,10,12-octahydrophenanthro[2',1':4,5]furo[2,3-*c*]pyridine -11(4*H*) -carboxamide**

Red solid (78.4 % yield). Rf = 0.3 (DCM / MeOH = 50:1); m.p. 140-145 ºC.

^{1}H NMR (400 MHz, CDCl$_3$) δ 7.61 (d, *J* = 8.2 Hz, 1H), 7.50 (d, *J* = 8.1 Hz, 1H), 4.33 (s, 2H), 3.46 (t, *J* = 5.5 Hz, 2H), 3.17 (t, *J* = 6.3 Hz, 2H), 2.91 (s, 6H), 2.87 (t, *J* = 5.4 Hz, 2H), 1.84 – 1.76 (m, 2H), 1.69 – 1.63 (m, 2H), 1.32 (s, 6H).

^{13}C NMR (101 MHz, CDCl$_3$) δ 183.32, 175.18, 164.63, 160.82, 150.64, 150.16, 144.76, 133.54, 127.44, 126.23, 120.11, 119.39, 116.40, 45.25, 44.15, 38.51 (×2), 37.88, 34.73, 31.89 (×2), 30.00, 21.89, 19.16.

HRMS (ESI) m/z calcd. for $C_{24}H_{27}N_2O_4$ $(M + H)^+$: 454.2018; Found: 454.2029.

3. HPLC Trace for 8b

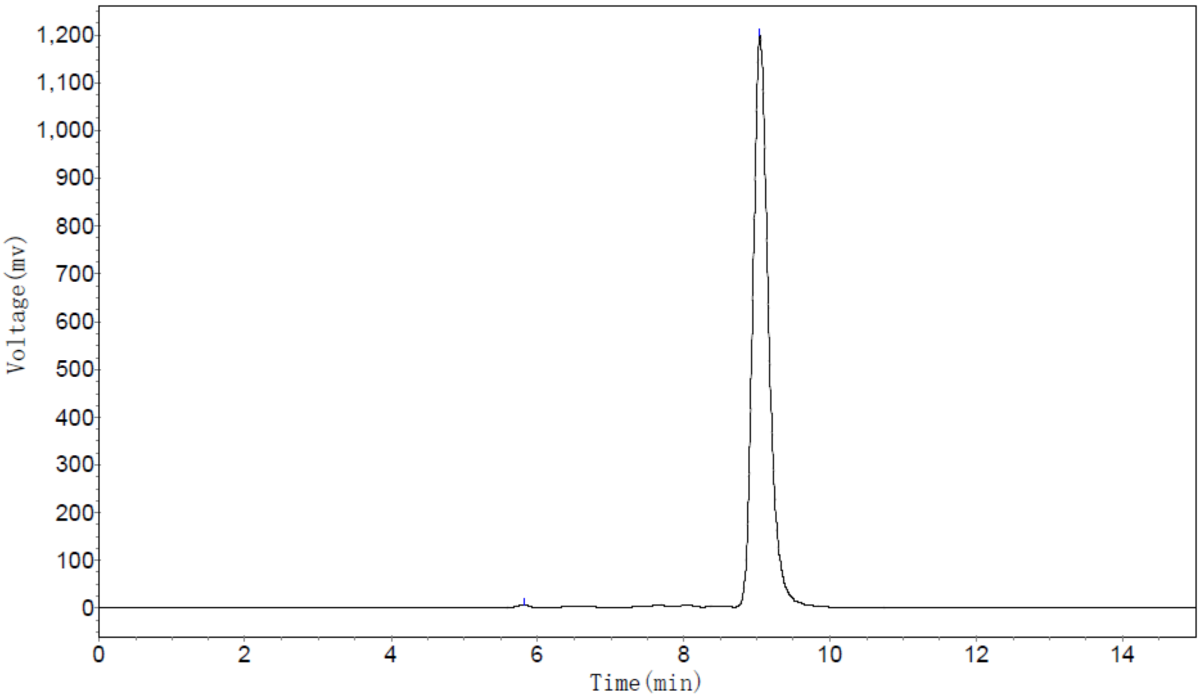

<Peak Table>

Detector A 254 nm

Peak #	Ret. Time	Area %
1	5.817	0.588
2	9.043	99.412
Total		100

4. Copies of NMR Spectra

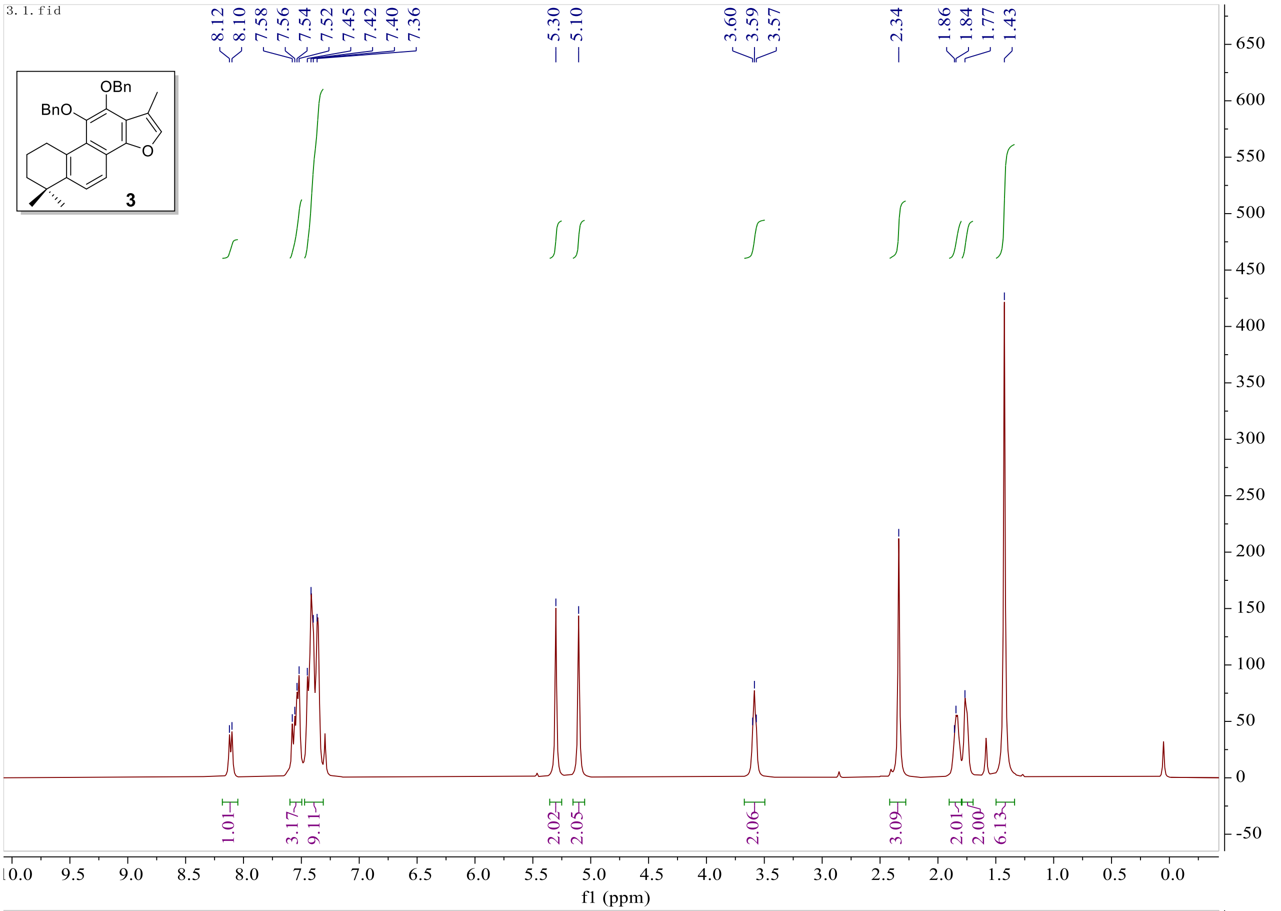

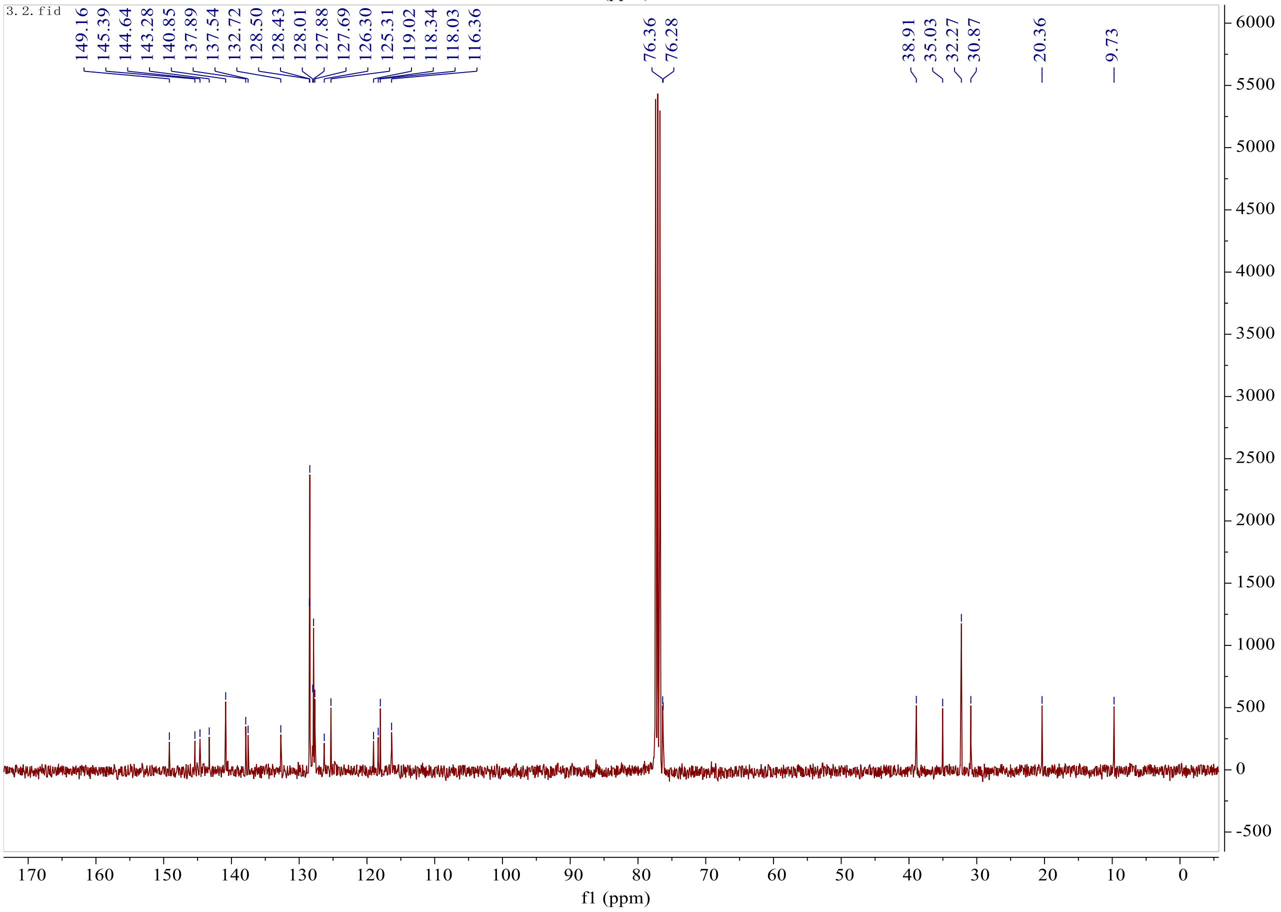

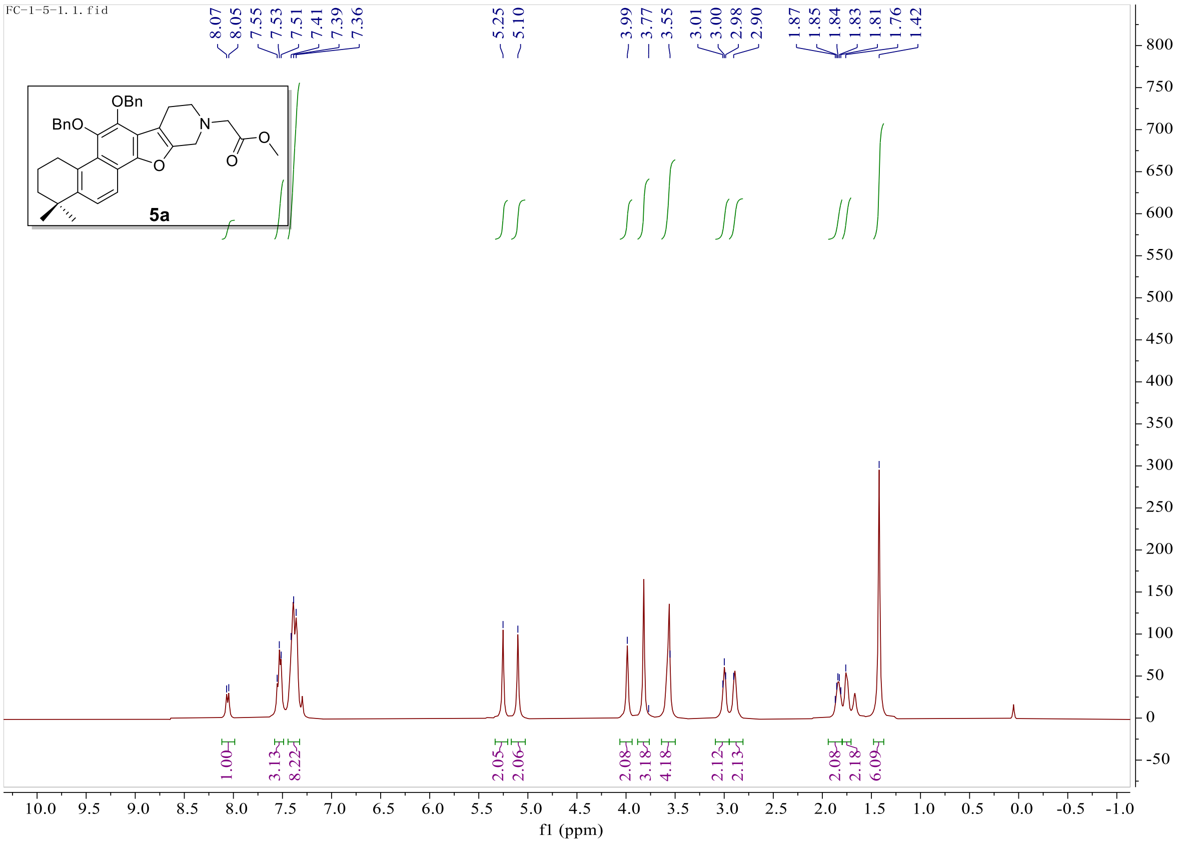
FC-1-5-1.1.fid
8.07
8.05
7.55
7.53
7.51
7.41
7.39
7.36
5.25
5.10
3.99
3.77
3.55
3.01
3.00
2.98
2.90
1.87
1.85
1.84
1.83
1.81
1.76
1.42
OBn
BnO
N
O
O
O
5a
1.00
3.13
8.22
2.05
2.06
2.08
3.18
4.18
2.12
2.13
2.08
2.18
6.09
f1 (ppm)

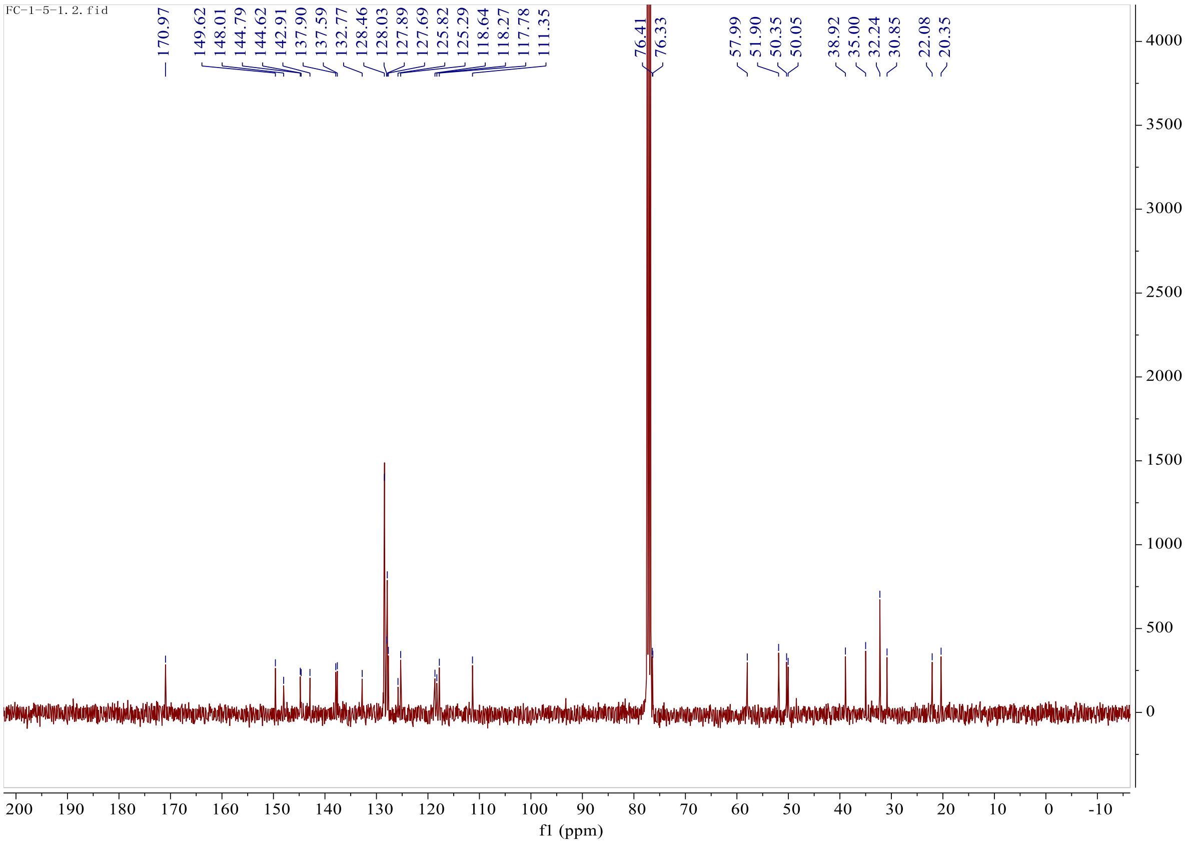
FC-1-5-1.2.fid
170.97
149.62
148.01
144.79
144.62
142.91
137.90
137.59
132.77
128.46
128.03
127.89
127.69
125.82
125.29
118.64
118.27
117.78
111.35
76.41
76.33
57.99
51.90
50.35
50.05
38.92
35.00
32.24
30.85
22.08
20.35
f1 (ppm)

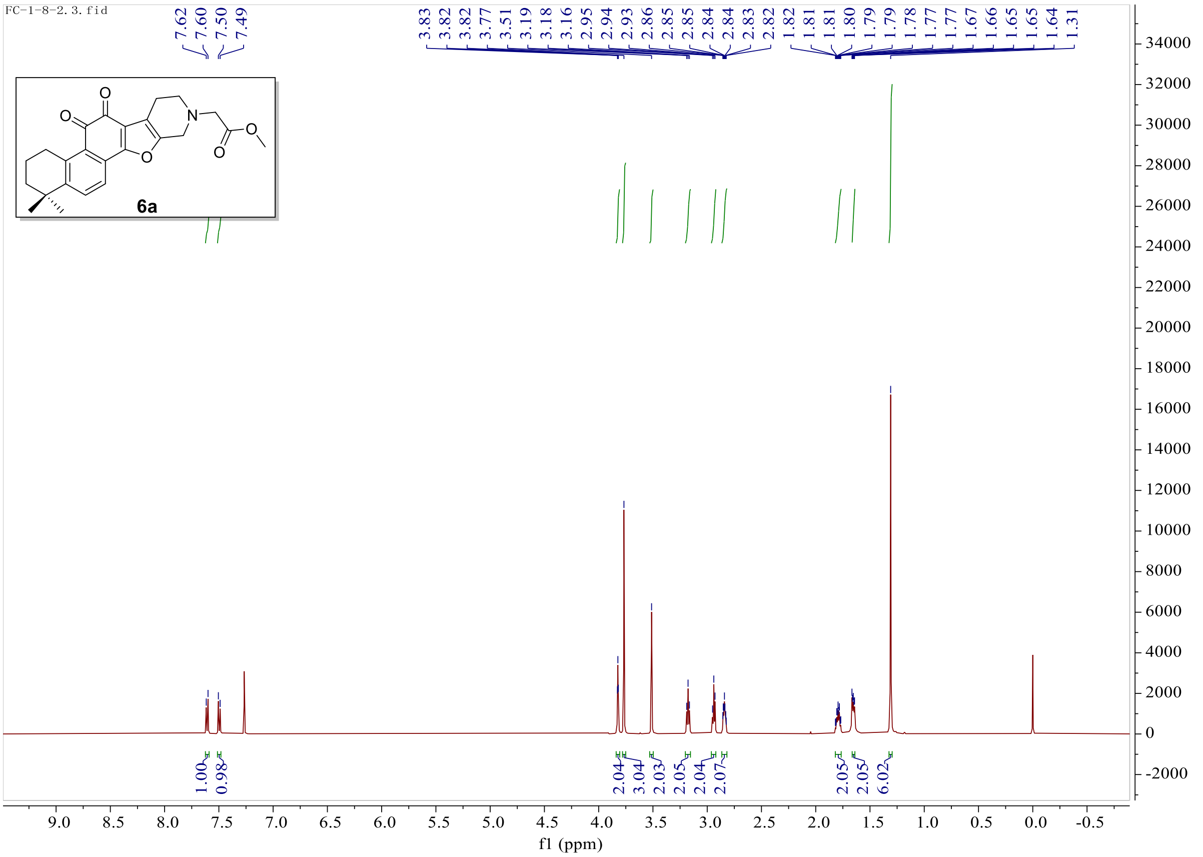
FC-1-8-2.3.fid
7.62
7.60
7.50
7.49
3.83
3.82
3.82
3.77
3.51
3.19
3.18
3.16
2.95
2.94
2.93
2.86
2.85
2.85
2.84
2.84
2.83
2.82
1.82
1.81
1.81
1.80
1.79
1.79
1.78
1.77
1.77
1.67
1.66
1.65
1.65
1.64
1.31
6a
1.00
0.98
2.04
3.04
2.03
2.05
2.04
2.07
2.05
2.05
6.02
f1 (ppm)

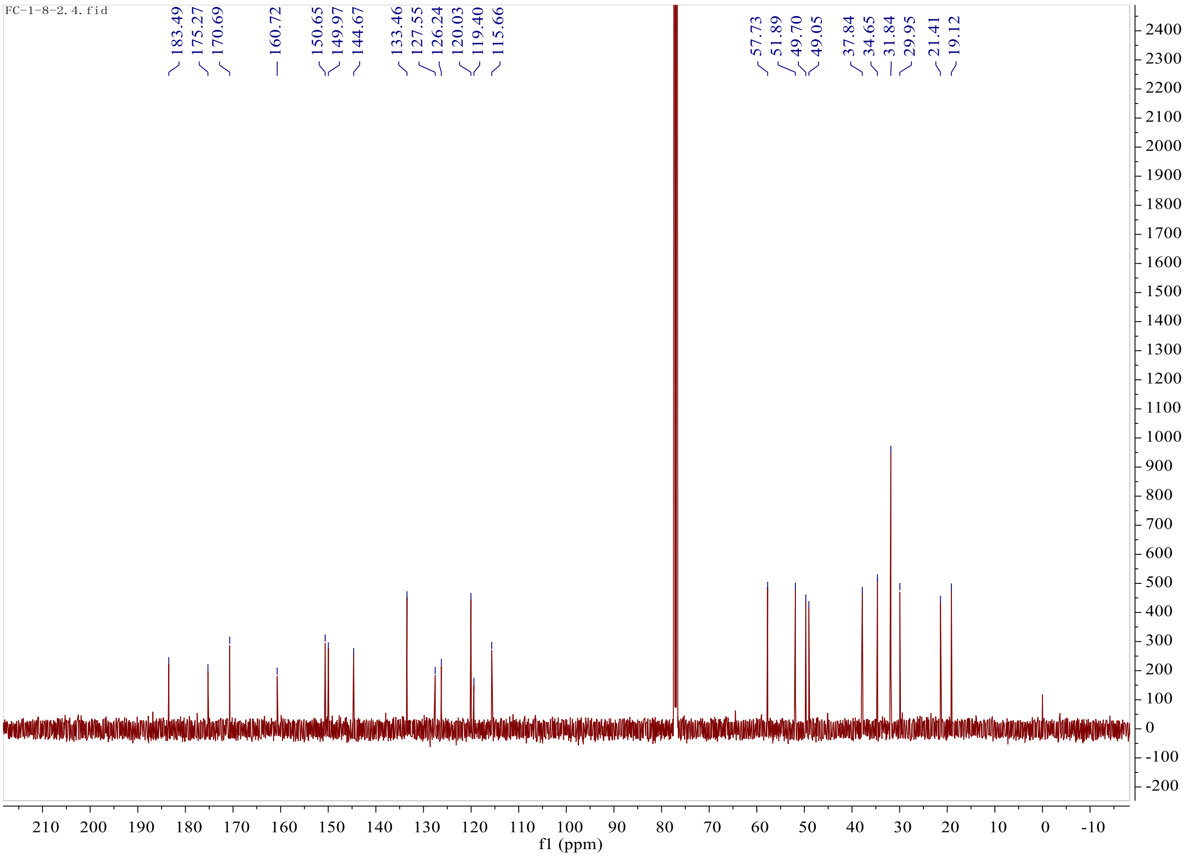
FC-1-8-2.4.fid
183.49
175.27
170.69
160.72
150.65
149.97
144.67
133.46
127.55
126.24
120.03
119.40
115.66
57.73
51.89
49.70
49.05
37.84
34.65
31.84
29.95
21.41
19.12
f1 (ppm)

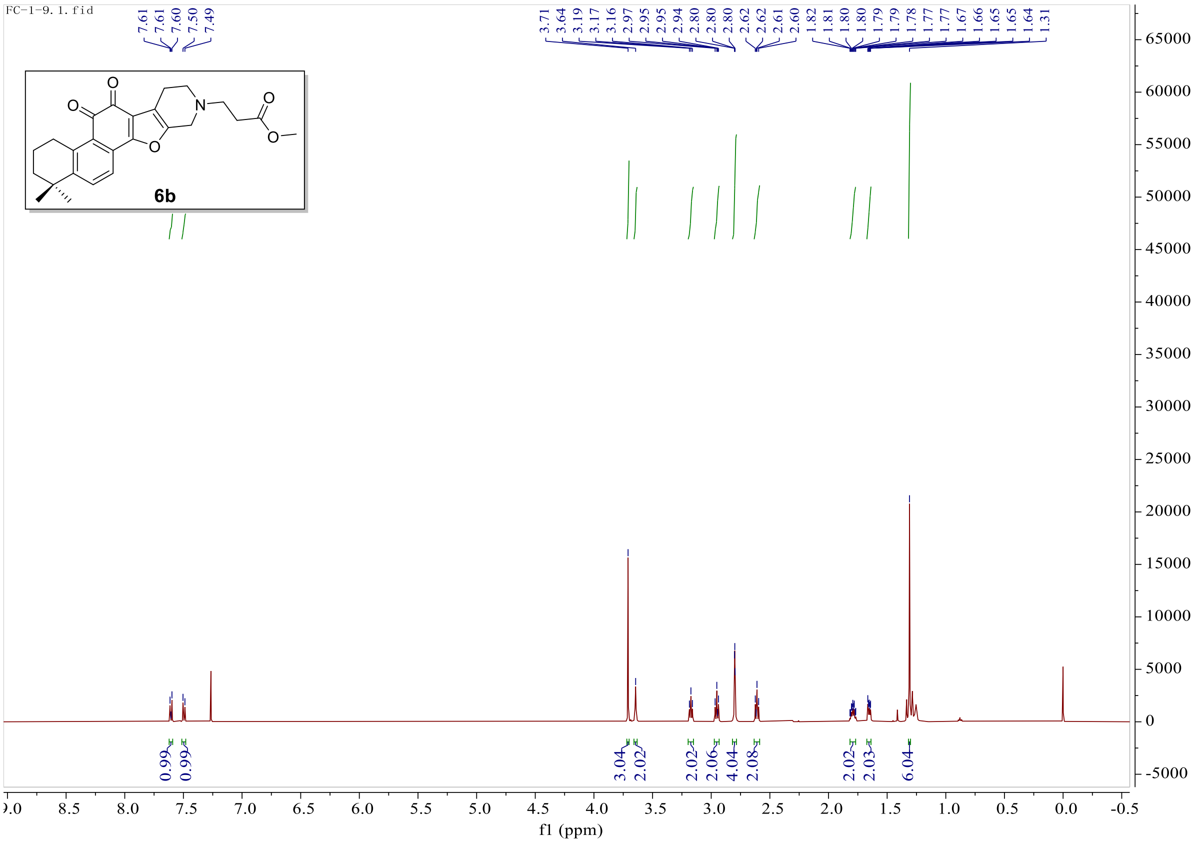
FC-1-9.1.fid
7.61 7.61 7.60 7.50 7.49
3.71 3.64 3.19 3.17 3.16 2.97 2.95 2.95 2.94 2.80 2.80 2.80 2.62 2.62 2.61 2.60 1.82 1.81 1.80 1.80 1.79 1.79 1.78 1.77 1.77 1.67 1.66 1.65 1.65 1.64 1.31
6b
0.99 0.99 3.04 2.02 2.02 2.06 4.04 2.08 2.02 2.03 6.04
f1 (ppm)

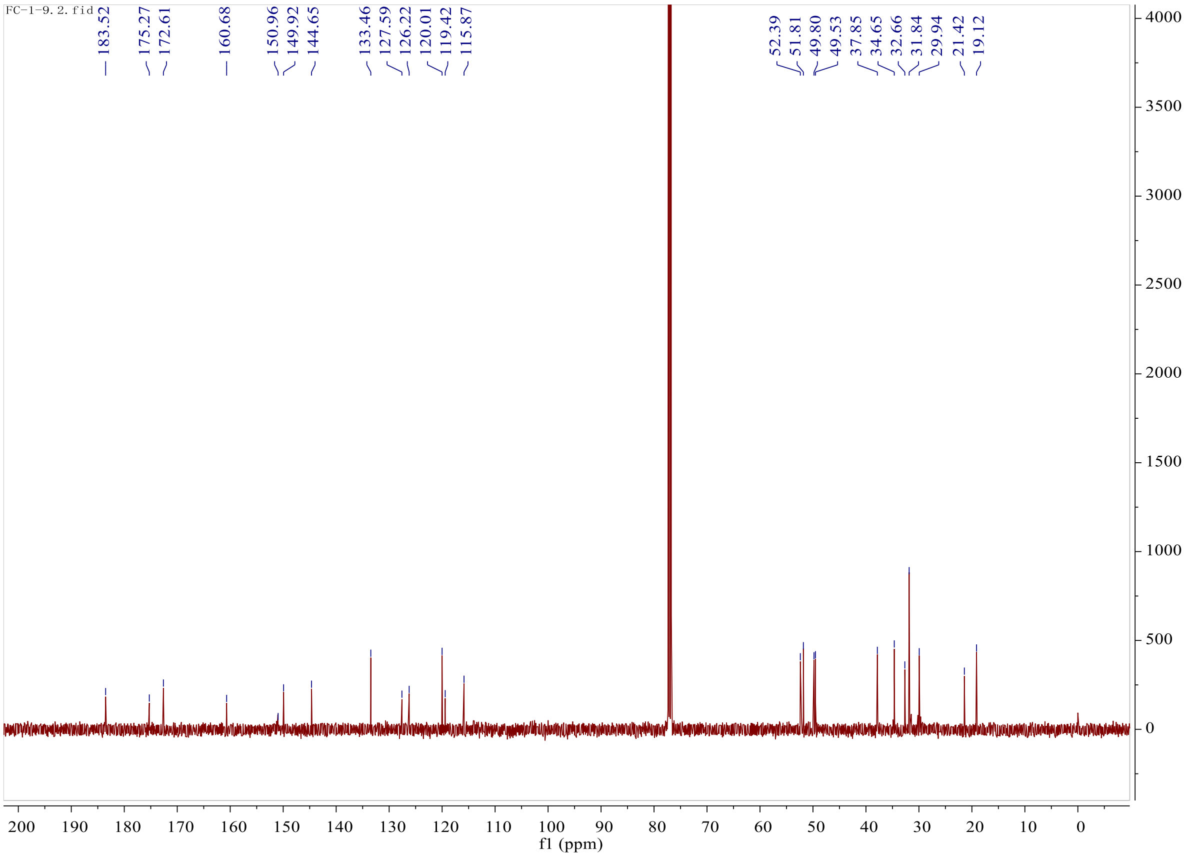
FC-1-9.2.fid
183.52 175.27 172.61 160.68 150.96 149.92 144.65 133.46 127.59 126.22 120.01 119.42 115.87
52.39 51.81 49.80 49.53 37.85 34.65 32.66 31.84 29.94 21.42 19.12
f1 (ppm)

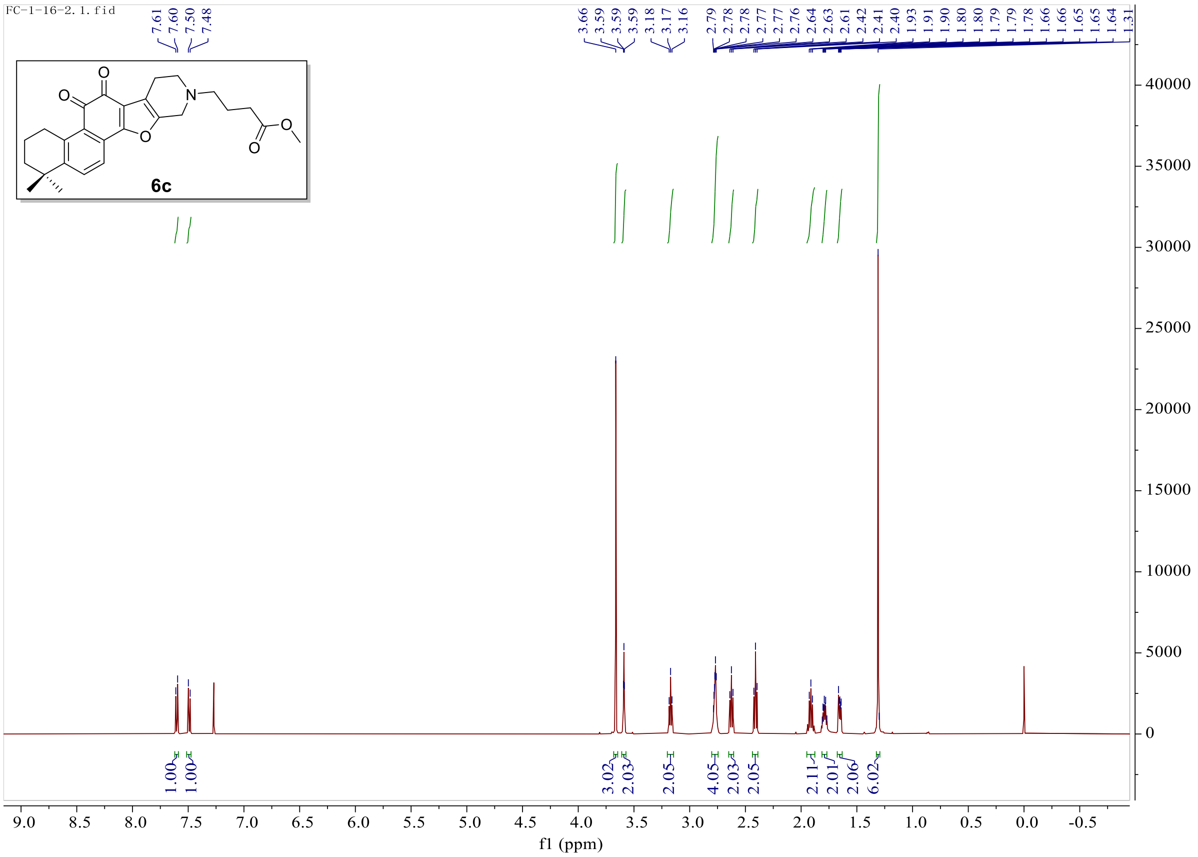
FC-1-16-2.1.fid
7.61 7.60 7.50 7.48
3.66 3.59 3.59 3.59 3.18 3.17 3.16
2.79 2.78 2.78 2.77 2.77 2.76 2.64 2.63 2.61 2.42 2.41 2.40 1.93 1.91 1.90 1.80 1.80 1.79 1.79 1.78 1.66 1.66 1.65 1.64 1.31
6c
1.00 1.00 3.02 2.03 2.05 4.05 2.03 2.05 2.11 2.01 2.06 6.02
f1 (ppm)

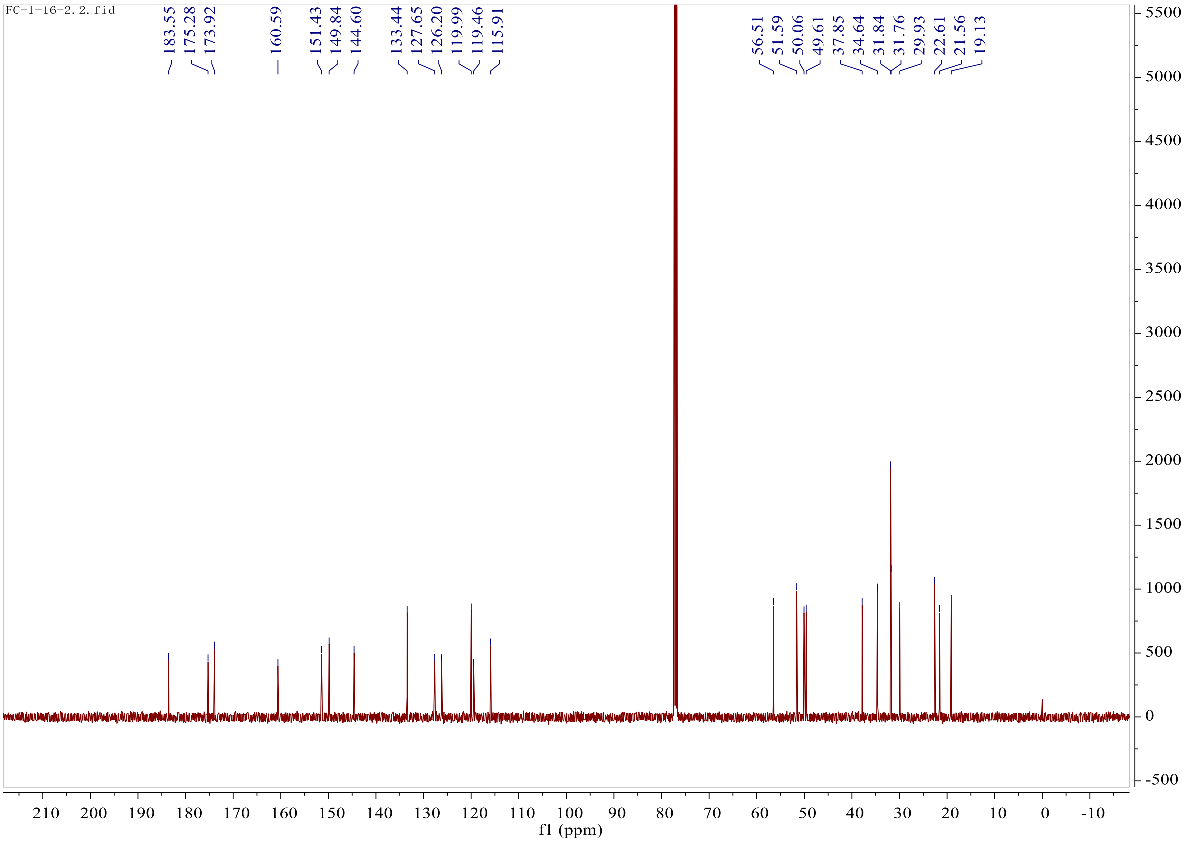
FC-1-16-2.2.fid
183.55 175.28 173.92 160.59 151.43 149.84 144.60 133.44 127.65 126.20 119.99 119.46 115.91
56.51 51.59 50.06 49.61 37.85 34.64 31.84 31.76 29.93 22.61 21.56 19.13
f1 (ppm)

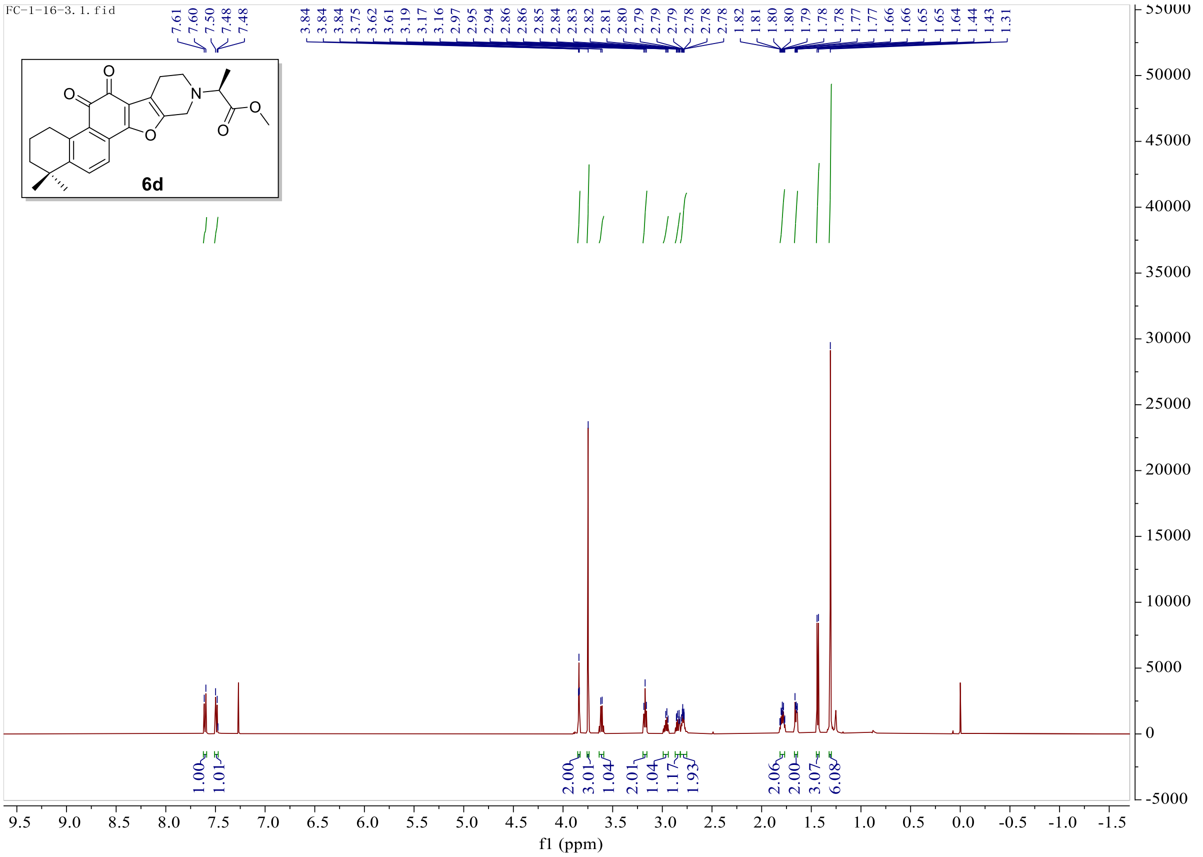
FC-1-16-3.1.fid
7.61
7.60
7.50
7.48
7.48
3.84
3.84
3.84
3.75
3.62
3.61
3.19
3.17
3.16
2.97
2.95
2.94
2.86
2.86
2.85
2.84
2.83
2.82
2.81
2.80
2.79
2.79
2.79
2.78
2.78
2.78
1.82
1.81
1.80
1.80
1.79
1.78
1.78
1.77
1.77
1.66
1.65
1.65
1.64
1.44
1.43
1.31
6d
1.00
1.01
2.00
3.01
1.04
2.01
1.04
1.17
1.93
2.06
2.00
3.07
6.08
f1 (ppm)

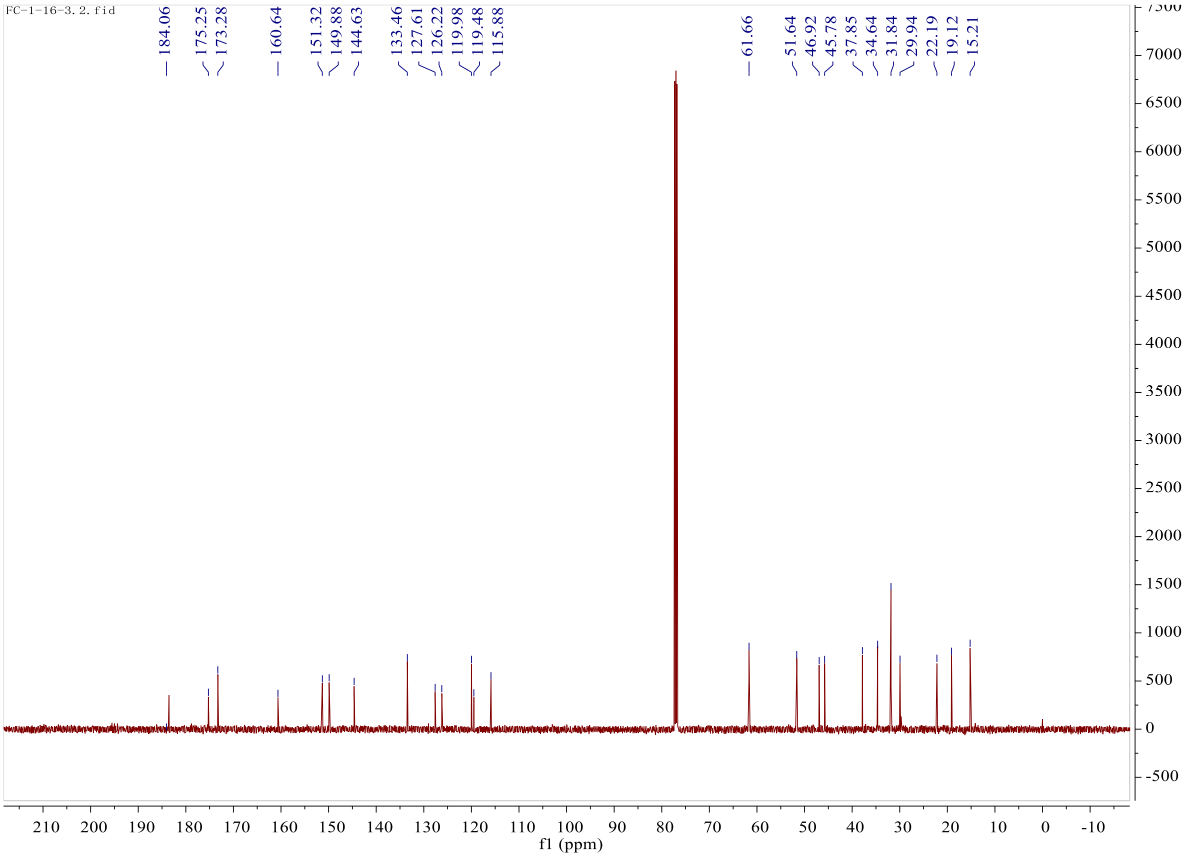
FC-1-16-3.2.fid
184.06
175.25
173.28
160.64
151.32
149.88
144.63
133.46
127.61
126.22
119.98
119.48
115.88
61.66
51.64
46.92
45.78
37.85
34.64
31.84
29.94
22.19
19.12
15.21
f1 (ppm)

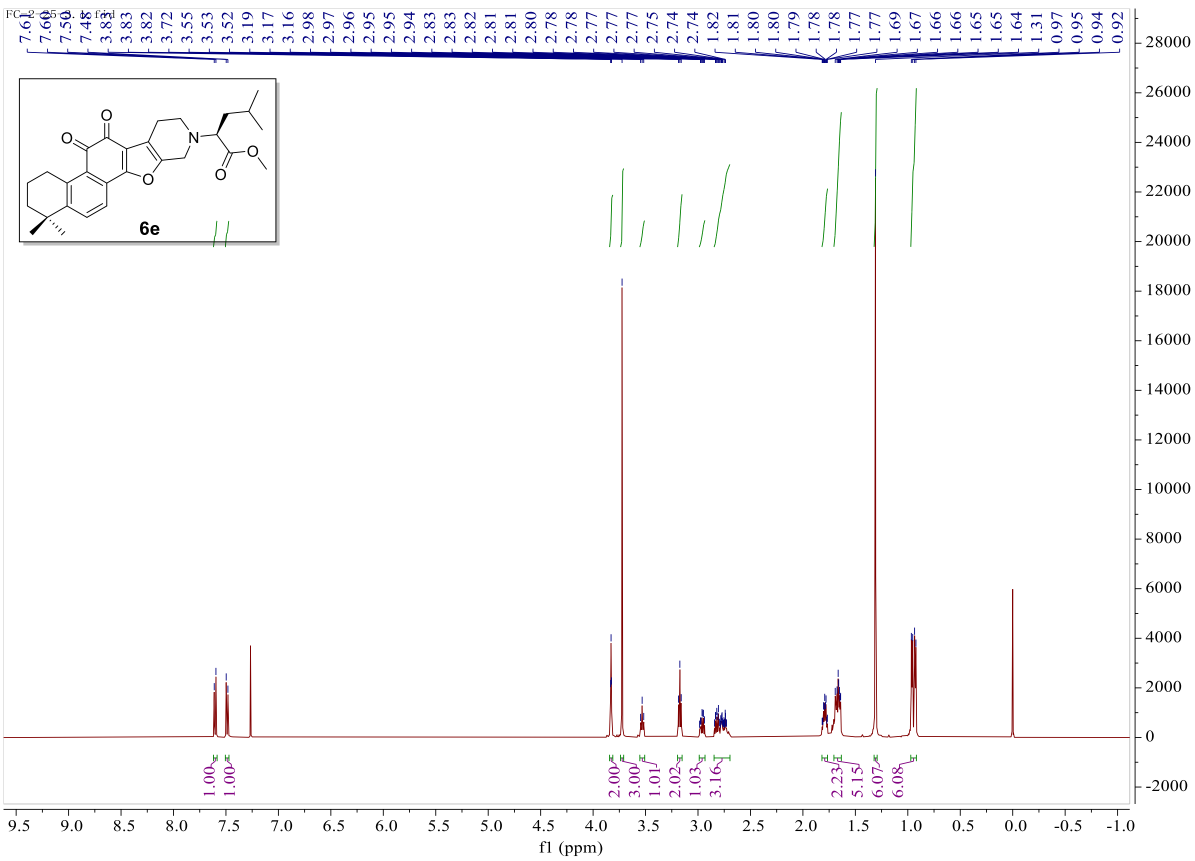
FC-2-25-3.1.fid
7.61
7.60
7.50
7.48
3.83
3.83
3.82
3.72
3.55
3.53
3.52
3.19
3.17
3.16
2.98
2.97
2.96
2.95
2.95
2.94
2.83
2.83
2.82
2.81
2.81
2.80
2.78
2.78
2.77
2.77
2.77
2.75
2.74
2.74
1.82
1.81
1.80
1.80
1.79
1.78
1.78
1.77
1.77
1.69
1.67
1.66
1.65
1.65
1.64
1.31
0.97
0.95
0.94
0.92
6e
1.00
1.00
2.00
3.00
1.01
2.02
1.03
3.16
2.23
5.15
6.07
6.08
f1 (ppm)

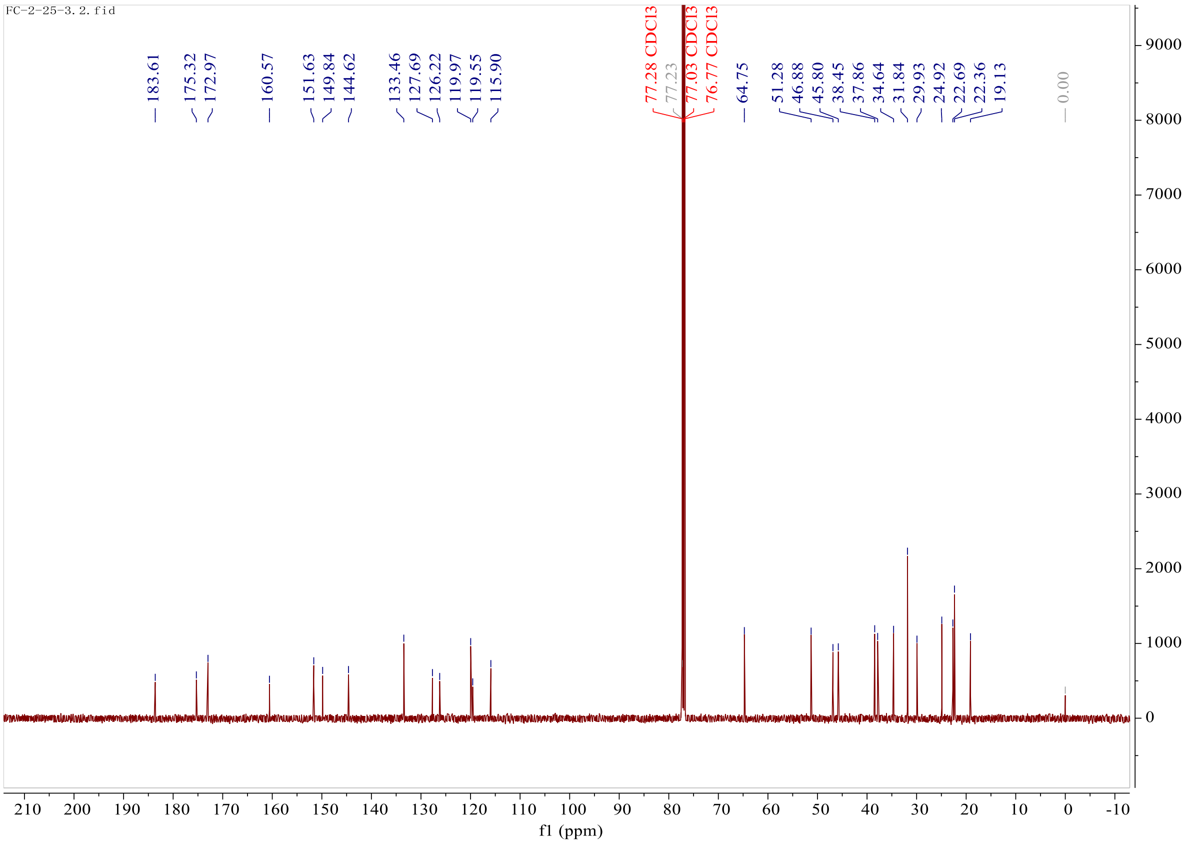
FC-2-25-3.2.fid
183.61
175.32
172.97
160.57
151.63
149.84
144.62
133.46
127.69
126.22
119.97
119.55
115.90
77.28 CDCl3
77.23
77.03 CDCl3
76.77 CDCl3
64.75
51.28
46.88
45.80
38.45
37.86
34.64
31.84
29.93
24.92
22.69
22.36
19.13
0.00
f1 (ppm)

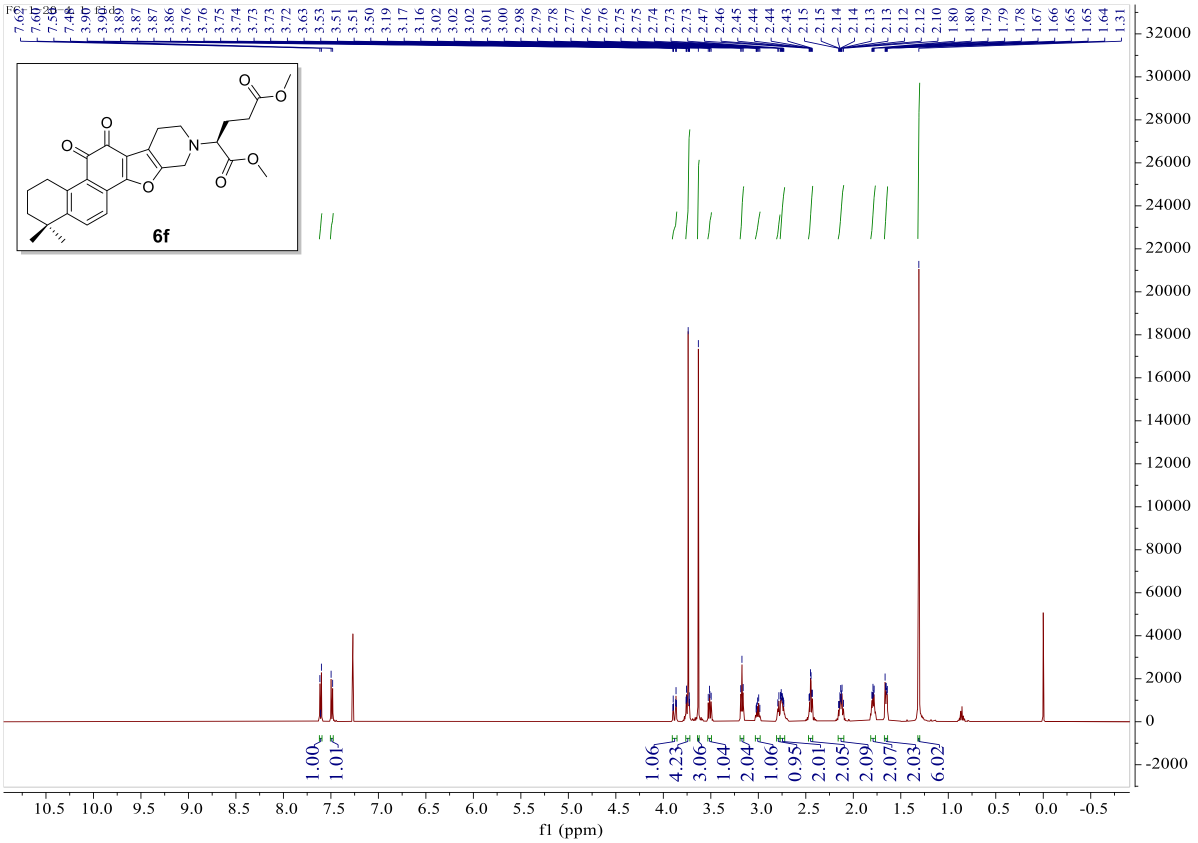

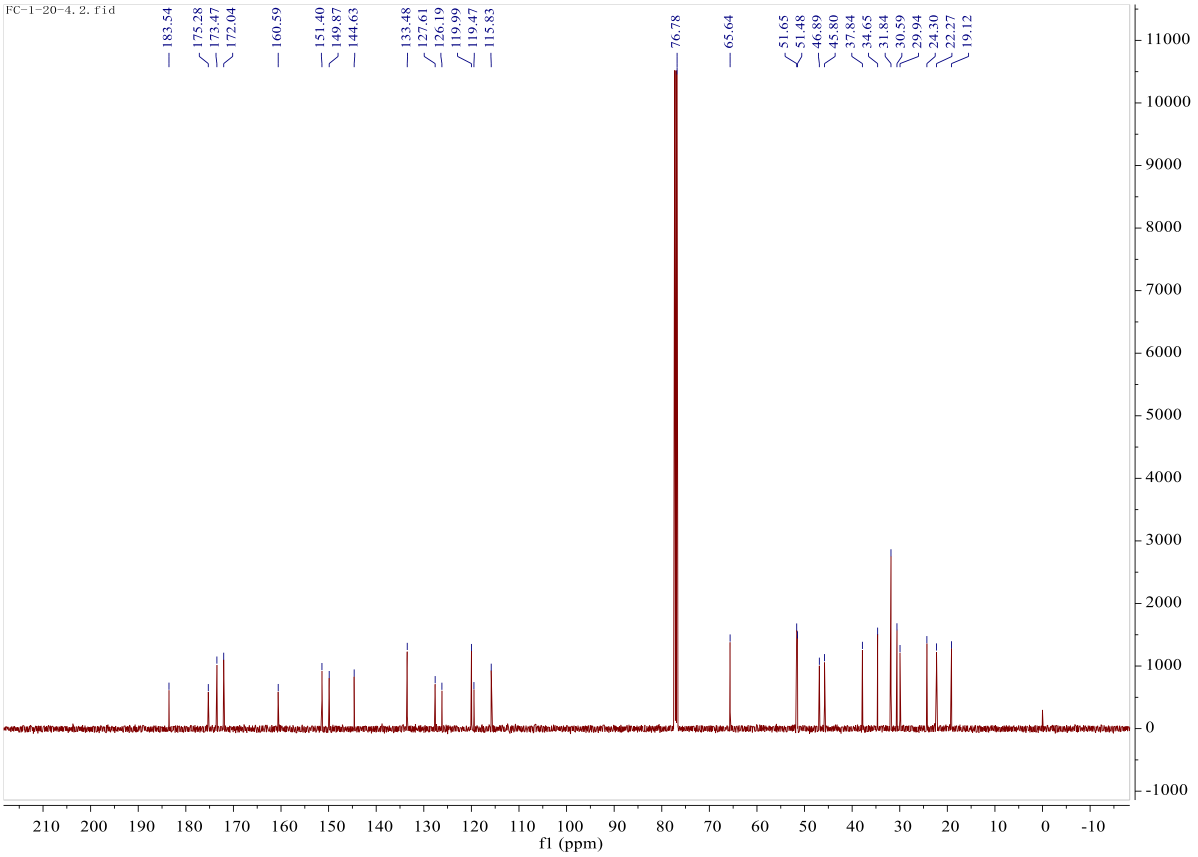

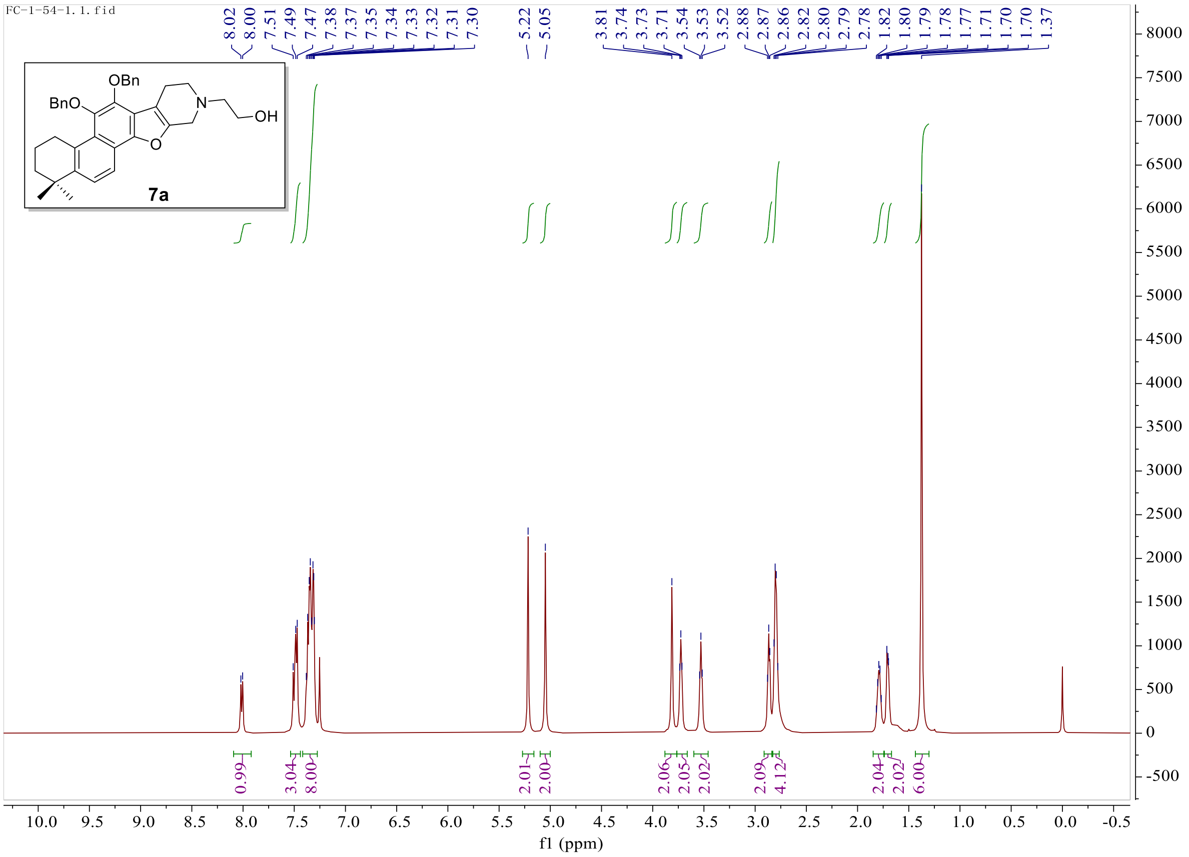
FC-1-54-1.1.fid
BnO
OBn
N
OH
O
7a
8.02 8.00 7.51 7.49 7.47 7.38 7.37 7.35 7.34 7.33 7.32 7.31 7.30 5.22 5.05 3.81 3.74 3.73 3.71 3.54 3.53 3.52 2.88 2.87 2.86 2.82 2.80 2.79 2.78 1.82 1.80 1.79 1.78 1.77 1.71 1.70 1.70 1.37
0.99 3.04 8.00 2.01 2.00 2.06 2.05 2.02 2.09 4.12 2.04 2.02 6.00
f1 (ppm)

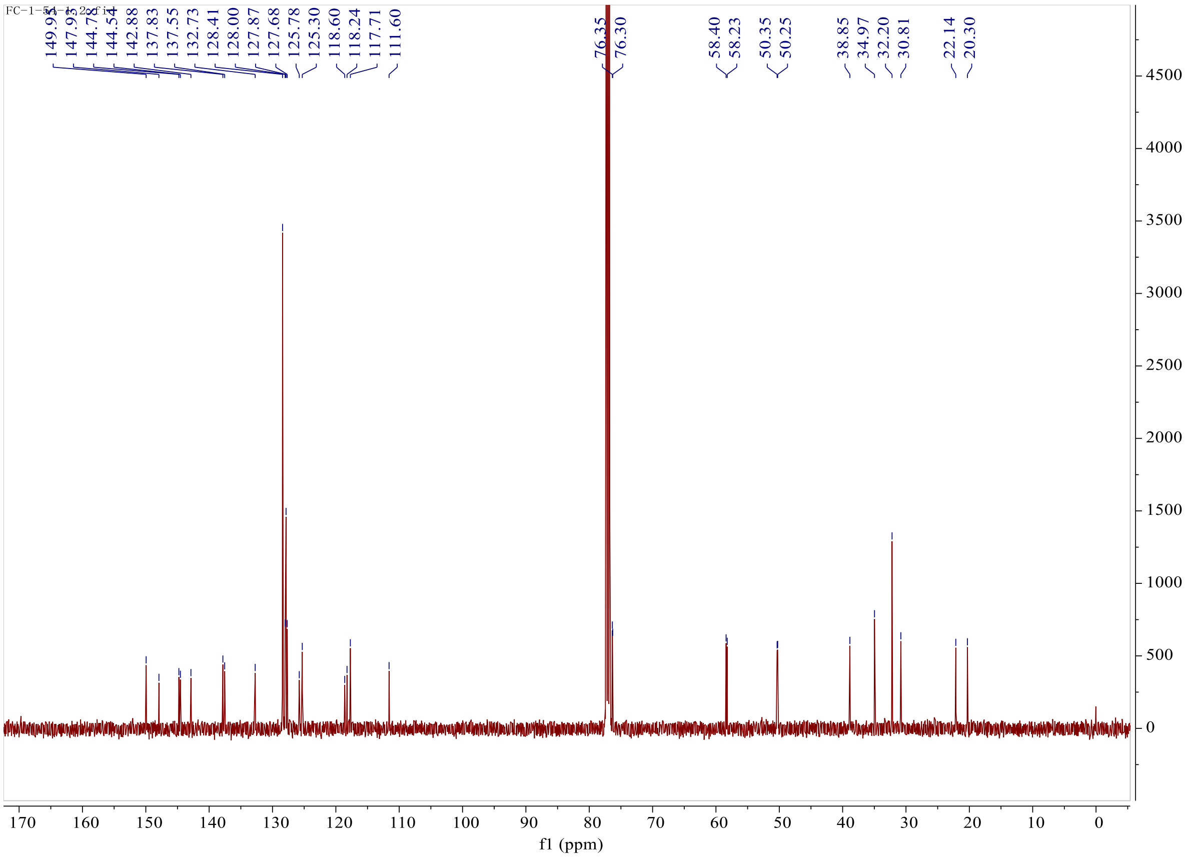
149.93 147.93 144.78 144.54 142.88 137.83 137.55 132.73 128.41 128.00 127.87 127.68 125.78 125.30 118.60 118.24 117.71 111.60 76.35 76.30 58.40 58.23 50.35 50.25 38.85 34.97 32.20 30.81 22.14 20.30
f1 (ppm)

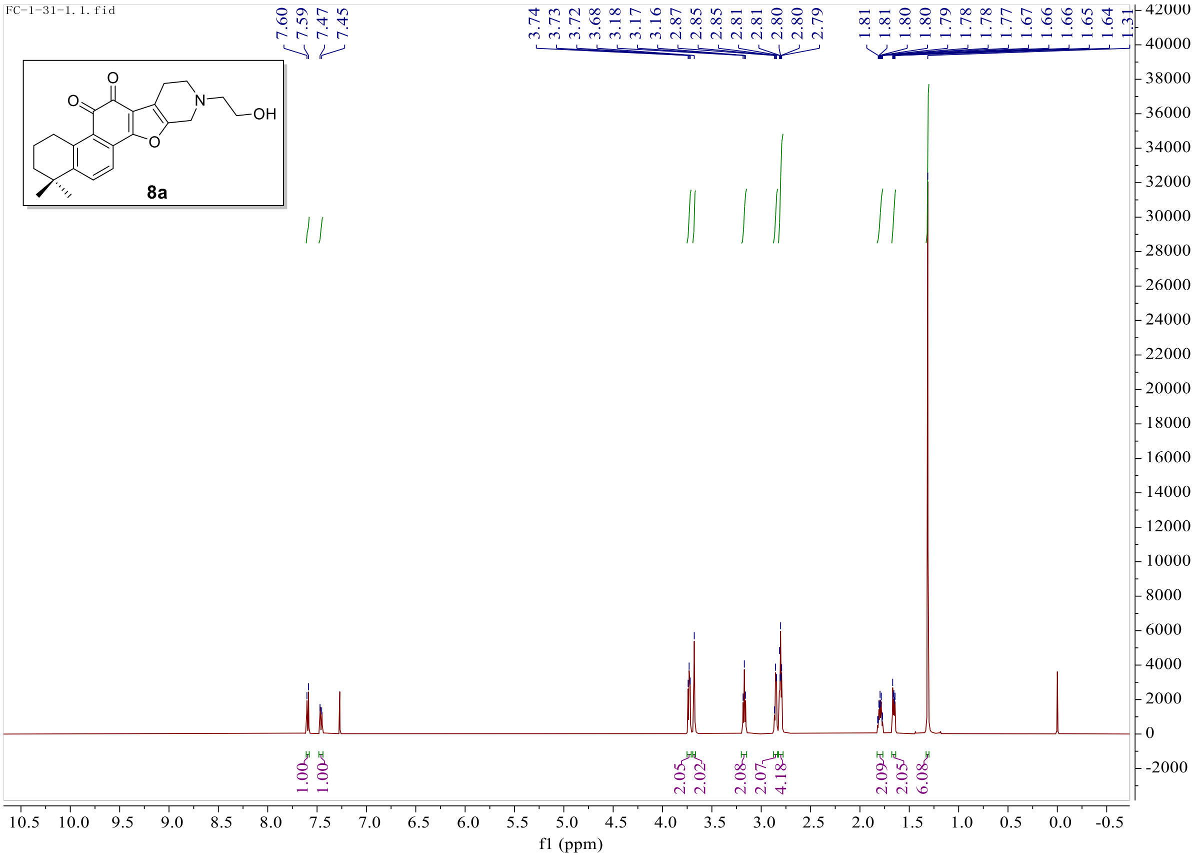
FC-1-31-1.1.fid
7.60 7.59 7.47 7.45
3.74 3.73 3.72 3.68 3.18 3.17 3.16 2.87 2.85 2.85 2.81 2.81 2.80 2.80 2.79
1.81 1.81 1.80 1.80 1.79 1.78 1.78 1.77 1.67 1.66 1.66 1.65 1.64 1.31
8a
1.00 1.00 2.05 2.02 2.08 2.07 4.18 2.09 2.05 6.08
f1 (ppm)

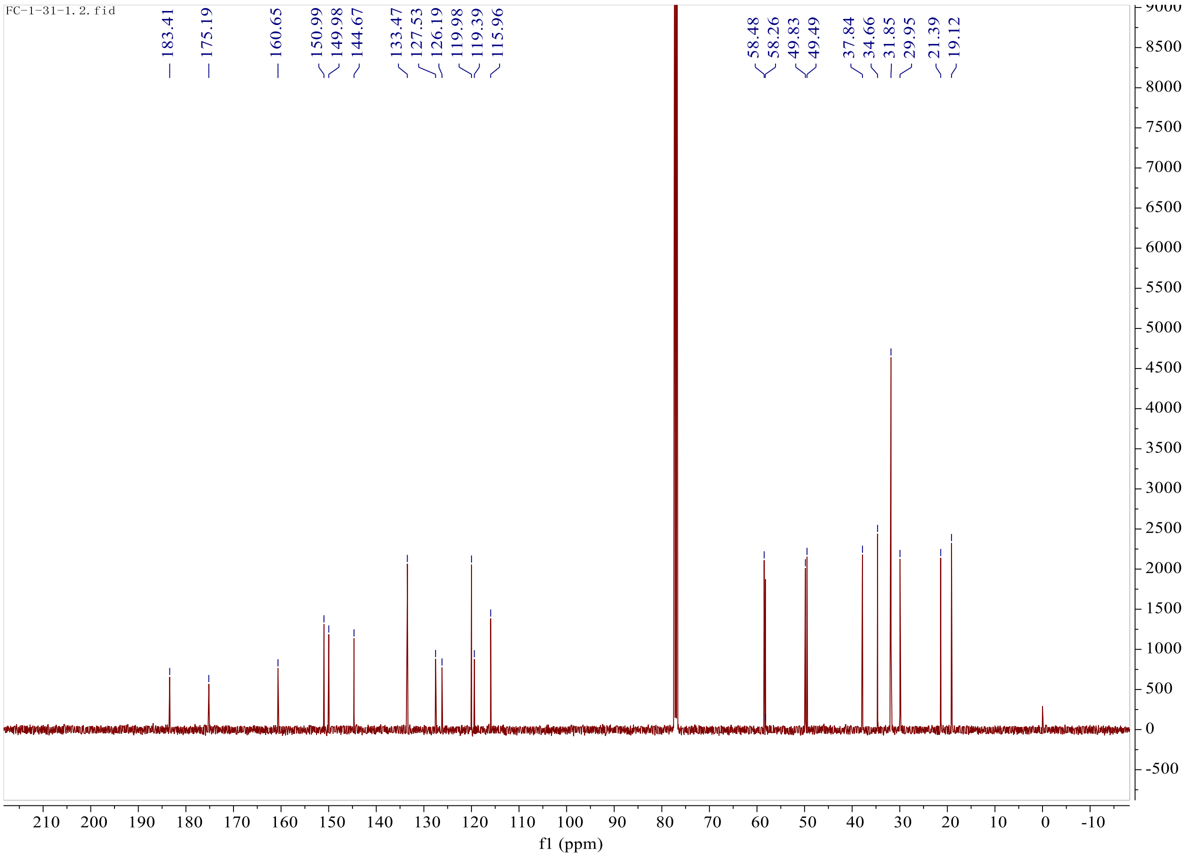
FC-1-31-1.2.fid
183.41 175.19 160.65 150.99 149.98 144.67 133.47 127.53 126.19 119.98 119.39 115.96
58.48 58.26 49.83 49.49 37.84 34.66 31.85 29.95 21.39 19.12
f1 (ppm)

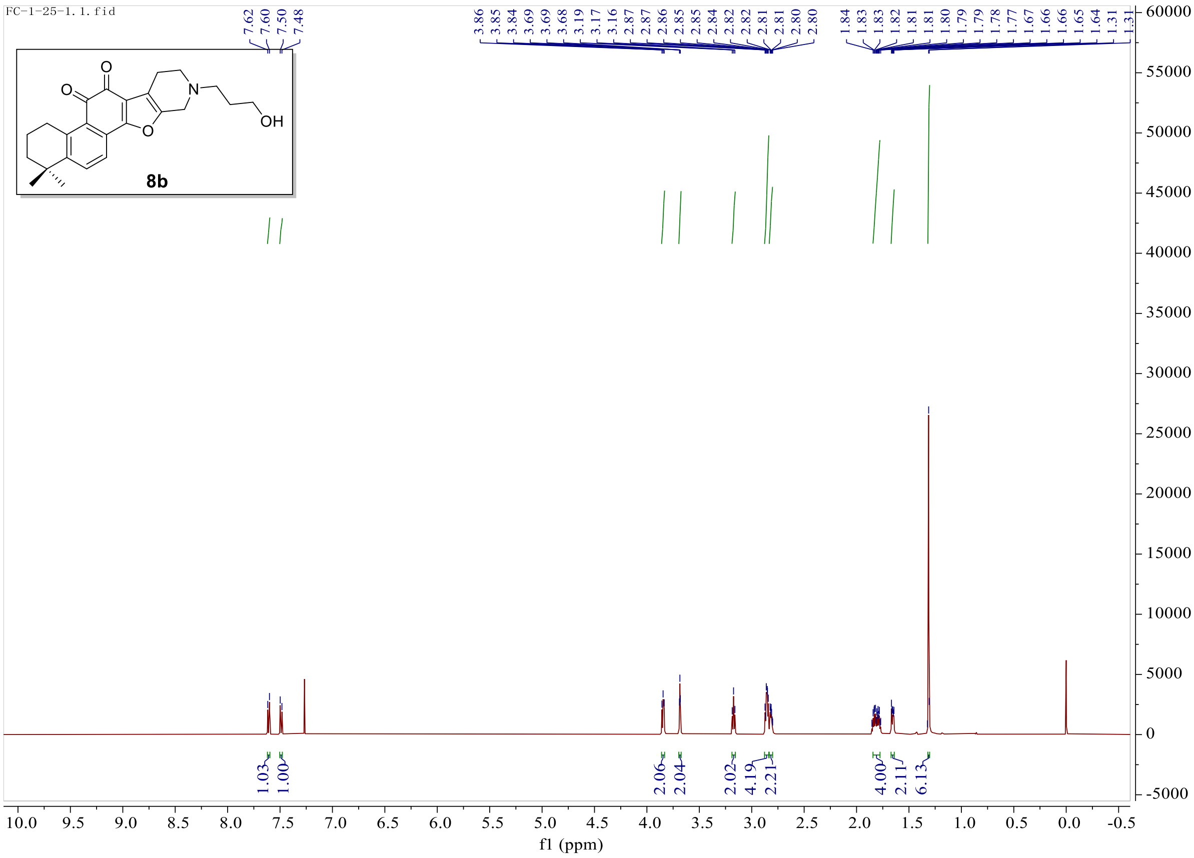
FC-1-25-1.1.fid
7.62
7.60
7.50
7.48
3.86
3.85
3.84
3.69
3.69
3.68
3.19
3.17
3.16
2.87
2.87
2.86
2.85
2.85
2.84
2.82
2.82
2.81
2.81
2.81
2.80
2.80
1.84
1.83
1.83
1.82
1.81
1.81
1.80
1.79
1.79
1.78
1.77
1.67
1.66
1.66
1.65
1.64
1.31
1.31
O
O
N
OH
O
8b
1.03
1.00
2.06
2.04
2.02
4.19
2.21
4.00
2.11
6.13
f1 (ppm)

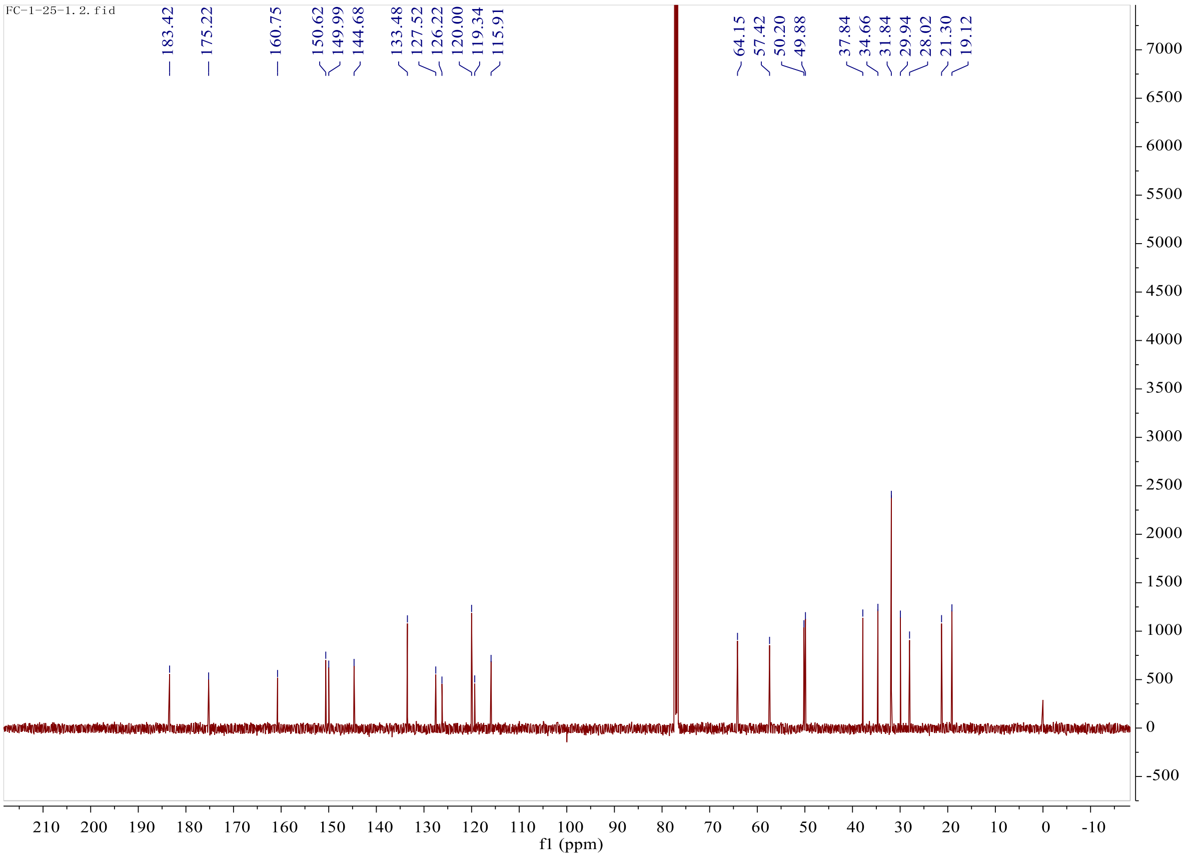
FC-1-25-1.2.fid
183.42
175.22
160.75
150.62
149.99
144.68
133.48
127.52
126.22
120.00
119.34
115.91
64.15
57.42
50.20
49.88
37.84
34.66
31.84
29.94
28.02
21.30
19.12
f1 (ppm)

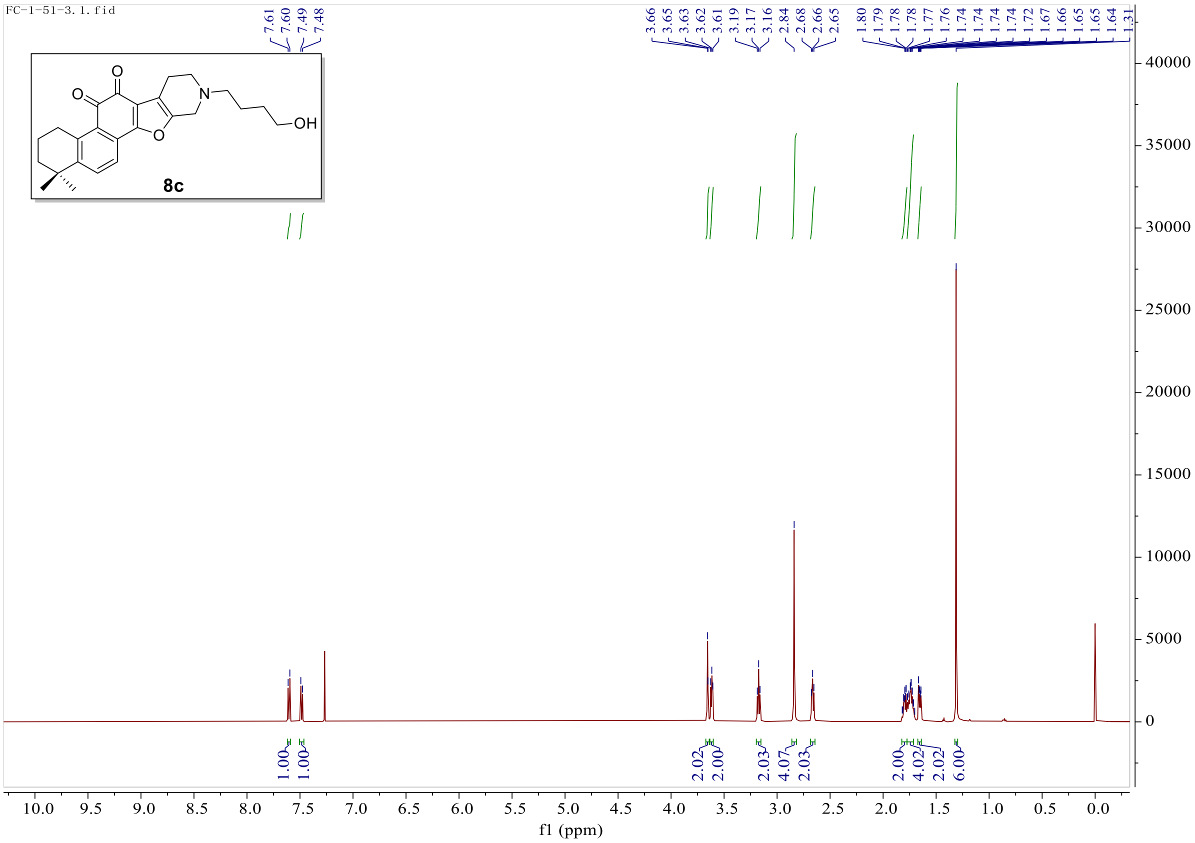
FC-1-51-3.1.fid
7.61
7.60
7.49
7.48
3.66
3.65
3.63
3.62
3.61
3.19
3.17
3.16
2.84
2.68
2.66
2.65
1.80
1.79
1.78
1.78
1.77
1.76
1.74
1.74
1.74
1.74
1.72
1.67
1.66
1.65
1.65
1.64
1.31
O
O
N
O
OH
8c
1.00
1.00
2.02
2.00
2.03
4.07
2.03
2.00
4.02
2.02
6.00
40000
35000
30000
25000
20000
15000
10000
5000
0
10.0
9.5
9.0
8.5
8.0
7.5
7.0
6.5
6.0
5.5
5.0
4.5
4.0
3.5
3.0
2.5
2.0
1.5
1.0
0.5
0.0
f1 (ppm)

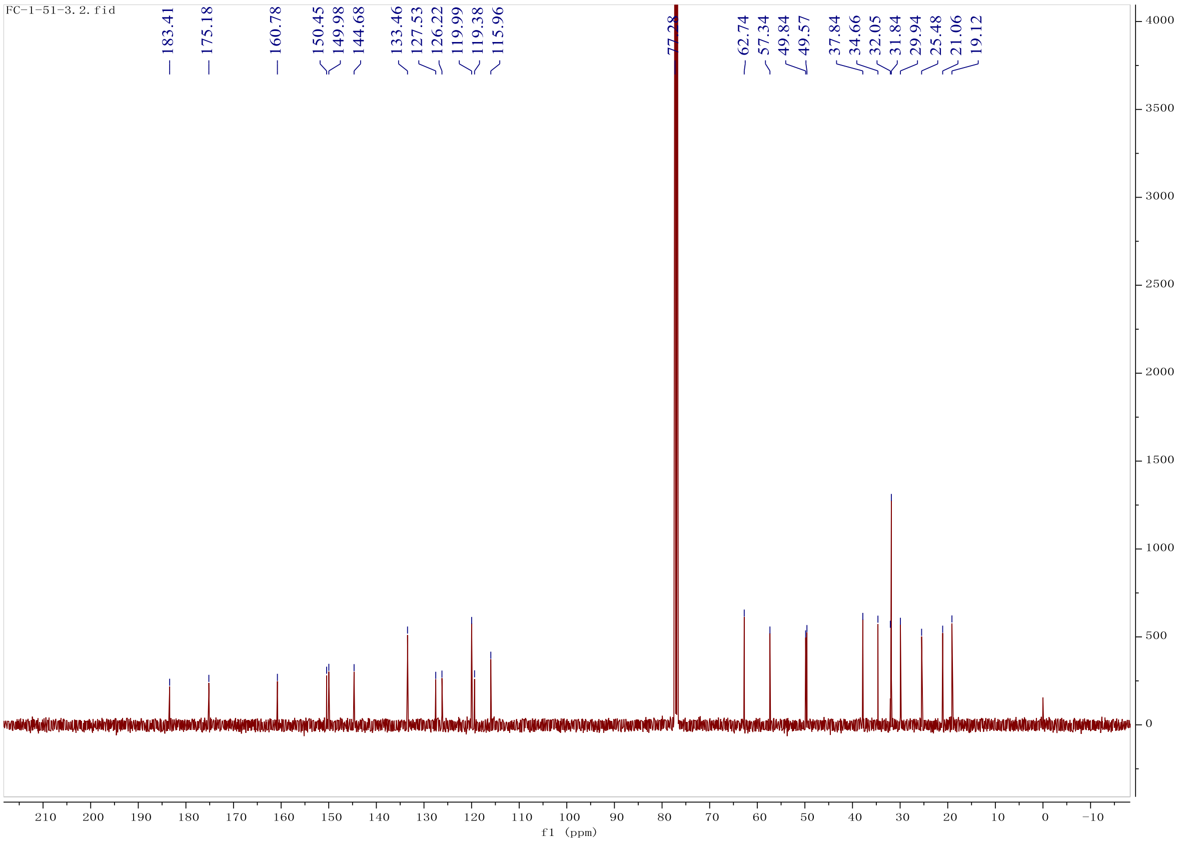
FC-1-51-3.2.fid
183.41
175.18
160.78
150.45
149.98
144.68
133.46
127.53
126.22
119.99
119.38
115.96
77.28
62.74
57.34
49.84
49.57
37.84
34.66
32.05
31.84
29.94
25.48
21.06
19.12
4000
3500
3000
2500
2000
1500
1000
500
0
210
200
190
180
170
160
150
140
130
120
110
100
90
80
70
60
50
40
30
20
10
0
-10
f1 (ppm)

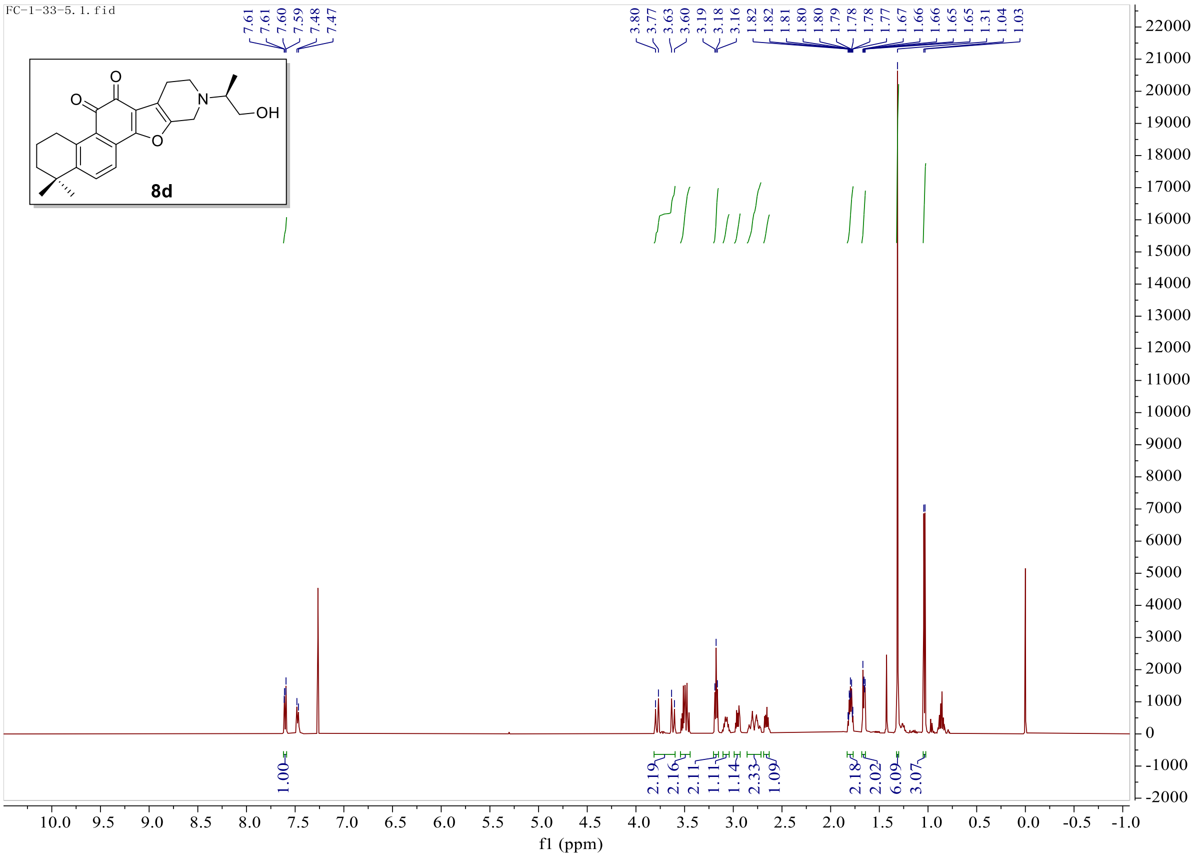
FC-1-33-5.1.fid
8d
7.61 7.61 7.60 7.59 7.48 7.47
3.80 3.77 3.63 3.60 3.19 3.18 3.16 1.82 1.82 1.81 1.80 1.80 1.79 1.78 1.78 1.77 1.67 1.66 1.66 1.65 1.65 1.31 1.04 1.03
1.00 2.19 2.16 2.11 1.11 1.14 2.33 1.09 2.18 2.02 6.09 3.07
f1 (ppm)

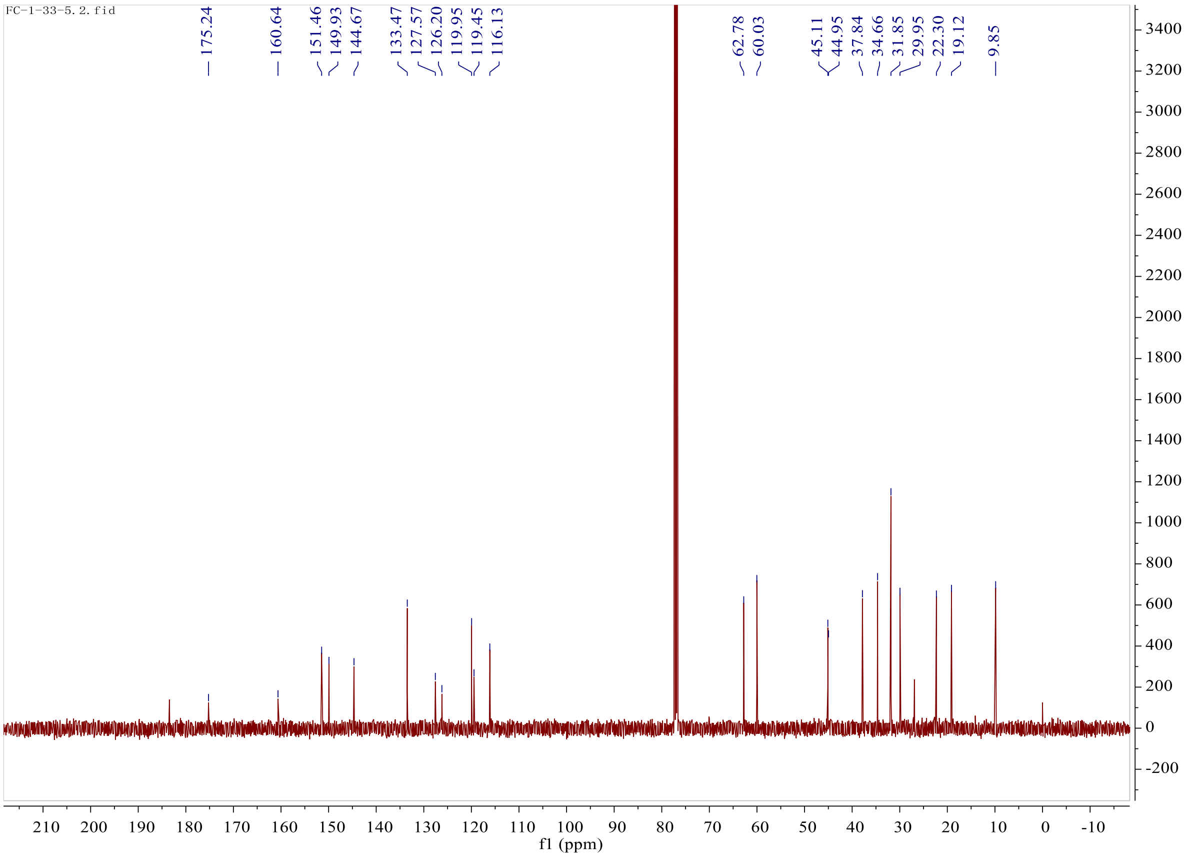
FC-1-33-5.2.fid
175.24 160.64 151.46 149.93 144.67 133.47 127.57 126.20 119.95 119.45 116.13 62.78 60.03 45.11 44.95 37.84 34.66 31.85 29.95 22.30 19.12 9.85
f1 (ppm)

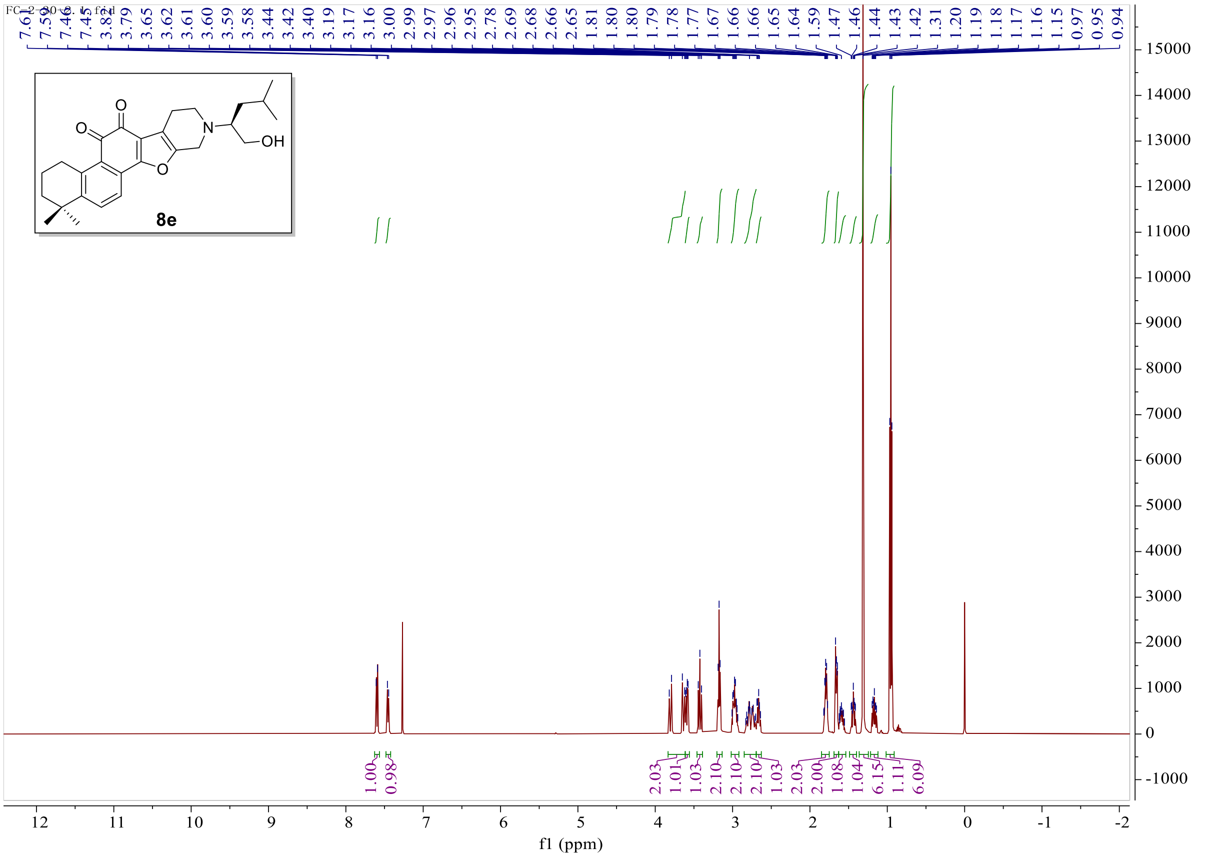
8e
O
O
N
OH
f1 (ppm)

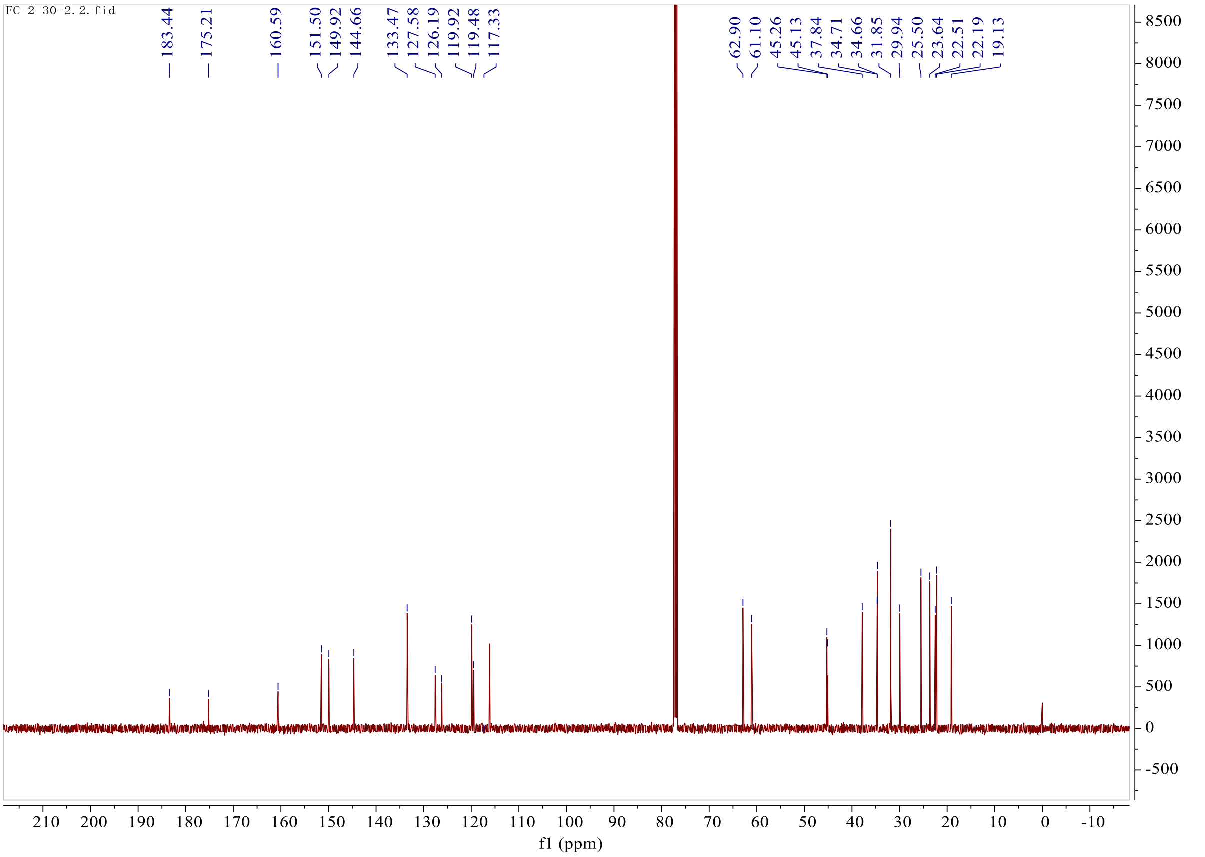
FC-2-30-2.2.fid
183.44
175.21
160.59
151.50
149.92
144.66
133.47
127.58
126.19
119.92
119.48
117.33
62.90
61.10
45.26
45.13
37.84
34.71
34.66
31.85
29.94
25.50
23.64
22.51
22.19
19.13
f1 (ppm)

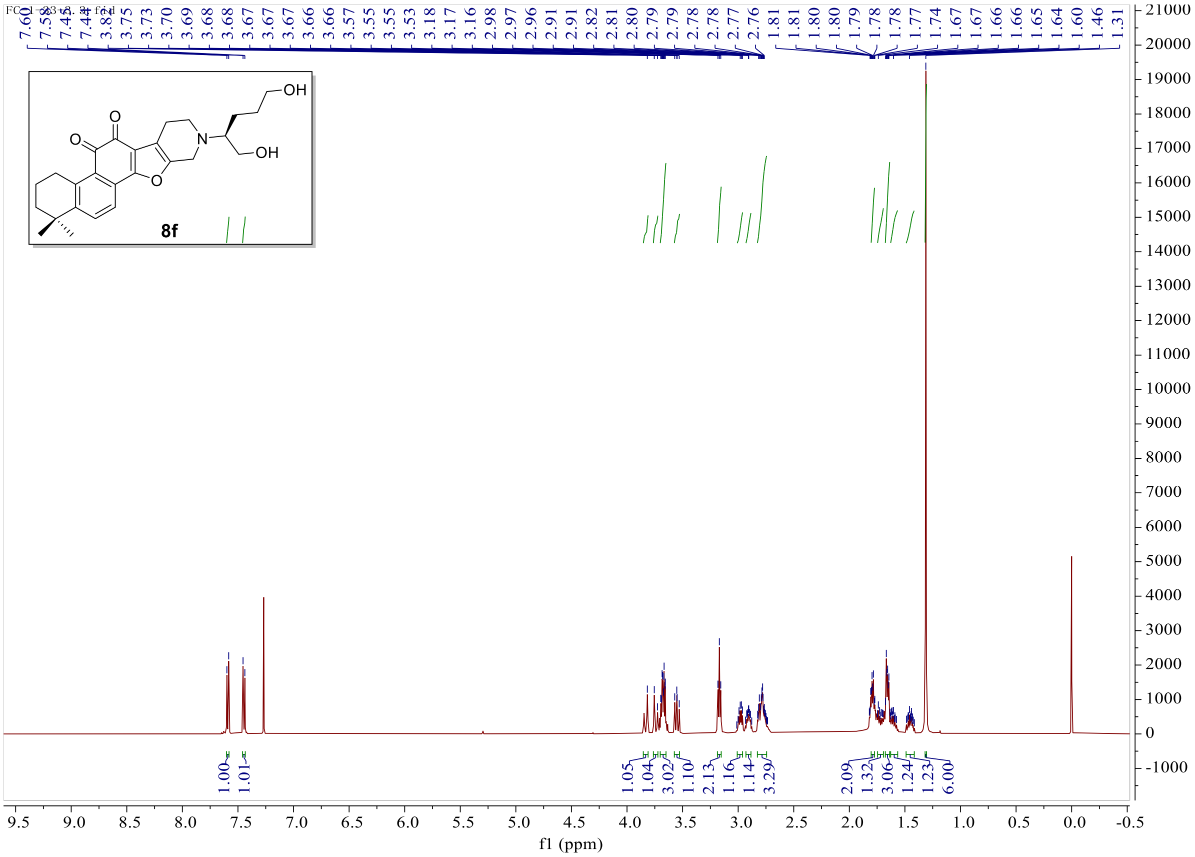
FC-1-33-3.1 [illegible]
7.60 7.58 7.45 7.44 3.82 3.75 3.73 3.70 3.69 3.68 3.68 3.67 3.67 3.67 3.66 3.66 3.57 3.55 3.55 3.53 3.18 3.17 3.16 2.98 2.97 2.96 2.91 2.91 2.82 2.81 2.80 2.79 2.79 2.78 2.78 2.77 2.76 1.81 1.81 1.80 1.80 1.79 1.78 1.78 1.77 1.74 1.67 1.67 1.66 1.66 1.65 1.64 1.60 1.46 1.31
OH
O
O
N
OH
O
8f
1.00 1.01 1.05 1.04 3.02 1.10 2.13 1.16 1.14 3.29 2.09 1.32 3.06 1.24 1.23 6.00
f1 (ppm)

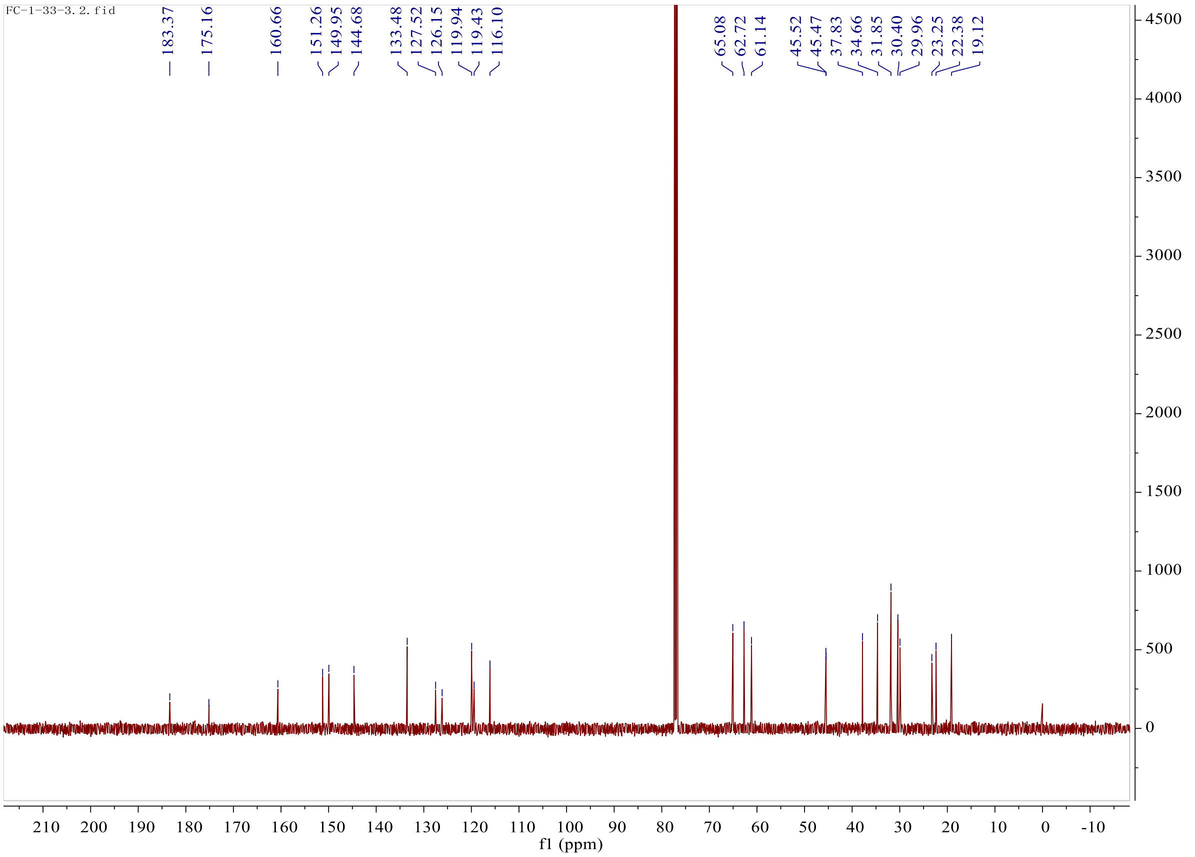
FC-1-33-3.2.fid
183.37 175.16 160.66 151.26 149.95 144.68 133.48 127.52 126.15 119.94 119.43 116.10 65.08 62.72 61.14 45.52 45.47 37.83 34.66 31.85 30.40 29.96 23.25 22.38 19.12
f1 (ppm)

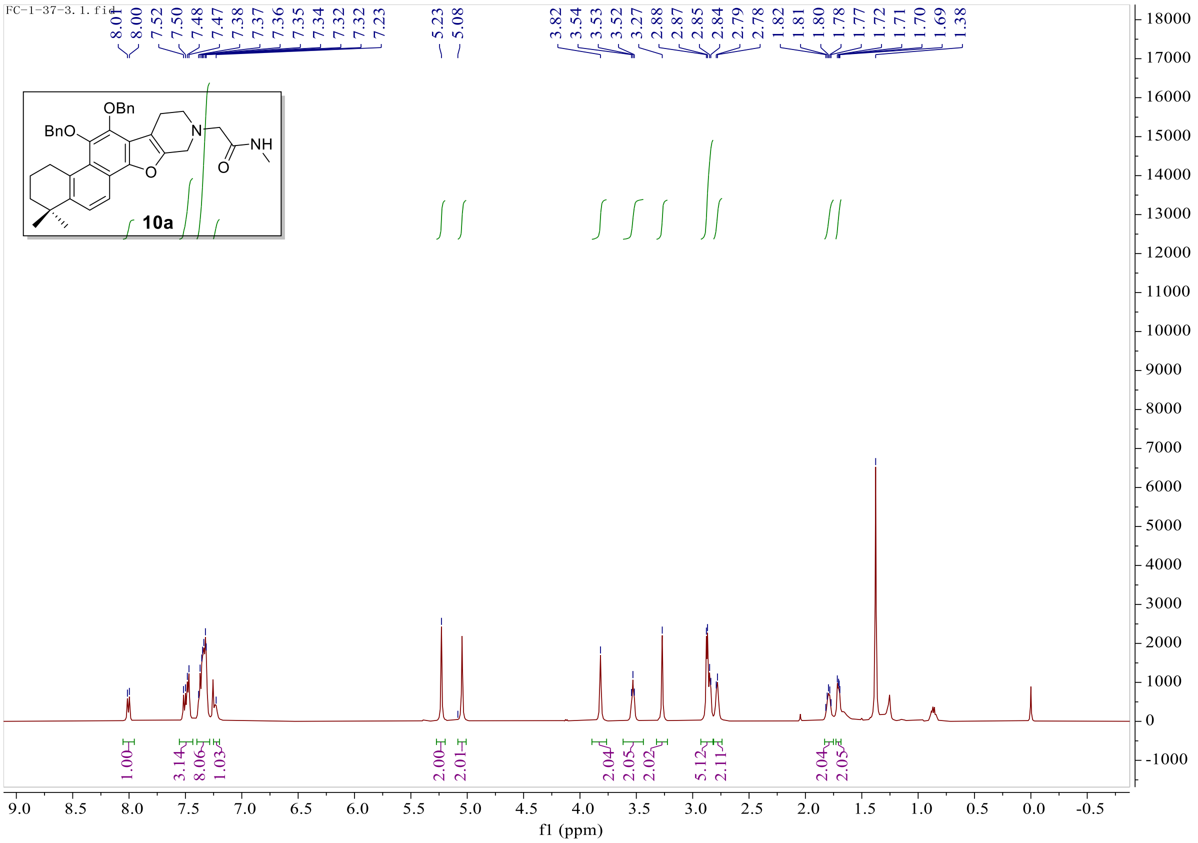
FC-1-37-3.1.fid
8.01
8.00
7.52
7.50
7.48
7.47
7.38
7.37
7.36
7.35
7.34
7.32
7.32
7.23
5.23
5.08
3.82
3.54
3.53
3.52
3.27
2.88
2.87
2.85
2.84
2.79
2.78
1.82
1.81
1.80
1.78
1.77
1.72
1.71
1.70
1.69
1.38
OBn
BnO
N
NH
O
O
10a
1.00
3.14
8.06
1.03
2.00
2.01
2.04
2.05
2.02
5.12
2.11
2.04
2.05
f1 (ppm)

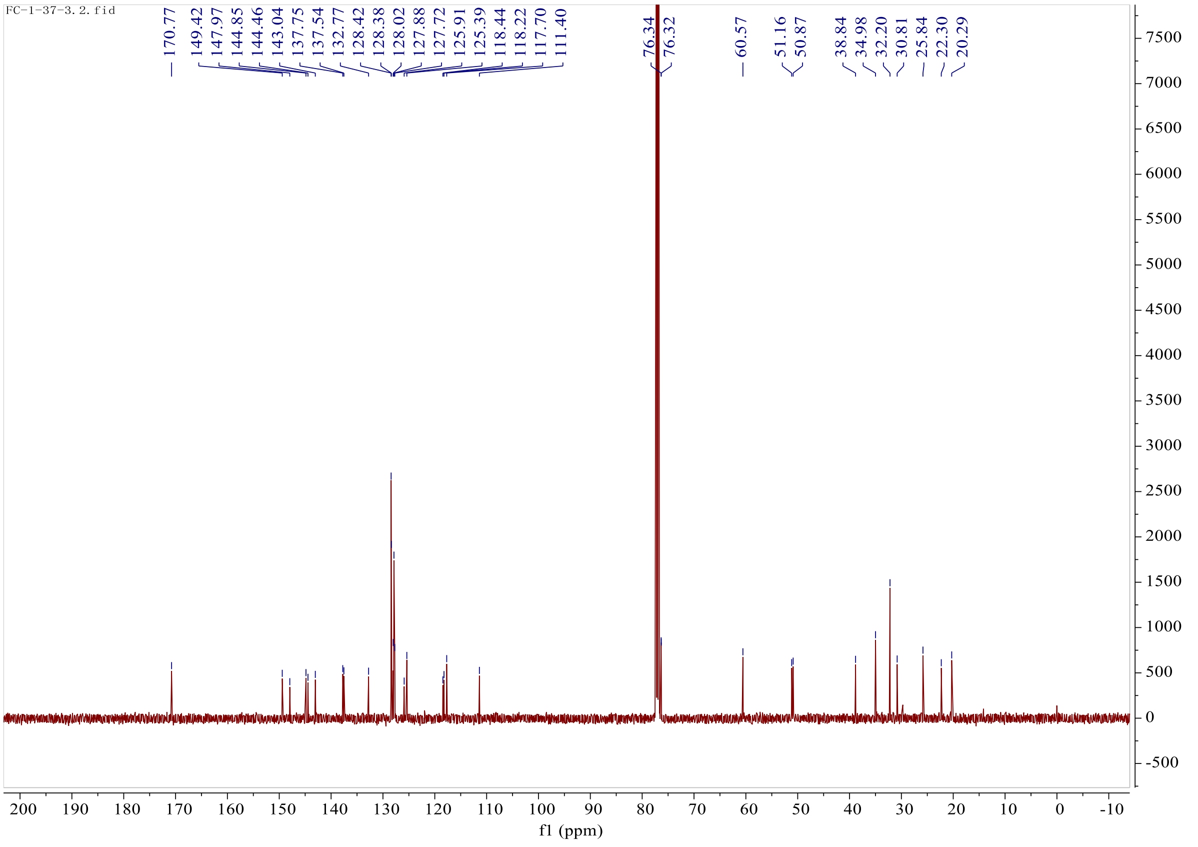
FC-1-37-3.2.fid
170.77
149.42
147.97
144.85
144.46
143.04
137.75
137.54
132.77
128.42
128.38
128.02
127.88
127.72
125.91
125.39
118.44
118.22
117.70
111.40
76.34
76.32
60.57
51.16
50.87
38.84
34.98
32.20
30.81
25.84
22.30
20.29
f1 (ppm)

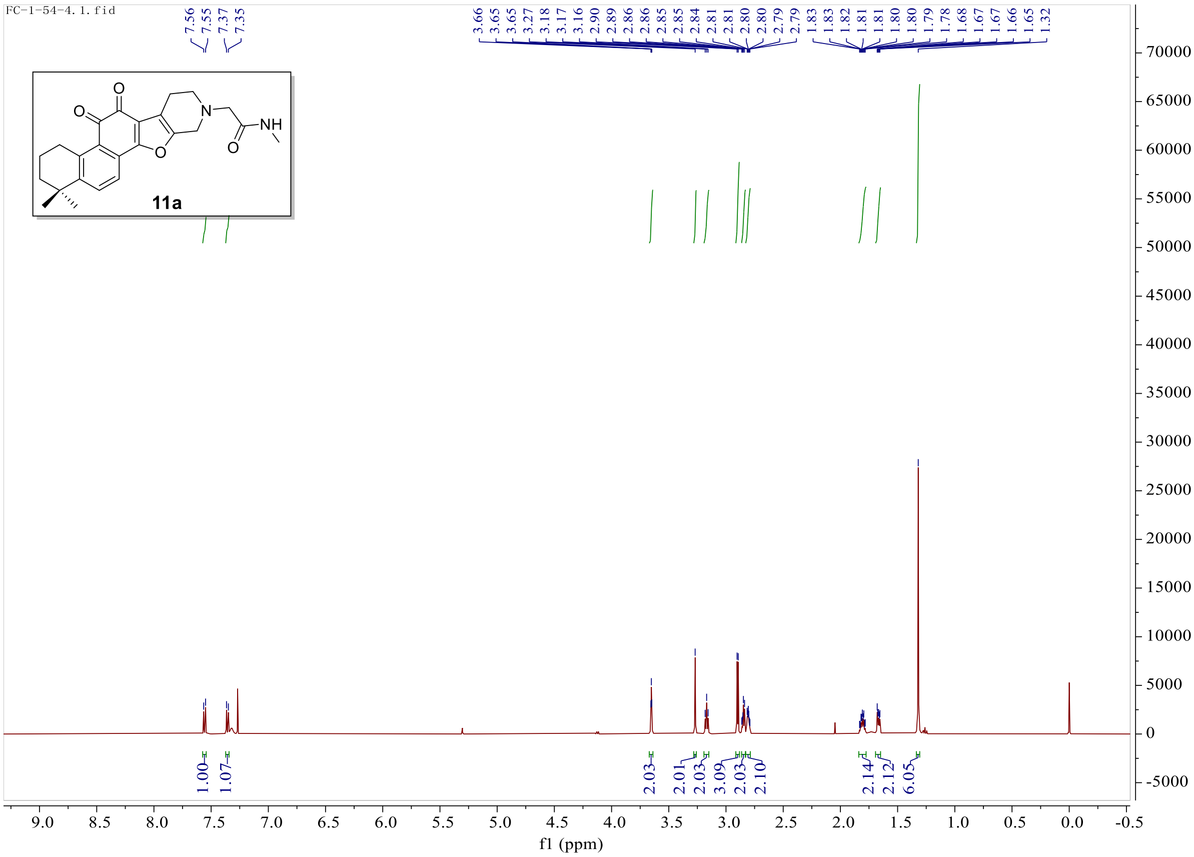
FC-1-54-4.1.fid
11a
f1 (ppm)

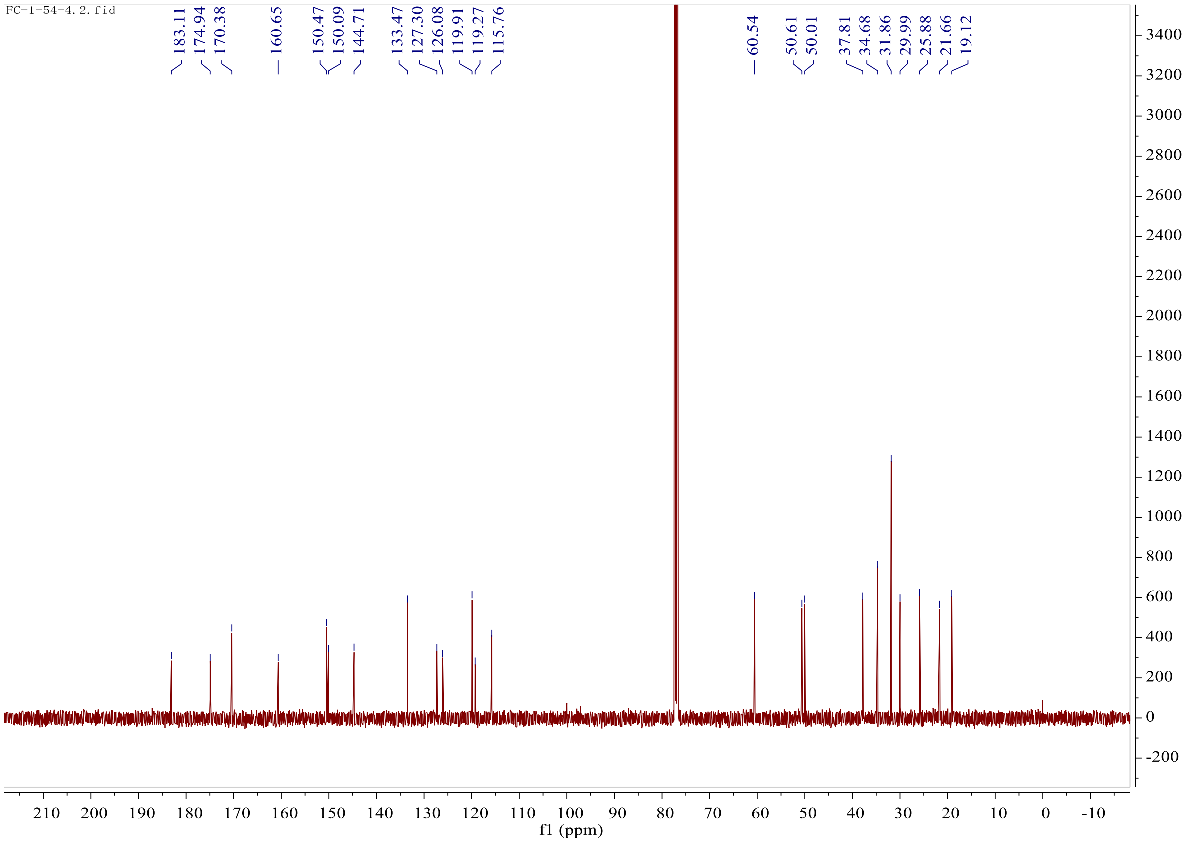
FC-1-54-4.2.fid
f1 (ppm)

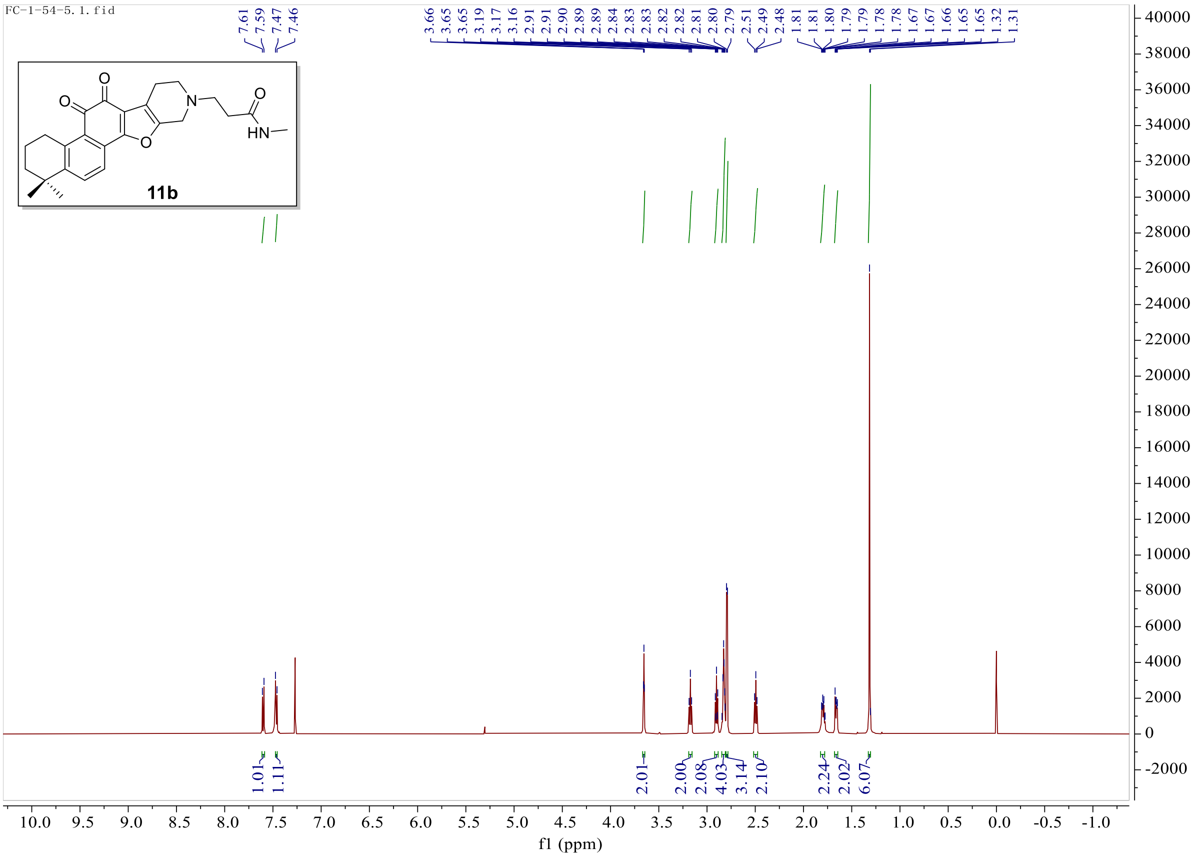
FC-1-54-5.1.fid
7.61
7.59
7.47
7.46
3.66
3.65
3.65
3.19
3.17
3.16
2.91
2.91
2.90
2.89
2.89
2.84
2.83
2.83
2.82
2.82
2.81
2.80
2.79
2.51
2.49
2.48
1.81
1.81
1.80
1.79
1.79
1.78
1.78
1.67
1.66
1.65
1.65
1.32
1.31
O
O
N
O
HN
O
11b
1.01
1.11
2.01
2.00
2.08
4.03
3.14
2.10
2.24
2.02
6.07
f1 (ppm)

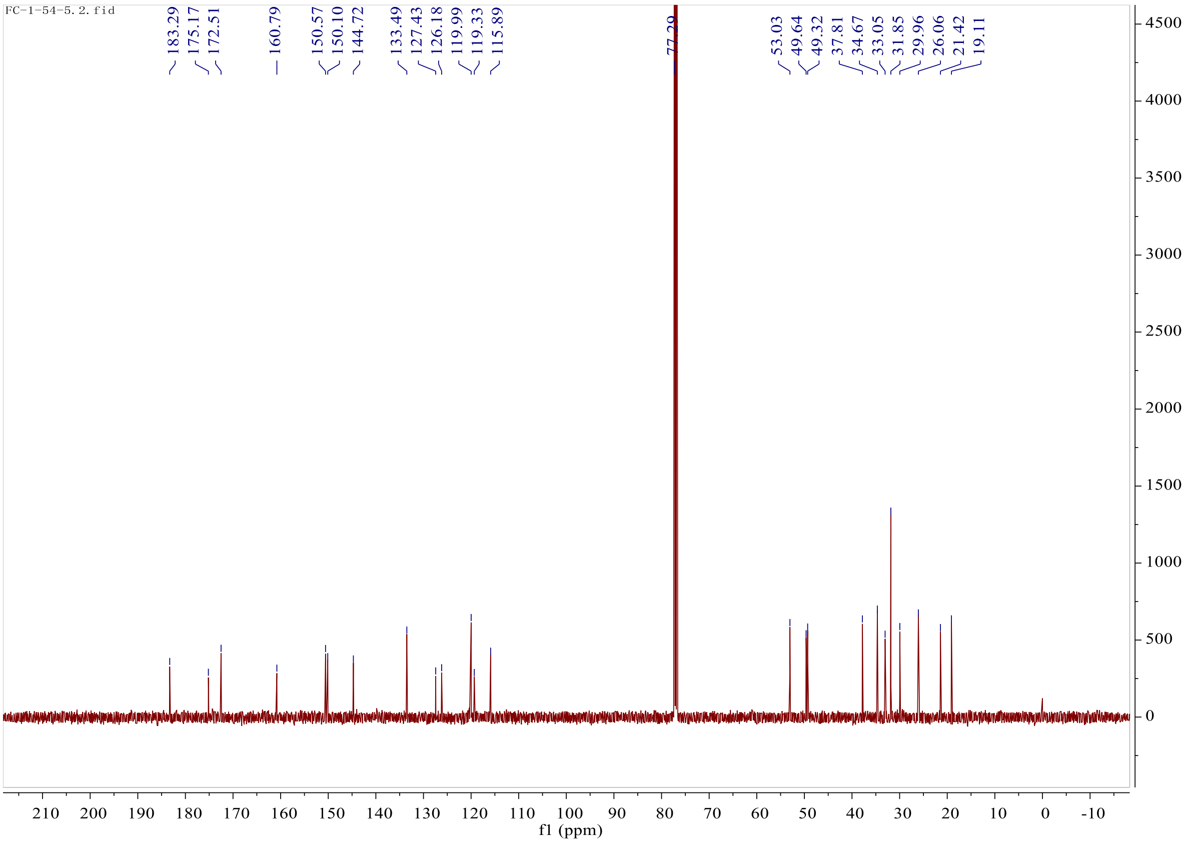
FC-1-54-5.2.fid
183.29
175.17
172.51
160.79
150.57
150.10
144.72
133.49
127.43
126.18
119.99
119.33
115.89
77.29
53.03
49.64
49.32
37.81
34.67
33.05
31.85
29.96
26.06
21.42
19.11
f1 (ppm)

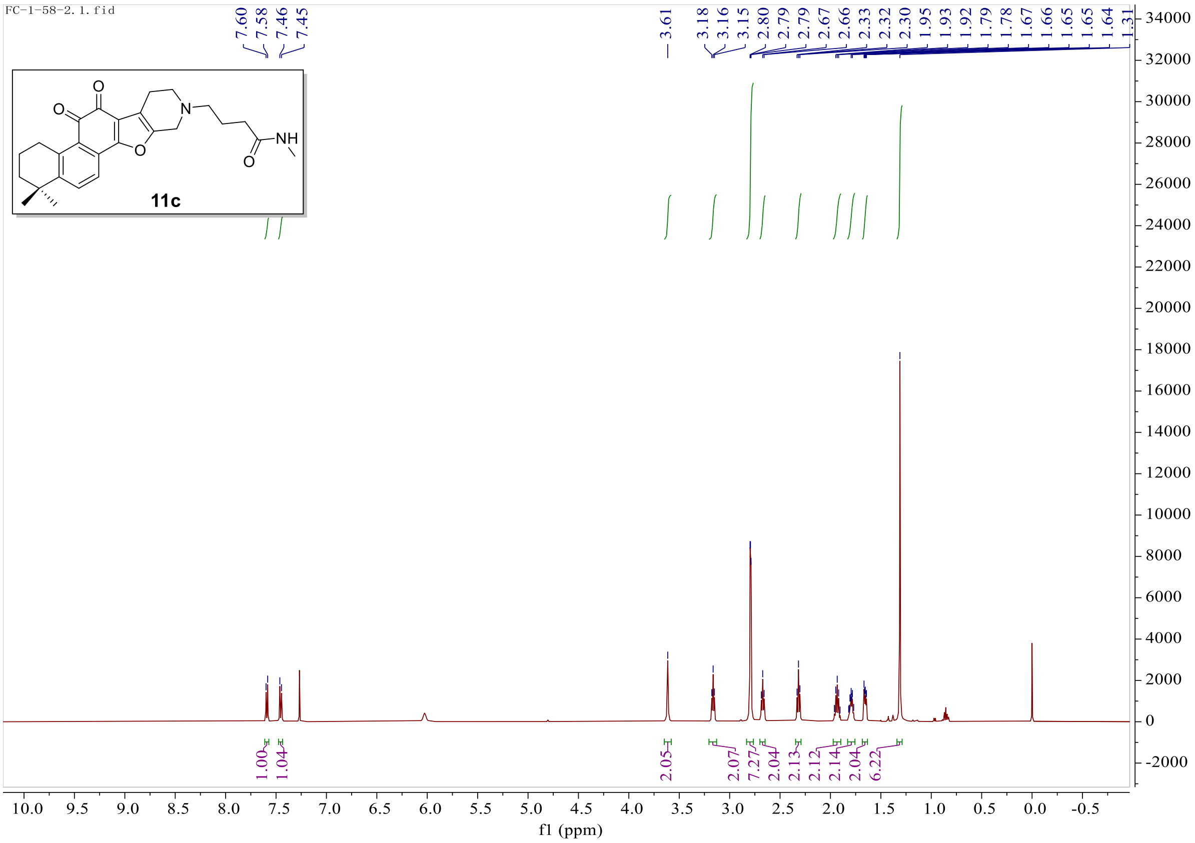
FC-1-58-2.1.fid
11c
7.60
7.58
7.46
7.45
3.61
3.18
3.16
3.15
2.80
2.79
2.77
2.67
2.66
2.33
2.32
2.30
1.95
1.93
1.92
1.79
1.78
1.67
1.66
1.65
1.65
1.64
1.31
1.00
1.04
2.05
2.07
7.27
2.04
2.13
2.12
2.14
2.04
6.22
f1 (ppm)

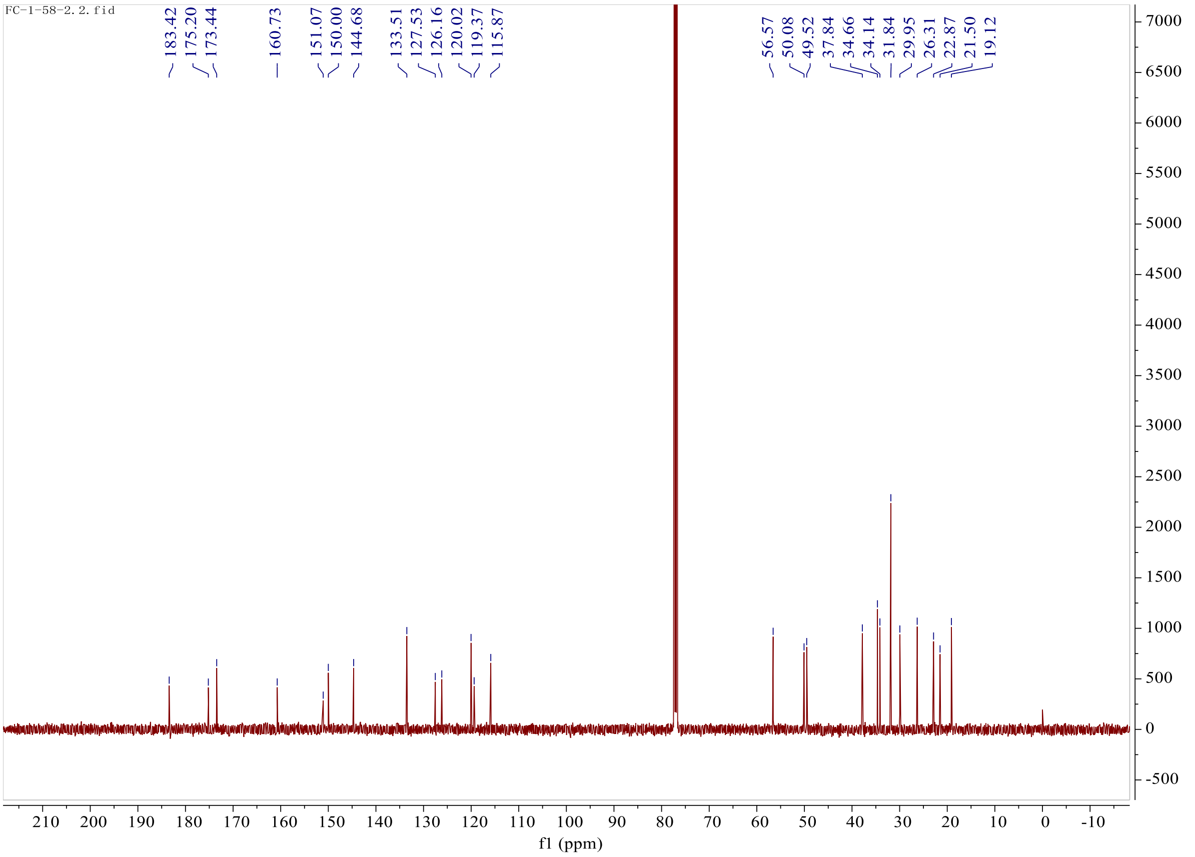
FC-1-58-2.2.fid
183.42
175.20
173.44
160.73
151.07
150.00
144.68
133.51
127.53
126.16
120.02
119.37
115.87
56.57
50.08
49.52
37.84
34.66
34.14
31.84
29.95
26.31
22.87
21.50
19.12
f1 (ppm)

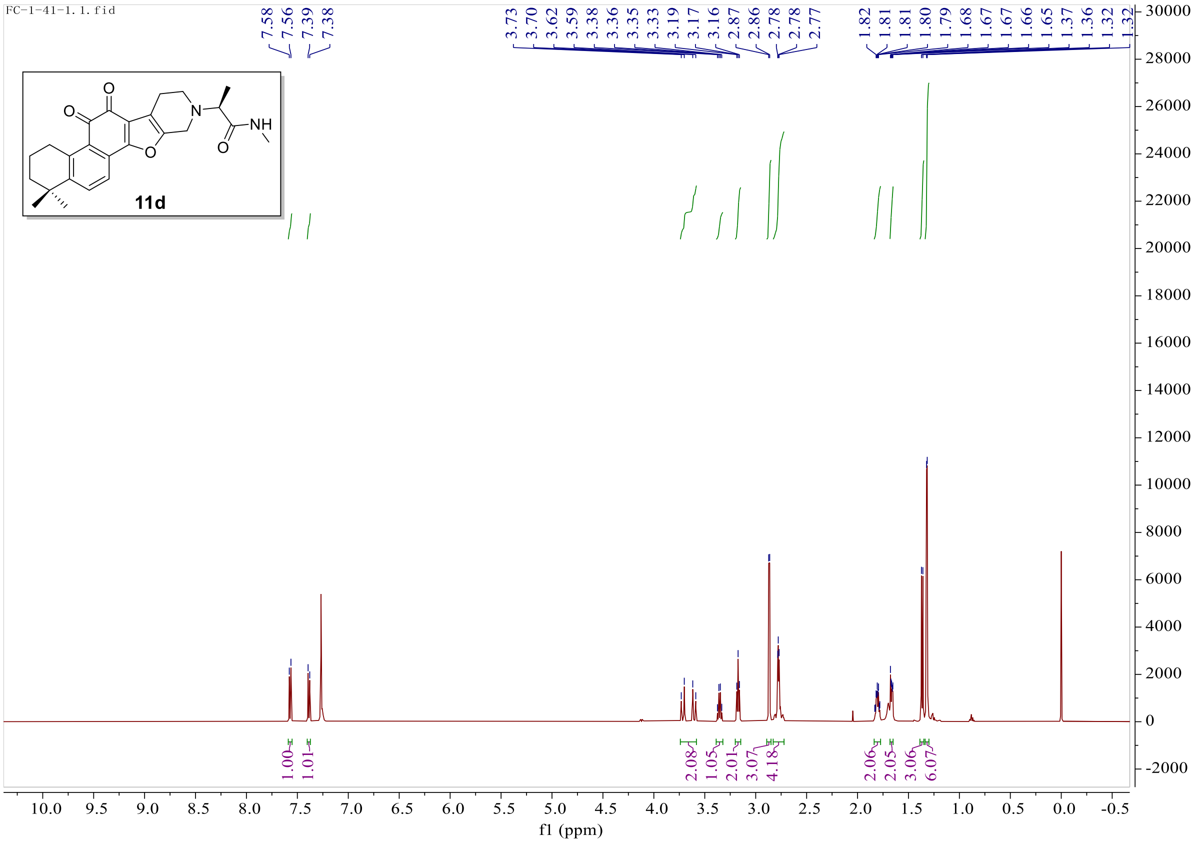
FC-1-41-1.1.fid
7.58
7.56
7.39
7.38
3.73
3.70
3.62
3.59
3.38
3.36
3.35
3.33
3.19
3.17
3.16
2.87
2.86
2.78
2.78
2.77
1.82
1.81
1.81
1.80
1.79
1.68
1.67
1.67
1.66
1.65
1.37
1.36
1.32
1.32
11d
1.00
1.01
2.08
1.05
2.01
3.07
4.18
2.06
2.05
3.06
6.07
f1 (ppm)

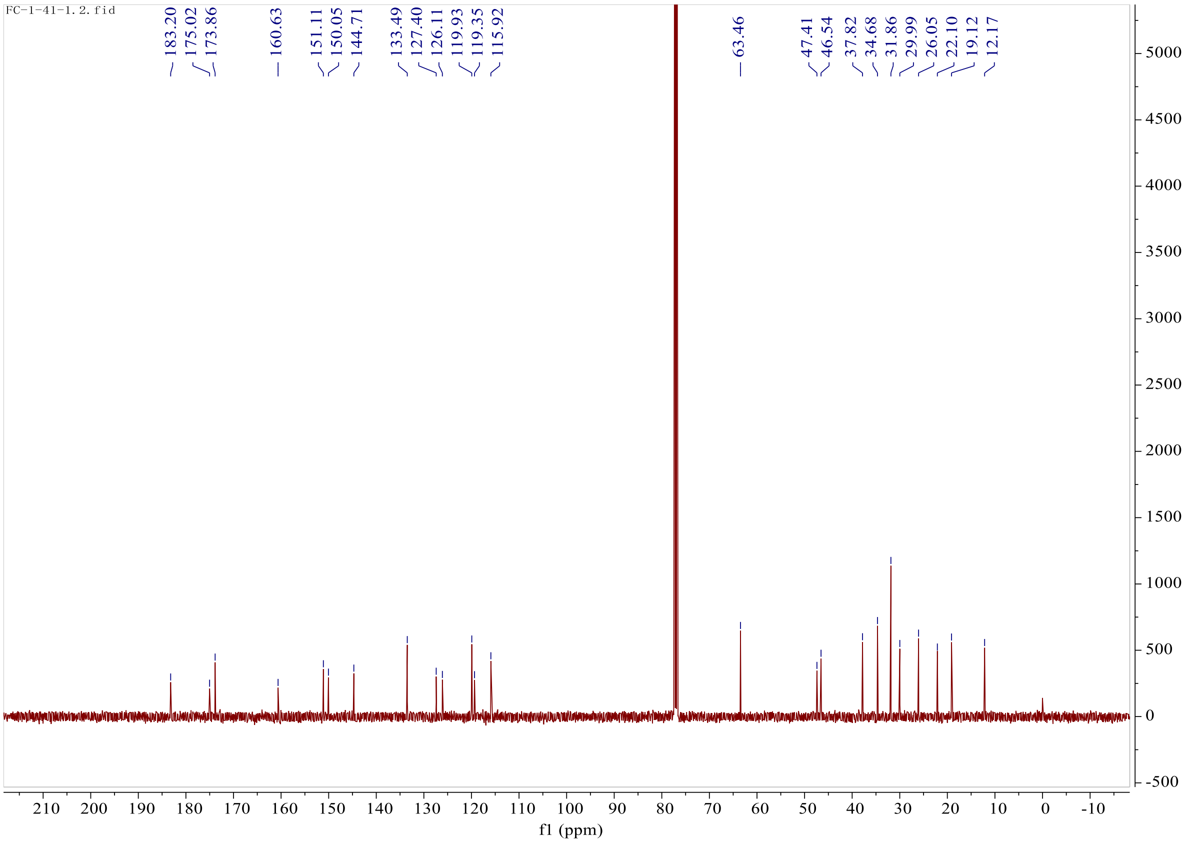
FC-1-41-1.2.fid
183.20
175.02
173.86
160.63
151.11
150.05
144.71
133.49
127.40
126.11
119.93
119.35
115.92
63.46
47.41
46.54
37.82
34.68
31.86
29.99
26.05
22.10
19.12
12.17
f1 (ppm)

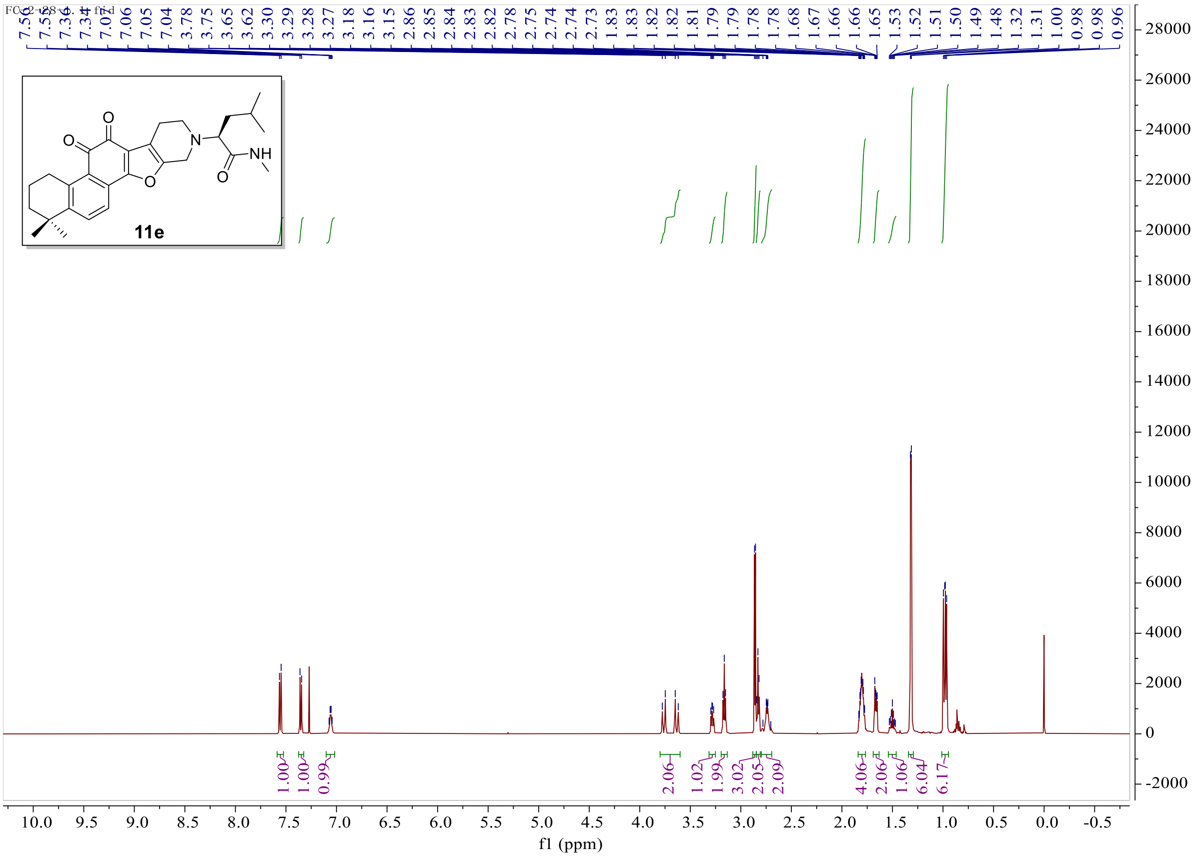
7.36
7.34
7.07
7.06
7.05
7.04
3.78
3.75
3.65
3.62
3.30
3.29
3.28
3.27
3.18
3.16
3.15
2.86
2.85
2.84
2.83
2.82
2.78
2.75
2.74
2.74
2.73
1.83
1.83
1.82
1.82
1.81
1.79
1.79
1.78
1.78
1.68
1.67
1.66
1.66
1.65
1.53
1.52
1.51
1.50
1.49
1.48
1.32
1.31
1.00
0.98
0.96
11e
1.00
1.00
0.99
2.06
1.02
1.99
3.02
2.05
2.09
4.06
2.06
1.06
6.04
6.17
f1 (ppm)

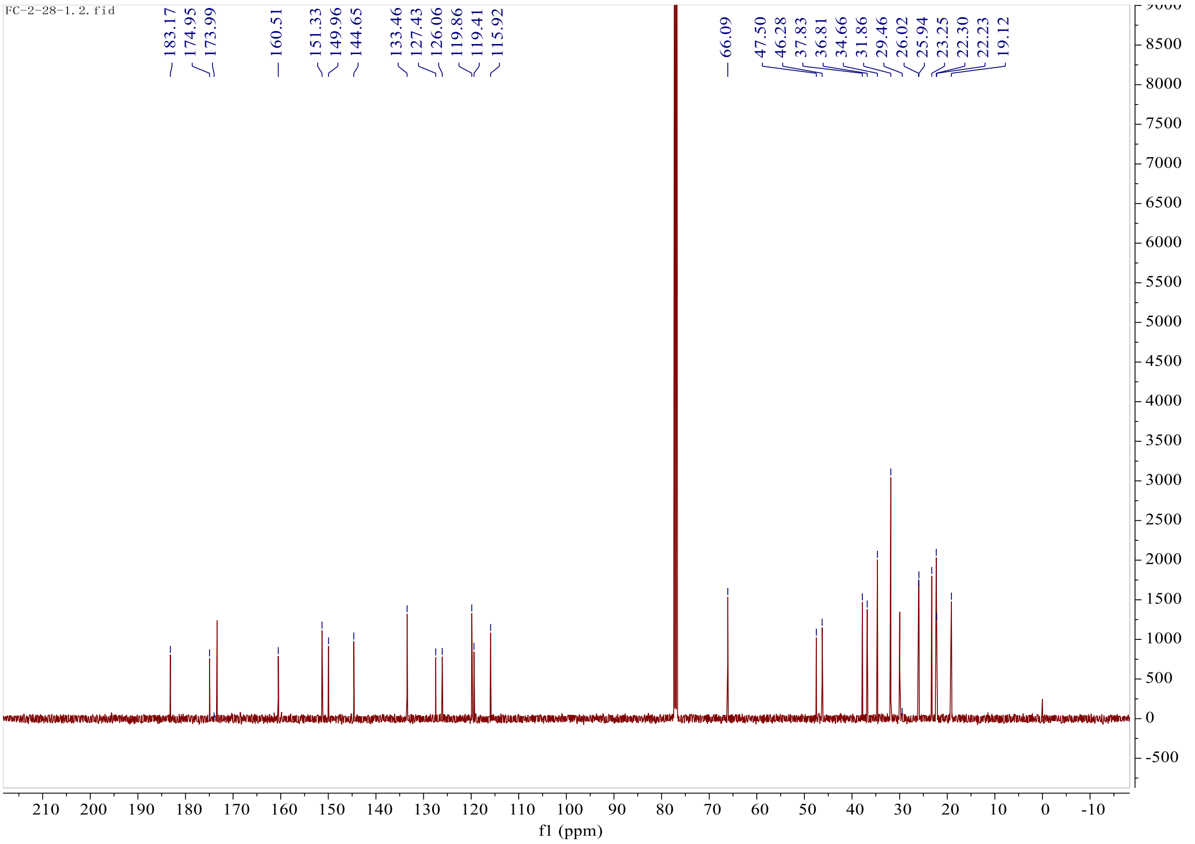
FC-2-28-1.2.fid
183.17
174.95
173.99
160.51
151.33
149.96
144.65
133.46
127.43
126.06
119.86
119.41
115.92
66.09
47.50
46.28
37.83
36.81
34.66
31.86
29.46
26.02
25.94
23.25
22.30
22.23
19.12
f1 (ppm)

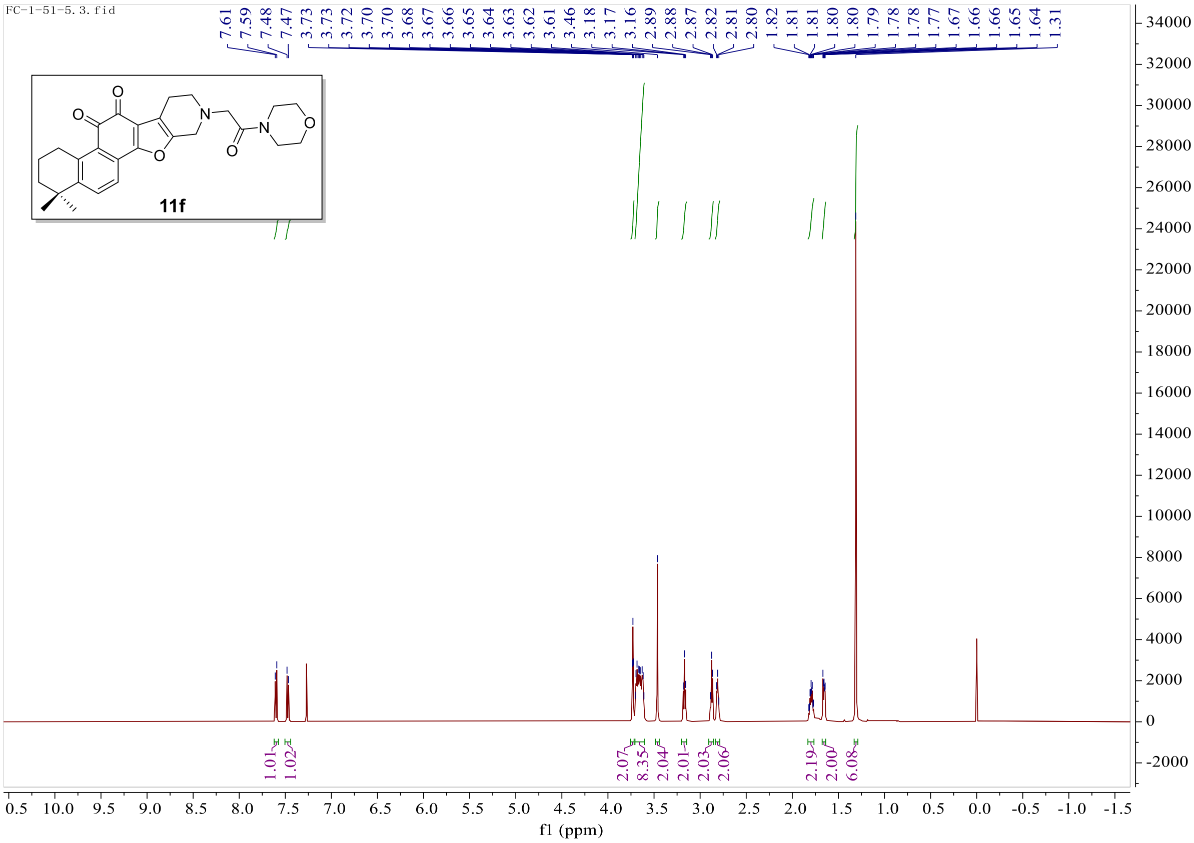
FC-1-51-5.3.fid
7.61 7.59 7.48 7.47 3.73 3.73 3.72 3.70 3.70 3.68 3.67 3.66 3.65 3.64 3.63 3.62 3.61 3.46 3.18 3.17 3.16 2.89 2.88 2.87 2.82 2.81 2.80 1.82 1.81 1.81 1.80 1.80 1.79 1.78 1.78 1.77 1.67 1.66 1.66 1.65 1.64 1.31
11f
1.01 1.02 2.07 8.35 2.04 2.01 2.03 2.06 2.19 2.00 6.08
f1 (ppm)

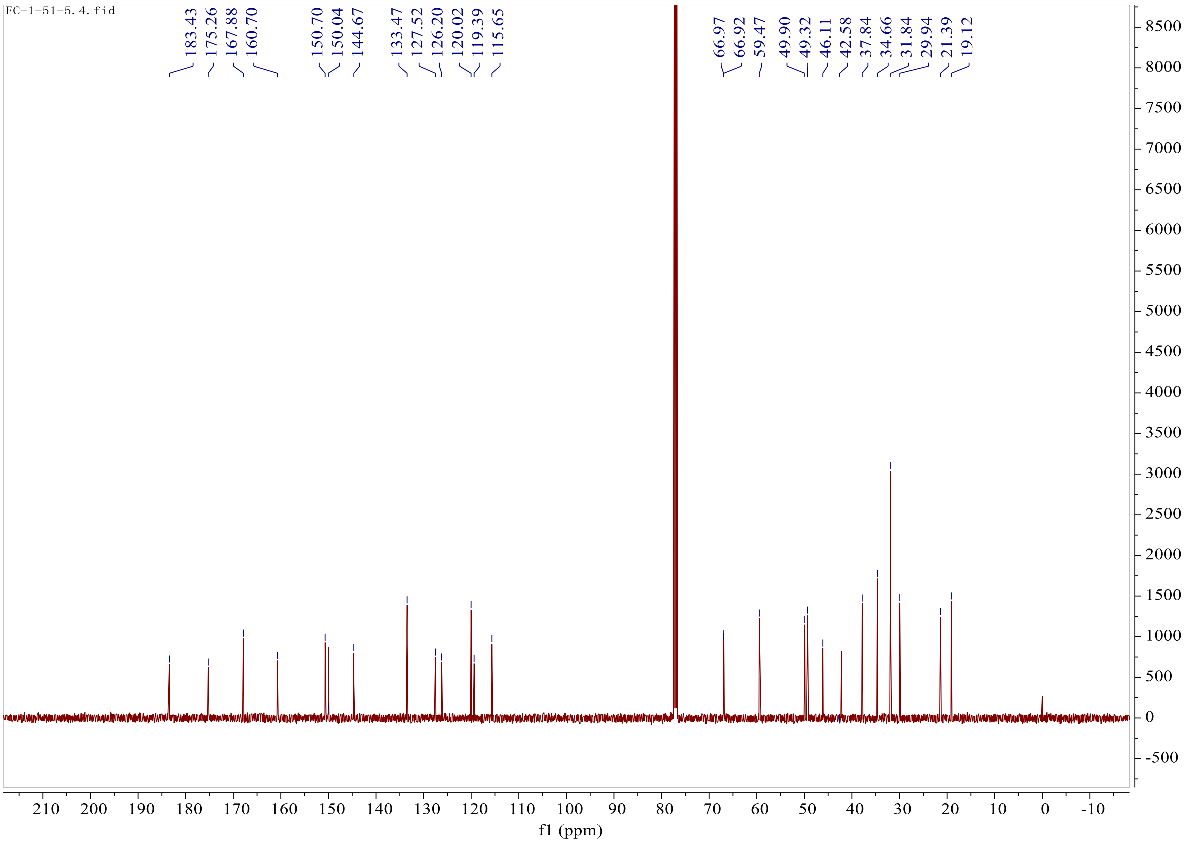
FC-1-51-5.4.fid
183.43 175.26 167.88 160.70 150.70 150.04 144.67 133.47 127.52 126.20 120.02 119.39 115.65 66.97 66.92 59.47 49.90 49.32 46.11 42.58 37.84 34.66 31.84 29.94 21.39 19.12
f1 (ppm)

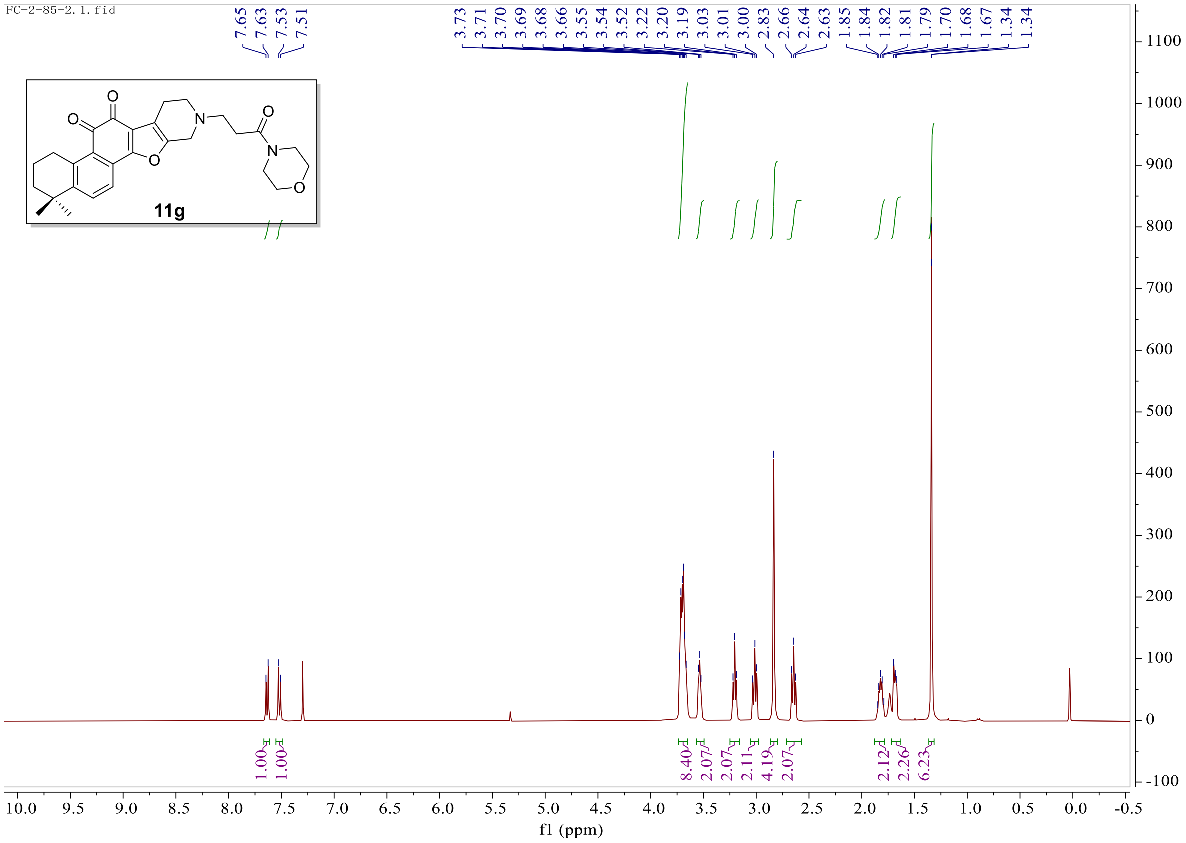
FC-2-85-2.1.fid
11g
7.65
7.63
7.53
7.51
3.73
3.71
3.70
3.69
3.68
3.66
3.55
3.54
3.52
3.22
3.20
3.19
3.03
3.01
3.00
2.83
2.66
2.64
2.63
1.85
1.84
1.82
1.81
1.79
1.70
1.68
1.67
1.34
1.34
1.00
1.00
8.40
2.07
2.07
2.11
4.19
2.07
2.12
2.26
6.23
f1 (ppm)

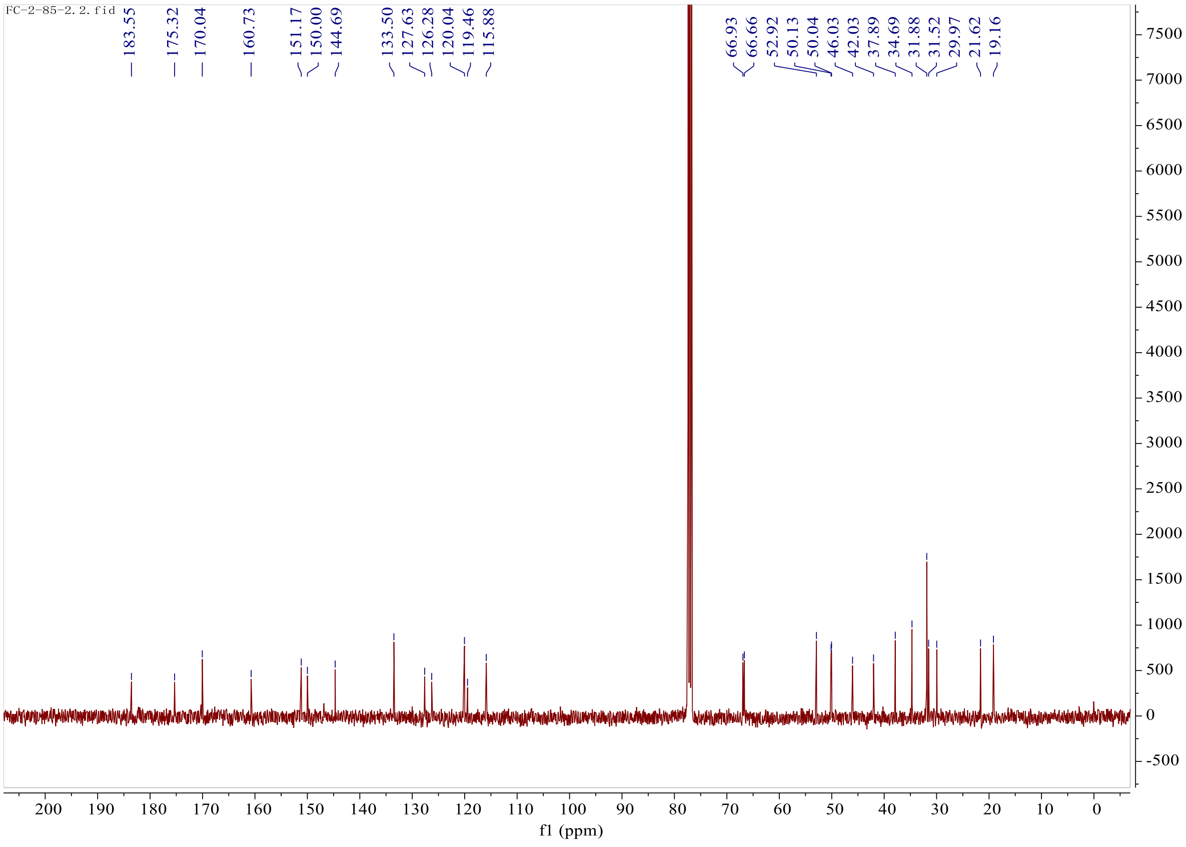
FC-2-85-2.2.fid
183.55
175.32
170.04
160.73
151.17
150.00
144.69
133.50
127.63
126.28
120.04
119.46
115.88
66.93
66.66
52.92
50.13
50.04
46.03
42.03
37.89
34.69
31.88
31.52
29.97
21.62
19.16
f1 (ppm)

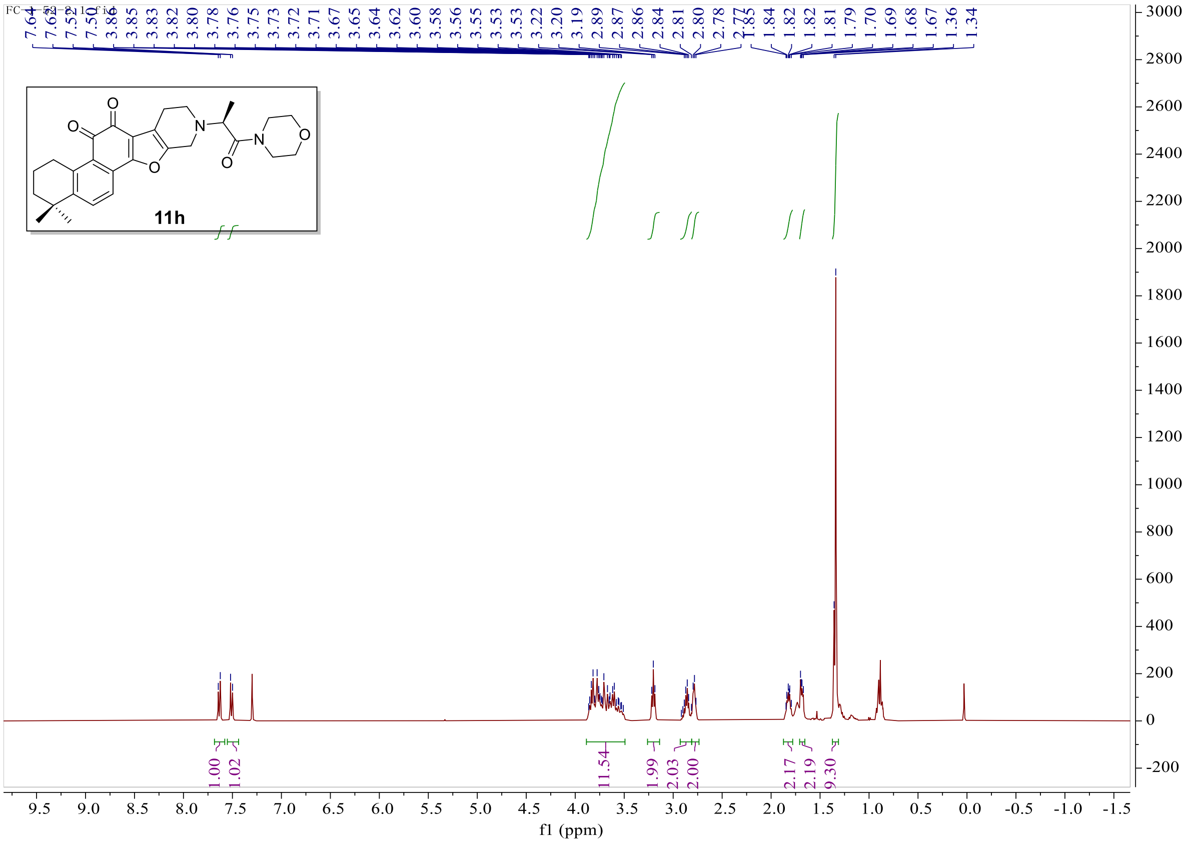

7.64 7.62 7.52 7.50 3.86 3.85 3.83 3.82 3.80 3.78 3.76 3.75 3.73 3.72 3.71 3.67 3.65 3.64 3.62 3.60 3.58 3.56 3.55 3.53 3.53 3.22 3.20 3.19 2.89 2.87 2.86 2.84 2.81 2.80 2.78 2.77 1.85 1.84 1.82 1.82 1.81 1.79 1.70 1.69 1.68 1.67 1.36 1.34
11h
1.00 1.02 11.54 1.99 2.03 2.00 2.17 2.19 9.30
f1 (ppm)

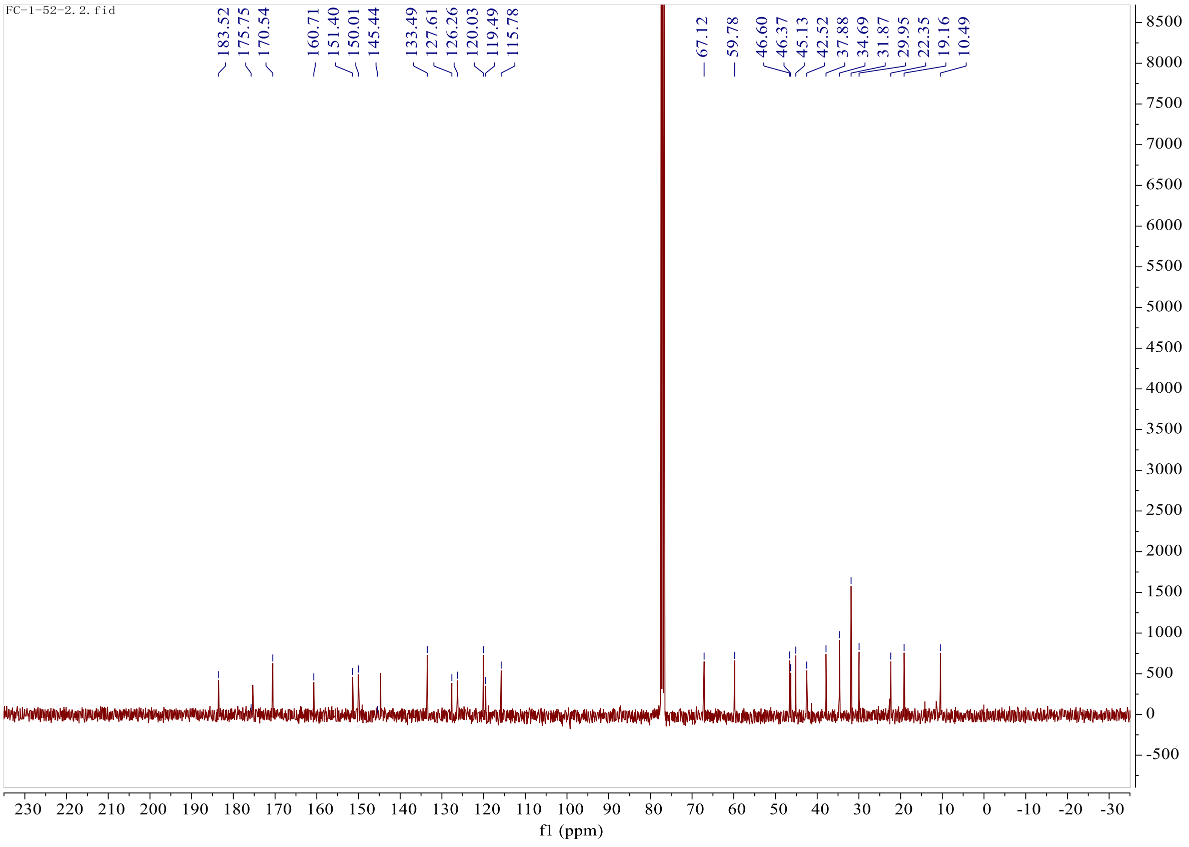

FC-1-52-2.2.fid
183.52 175.75 170.54 160.71 151.40 150.01 145.44 133.49 127.61 126.26 120.03 119.49 115.78 67.12 59.78 46.60 46.37 45.13 42.52 37.88 34.69 31.87 29.95 22.35 19.16 10.49
f1 (ppm)

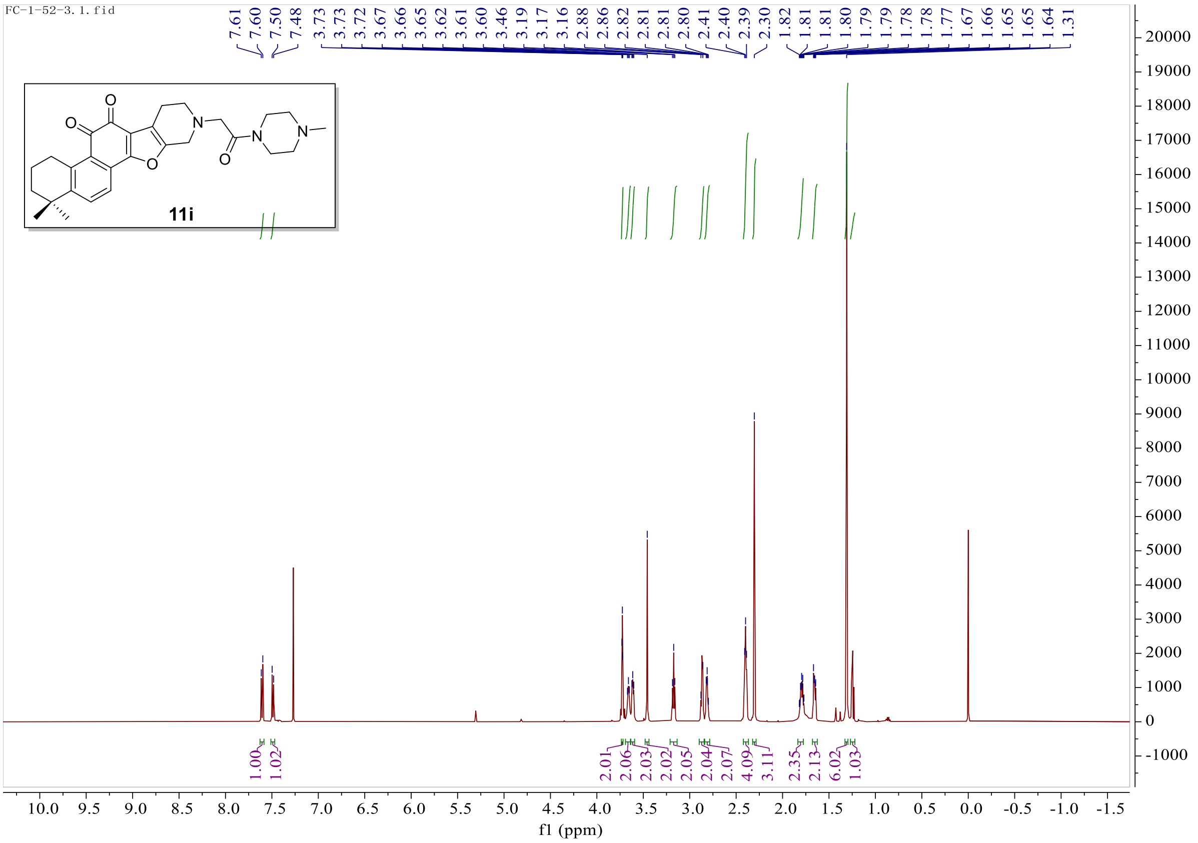
FC-1-52-3.1.fid
7.61 7.60 7.50 7.48 3.73 3.73 3.72 3.67 3.66 3.65 3.62 3.61 3.60 3.46 3.19 3.17 3.16 2.88 2.86 2.82 2.81 2.81 2.80 2.41 2.40 2.39 2.30 1.82 1.81 1.81 1.80 1.79 1.79 1.78 1.78 1.77 1.67 1.66 1.65 1.65 1.64 1.31
11i
1.00 1.02 2.01 2.06 2.03 2.02 2.05 2.04 2.07 4.09 3.11 2.35 2.13 6.02 1.03
f1 (ppm)

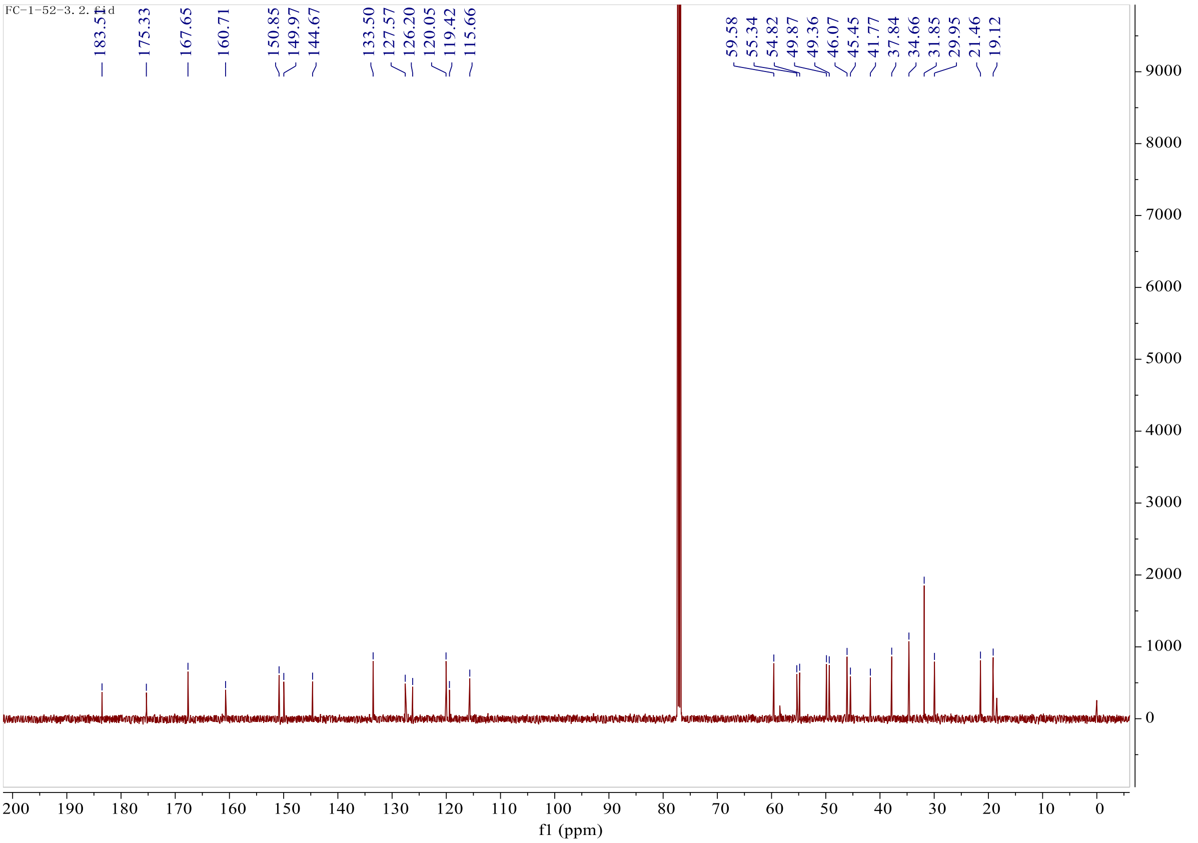
FC-1-52-3.2.fid
183.51 175.33 167.65 160.71 150.85 149.97 144.67 133.50 127.57 126.20 120.05 119.42 115.66
59.58 55.34 54.82 49.87 49.36 46.07 45.45 41.77 37.84 34.66 31.85 29.95 21.46 19.12
f1 (ppm)

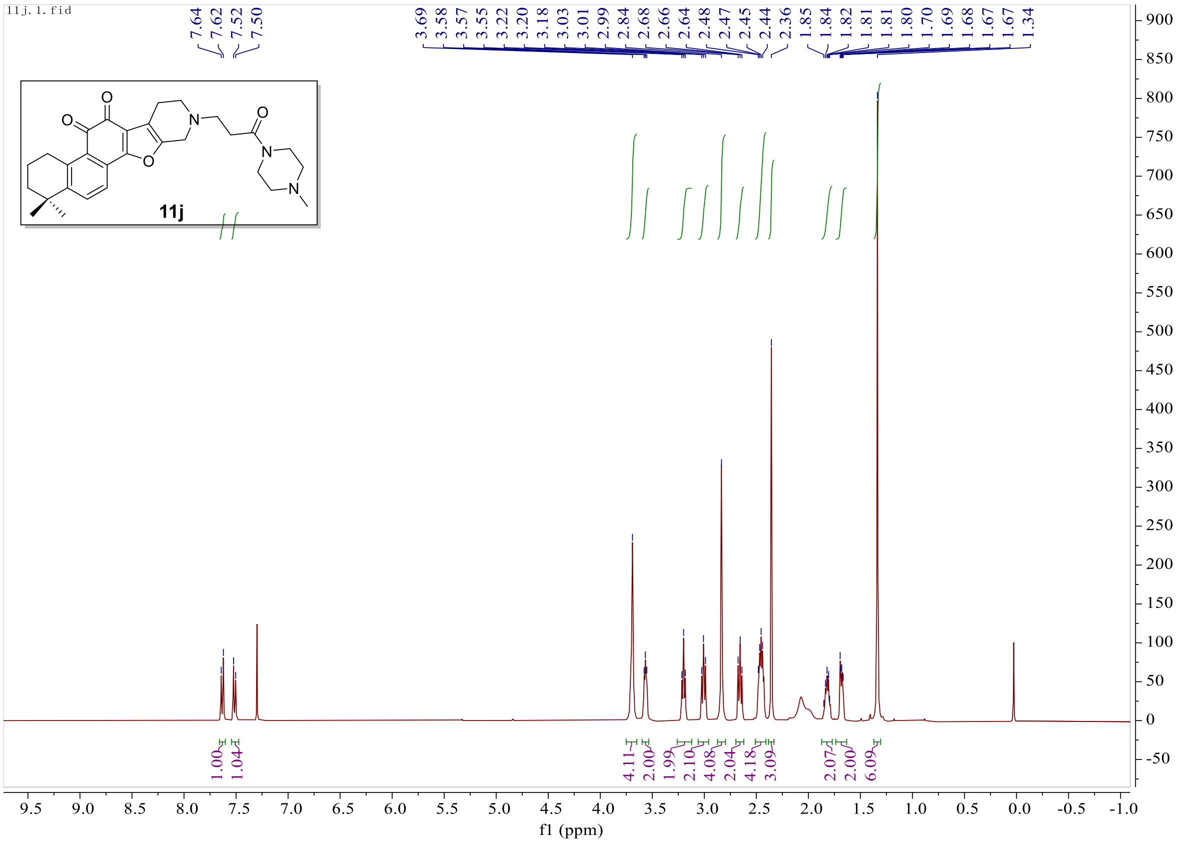
11j. 1. fid
7.64
7.62
7.52
7.50
3.69
3.58
3.57
3.55
3.22
3.20
3.18
3.03
3.01
2.99
2.84
2.68
2.66
2.64
2.48
2.47
2.45
2.44
2.36
1.85
1.84
1.82
1.81
1.81
1.80
1.70
1.69
1.68
1.67
1.67
1.34
11j
1.00
1.04
4.11
2.00
1.99
2.10
4.08
2.04
4.18
3.09
2.07
2.00
6.09
f1 (ppm)

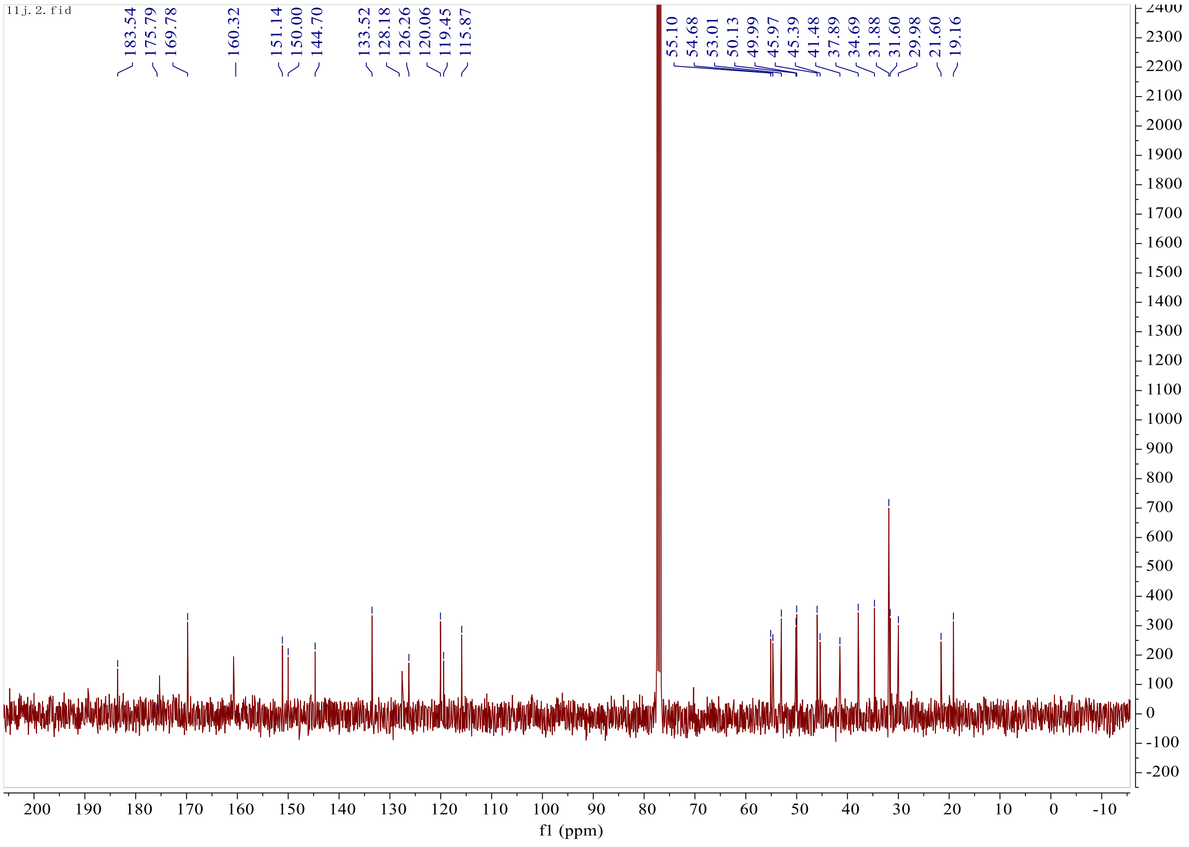
11j. 2. fid
183.54
175.79
169.78
160.32
151.14
150.00
144.70
133.52
128.18
126.26
120.06
119.45
115.87
55.10
54.68
53.01
50.13
49.99
45.97
45.39
41.48
37.89
34.69
31.88
31.60
29.98
21.60
19.16
f1 (ppm)

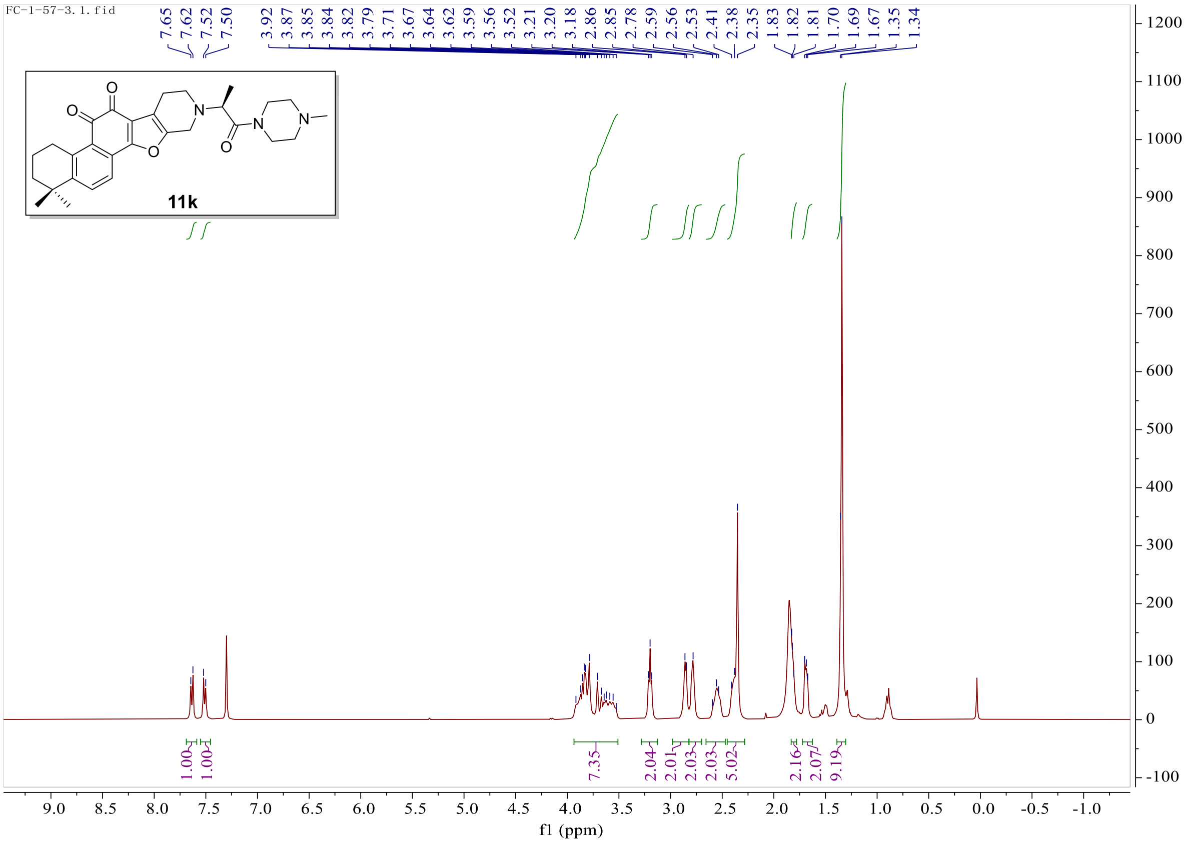
FC-1-57-3.1.fid
7.65
7.62
7.52
7.50
3.92
3.87
3.85
3.84
3.82
3.79
3.71
3.67
3.64
3.62
3.59
3.56
3.52
3.21
3.20
3.18
2.86
2.85
2.78
2.59
2.56
2.53
2.41
2.38
2.35
1.83
1.82
1.81
1.70
1.69
1.67
1.35
1.34
11k
1.00
1.00
7.35
2.04
2.01
2.03
2.03
5.02
2.16
2.07
9.19
f1 (ppm)

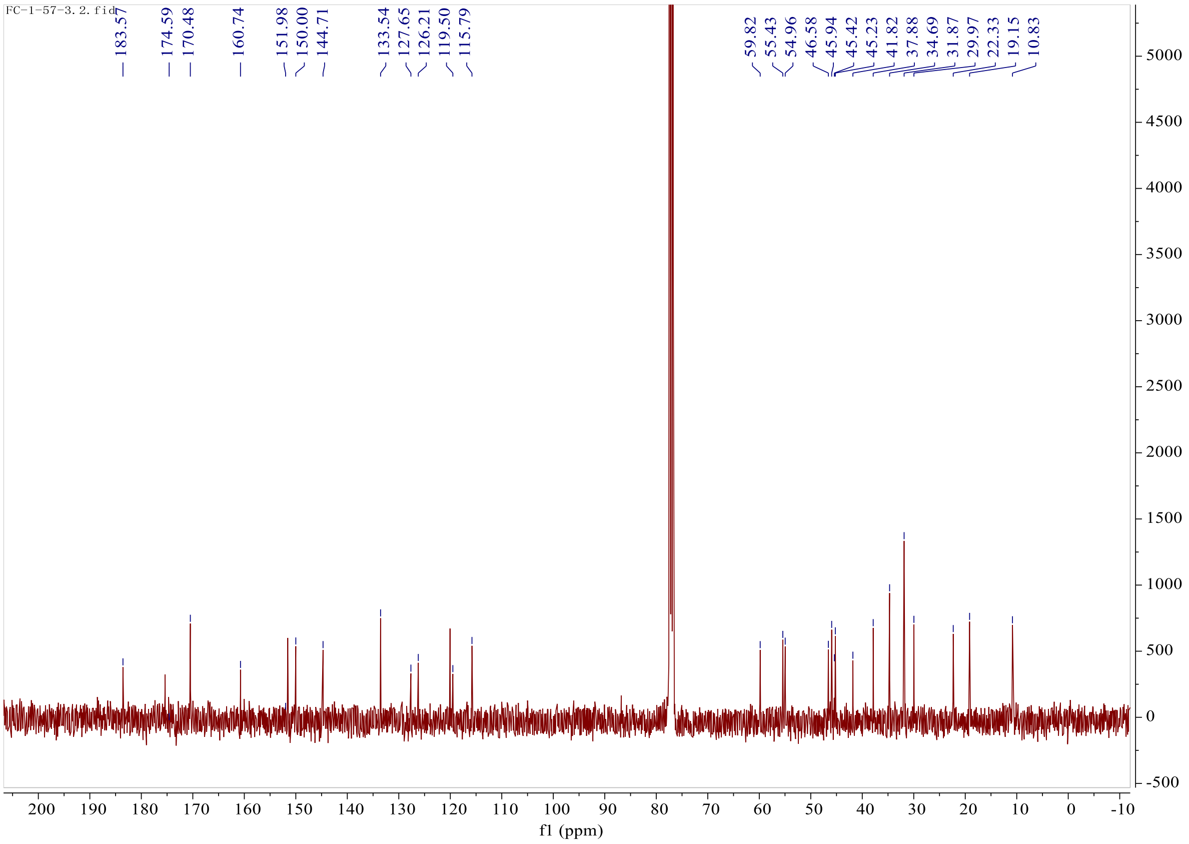
FC-1-57-3.2.fid
183.57
174.59
170.48
160.74
151.98
150.00
144.71
133.54
127.65
126.21
119.50
115.79
59.82
55.43
54.96
46.58
45.94
45.42
45.23
41.82
37.88
34.69
31.87
29.97
22.33
19.15
10.83
f1 (ppm)

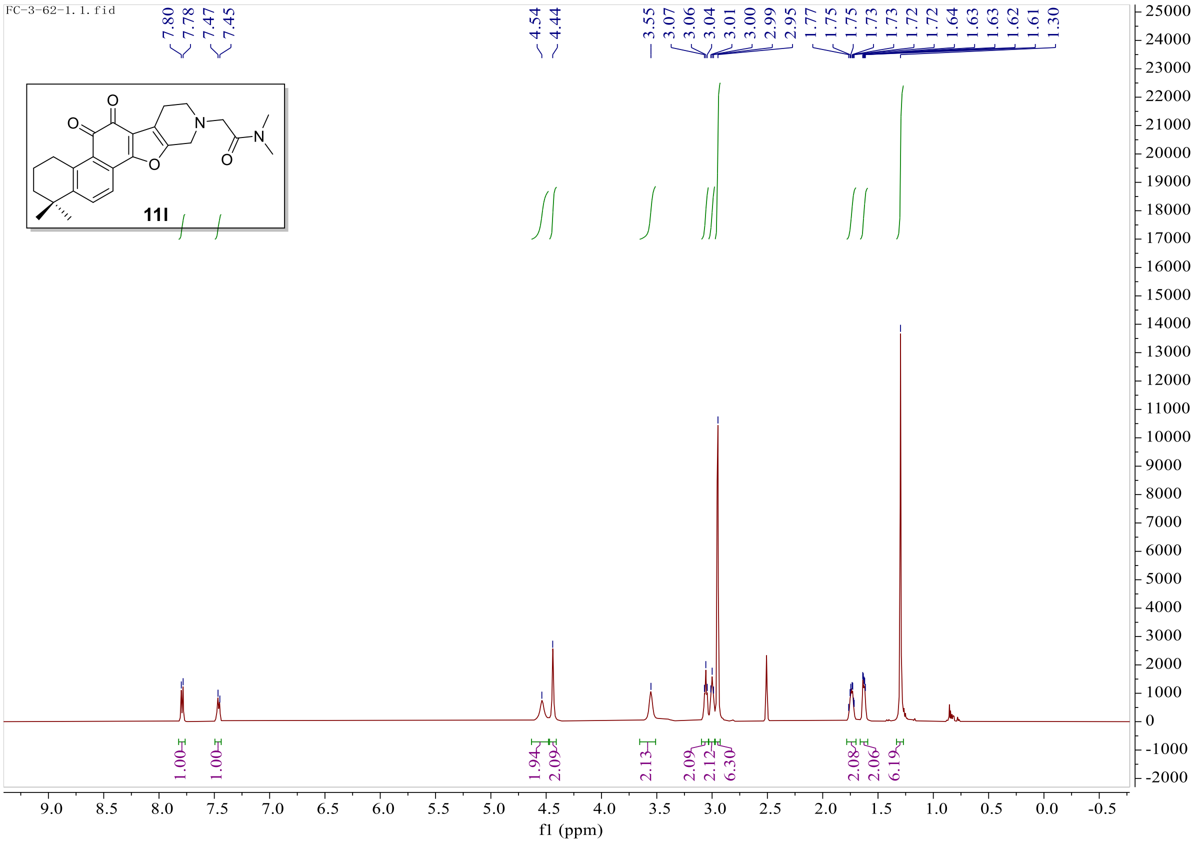
FC-3-62-1.1.fid
7.80
7.78
7.47
7.45
4.54
4.44
3.55
3.07
3.06
3.04
3.01
3.00
2.99
2.95
1.77
1.75
1.75
1.73
1.73
1.72
1.72
1.64
1.63
1.63
1.62
1.61
1.30
11l
1.00
1.00
1.94
2.09
2.13
2.09
2.12
6.30
2.08
2.06
6.19
f1 (ppm)

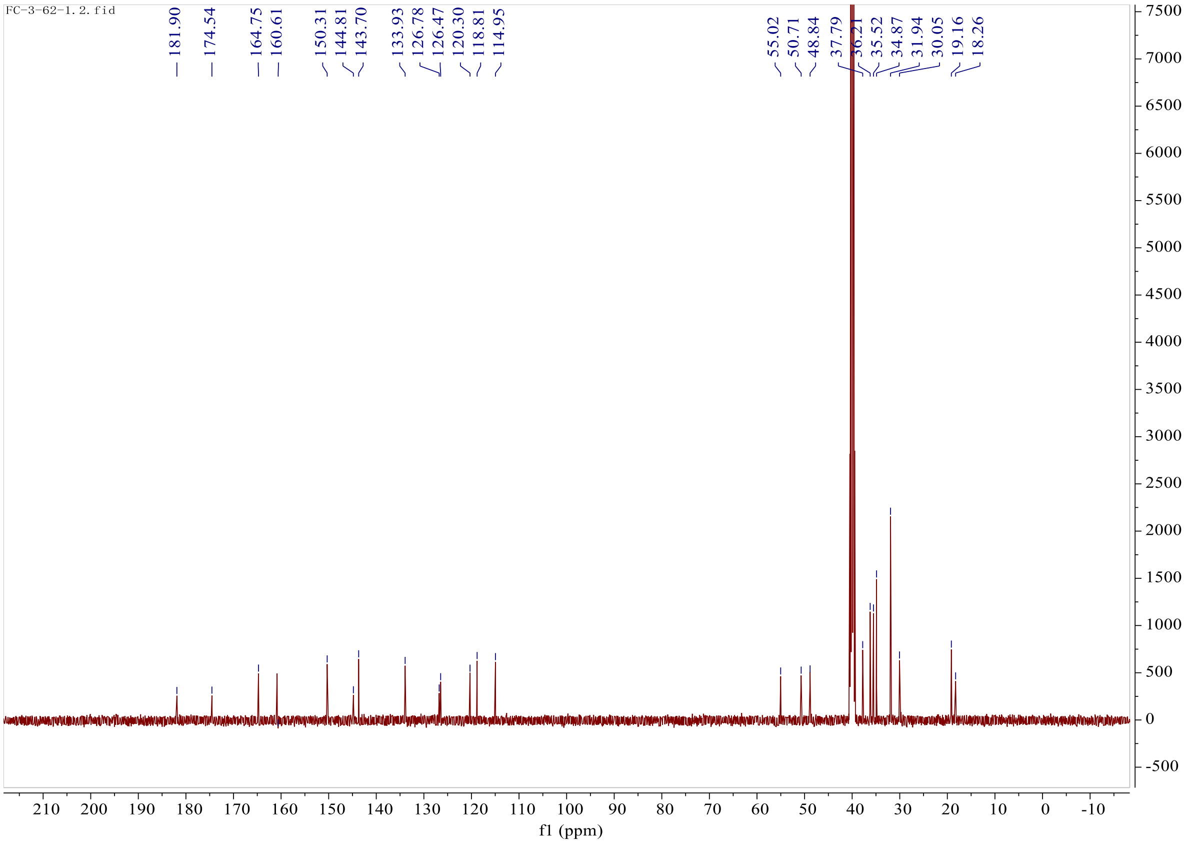
FC-3-62-1.2.fid
181.90
174.54
164.75
160.61
150.31
144.81
143.70
133.93
126.78
126.47
120.30
118.81
114.95
55.02
50.71
48.84
37.79
36.21
35.52
34.87
31.94
30.05
19.16
18.26
f1 (ppm)

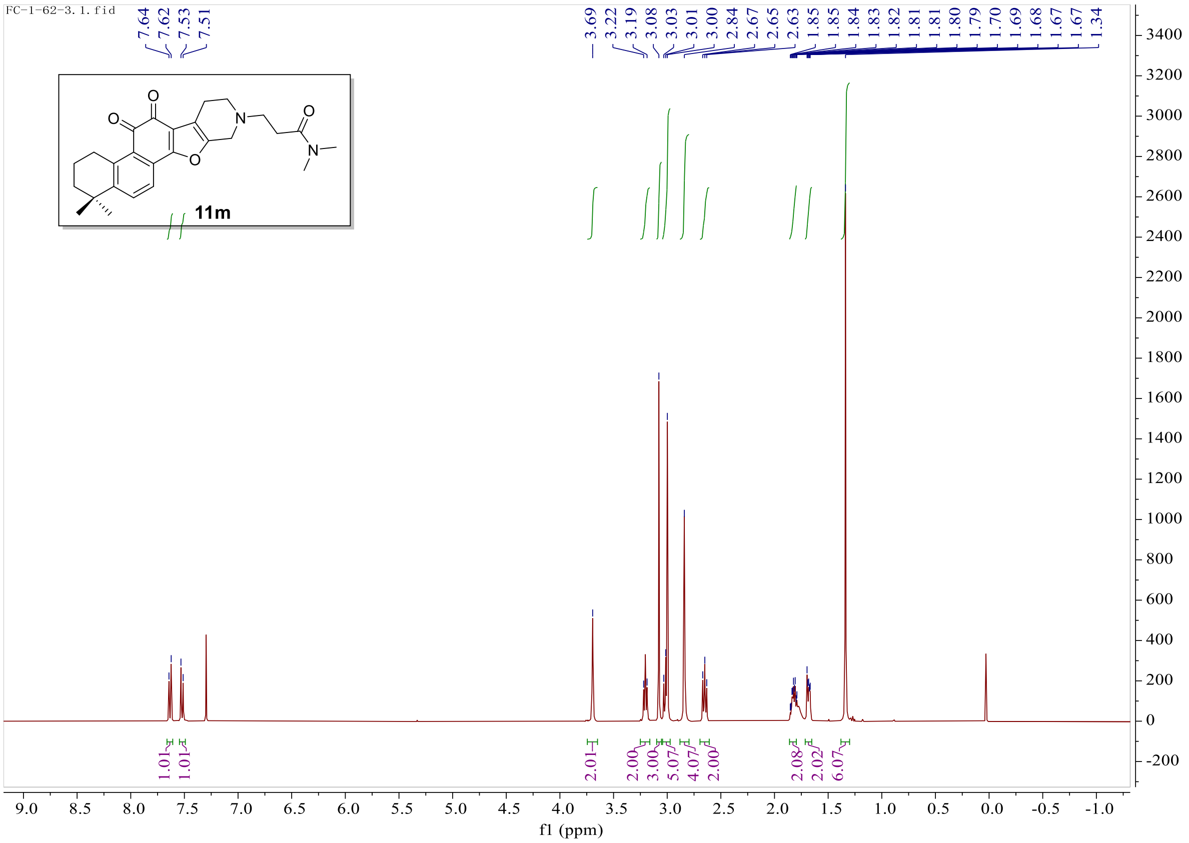
FC-1-62-3.1.fid
7.64
7.62
7.53
7.51
3.69
3.22
3.19
3.08
3.03
3.01
3.00
2.84
2.67
2.65
2.63
1.85
1.85
1.84
1.83
1.82
1.81
1.81
1.80
1.79
1.70
1.69
1.68
1.67
1.67
1.34
11m
1.01
1.01
2.01
2.00
3.00
5.07
4.07
2.00
2.08
2.02
6.07
f1 (ppm)

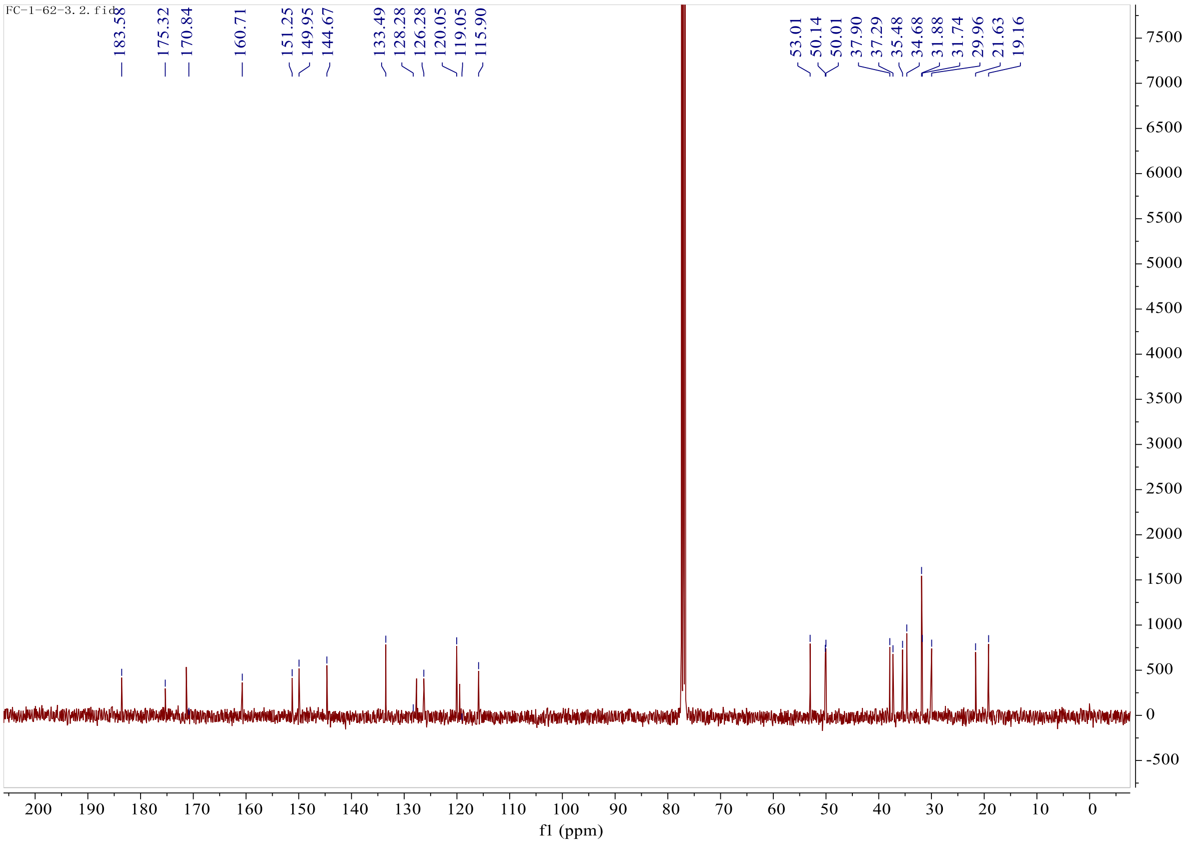
FC-1-62-3.2.fid
183.58
175.32
170.84
160.71
151.25
149.95
144.67
133.49
128.28
126.28
120.05
119.05
115.90
53.01
50.14
50.01
37.90
37.29
35.48
34.68
31.88
31.74
29.96
21.63
19.16
f1 (ppm)

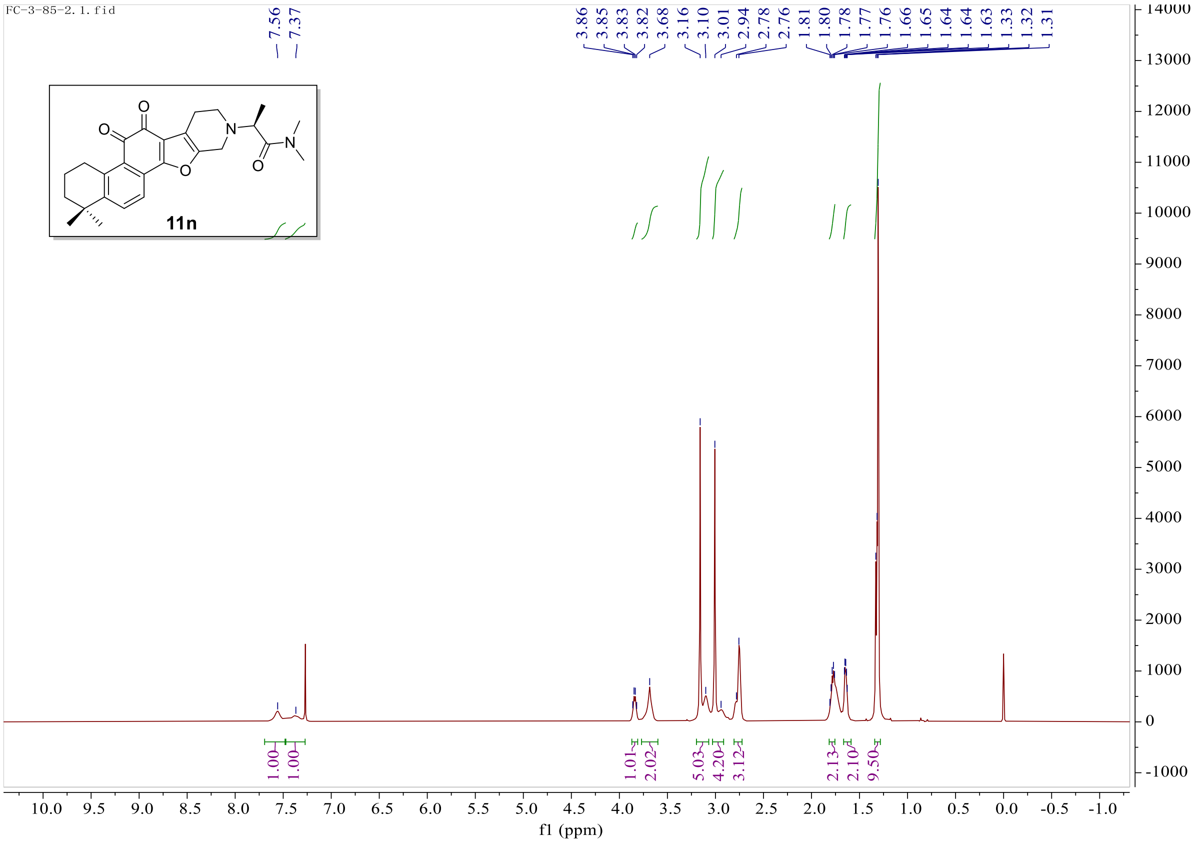
FC-3-85-2.1.fid
11n
7.56
7.37
3.86
3.85
3.83
3.82
3.68
3.16
3.10
3.01
2.94
2.78
2.76
1.81
1.80
1.78
1.77
1.76
1.66
1.65
1.64
1.64
1.63
1.33
1.32
1.31
1.00
1.00
1.01
2.02
5.03
4.20
3.12
2.13
2.10
9.50
f1 (ppm)

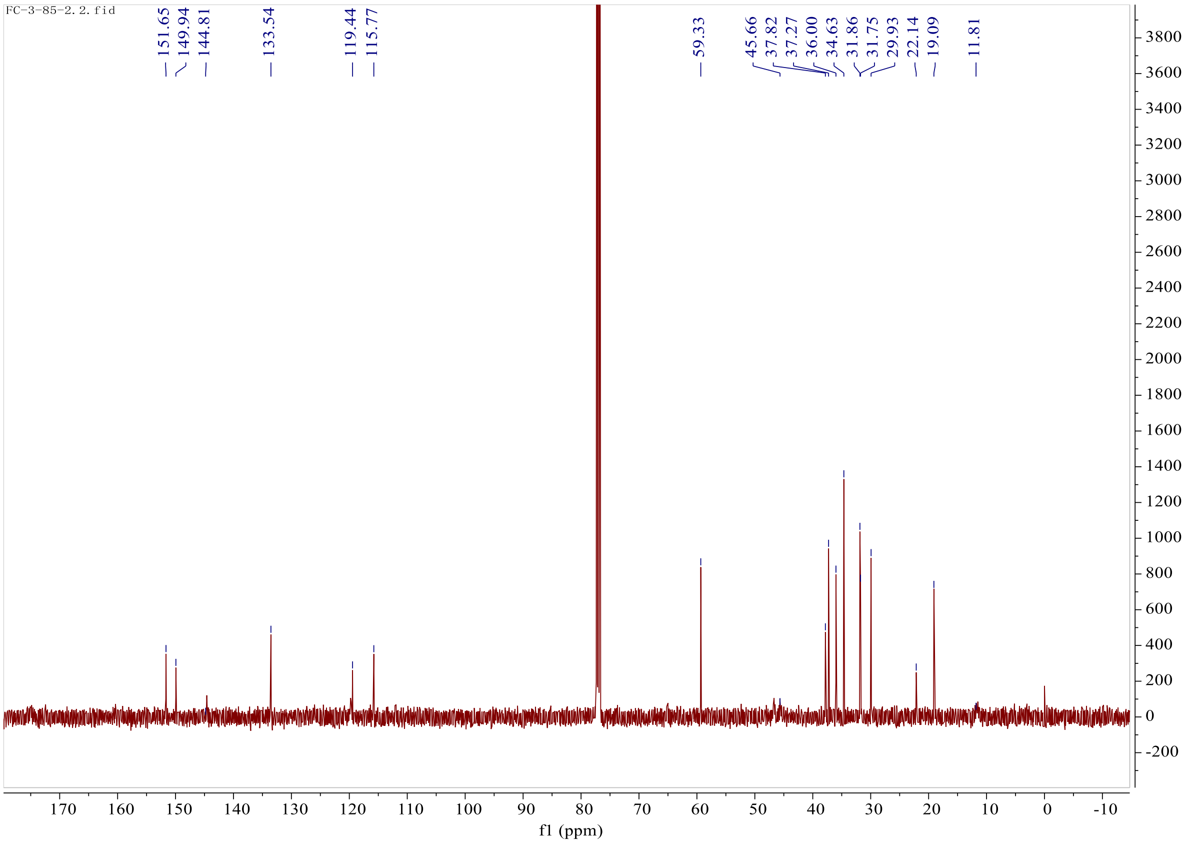
FC-3-85-2.2.fid
151.65
149.94
144.81
133.54
119.44
115.77
59.33
45.66
37.82
37.27
36.00
34.63
31.86
31.75
29.93
22.14
19.09
11.81
f1 (ppm)

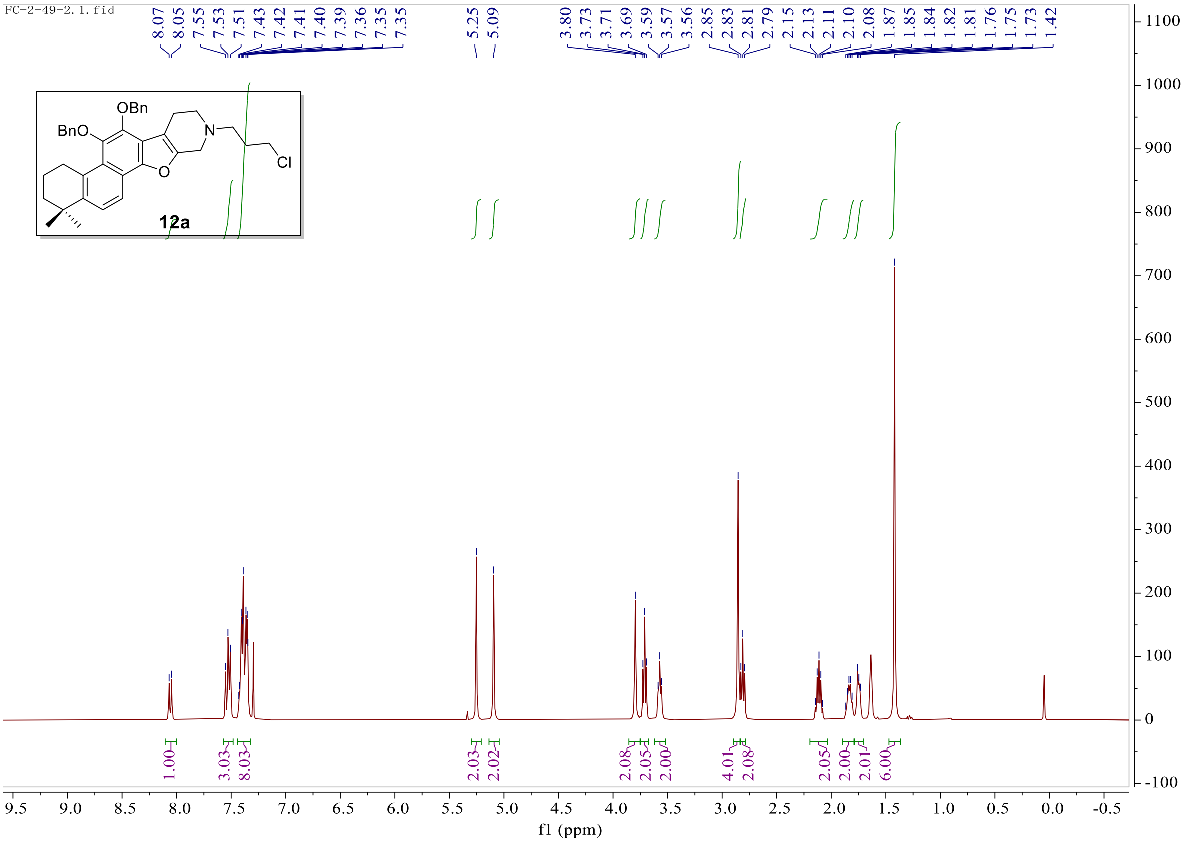

FC-2-49-2.1.fid
8.07
8.05
7.55
7.53
7.51
7.43
7.42
7.41
7.40
7.39
7.36
7.35
7.35
5.25
5.09
3.80
3.73
3.71
3.69
3.59
3.57
3.56
2.85
2.83
2.81
2.79
2.15
2.13
2.11
2.10
2.08
1.87
1.85
1.84
1.82
1.81
1.76
1.75
1.73
1.42
OBn
BnO
N
Cl
O
12a
1.00
3.03
8.03
2.03
2.02
2.08
2.05
2.00
4.01
2.08
2.05
2.00
2.01
6.00
f1 (ppm)

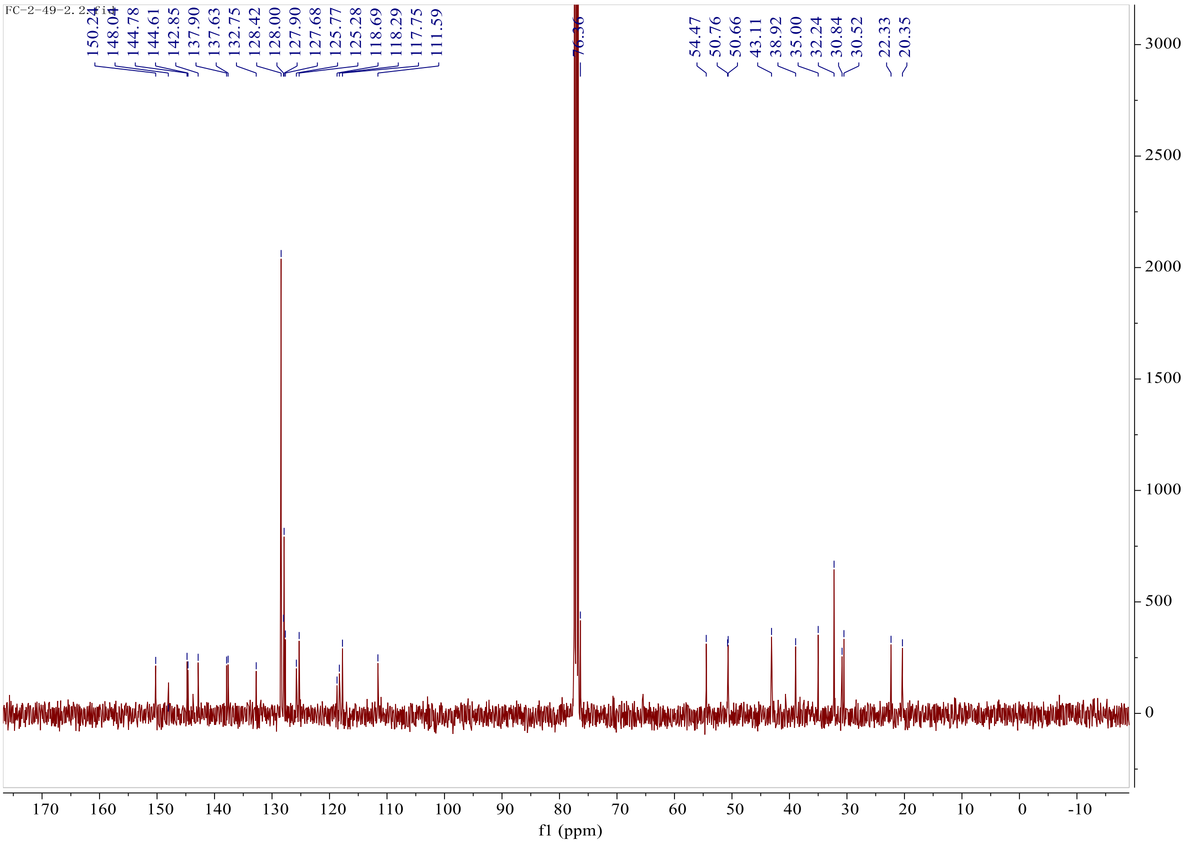

FC-2-49-2.2.fid
150.24
148.04
144.78
144.61
142.85
137.90
137.63
132.75
128.42
128.00
127.90
127.68
125.77
125.28
118.69
118.29
117.75
111.59
76.36
54.47
50.76
50.66
43.11
38.92
35.00
32.24
30.84
30.52
22.33
20.35
f1 (ppm)

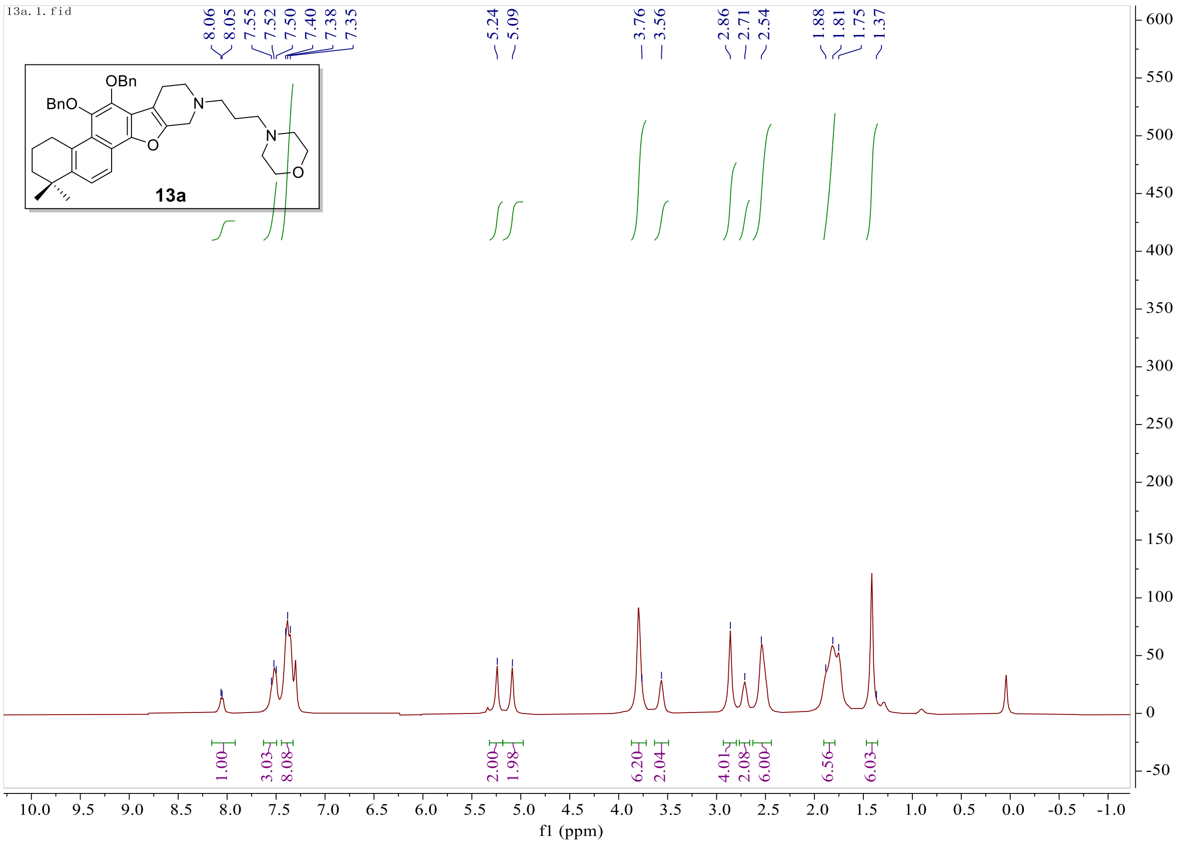
13a. 1. fid
8.06
8.05
7.55
7.52
7.50
7.40
7.38
7.35
5.24
5.09
3.76
3.56
2.86
2.71
2.54
1.88
1.81
1.75
1.37
OBn
BnO
N
N
O
O
13a
1.00
3.03
8.08
2.00
1.98
6.20
2.04
4.01
2.08
6.00
6.56
6.03
f1 (ppm)

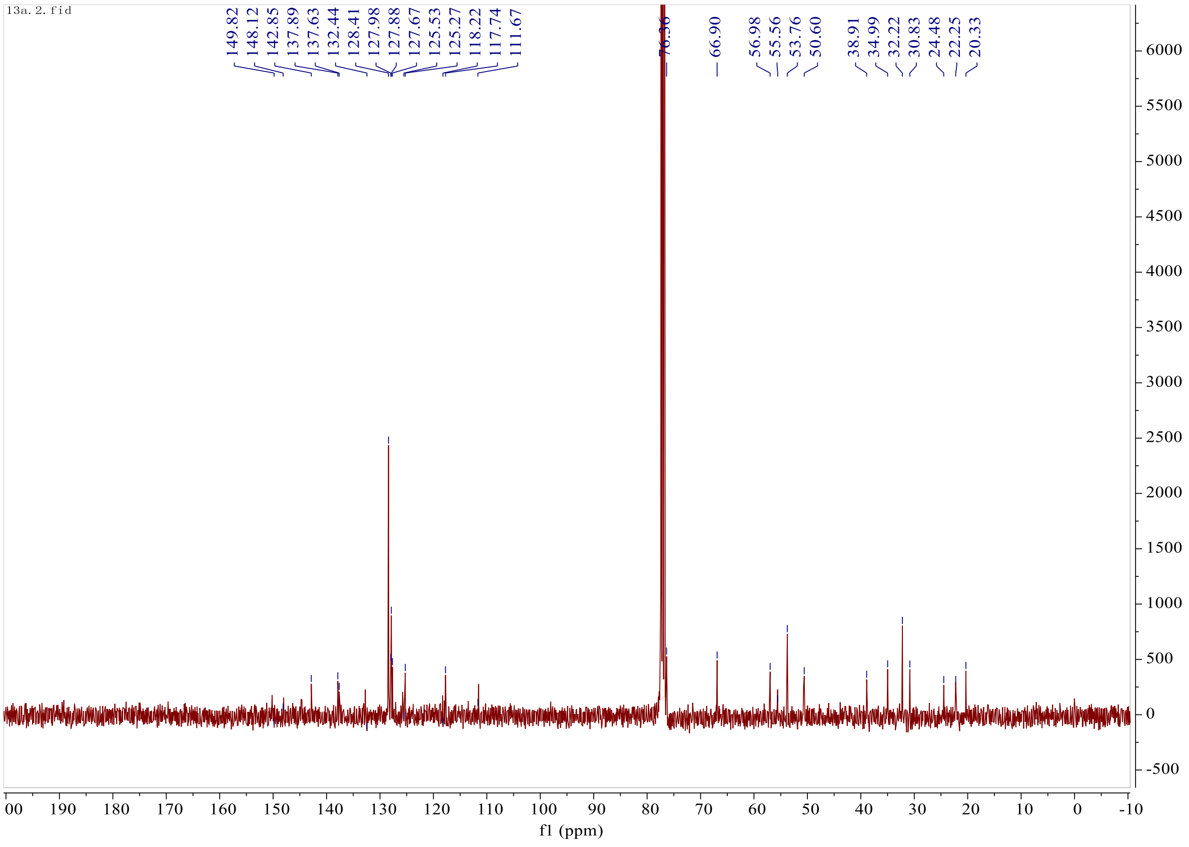
13a. 2. fid
149.82
148.12
142.85
137.89
137.63
132.44
128.41
127.98
127.88
127.67
125.53
125.27
118.22
117.74
111.67
66.90
56.98
55.56
53.76
50.60
38.91
34.99
32.22
30.83
24.48
22.25
20.33
f1 (ppm)

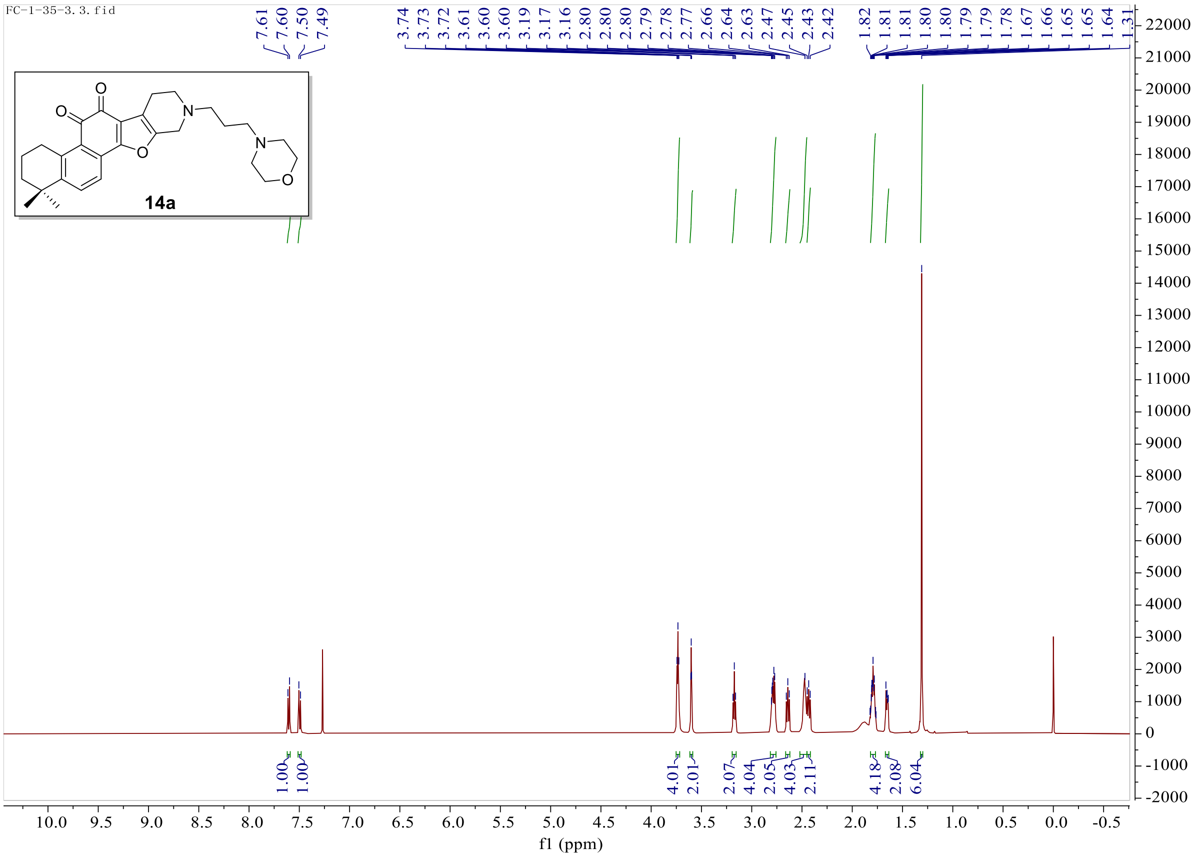
FC-1-35-3.3.fid
7.61
7.60
7.50
7.49
3.74
3.73
3.72
3.61
3.60
3.60
3.19
3.17
3.16
2.80
2.80
2.80
2.79
2.78
2.77
2.66
2.64
2.63
2.47
2.45
2.43
2.42
1.82
1.81
1.81
1.80
1.80
1.79
1.79
1.78
1.67
1.66
1.65
1.64
1.31
14a
f1 (ppm)

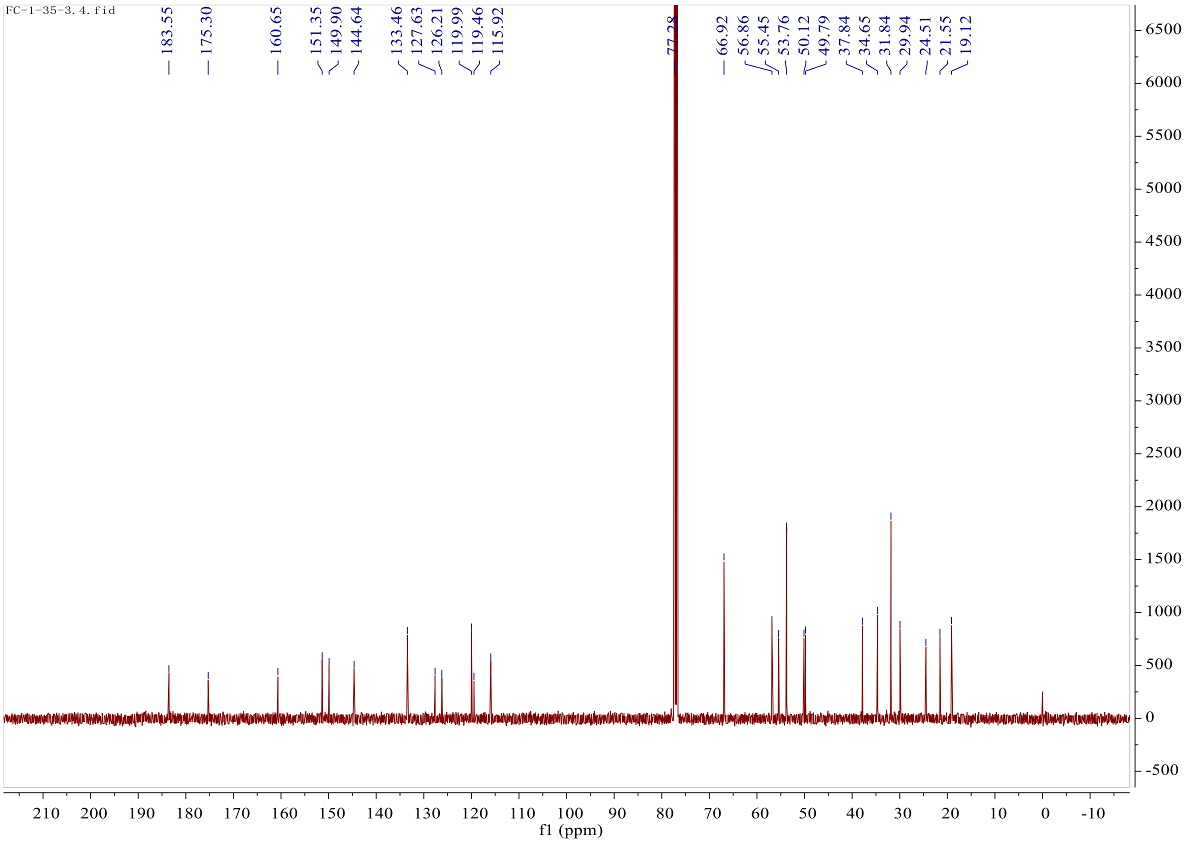
FC-1-35-3.4.fid
183.55
175.30
160.65
151.35
149.90
144.64
133.46
127.63
126.21
119.99
119.46
115.92
77.28
66.92
56.86
55.45
53.76
50.12
49.79
37.84
34.65
31.84
29.94
24.51
21.55
19.12
f1 (ppm)

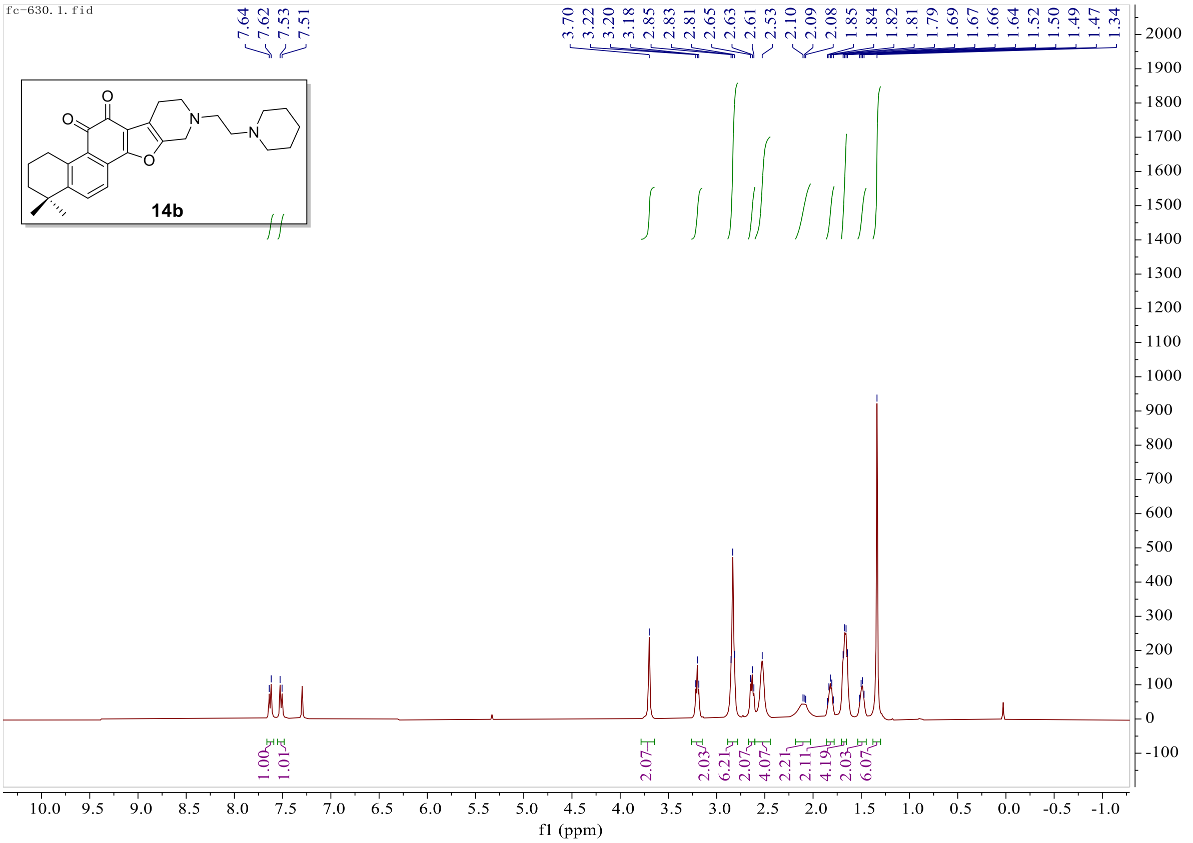
fc-630.1.fid
7.64
7.62
7.53
7.51
3.70
3.22
3.20
3.18
2.85
2.83
2.81
2.65
2.63
2.61
2.53
2.10
2.09
2.08
1.85
1.84
1.82
1.81
1.79
1.69
1.67
1.66
1.64
1.52
1.50
1.49
1.47
1.34
14b
1.00
1.01
2.07
2.03
6.21
2.07
4.07
2.21
2.11
4.19
2.03
6.07
f1 (ppm)

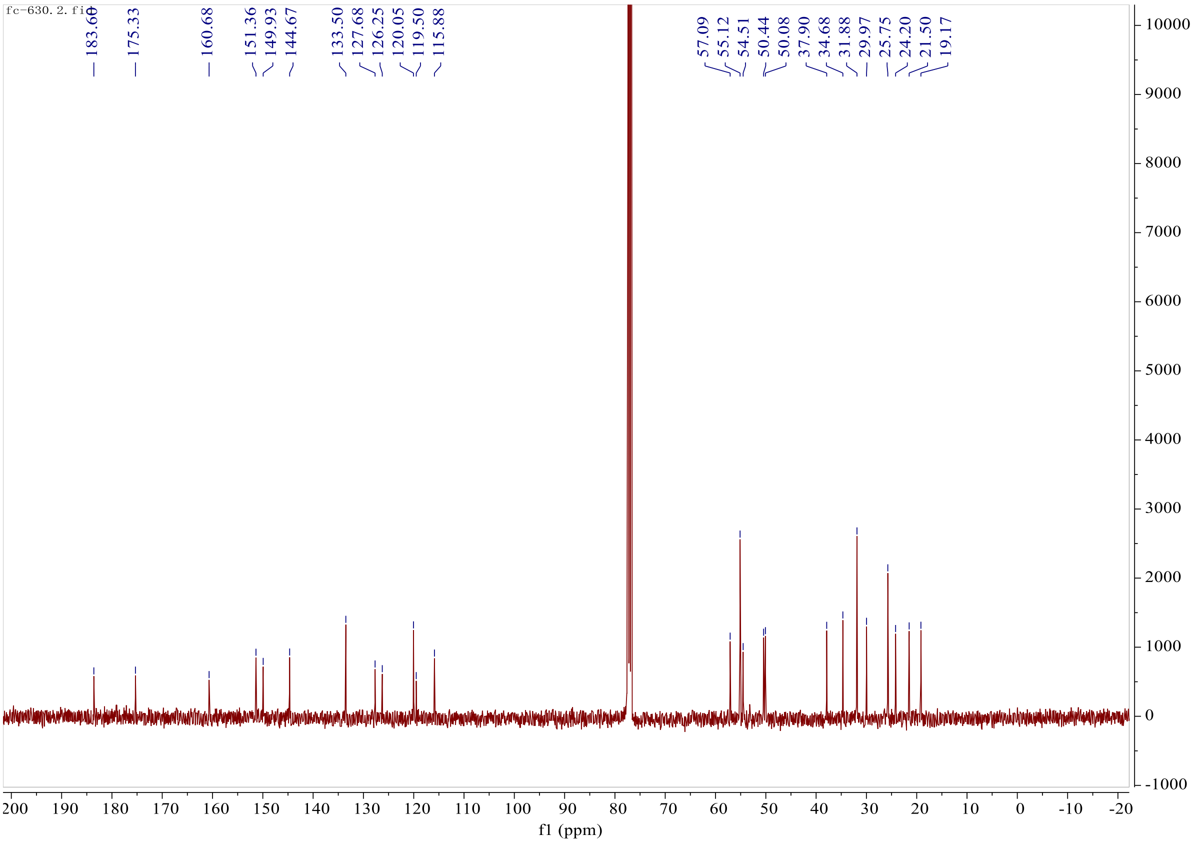
fc-630.2.fid
183.60
175.33
160.68
151.36
149.93
144.67
133.50
127.68
126.25
120.05
119.50
115.88
57.09
55.12
54.51
50.44
50.08
37.90
34.68
31.88
29.97
25.75
24.20
21.50
19.17
f1 (ppm)

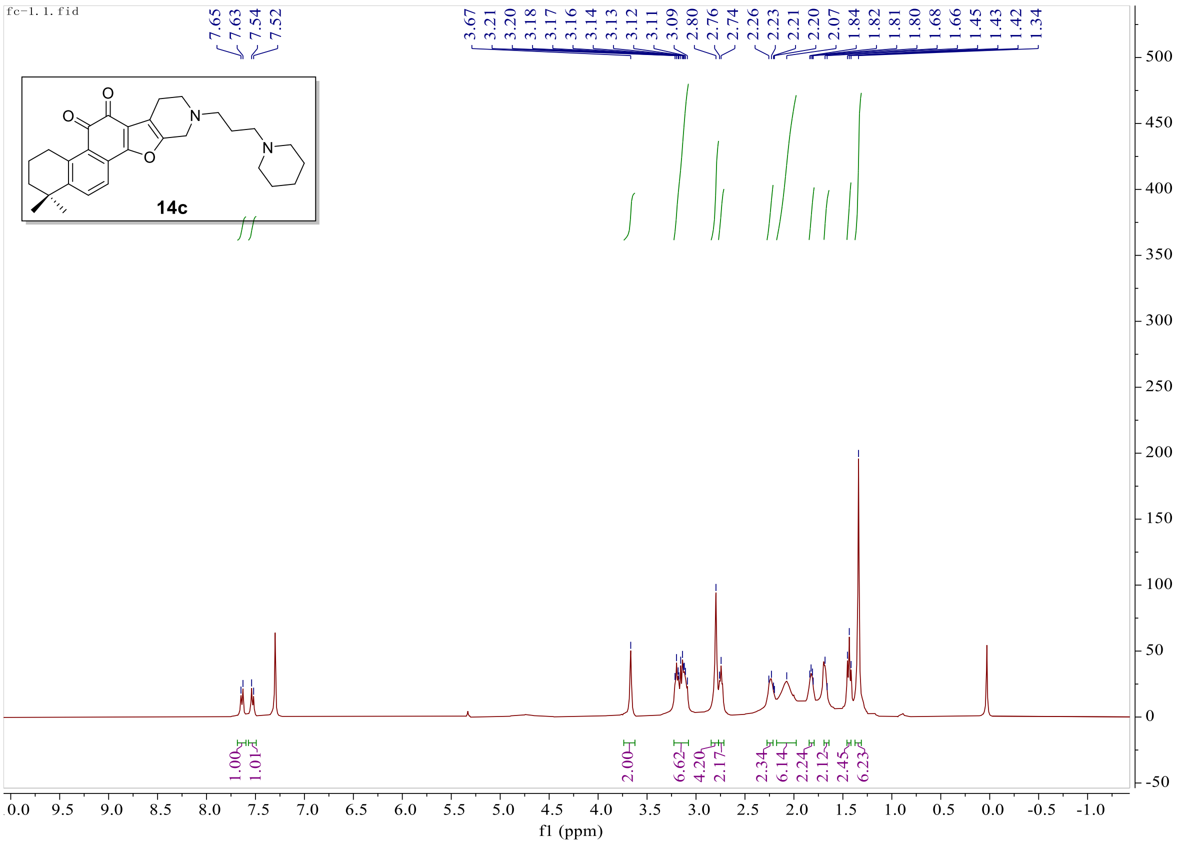
fc-1.1.fid
7.65
7.63
7.54
7.52
3.67
3.21
3.20
3.18
3.17
3.16
3.14
3.13
3.12
3.11
3.09
2.80
2.76
2.74
2.26
2.23
2.21
2.20
2.07
1.84
1.82
1.81
1.80
1.68
1.66
1.45
1.43
1.42
1.34
14c
1.00
1.01
2.00
6.62
4.20
2.17
2.34
6.14
2.24
2.12
2.45
6.23
f1 (ppm)

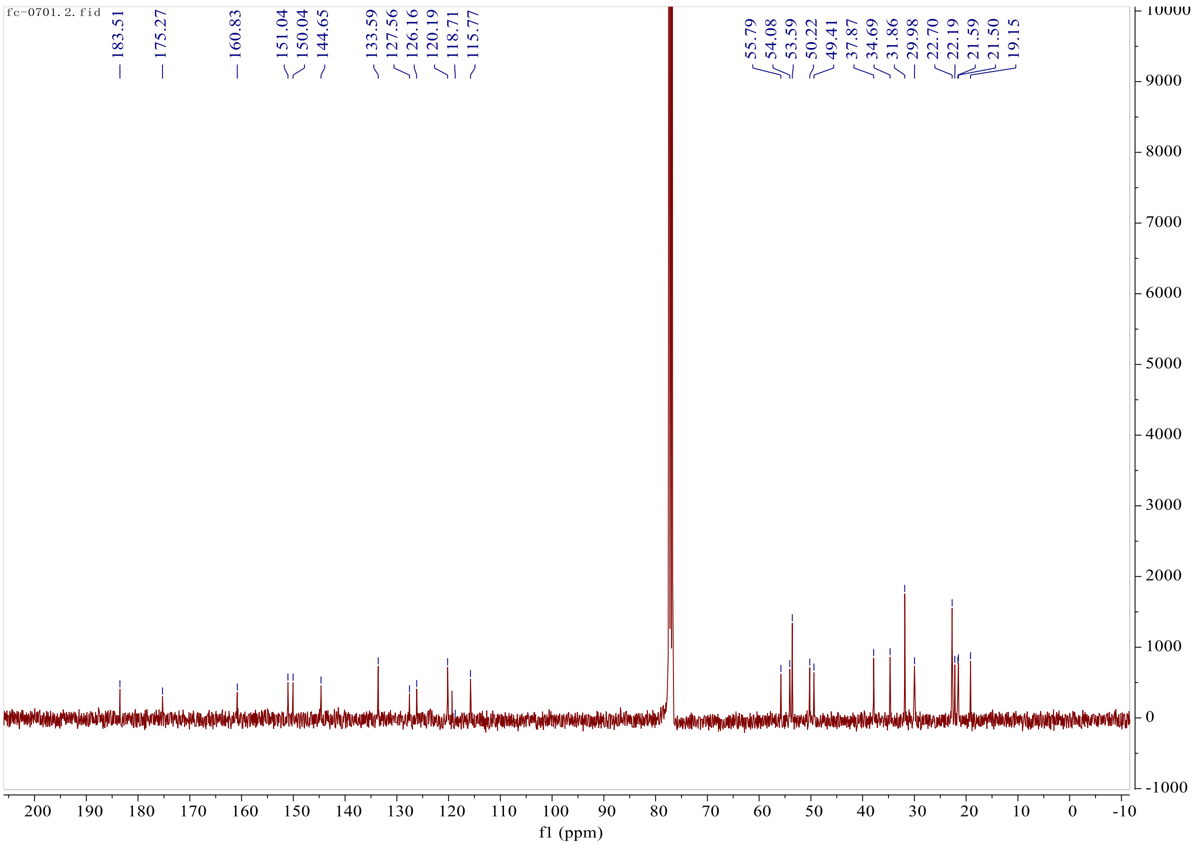
fc-0701.2.fid
183.51
175.27
160.83
151.04
150.04
144.65
133.59
127.56
126.16
120.19
118.71
115.77
55.79
54.08
53.59
50.22
49.41
37.87
34.69
31.86
29.98
22.70
22.19
21.59
21.50
19.15
f1 (ppm)

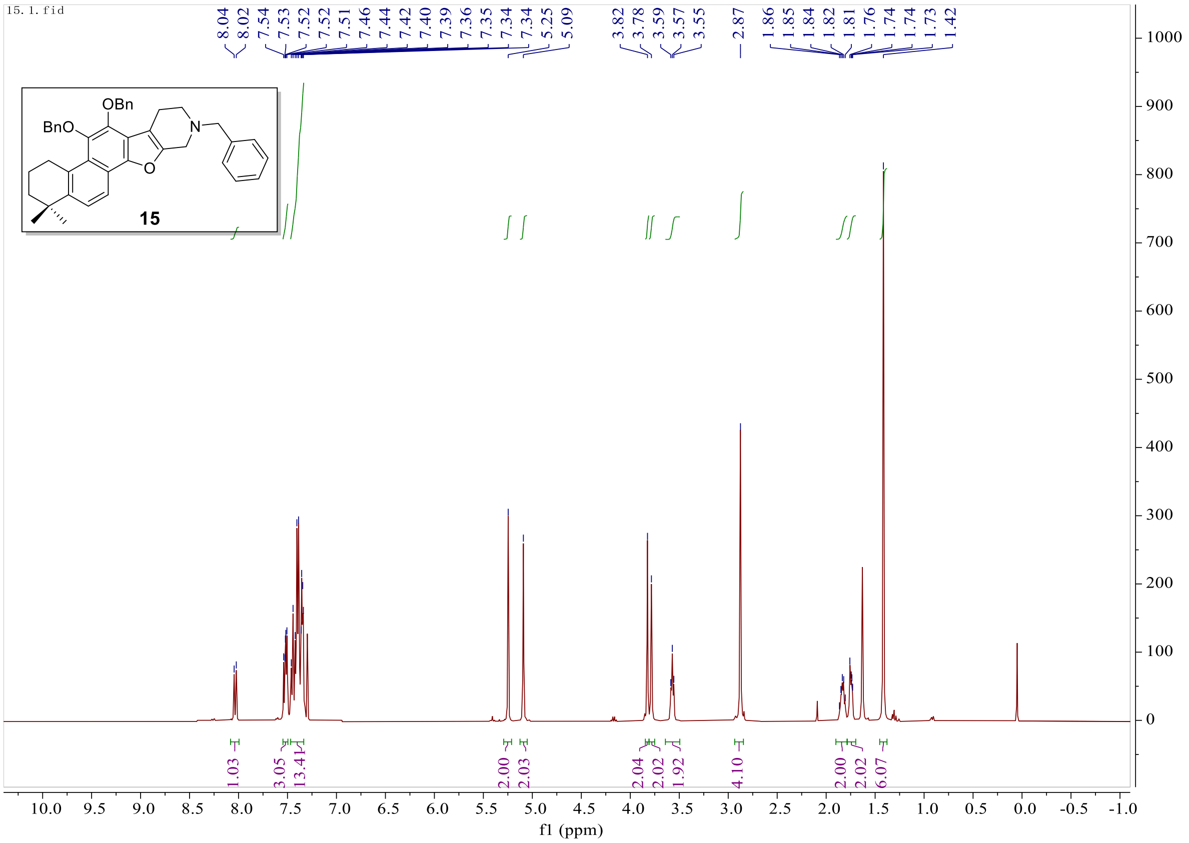

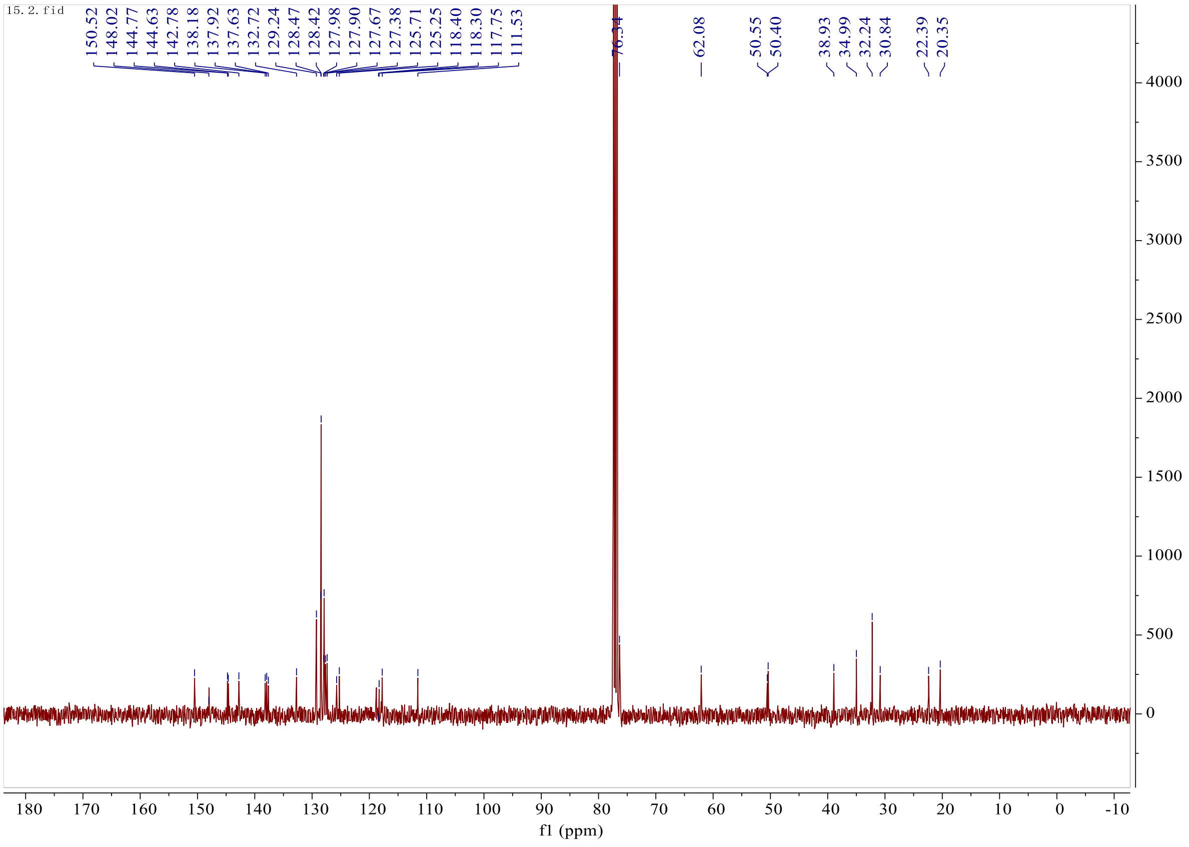

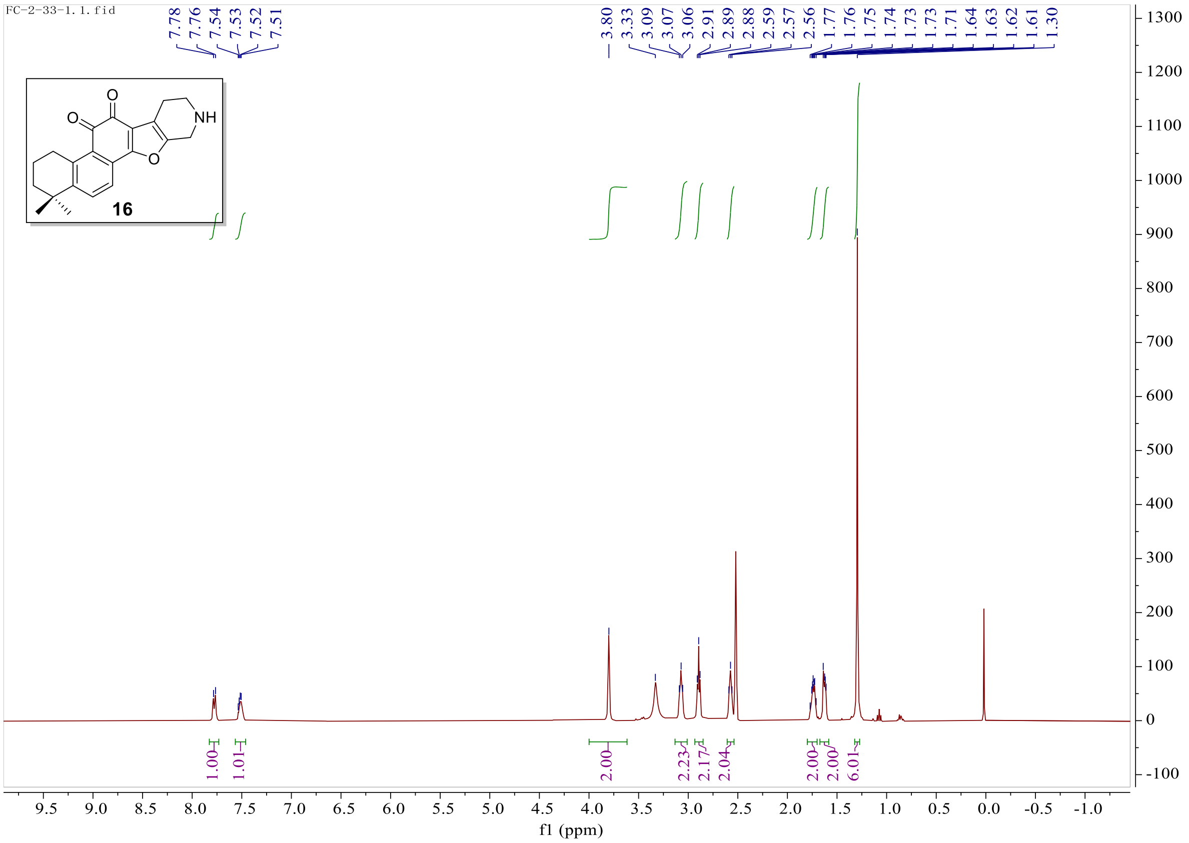
FC-2-33-1.1.fid
7.78
7.76
7.54
7.53
7.52
7.51
3.80
3.33
3.09
3.07
3.06
2.91
2.89
2.88
2.59
2.57
2.56
1.77
1.76
1.75
1.74
1.73
1.73
1.71
1.64
1.63
1.62
1.61
1.30
NH
16
1.00
1.01
2.00
2.23
2.17
2.04
2.00
2.00
6.01
f1 (ppm)

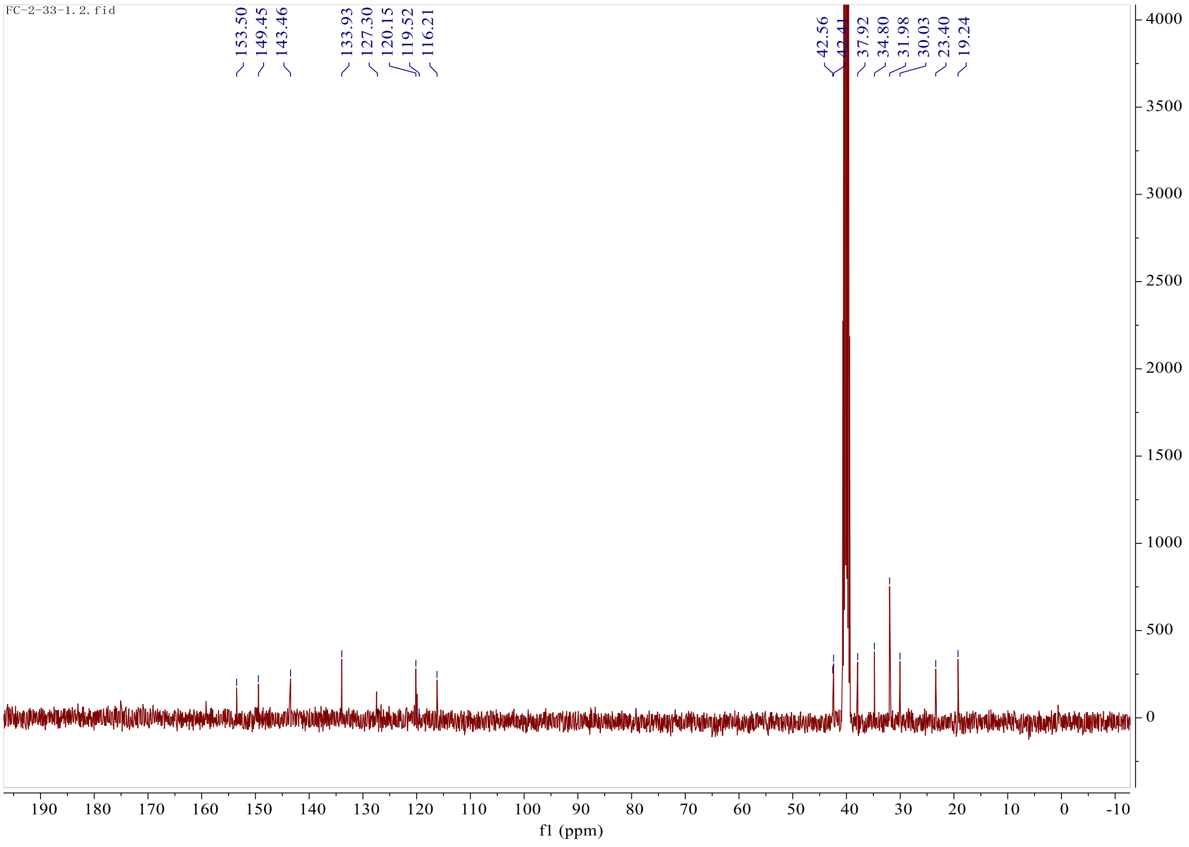
FC-2-33-1.2.fid
153.50
149.45
143.46
133.93
127.30
120.15
119.52
116.21
42.56
42.41
37.92
34.80
31.98
30.03
23.40
19.24
f1 (ppm)

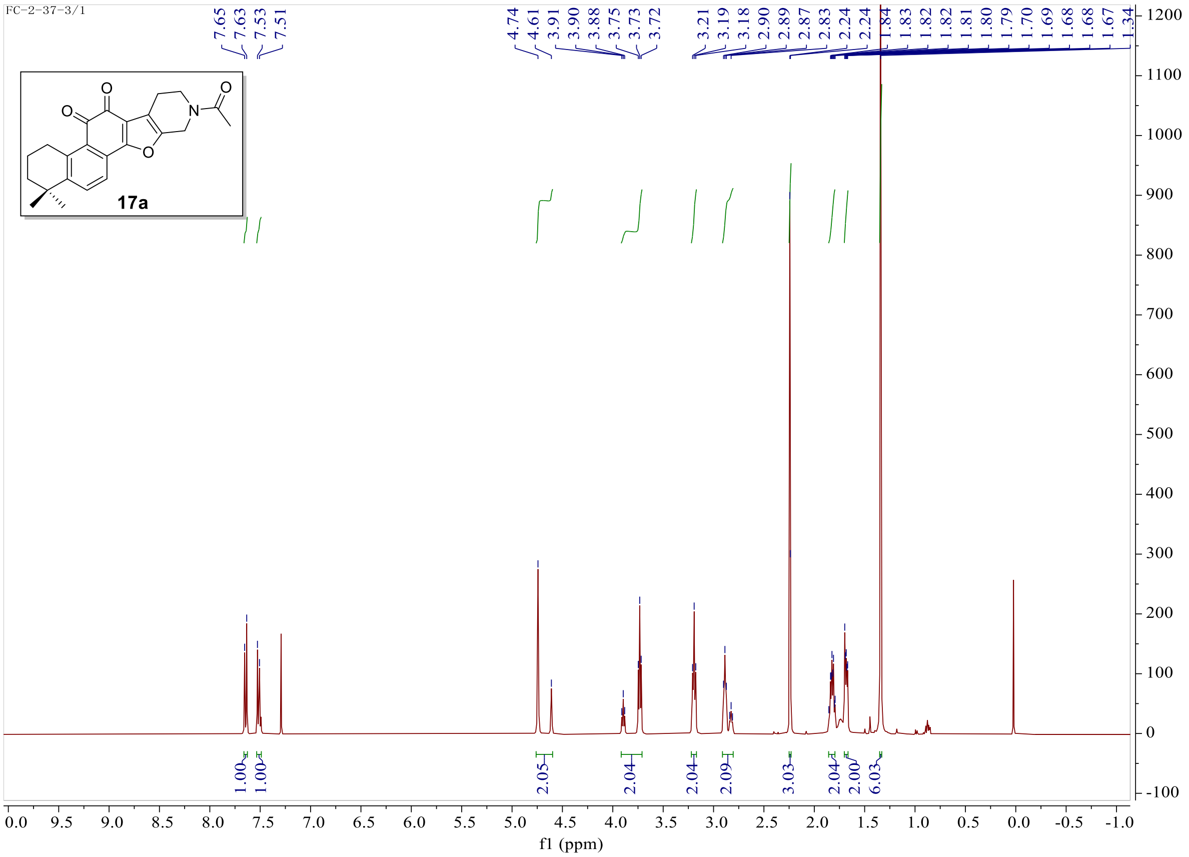
FC-2-37-3/1
7.65
7.63
7.53
7.51
4.74
4.61
3.91
3.90
3.88
3.75
3.73
3.72
3.21
3.19
3.18
2.90
2.89
2.87
2.83
2.24
2.24
1.84
1.83
1.82
1.82
1.81
1.80
1.79
1.70
1.69
1.68
1.68
1.67
1.34
17a
1.00
1.00
2.05
2.04
2.04
2.09
3.03
2.04
2.00
6.03
f1 (ppm)

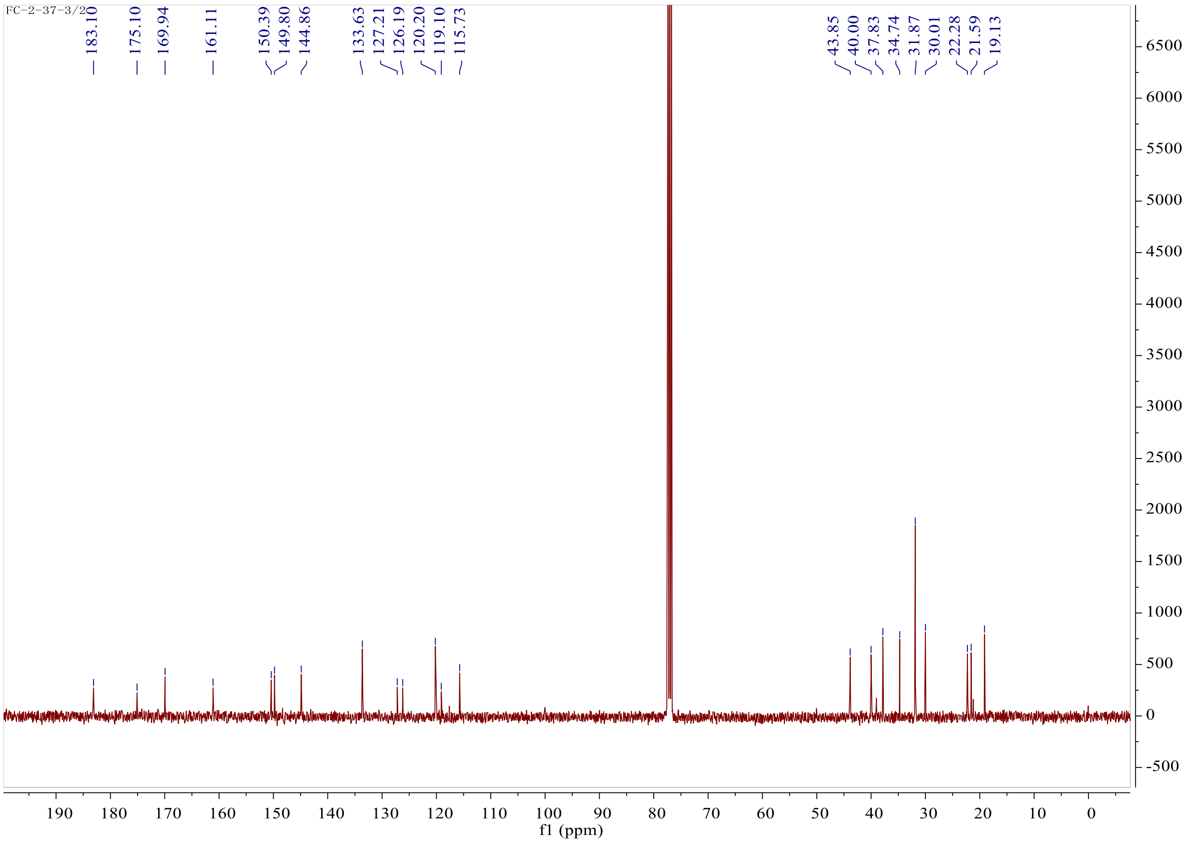
FC-2-37-3/2
183.10
175.10
169.94
161.11
150.39
149.80
144.86
133.63
127.21
126.19
120.20
119.10
115.73
43.85
40.00
37.83
34.74
31.87
30.01
22.28
21.59
19.13
f1 (ppm)

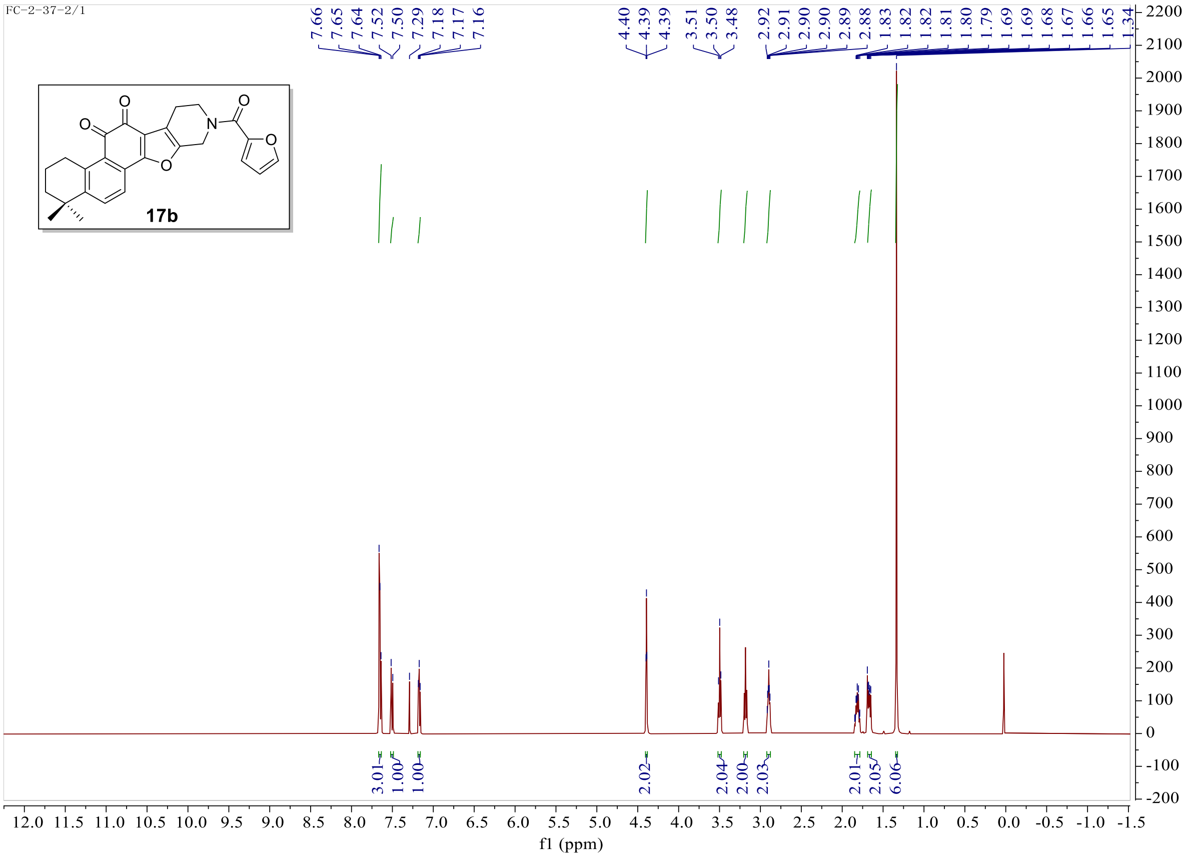
FC-2-37-2/1
7.66 7.65 7.64 7.52 7.50 7.29 7.18 7.17 7.16
4.40 4.39 4.39 3.51 3.50 3.48
2.92 2.91 2.90 2.90 2.89 2.88 1.83 1.82 1.82 1.81 1.80 1.79 1.69 1.69 1.68 1.67 1.66 1.65 1.34
17b
3.01 1.00 1.00 2.02 2.04 2.00 2.03 2.01 2.05 6.06
f1 (ppm)

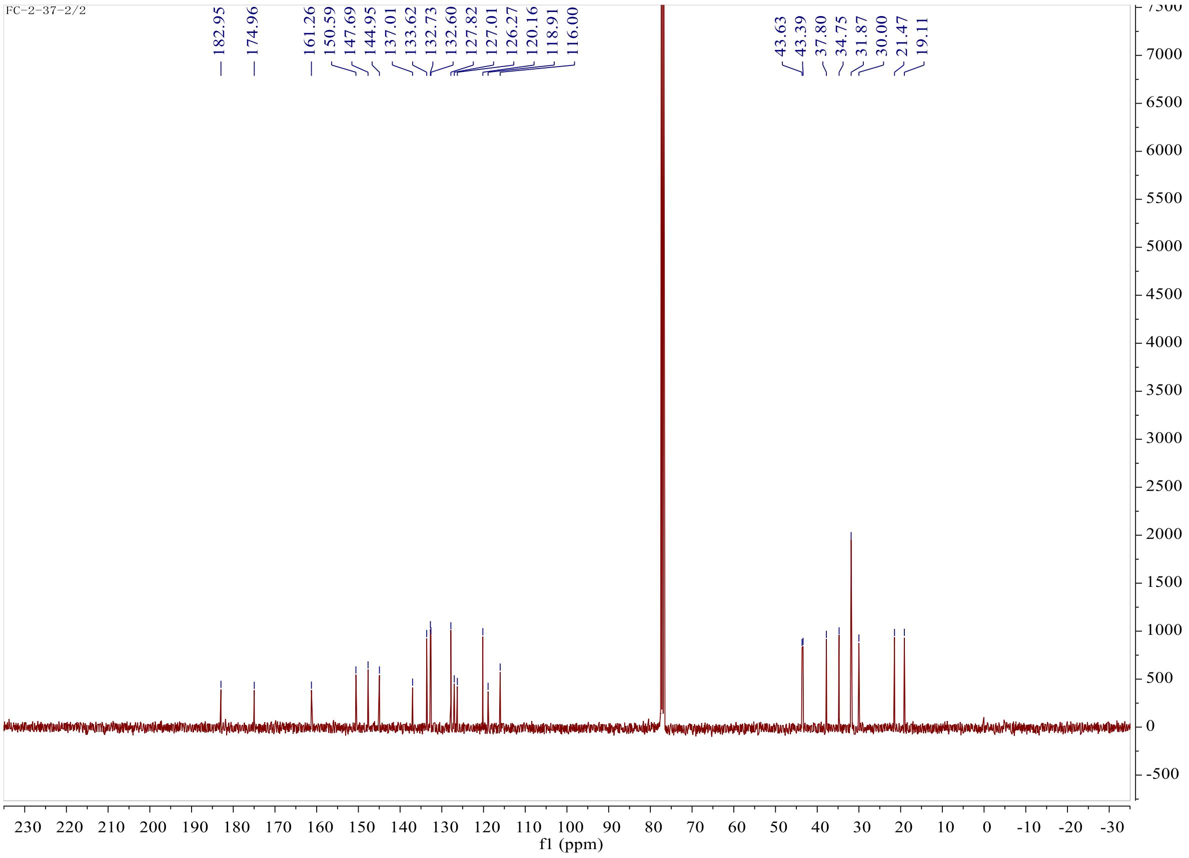
FC-2-37-2/2
182.95 174.96 161.26 150.59 147.69 144.95 137.01 133.62 132.73 132.60 127.82 127.01 126.27 120.16 118.91 116.00
43.63 43.39 37.80 34.75 31.87 30.00 21.47 19.11
f1 (ppm)

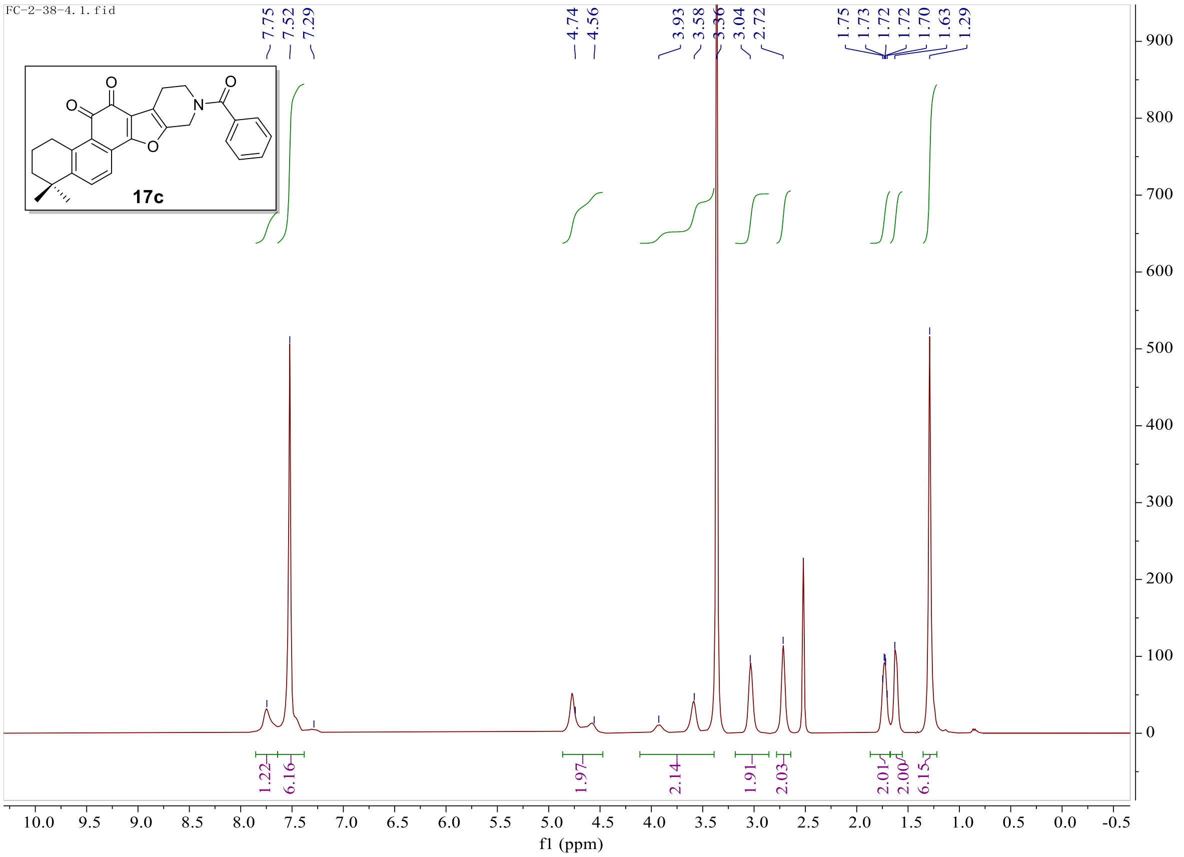
FC-2-38-4.1.fid
7.75
7.52
7.29
4.74
4.56
3.93
3.58
3.36
3.04
2.72
1.75
1.73
1.72
1.72
1.70
1.63
1.29
17c
1.22
6.16
1.97
2.14
1.91
2.03
2.01
2.00
6.15
f1 (ppm)

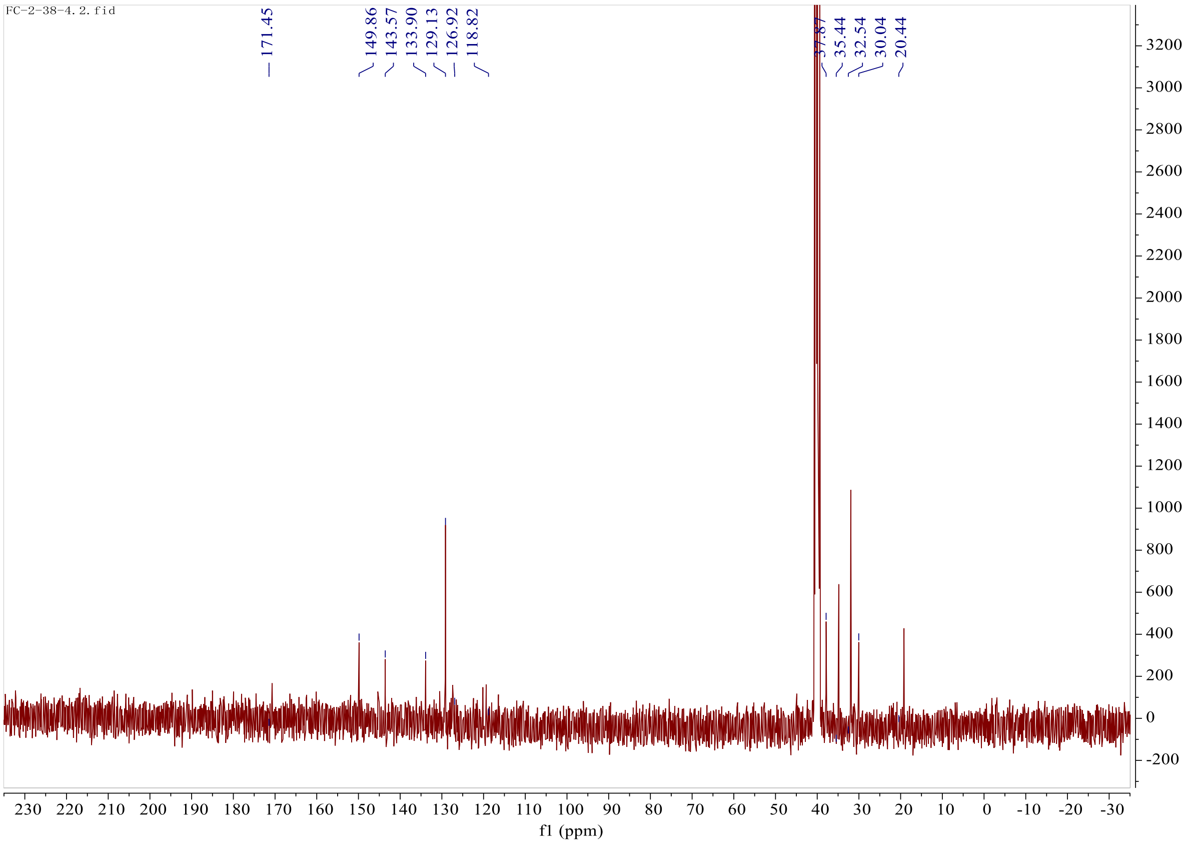
FC-2-38-4.2.fid
171.45
149.86
143.57
133.90
129.13
126.92
118.82
37.87
35.44
32.54
30.04
20.44
f1 (ppm)

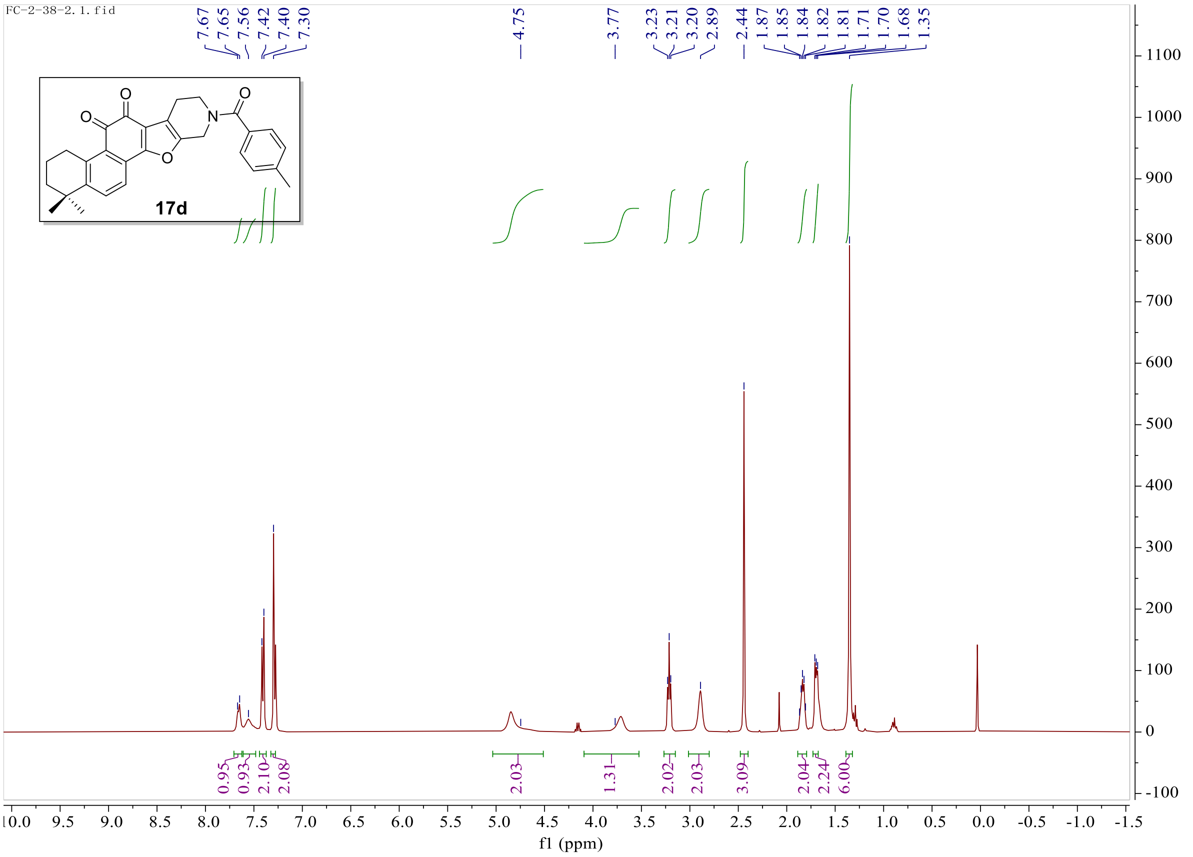
FC-2-38-2.1.fid
7.67
7.65
7.56
7.42
7.40
7.30
4.75
3.77
3.23
3.21
3.20
2.89
2.44
1.87
1.85
1.84
1.82
1.81
1.71
1.70
1.68
1.35
17d
0.95
0.93
2.10
2.08
2.03
1.31
2.02
2.03
3.09
2.04
2.24
6.00
f1 (ppm)

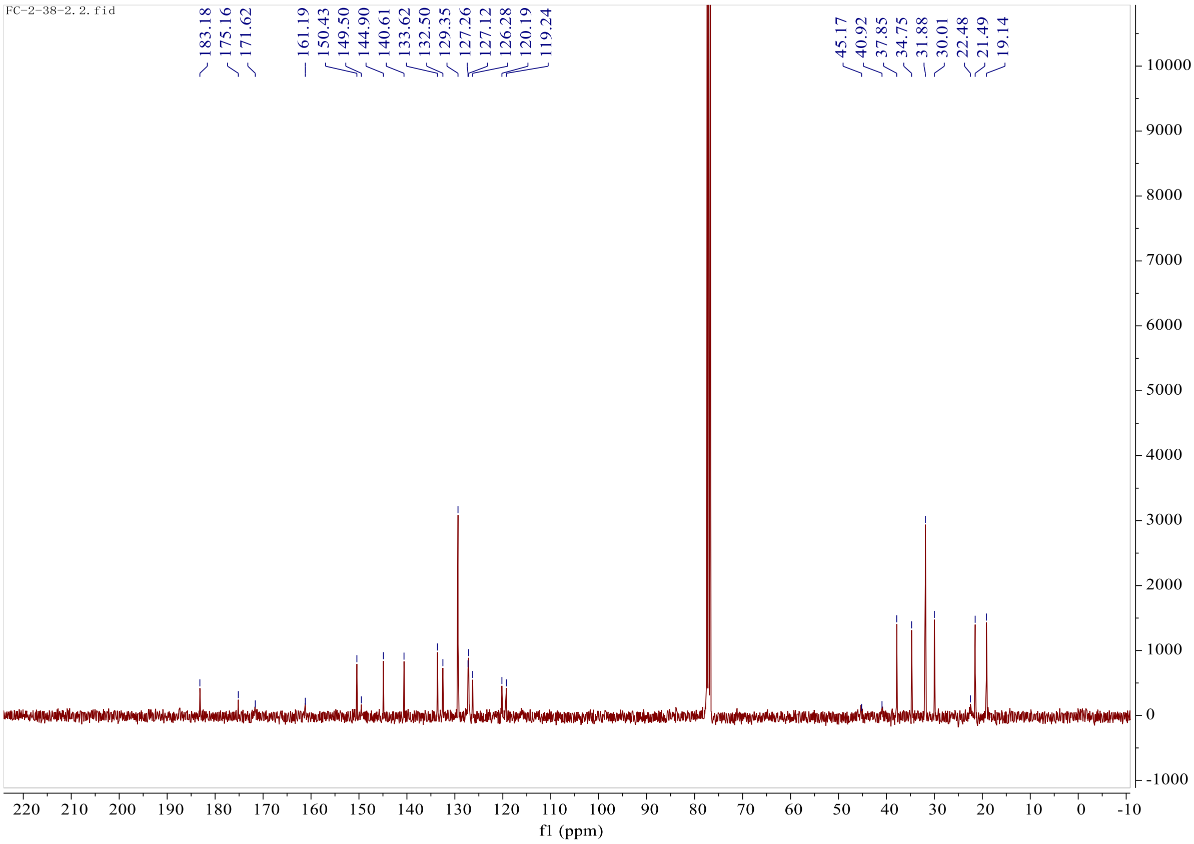
FC-2-38-2.2.fid
183.18
175.16
171.62
161.19
150.43
149.50
144.90
140.61
133.62
132.50
129.35
127.26
127.12
126.28
120.19
119.24
45.17
40.92
37.85
34.75
31.88
30.01
22.48
21.49
19.14
f1 (ppm)

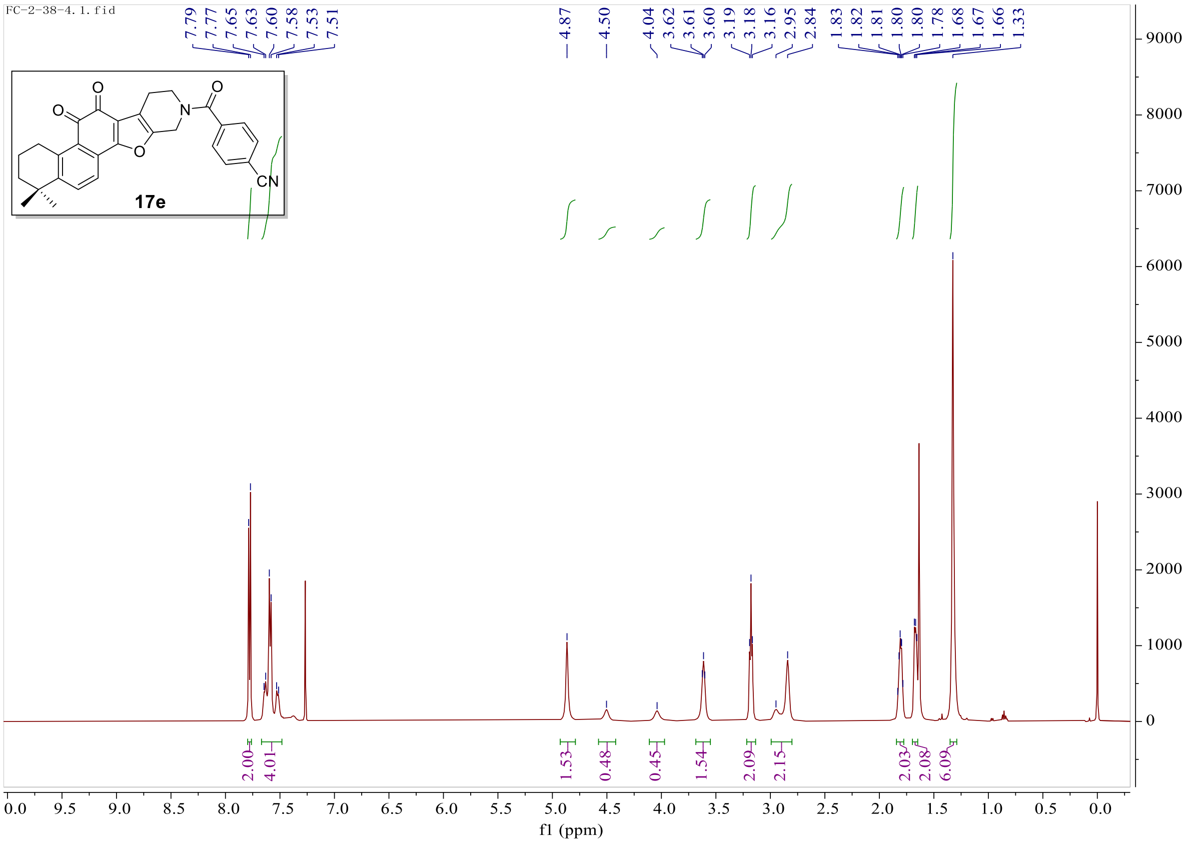
FC-2-38-4.1.fid
7.79
7.77
7.65
7.63
7.60
7.58
7.53
7.51
4.87
4.50
4.04
3.62
3.61
3.60
3.19
3.18
3.16
2.95
2.84
1.83
1.82
1.81
1.80
1.80
1.78
1.68
1.67
1.66
1.33
17e
2.00
4.01
1.53
0.48
0.45
1.54
2.09
2.15
2.03
2.08
6.09
f1 (ppm)

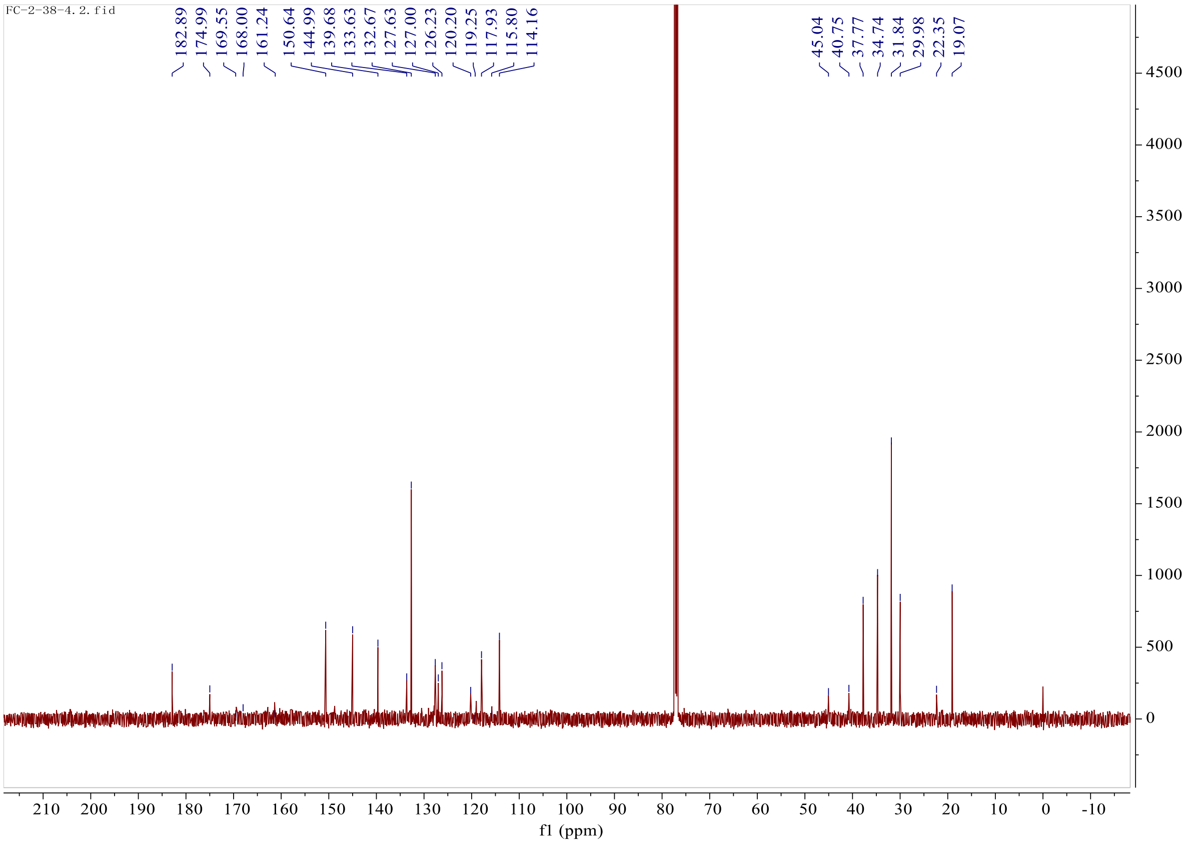
FC-2-38-4.2.fid
182.89
174.99
169.55
168.00
161.24
150.64
144.99
139.68
133.63
132.67
127.63
127.00
126.23
120.20
119.25
117.93
115.80
114.16
45.04
40.75
37.77
34.74
31.84
29.98
22.35
19.07
f1 (ppm)

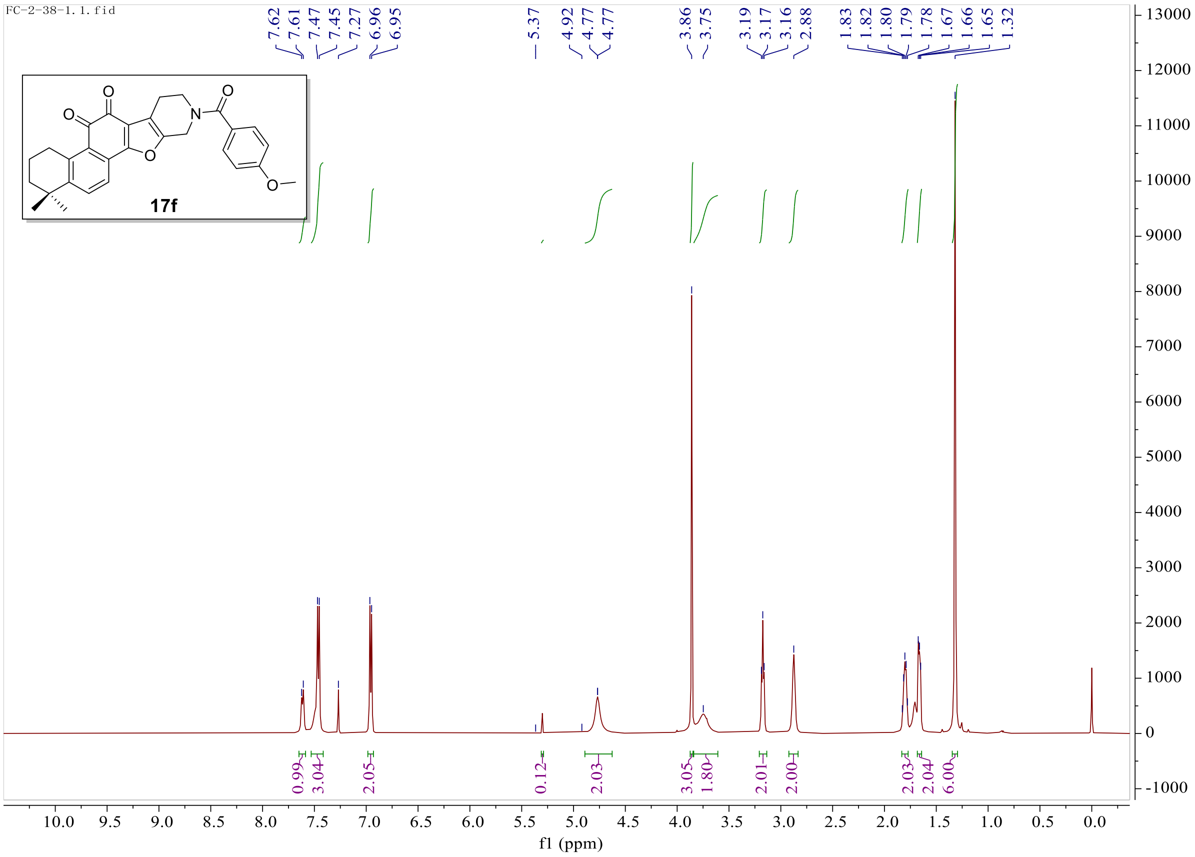
FC-2-38-1.1.fid
7.62
7.61
7.47
7.45
7.27
6.96
6.95
5.37
4.92
4.77
4.77
3.86
3.75
3.19
3.17
3.16
2.88
1.83
1.82
1.80
1.79
1.78
1.67
1.66
1.65
1.32
17f
0.99
3.04
2.05
0.12
2.03
3.05
1.80
2.01
2.00
2.03
2.04
6.00
f1 (ppm)

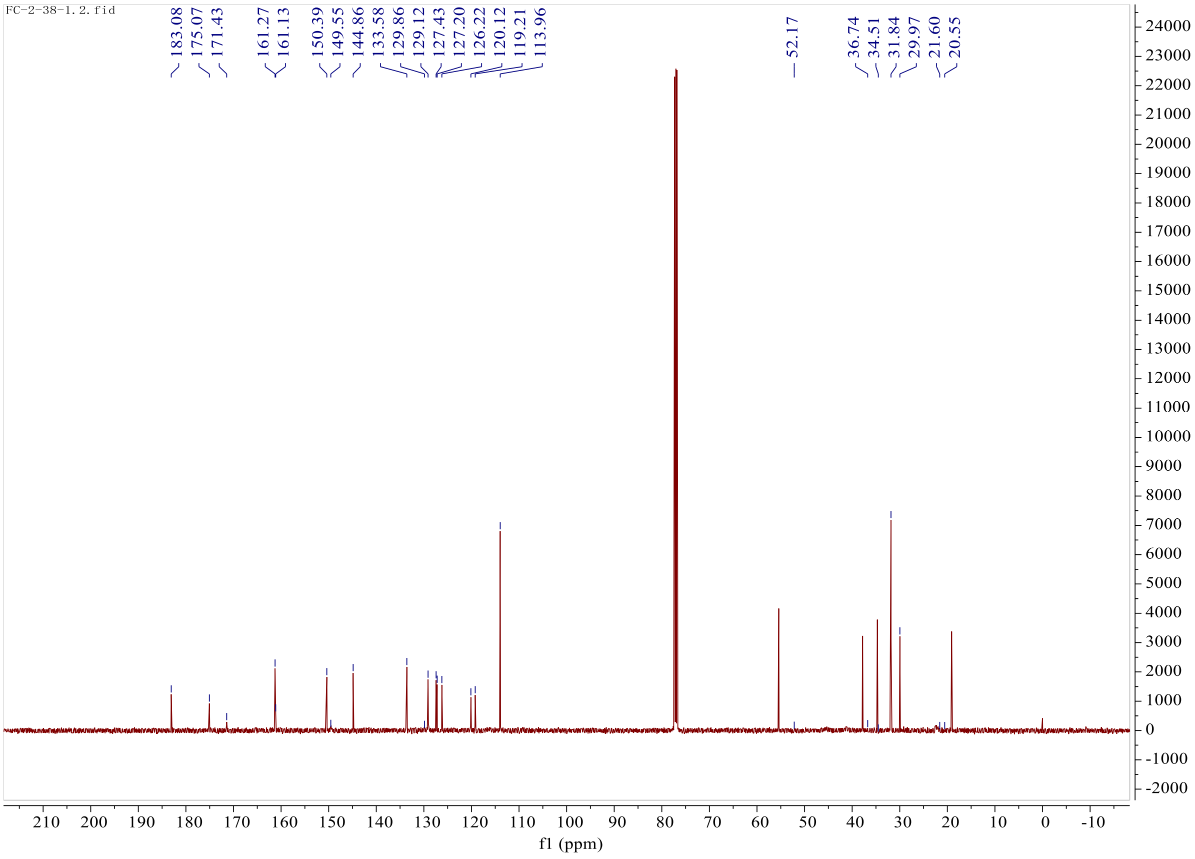
FC-2-38-1.2.fid
183.08
175.07
171.43
161.27
161.13
150.39
149.55
144.86
133.58
129.86
129.12
127.43
127.20
126.22
120.12
119.21
113.96
52.17
36.74
34.51
31.84
29.97
21.60
20.55
f1 (ppm)

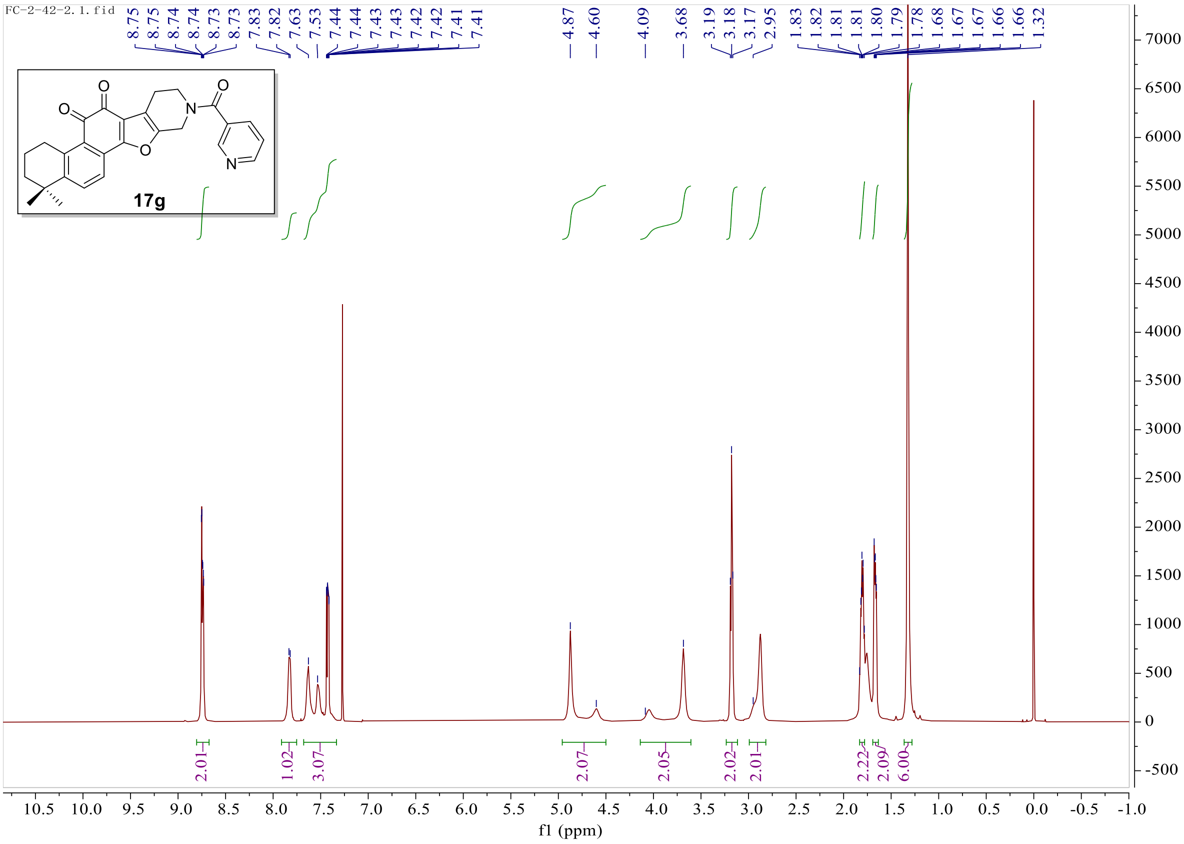
FC-2-42-2.1.fid
8.75 8.75 8.74 8.74 8.73 8.73 7.83 7.82 7.63 7.53 7.44 7.44 7.43 7.43 7.42 7.42 7.41 7.41
4.87 4.60 4.09 3.68 3.19 3.18 3.17 2.95
1.83 1.82 1.81 1.81 1.80 1.79 1.78 1.68 1.67 1.67 1.66 1.66 1.32
17g
2.01 1.02 3.07 2.07 2.05 2.02 2.01 2.22 2.09 6.00
f1 (ppm)

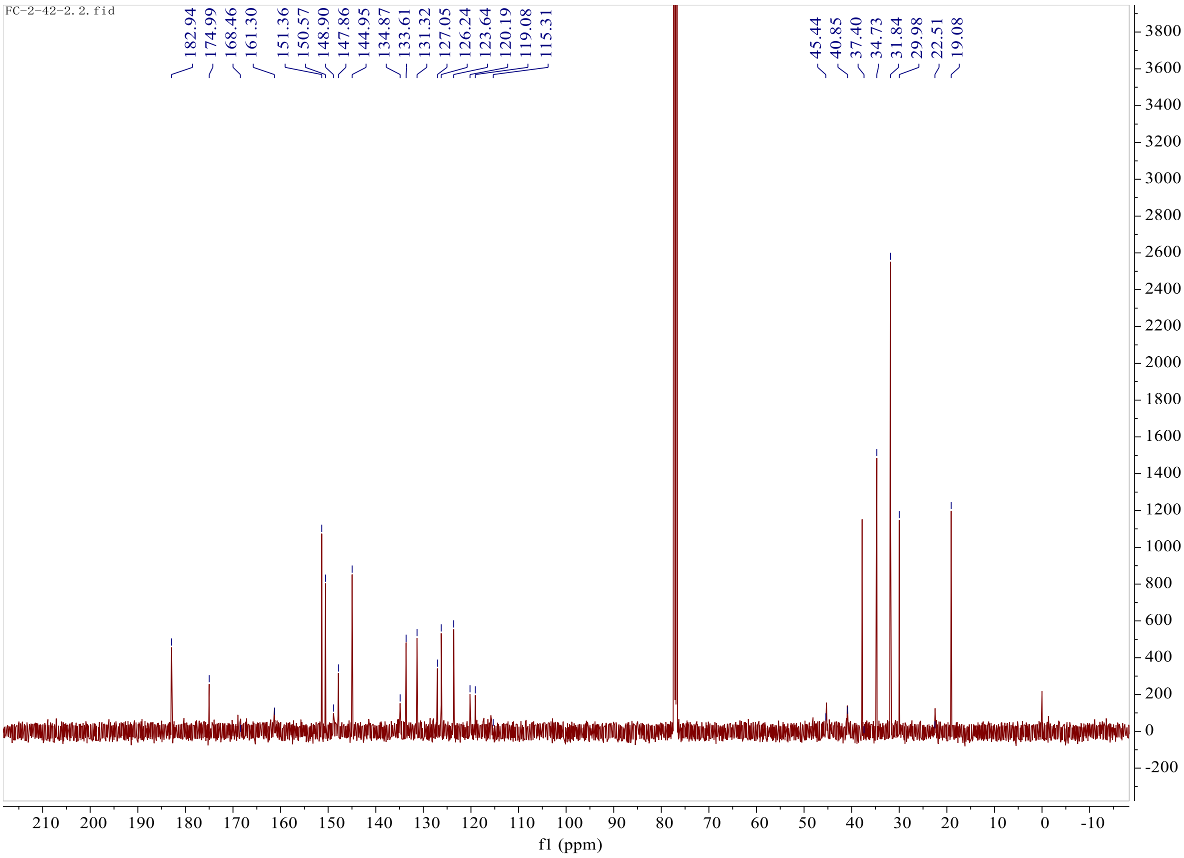
FC-2-42-2.2.fid
182.94 174.99 168.46 161.30 151.36 150.57 148.90 147.86 144.95 134.87 133.61 131.32 127.05 126.24 123.64 120.19 119.08 115.31
45.44 40.85 37.40 34.73 31.84 29.98 22.51 19.08
f1 (ppm)

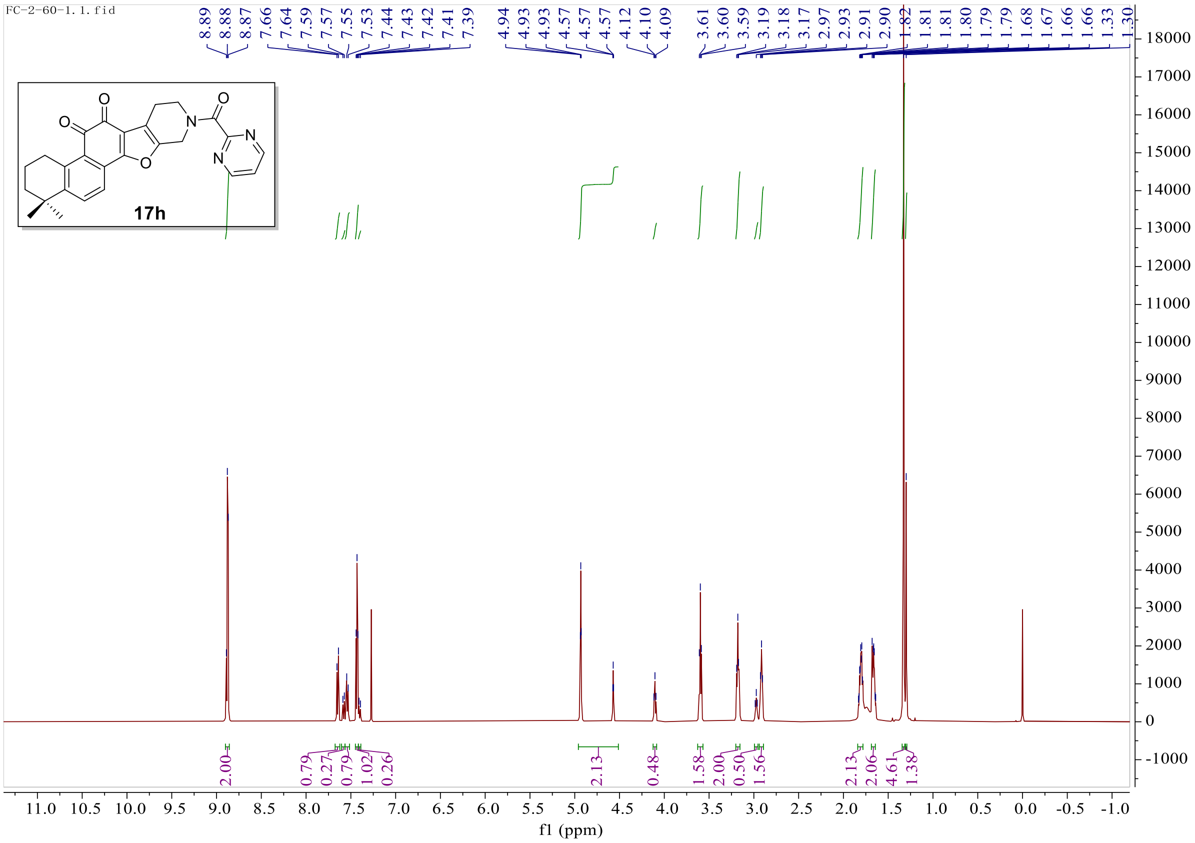
FC-2-60-1.1.fid
8.89
8.88
8.87
7.66
7.64
7.59
7.57
7.55
7.53
7.44
7.43
7.42
7.41
7.39
4.94
4.93
4.93
4.57
4.57
4.57
4.12
4.10
4.09
3.61
3.60
3.59
3.19
3.18
3.17
2.97
2.93
2.91
2.90
1.82
1.81
1.81
1.80
1.79
1.79
1.68
1.67
1.66
1.66
1.33
1.30
17h
2.00
0.79
0.27
0.79
1.02
0.26
2.13
0.48
1.58
2.00
0.50
1.56
2.13
2.06
4.61
1.38
f1 (ppm)

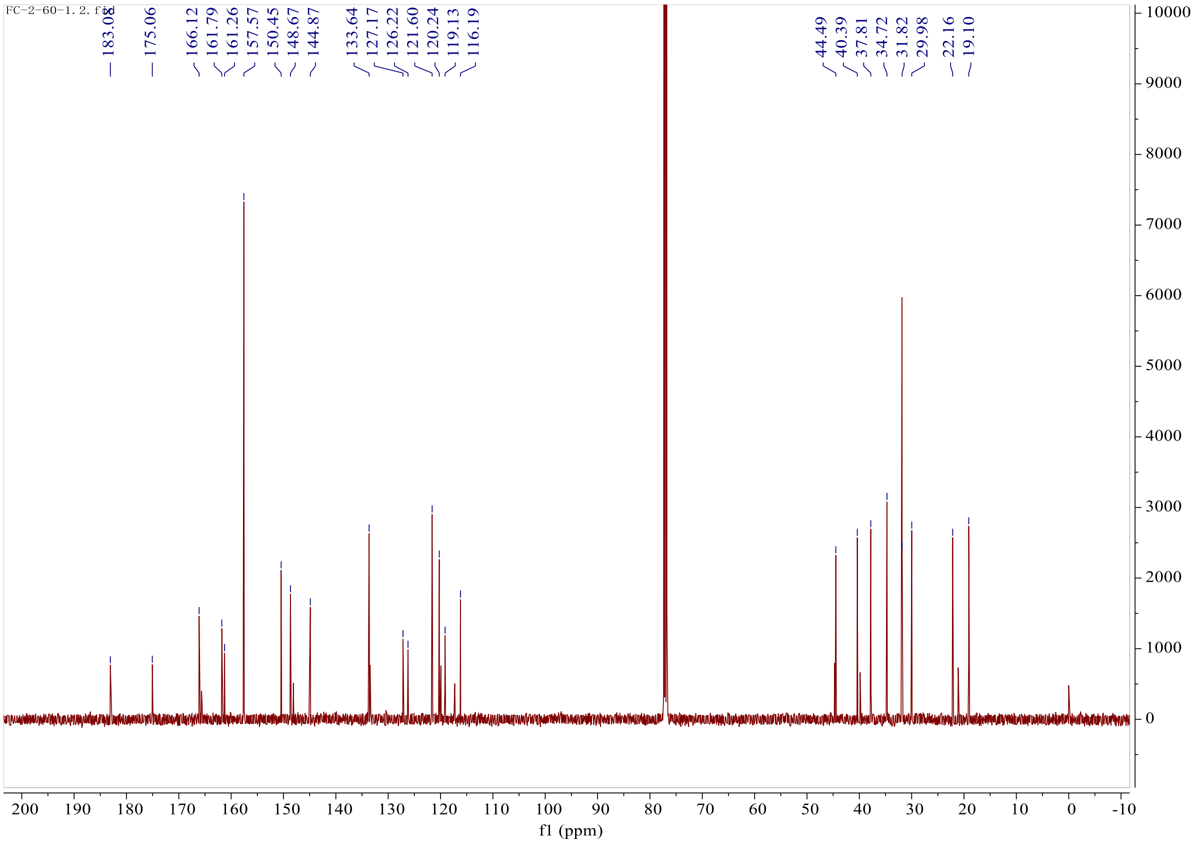
FC-2-60-1.2.fid
183.08
175.06
166.12
161.79
161.26
157.57
150.45
148.67
144.87
133.64
127.17
126.22
121.60
120.24
119.13
116.19
44.49
40.39
37.81
34.72
31.82
29.98
22.16
19.10
f1 (ppm)

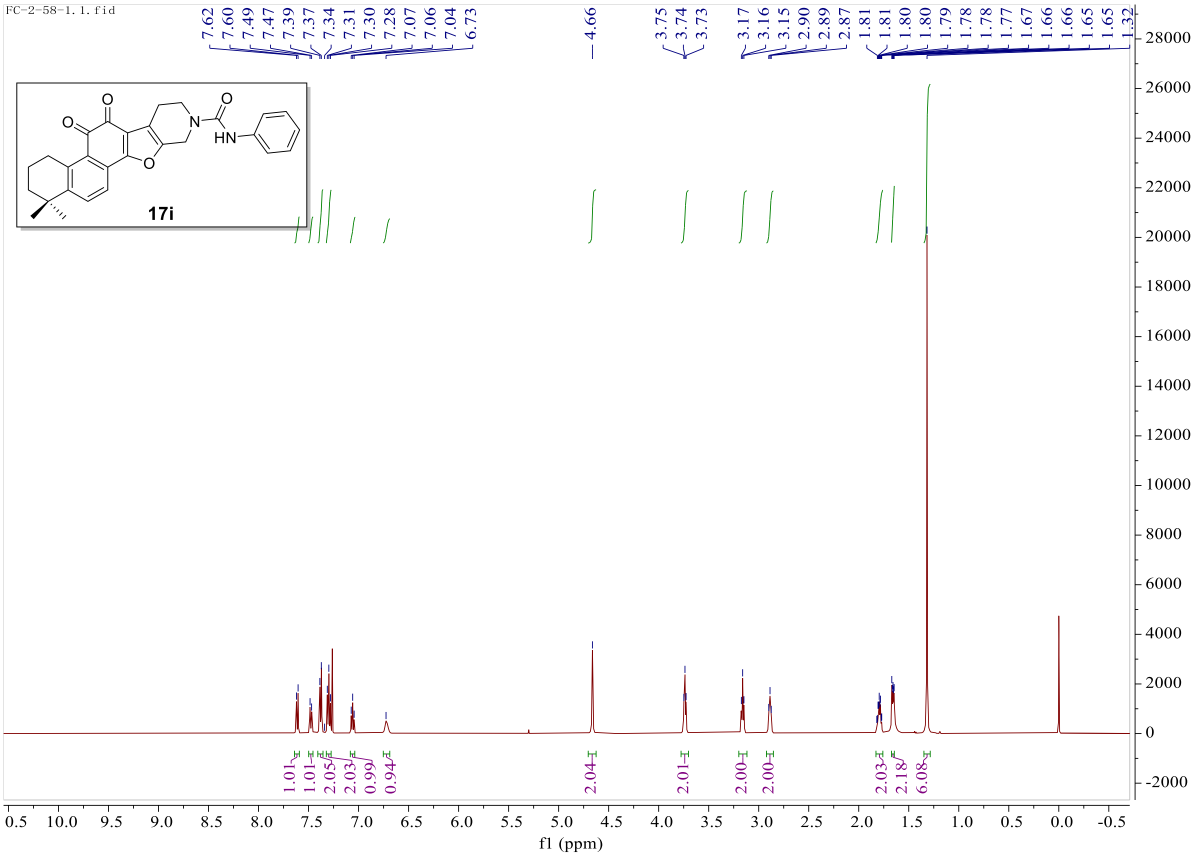
FC-2-58-1.1.fid
7.62 7.60 7.49 7.47 7.39 7.37 7.34 7.31 7.30 7.28 7.07 7.06 7.04 6.73
4.66
3.75 3.74 3.73
3.17 3.16 3.15 2.90 2.89 2.87
1.81 1.81 1.80 1.80 1.79 1.78 1.78 1.77 1.67 1.66 1.66 1.65 1.65 1.32
17i
1.01 1.01 2.05 2.03 0.99 0.94 2.04 2.01 2.00 2.00 2.03 2.18 6.08
f1 (ppm)

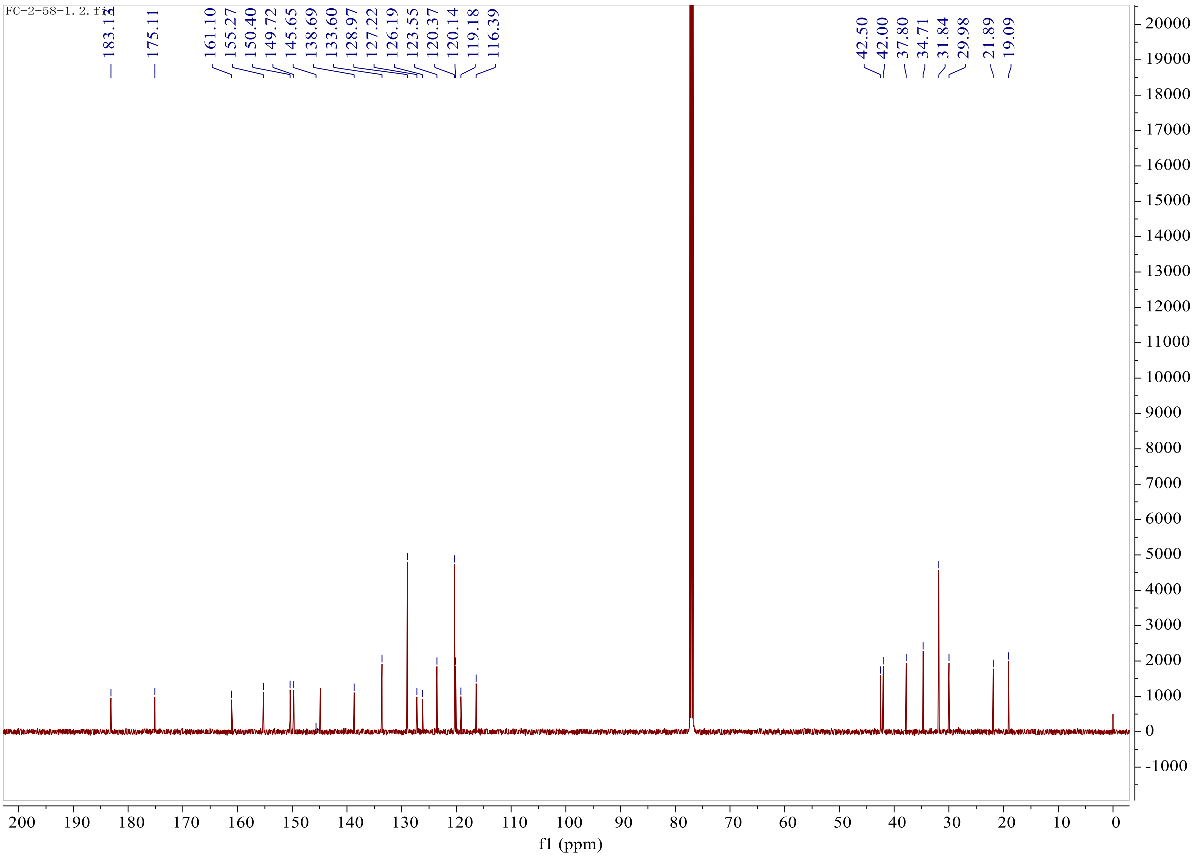
FC-2-58-1.2.fid
183.13
175.11
161.10 155.27 150.40 149.72 145.65 138.69 133.60 128.97 127.22 126.19 123.55 120.37 120.14 119.18 116.39
42.50 42.00 37.80 34.71 31.84 29.98 21.89 19.09
f1 (ppm)

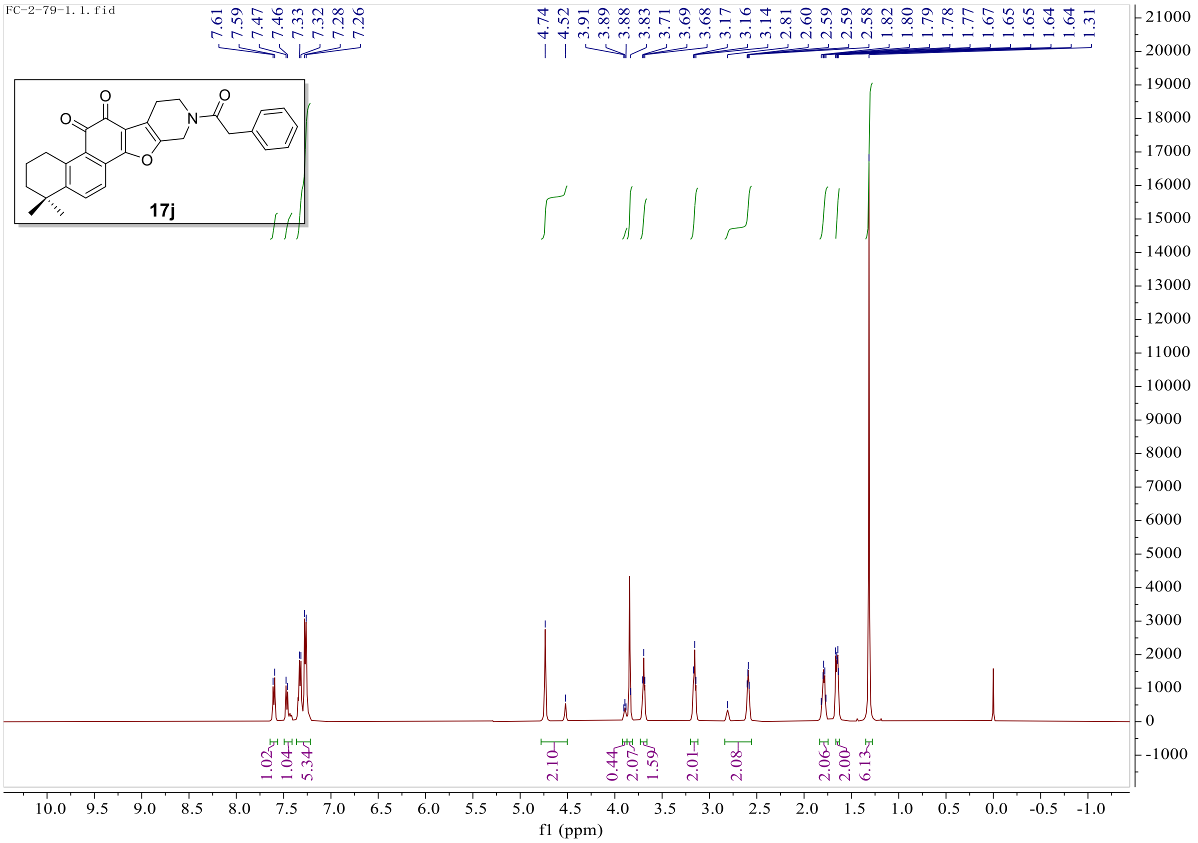
FC-2-79-1.1.fid
7.61 7.59 7.47 7.46 7.33 7.32 7.28 7.26
4.74 4.52 3.91 3.89 3.88 3.83 3.71 3.69 3.68 3.17 3.16 3.14 2.81 2.60 2.59 2.59 2.58 1.82 1.80 1.79 1.78 1.77 1.67 1.65 1.65 1.64 1.64 1.31
17j
1.02 1.04 5.34 2.10 0.44 2.07 1.59 2.01 2.08 2.06 2.00 6.13
f1 (ppm)

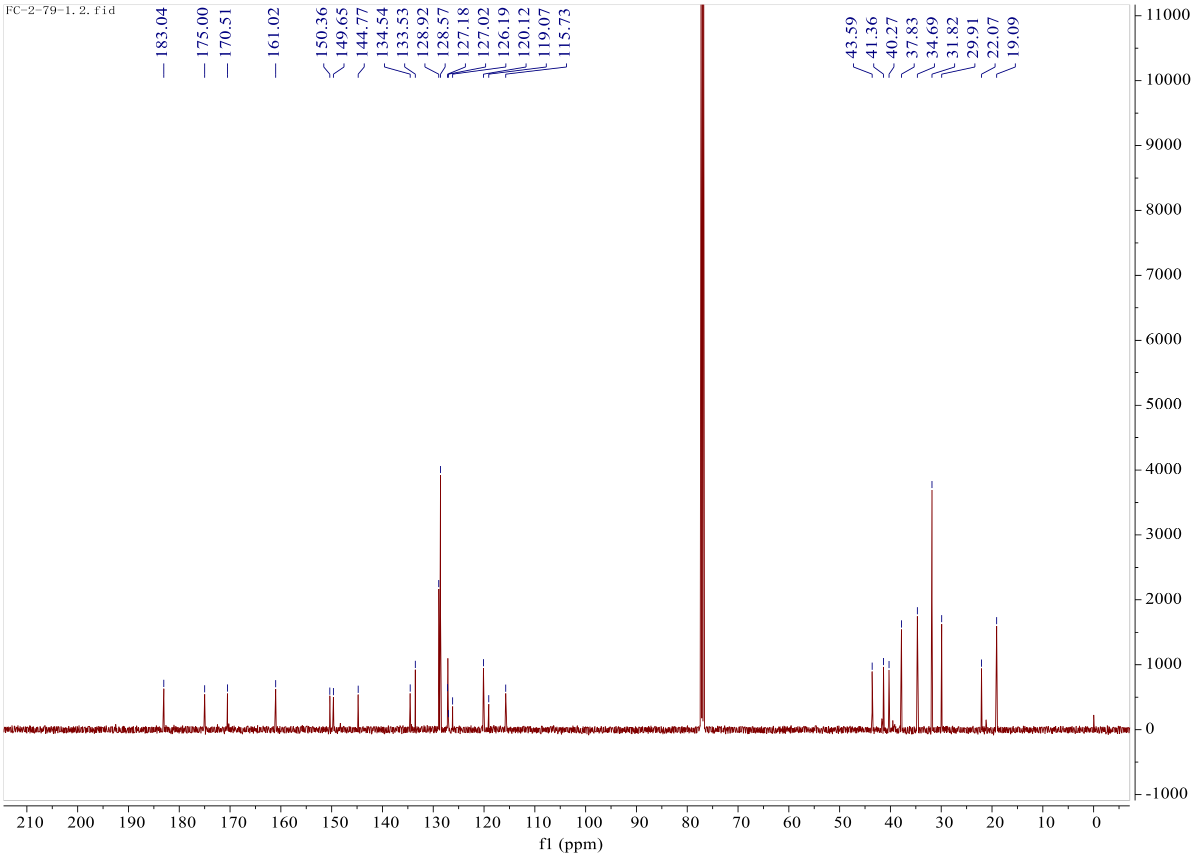
FC-2-79-1.2.fid
183.04 175.00 170.51 161.02 150.36 149.65 144.77 134.54 133.53 128.92 128.57 127.18 127.02 126.19 120.12 119.07 115.73
43.59 41.36 40.27 37.83 34.69 31.82 29.91 22.07 19.09
f1 (ppm)

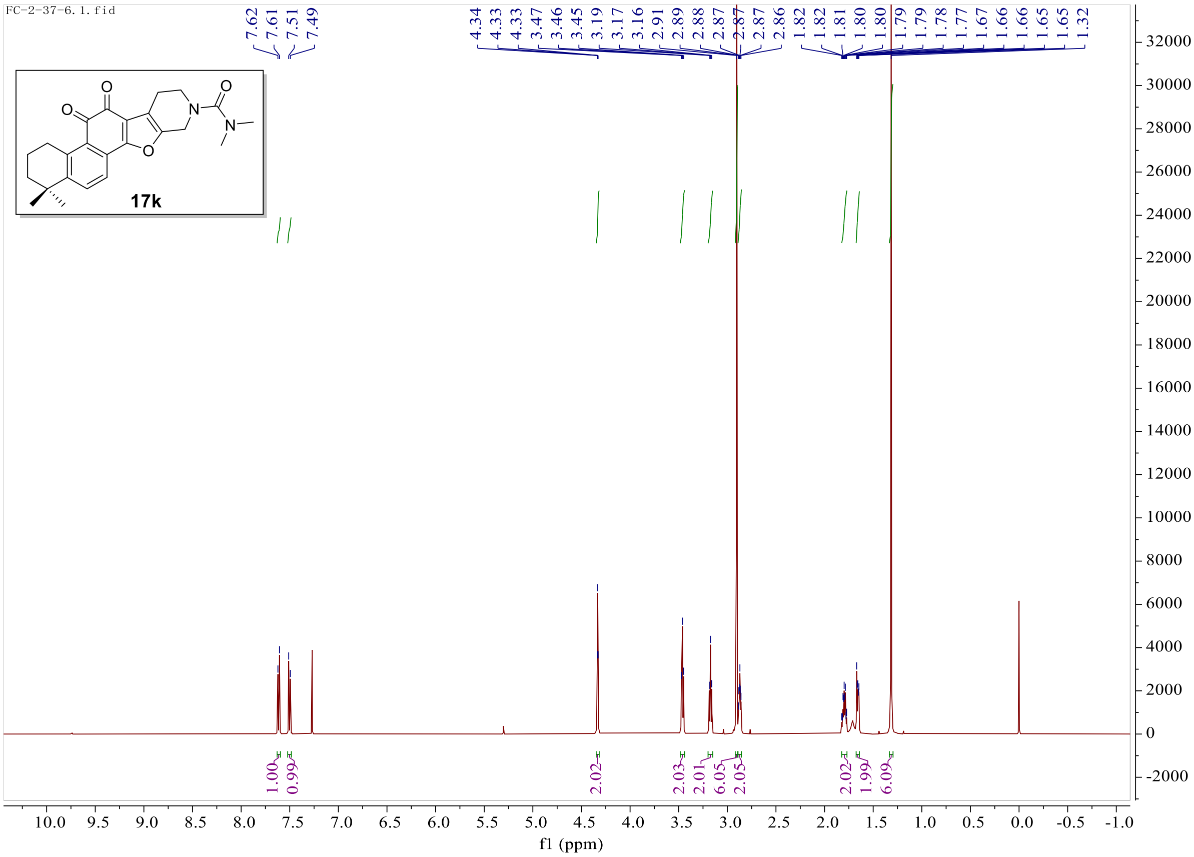
FC-2-37-6.1.fid
7.62
7.61
7.51
7.49
4.34
4.33
4.33
3.47
3.46
3.45
3.19
3.17
3.16
2.91
2.89
2.88
2.87
2.87
2.87
2.86
1.82
1.81
1.80
1.80
1.79
1.79
1.78
1.77
1.67
1.66
1.66
1.65
1.65
1.32
17k
1.00
0.99
2.02
2.03
2.01
6.05
2.05
2.02
1.99
6.09
f1 (ppm)

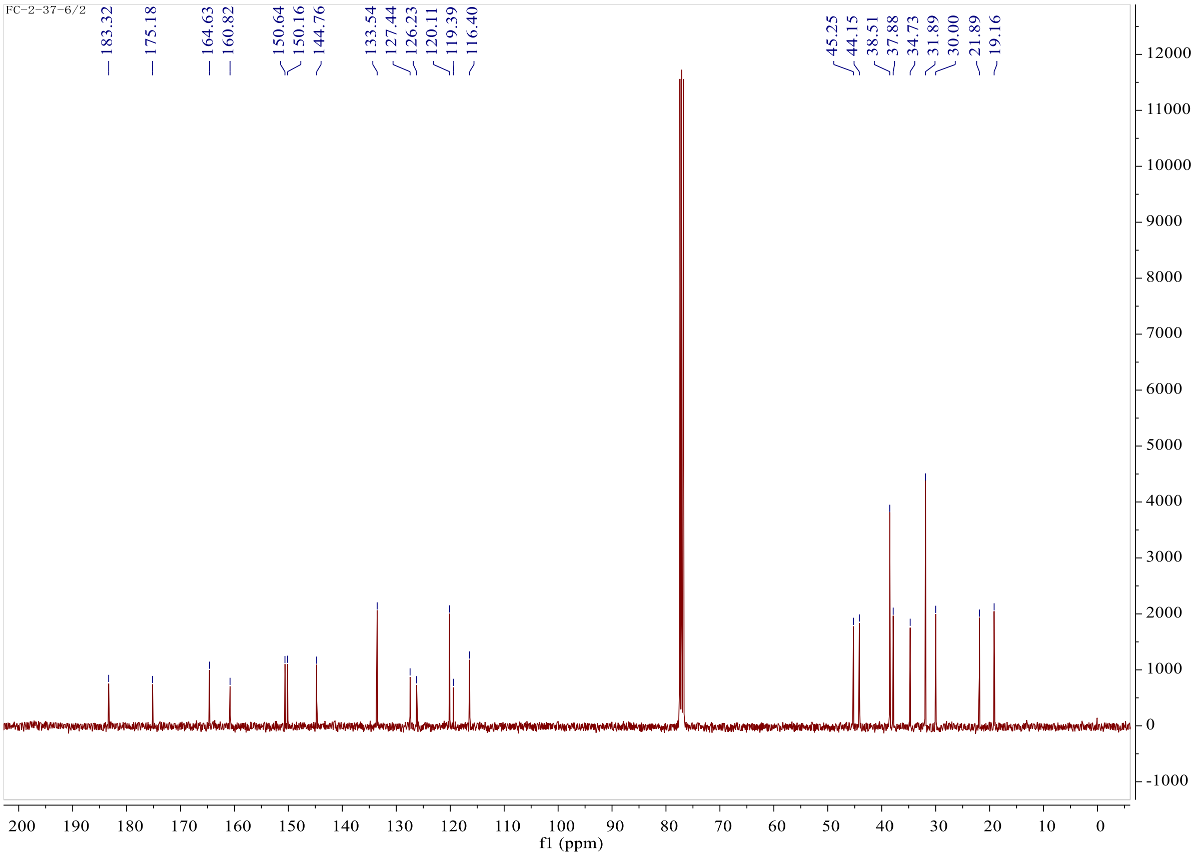
FC-2-37-6/2
183.32
175.18
164.63
160.82
150.64
150.16
144.76
133.54
127.44
126.23
120.11
119.39
116.40
45.25
44.15
38.51
37.88
34.73
31.89
30.00
21.89
19.16
f1 (ppm)

5. Figures

X-Ray Crystallographic Data

Compounds **3** and **5b** were crystallized from petroleum ether / EtOAc. Intensity data for the two compounds were collected on zju Bruker D8 Venture Ims3.0. The details of crystal data collection and refinement of **3** and **5b** are summarized in Table **S1**.

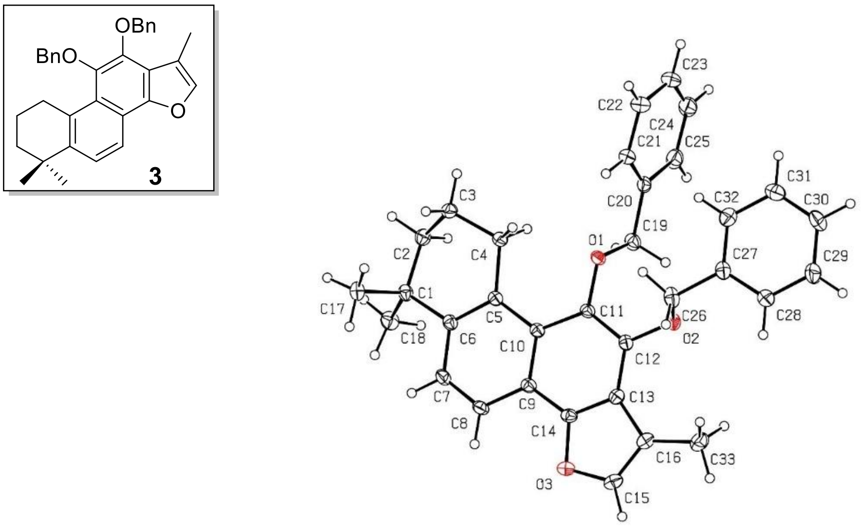

Figure S1. X-ray crystallographic structure of **3** (CCDC 2177105).

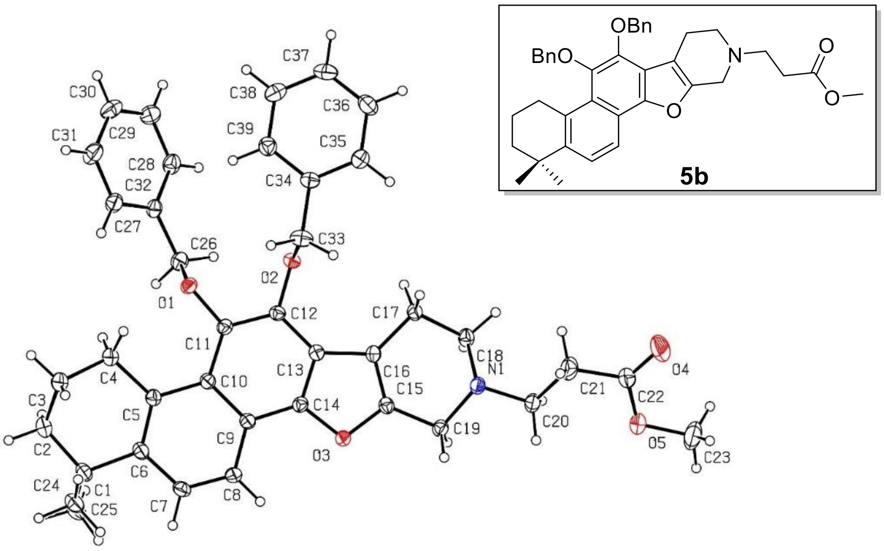

Figure S2. X-ray crystallographic structure of **5b** (CCDC 2153324).

Table S1. Crystal data and structure refinements for **3** and **5b**.

Compounds	**3**	**5b**
CCDC code	2177105	2153324

Empirical formula	$C_{33}H_{32}O_3$	C39H41NO5
Formula weight	476.58	603.73
Temperature / K	170	170.0
Crystal system	orthorhombic	triclinic
Space group	$P2_1/n$	P-1
a / Å	17.745(3)	9.6719(3)
b / Å	7.4025(14)	12.4125(4)
c / Å	20.990(4)	13.7892(4)
α / °	90	89.9120(10)
β / °	112.248(11)	83.5140(10)
γ / °	90	76.1200(10)
Volume / $Å^3$	2552.0(8)	1596.24(9)
Z	4	2
ρ_{calc} g / cm^3	1.240	1.256
μ / mm^{-1}	0.084	0.082
F (000)	1016.0	644.0
Crystal size / mm^3	0.48 × 0.23 × 0.19	0.4 × 0.23 × 0.19
Radiation	5.578 to 136.526	MoKα (λ = 0.71073)
Index ranges	-20 ≤ h ≤ 21, -8 ≤ k ≤ 8, -25 ≤ l ≤ 25	-12 ≤ h ≤ 12, -15 ≤ k ≤ 15, -17 ≤ l ≤ 17
Reflections collected	38287	26751
Independent reflections	4664 [R_{int} = 0.0307, R_{sigma} = 0.0204]	7054 [R_{int} = 0.0258, R_{sigma} = 0.0243]
Dat/restraints/parameters	4664/0/328	7054/0/409
Goodness-of-fit on F^2	1.040	1.026
Final R indexes [I>=2σ (I)]	R_1 = 0.0392, wR_2 = 0.0995	R_1 = 0.0428, wR_2 = 0.1096
Final R indexes [all data]	R_1 = 0.0408, wR_2 = 0.1011	R_1 = 0.0509, wR_2 = 0.1157
Largest diff. peak/hole/e$Å^{-3}$	0.16 / -0.27	0.33 / -0.24

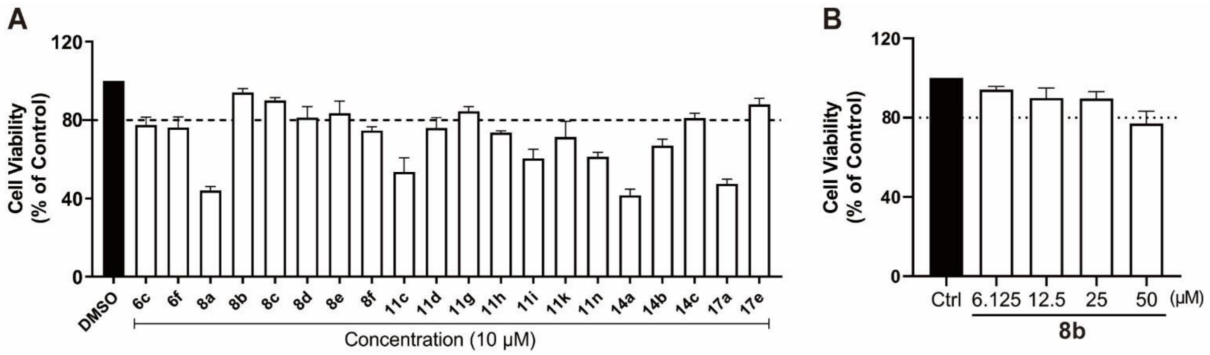

Figure S3. The cytotoxicity evaluation in mice peritoneal macrophages analyzed by an MTT assay. (A) Macrophages (per well: 1×10^4) were seeded in 96-well plates and treated with compounds at concentration of 10 μM. (B) Cytotoxicity evaluation of **8b** against macrophages at different concentrations. Data was presented as mean ± SD (n = 3).

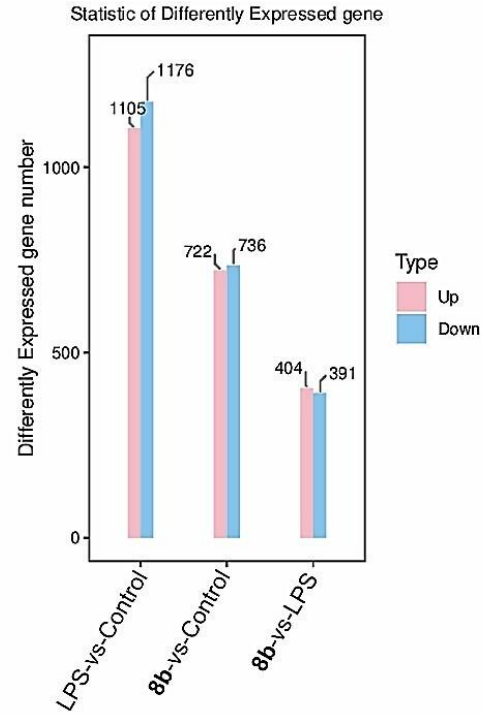

Figure S4. Statistic of differently expressed genes.

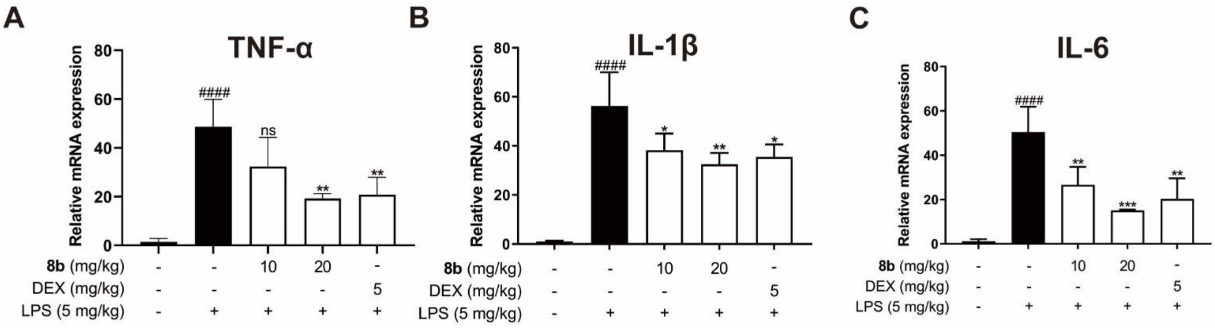

Figure S5. Effects of **8b** on the LPS-induced mRNA expression of cytokines. (A - C) The mRNA levels of TNF-α, IL-1β, IL-6 in LPS-induced mice lung tissues. All data were presented as means ± SD (n = 3; * vs LPS, # vs Con, * P <0.05; ** P < 0.01; *** P < 0.001; **** P < 0.0001; #### P < 0.0001; ns, no significant vs LPS). Significant difference was calculated by one-way ANOVA followed by Bonferroni's multiple comparisons test.

Table S2. Pharmacokinetic parameters for compound **8b**.

Parameter[a]	PO[b]	IP	IV
Dosage (mg/kg)[c]	10	10	5
$T_{1/2}$ (h)[c]	2.1	1.5	1.4
$AUC_{0\text{-}t}$ (h·μg/L)	72.6	925.1	981.9
C_{max} (μg/L)	37.8	311.7	340.3
T_{max} (h)	0.51	0.11	0.08
F (%)	4	-	-

[a]Route of administration: PO, oral administration; IP, intraperitoneal injection; IV, intravenous administration, n = 3. [b]PO, IP, IV formulation: hydrochloride salt of **8b** was dissolved in distilled water. [c]$T_{1/2}$, half-life; AUC, area under the plasma concentration time curve; T_{max}, time of maximum concentration; *F*, oral bioavailability.

Table S3. Lung tissue distribution of compound **8b.**

Tissue	Concentrations of **8b**[a]		
	0.25 h	2 h	12 h
Lung (ng/g)	9160	1790	421.12

[a]IP formulation: hydrochloride salt of **8b** was dissolved in distilled water and administration dosage is 10 mg/kg, n = 3.

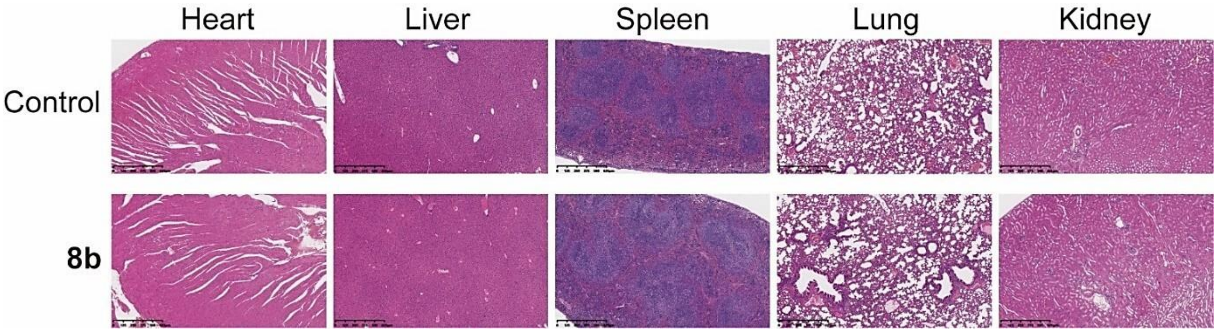

Figure S6. Histopathological changes induced by **8b** in mice. Tissues of mice treated with physiological saline as Control group and treated with compound **8b** at 200 mg/kg (n = 3).
